# Genomic consequences of dietary diversification and parallel evolution due to nectarivory in leaf-nosed bats

**DOI:** 10.1093/gigascience/giaa059

**Published:** 2020-06-06

**Authors:** Yocelyn T Gutiérrez-Guerrero, Enrique Ibarra-Laclette, Carlos Martínez del Río, Josué Barrera-Redondo, Eria A Rebollar, Jorge Ortega, Livia León-Paniagua, Araxi Urrutia, Erika Aguirre-Planter, Luis E Eguiarte

**Affiliations:** 1 Departamento de Ecología Evolutiva, Instituto de Ecología, Universidad Nacional Autónoma de México (UNAM), Ciudad Universitaria, 04510 Coyoacán, Mexico City, Mexico; 2 Red de Estudios Moleculares Avanzados, Instituto de Ecología AC, 91070 Xalapa, Veracruz, Mexico; 3 Department of Zoology and Physiology, University of Wyoming, 82071 Laramie, Wyoming, USA; 4 Centro de Ciencias Genómicas, Universidad Nacional Autónoma de México, 62210 Cuernavaca, Morelos, Mexico; 5 Departamento de Zoología, Laboratorio de Bioconservación y Manejo, Posgrado en Ciencias Quimicobiológicas, Instituto Politécnico Nacional-ENCB, 11340 Mexico City, Mexico; 6 Facultad de Ciencias, Universidad Nacional Autónoma de México, Ciudad Universitaria, 04510 Coyoacán, Mexico City, Mexico; 7 Departamento de Ecología Funcional, Instituto de Ecología, Universidad Nacional Autónoma de México (UNAM), Ciudad Universitaria, 04510 Coyoacán, Mexico City, Mexico

**Keywords:** Adaptation, Comparative genomics, Diet, Parallel evolution, Phyllostomid, Specialization

## Abstract

**Background:**

The New World leaf-nosed bats (Phyllostomids) exhibit a diverse spectrum of feeding habits and innovations in their nutrient acquisition and foraging mechanisms. However, the genomic signatures associated with their distinct diets are unknown.

**Results:**

We conducted a genomic comparative analysis to study the evolutionary dynamics related to dietary diversification and specialization. We sequenced, assembled, and annotated the genomes of five Phyllostomid species: one insect feeder (*Macrotus waterhousii*), one fruit feeder (*Artibeus jamaicensis*), and three nectar feeders from the Glossophaginae subfamily (*Leptonycteris yerbabuenae, Leptonycteris nivalis*, and *Musonycteris harrisoni*), also including the previously sequenced vampire *Desmodus rotundus*. Our phylogenomic analysis based on 22,388 gene families displayed differences in expansion and contraction events across the Phyllostomid lineages. Independently of diet, genes relevant for feeding strategies and food intake experienced multiple expansions and signatures of positive selection. We also found adaptation signatures associated with specialized diets: the vampire exhibited traits associated with a blood diet (i.e., coagulation mechanisms), whereas the nectarivore clade shares a group of positively selected genes involved in sugar, lipid, and iron metabolism. Interestingly, in fruit-nectar–feeding Phyllostomid and Pteropodids bats, we detected positive selection in two genes: *AACS* and *ALKBH7*, which are crucial in sugar and fat metabolism. Moreover, in these two proteins we found parallel amino acid substitutions in conserved positions exclusive to the tribe Glossophagini and to Pteropodids.

**Conclusions:**

Our findings illuminate the genomic and molecular shifts associated with the evolution of nectarivory and shed light on how nectar-feeding bats can avoid the adverse effects of diets with high glucose content.

## Background

Evolutionary shifts related to changes in feeding habits are considered one of the most important events in animal evolution [1]. Diet changes open new ecological and physiological opportunities [1, 2]. These shifts often involve changes in feeding behavior, dramatic innovations in the mechanism by which nutrients are assimilated and metabolized, and sometimes drastic morphological modifications [3]. Evolutionary diet shifts are sometimes accompanied by species diversification and adaptive functional trait radiation [4].

The New World leaf-nosed bats (family Phyllostomidae) are one of the most species-rich mammalian taxa, with 216 species in 60 genera [5, 6]. Leaf-nosed bats evolved from an insect-feeding common ancestor and now display a large and diverse spectrum of feeding habits that include insectivory, carnivory, frugivory, blood feeding, nectar-pollen feeding, and omnivory [5–7]. Moreover, dietary specializations and species diversification seem to be correlated in these bats [7, 6].

Although most extant Phyllostomids are insectivorous or omnivorous [6, 7], two lineages have extreme dietary specialization: blood feeding within the subfamily Desmodotinae (including *Desmodus, Diphylla*, and *Diademus*) and the nectar-pollen–feeding species within the subfamily Glossophaginae (including *Leptonycteris, Glossophaga, Choeronycteris*, and *Musonycteris*), which feed primarily on nectar and pollen [5, 6]. Among these nectar-pollen–feeding species, *Leptonycteris yerbabuenae* (lesser long-nosed bat) is notable owing to its tight co-evolutionary interactions with plants and seeming specialization to nectarivory/pollinivory [8–10]. The blood feeder *Desmodus rotundus* and the two other species in the Desmodotinae subfamily have a feeding mode unique among mammals [11]. Data on the genome and microbiome of *D. rotundus* have revealed remarkable adaptive changes genes associated with blood diet [12].

Many studies have demonstrated evidence of evolutionary novelties associated with feeding diversification in leaf-nosed bats [6, 13–15]. These include morphological traits involved in nectar extraction [10], and physiological characteristics related to the processing of a diet high in sugars [13, 14]. However, the genomic signatures associated with dietary diversification and specialization during the evolution of Phyllostomid bats from an insect-feeding common ancestor remain largely unknown.

**Figure 1:**
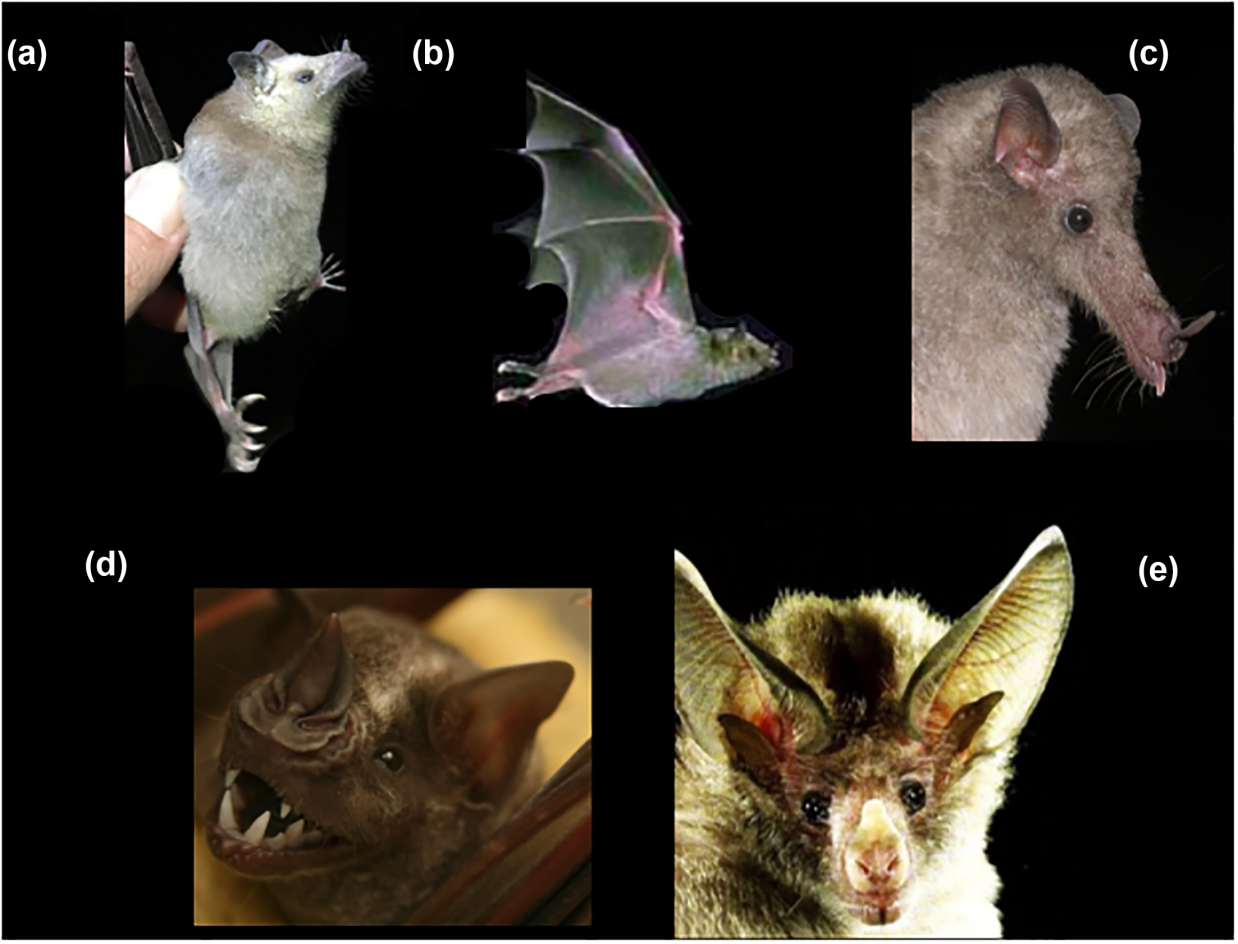
Phyllostomid species. (a) *Leptonycteris yerbabuenae*, (b) *Leptonycteris nivalis*, (c) *Musonycteris harrisoni*, (d) *Artibeus jamaicensis*, and (e) *Macrotus waterhousii*. Photo credits: (a-b) Daniel Zamora-Mejías, (c) Rodrigo Medellín-Legorreta, d) Melissa E. Rodríguez, and (e) Wikimedia, public domain.

We investigated the genomic and evolutionary dynamics associated with the dietary diversification and nectar-pollen–feeding specialization of Phyllostomid bats. We sequenced and assembled the whole genomes of five Phyllostomid bat species, including ecologically and economically important species. We sequenced the genomes of three nectar-pollen feeders *L. yerbabuenae, Leptonycteris nivalis*, and *Musonycteris harrisonii*; the fruit feeder *Artibeus jamaicensis*; and the insect feeder *Macrotus waterhousii* (Fig. 1). For comparative purposes our analyses incorporated genomic data of the vampire *Desmodus rotundus* [12] and other mammals.

Our research was guided by three sets of predictions. First, we predicted that the dietary diversification from insectivory, which is the ancestral condition in the group, to derived diets would be accompanied by evolutionary changes in relevant genes involved in food uptake and the metabolic pathways associated with the processing of assimilated nutrients. Second, we predicted that the dietary specializations observed in the subfamilies Desmodotidae and Glossophaginae would be correlated with evidence of selection in genes that facilitate the assimilation and metabolism of components of blood and nectar, respectively. More specifically, we expected the nectar-pollen feeder lineage to show adaptive signals in genes involved in sugar assimilation and metabolism. Our third prediction was that we should detect convergent evolution between the New World fruit and nectar feeders and the Old World fruit bats in genes important for carbohydrate metabolism.

We adopted a hierarchical approach: we examined our predictions in deep nodes of the phylogeny, then we identified the nodes that represent dietary transitions and investigated the changes that accompanied these transitions. We conducted a genomic comparative approach and performed a phylogenomic reconstruction to identify expansions/contractions of gene families across the Phyllostomids lineage. We also evaluated orthologous protein sequences that have been targets of selection and their relation to dietary diversification and specialization. Finally, to identify convergent evolutionary signals associated with the diet, we carried out a comparison between the genomes of the nectar-pollen–feeding Phyllostomid bats and the Old World fruit-feeding bats (family Pteropodidae), analysing radical amino acid substitutions in conserved positions.

## Data Description

We sequenced the genome of one adult male lesser long-nosed bat (*L. yerbabuenae*) by means of a whole high-throughput shotgun strategy and obtained a high-quality *de novo* assembly (104×) (Table 1; see Supplementary Tables S1 and S11). Additionally, we sequenced with medium coverage (∼24–56×) the genomes of four Phyllostomid bats: *M. waterhousii* (insect feeder), *A. jamaicensis* (fruit feeder), and the nectar-pollen feeders *M. harrisoni* and *L. nivalis*. (Fig. 1; see Supplementary Table S1).

**Table 1:** Global statistics for the nectar-pollen–feeding bat *L. yerbabuenae* genome assembly

Statistic	Value
**Sequencing**	
Total raw data (Gb)	254.4
No. Reads > Phred 30	690,759,531
Coverage (×)	103.6
**Assembly**	
Contig	
N50 (kb)	69.49
L50 (kb)	8,805
No.	78,626
Longest (Mb)	0.55
Total size (Gb)	2.05
Scaffold	
N50 (kb)	14,735.1
L50 (kb)	38
No.	34,419
Longest (Mb)	70.81
Total size (Gb)	2.05
BUSCO No. (%)	Completed
Completed	3,864 (94.1)
Fragmented	103 (2.5)
Missing	141 (3.4)
**Annotation**	
No. of Exons	119,036
No. of CDS/proteins	24,074
Repeats	
No.	3,010,348
Length (Mb)	547.05
%	26.64

CDS: coding sequence.

## The genomic landscape of New World leaf-nosed bats

The size of *L. yerbabuenae’*s genome was similar to those reported for other bats (2.05 Gb), with an N50 scaffold length of 14,735,151 bp, and L50 of 38 scaffolds (Table 1). Evaluation of the genome assembly for completeness based on BUSCO identified 94% of complete and 2.5% of fragmented genes from the mammalian database (Mammalia *odb9*). The genome contained 24,074 inferred coding sequences from an *ab initio* prediction, the transcript evidence, and homology evidence obtained from a set of proteins of several mammalian species (Table 1; see Supplementary Table S2). Approximately 26% of the genome assembly was composed of repetitive elements (547.05 Mb length) (see Supplementary Table S3).

We constructed a reference guide genome assembly based on *L. yerbabuenae* for the other four Phyllostomid bats, where we annotated from 18,000 to 24,471 coding sequences and proteins for each Phylllostomid (Table   2; see Supplementary Tables S4 and S5; Supplementary Fig. S1).

**Table 2:** Mapping statistics and single-nucleotide polymorphism identification in Phyllostomid bat genomes (based on *L. yerbabuenae* genome assembly)

Parameter	*Leptonycteris nivalis*	*Musonycteris harrisoni*	*Artibeus jamaicensis*	*Macrotus waterhousii*
**Diet**	Nectar-pollen	Nectar-pollen	Fruits	Insects
Total data (Gb)	131.4	69.2	56.6	128.4
Coverage (×)	54.8	30.45	25	56.34
BUSCO (%)				
Complete	93.7	94.0%	93.0%	93.3%
Fragmented	2.8	2.4%	3.5%	3.4%
Missing	3.5	3.6%	3.5%	3.3%
**No. of CDS/proteins**	24,471	20,135	18,756	19,171

CDS: coding sequence.

## Analyses

### Gene family evolution reflects distinct dietary needs

**Figure 2:**
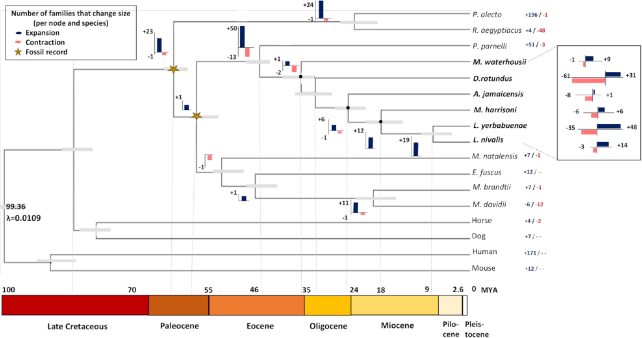
Phylogenetic tree constructed with 132 single-copy genes and estimates of divergence times based on 2 fossil records (yellow stars) (see Methods). Based on 22,388 gene families we analysed the number of orthologous families expanded (plus signs, blue) and contracted (minus signs, salmon) across the phylogeny: per node (bars) and per species branch (right), with a *P-*value ≤ 0.01. Gray bars reflect the divergence time interval based on 95% highest posterior density. MYA: million years ago.

To understand genomic evolution and to trace changes associated with dietary diversification and specialization, we reconstructed a phylogenomic tree using 132 single-copy orthologous genes (61,331 amino acid sites), which was calibrated using two fossil dates [16–18]. Based on the phylogenomic tree, we analysed the dynamics (expansion and contractions) for 22,388 gene families across the Phyllostomid bat genomes (Fig. 2 and Table 3).

**Table 3:** GO enrichment for significant gene families per node and habit food

Species and nodes	Function and metabolic pathway	GO	*P-*value < 0.01
Phyllostomid node			
Expansions	+ Structural constituent of ribosome	GO:0003735	<1e−30
	+ Translation	GO:0006412	<1e−30
Contractions	− Hydrolase activity	GO:0016788	0.00099
	− **Lipid metabolic process**	**GO:0006629**	**0.001**
	− Aspartic-type endopeptidase activity	GO:0004190	1.8e−06
*D. rotundus*			
Expansions	+ Response to biotic stimulus	GO:0009607	5.7e−12
	+ Defense response	GO:0006952	7.8e−12
	+ Signal transduction	GO:0007165	1.3e−08
	**+ Nitrate assimilation**	**GO:0042128**	**8.8e−08**
	**+ Regulation of appetite**	**GO:0032098**	**0.00034**
	+ Protein glycosylation in Golgi	GO:0033578	0.00313
	+ GTPase activity	GO:0003924	<1e−30
	+ Molybdenum ion binding	GO:0030151	4.5e−05
Contractions	− Translation	GO:0006412	1e−30
	− **Calcium ion transmembrane**	**GO:0070588**	**1.3e−16**
	− **Cellular calcium ion homeostasis**	**GO:0006874**	**3.5e−09**
	− Neuron development	GO:0048666	4.2e−09
	− Microtubule-based process	GO:0007017	5.5e−08
	− Homophilic cell adhesion via plasma	GO:0007156	1.7e−07
	− Peptidyl-prolyl cis-trans isomerase	GO:0003755	<1e−30
	− Ephrin receptor activity	GO:0005003	2.7e−21
	− Ryanodine-sensitive calcium channel	GO:0005219	6.2e−21
	− Inorganic anion exchanger activity	GO:0005452	2.1e−20
	− Ionotropic glutamate receptor activity	GO:0004970	6.3e−20
	− Voltage-gated calcium channel activity	GO:0005245	5.6e−16
*A. jamaicensi, M.harrisoni, L. yerbabuenae*, and *L. nivalis*			
Expansions	+ Translation	GO:0006412	<1e-30
	+ Integral component of membrane	GO:0016021	6.7e-06
	+ Immune response 458	GO:0006955	1.1e-05
	**+ Iron ion import membrane**	**GO:0098711**	**0.00018**
	**+ HFE-transferrin receptor complex**	**GO:1990712**	**5.7e-05**
	**+ Transferrin receptor binding**	**GO:1990459**	**7.3e-05**
Contractions	− Protein peptidyl-prolyl isomerization	GO:0000413	<1e-30
*M. harrisoni, L. yerbabuenae*, and *L. nivalis*			
Expansions	**+ Protein deubiquitination**	**GO:0016579**	**2.8e-09**
	+ Virion assembly 24	GO:0019068	0.00027
	+ Structural constituent of ribosome	GO:0003735	<1e-30
	+ Thiol-dependent ubiquitinyl hydrolase	GO:0036459	3.6e-11
	**+ Transferrin receptor binding**	**GO:1990459**	**0.0099**
	**+ HFE-transferrin receptor**	**GO:1990712**	**0.0099**
	**+ Iron ion import membrane**	**GO:0098711**	**0.0056**

Gene Ontologies (GO) annotations involved in metabolism and diet are in boldface (plus sign: gene family expansions; minus sign: contractions). GTP: guanosine triphosphate.

For all the Phyllostomid bats, the significant gene family enrichment functions were related to the cellular repair process and genetic make-up for protein synthesis. Furthermore, across the Phyllostomid bats many gene families exhibited changes with feeding habits, e.g., the Phyllostomid node had a contraction related to the lipid metabolism. The blood feeder lineage had a significant gain on gene families involved in the regulation of appetite and process for nitrogen acquisition, but this lineage also showed many contraction events involved in calcium metabolism (Table 3). The fruit- and nectar-feeding bats exhibited many expansion events in iron metabolism regulation pathways (Table 3).

### Rapidly evolving genes across the whole genome

**Figure 3:**
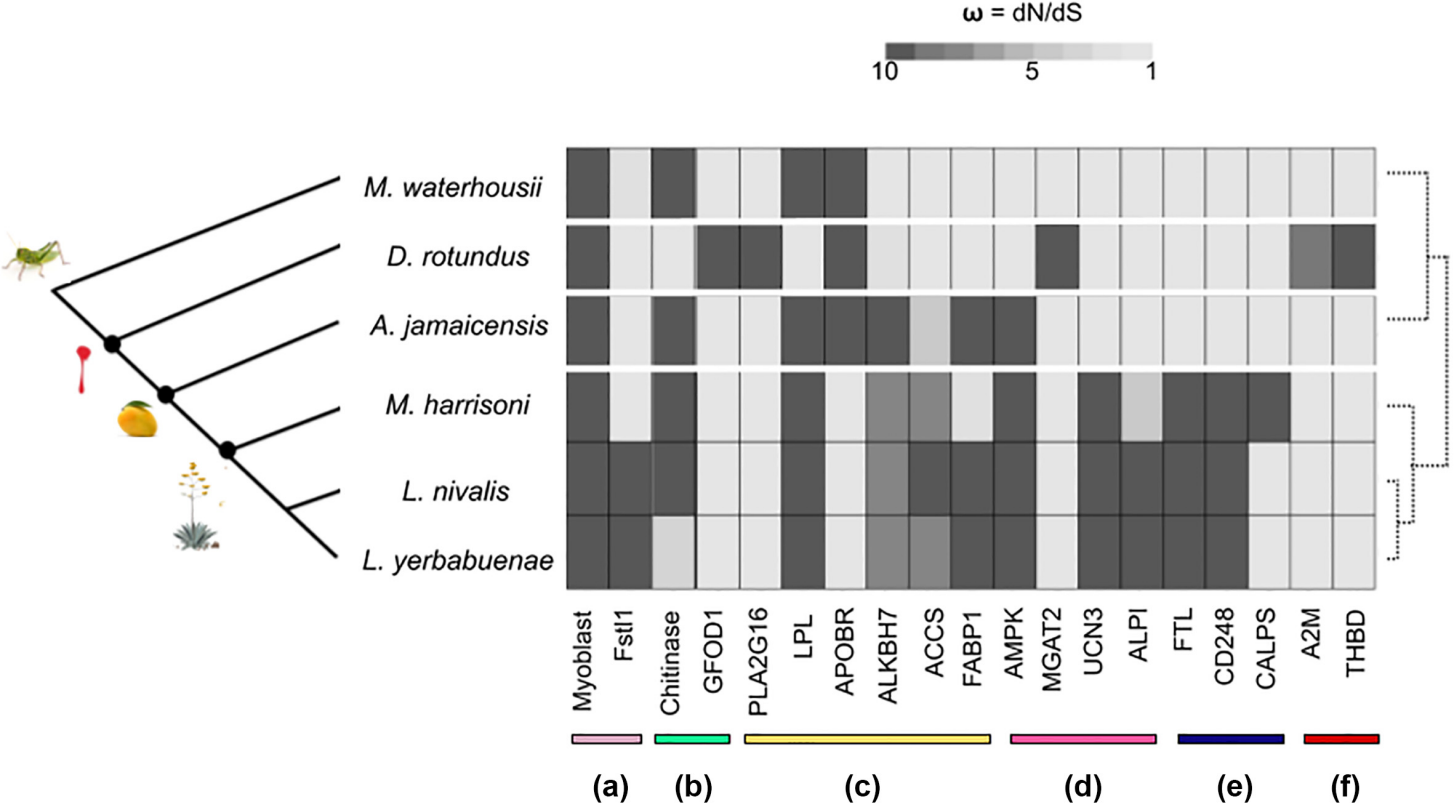
Positive selection in genes and proteins across the phylogeny of Phyllostomid bats, in comparison to the insect-feeding bat *Macrotus waterhousii*. Most of the positive selected genes likely contribute to the regulation and processing of (a) muscle and bone development, (b) carbohydrates, (c) lipids, (d) nutrients and food uptake, (e) iron storage and calcium sources, and (f) blood regulation (see gene and protein abbreviations in Supplementary Table S14).

For all Phyllostomid bats, we identified 42 genes with robust signals of positive selection (false discovery rate [FDR] *P* < 0.05). In accordance with the enrichment analysis, most of the adaptive genes are related to immune response, DNA repair, inflammatory response, RNA catalytic process, and genes that mediate muscle function (such as *Myoblast*and*PAMR1*) (Fig. 3; see Supplementary Tables S6–S8) [19].

### Ecological and feeding behavior adaptations across the Phyllostomid lineages

To understand shifts associated with dietary diversification, we analysed genes under positive selection involved in the mechanisms of carbohydrate digestion and lipid metabolism in each Phyllostomid species (Fig. 3).

In *M. waterhousii*, an insect feeder, we found evidence of positive selection in *Chitinase*, which codes for proteins in the degradation of insect exoskeleton [20] (Fig. 3). Interestingly, for *M. waterhousii*, the *Trehalase* is a partial gene that exhibited signals of positive selection, but for the rest of the Phyllostomid species, *Trehalase* is a pseudogene. This finding is relevant because trehalose is the principal sugar in insects' blood.

The vampire's genome revealed a complex set of genes crucial for maintaining a blood-feeding diet under positive selection, including *THBD* (hematopoietic cell pathway) and *A2M* (complement and coagulation cascade pathway) [21]. Only in the vampire did we find under positive selection genes involved in feeding and lipid-cholesterol metabolism such as *MGAT2, PLAS2G16*, and *GFOD1* [22, 23] (Fig. 3). Interestingly, the vampire was the only genome where the *Trehalase* gene was completely missing.

In the fruit bat *A. jamaicensis* most of the enzymes analysed involved in lipid and carbohydrate metabolic pathways showed positive selection pressures (Fig. 3; see Supplementary Table S8). The importance of these enzymes for this fruit bat might reflect the diversity of its diet because *A. jamaicensis* has been documented to eat, besides insects and fruits, seeds and leaves [24].

**Figure 4:**
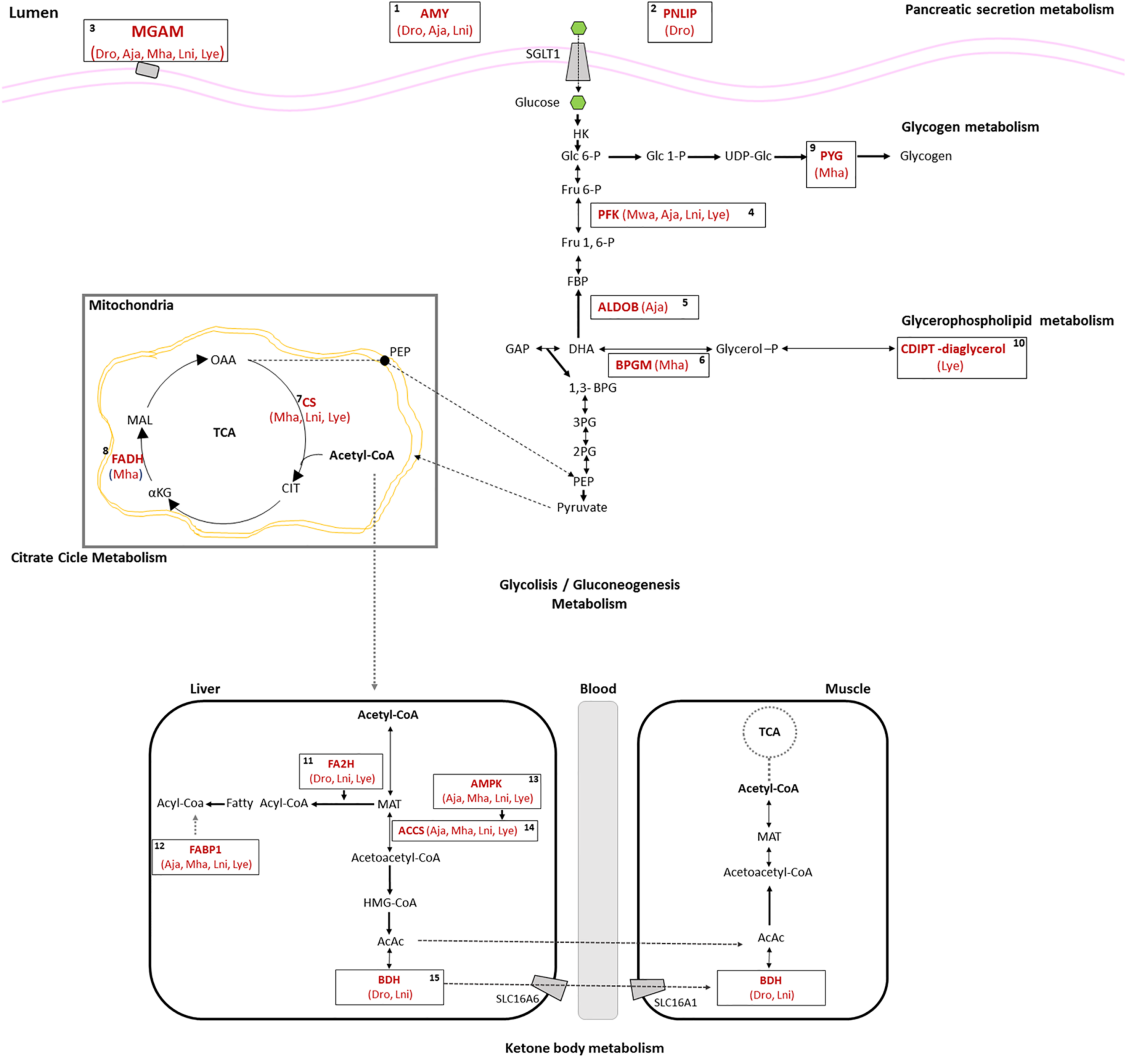
A subset of genes under positive selection (in red boldface) that are involved in glucose and ketone metabolism in the frugivorous (Aja: *A. jamaicensis*) and nectar-pollen bats (Mha: *M. harrisoni*; Lni: *L. nivalis*; and Lye: *L. yerbabuenae*). The diagram also identifies adaptative signals for some genes in the vampire *D. rotundus* (Dro) and the insectivore *M. waterhousii* (Mwa). The diagram is based on the KEGG metabolic pathways database and a review of the literature (see gene and protein abbreviations in Supplementary Table S8).

Every nectar-pollen feeder bat species (*M. harrisoni, L. nivalis*, and *L. yerbabuenae*) showed positive selection signatures for genes involved in insulin secretion (*UCN 3*) [25], calcium and iron storage (*CALP2, CD248*, and *FTL*) [26, 27], bone morphogenetic regulation (*Fslt1*) [28], and in *IAP*, the gene coding for the mucosa defense factor involved in proper gut homeostasis (Fig. 3) [29]. Interestingly, we found adaptive signatures for genes crucial for carbohydrate and lipid metabolic pathways, such as pancreatic secretion, glycolysis/gluconeogenesis, glycogen, glycerophospholipid, citrate acid metabolism, and ketone metabolism (Fig. 4; see Supplementary Table S8) [30–32].

Finally, for the nectar-pollen and fruit bat species, we detected strong selective pressures in four enzymes, *AACS* (which appears to participate in the regulation of lipid metabolism) [32], *ALKBH7* (which codes for a protein that appears to be involved in the regulation of body mass and fat content) [33], *FABP1* (regulates fatty acid trafficking and prevents lipotoxicity) [34], and *AMPK* (major regulator of cellular energy homeostasis) (Figs 3 and 4) [35].

### Adaptation and convergent signatures in fruit- and nectar-pollen–feeding bats

The sugar and lipid metabolism genes detected under positive selection for the fruit- and nectar-pollen–feeding Phyllostomid bats were also analysed in the genomes of three Old World bat species available in databases, including *Pteropus alecto, Pteropus vampyrus*, and *Rousettus aegyptiacus* (Family Pteropodidae). We found evidence of positive selection in *AACS* in *P. alecto, P. vampyrus*, and *R. aegyptiacus*. In *R. aegyptiacus*, we also found evidence of positive selection in *ALKBH7* (see Supplementary Table S8).

**Figure 5:**
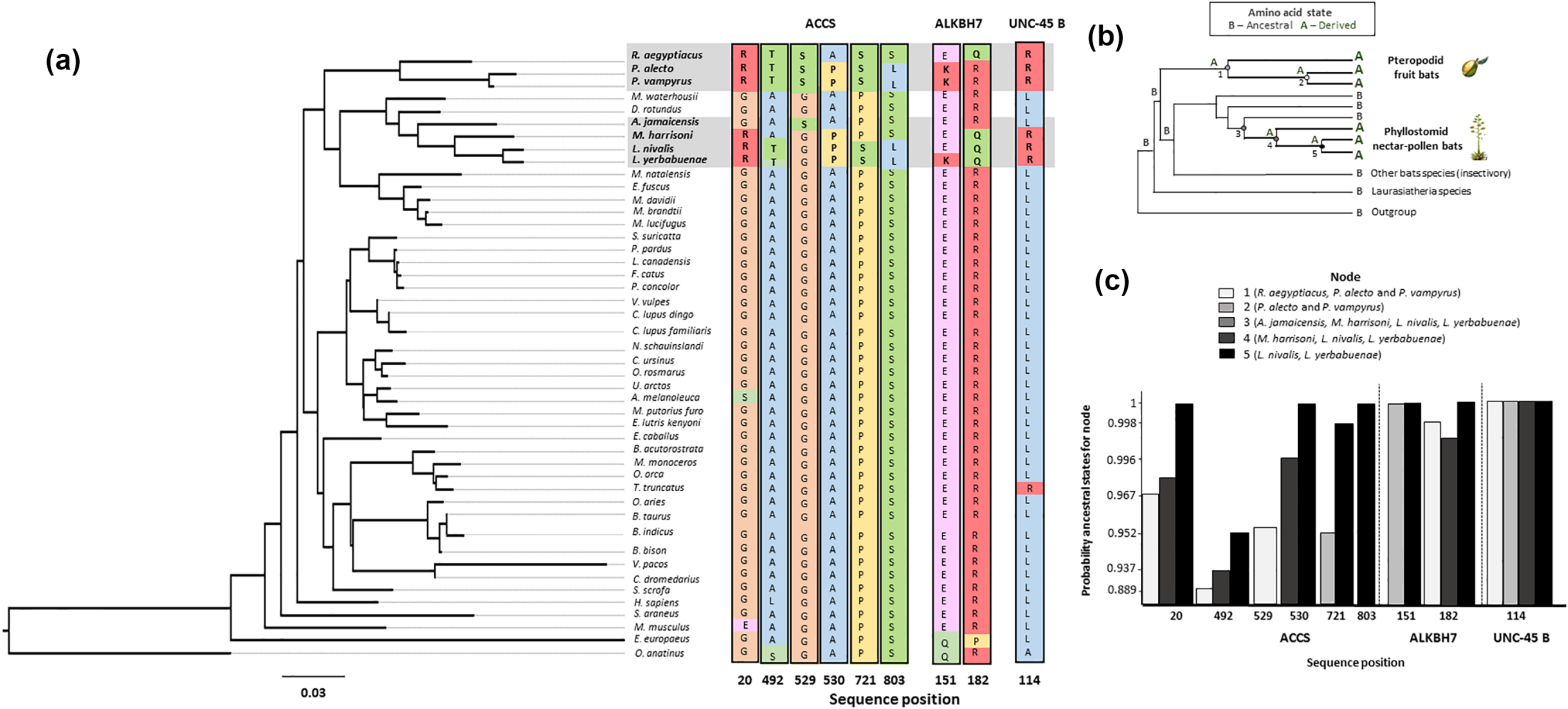
Parallel molecular evolution between Pteropodids (Old World) and Glossophagini (New World) bats, in three genes: *AACS, ALBKH7* and *UNC-45 B*. (a) Phylogeny reconstruction for these three genes by maximum likelihood (using 1,827 amino acids), for 47 mammal species. (b) Ancestral sequence reconstruction (for branches and nodes) to infer parallel substitutions in conserved positions for the three genes. (c) Probability of replacement at each ancestral state node for each sequence position. Amino acid abbreviations: A: alanine (non-polar); T: threonine (polar); Q: glutamine (polar); R: arginine (basic-charged); K: lysine (basic-charged); E: glutamic acid (acidic + charged); S: serine (polar); and L: leucine (non-polar).

We analysed 1,918 orthologous sequences and reconstructed the ancestral sequence states to identify some genes with unique and exclusively parallel substitutions for the Phyllostomid fruit bat, the Glossophagini and Pteropodid lineages, in a conserved position for the rest of the bats and mammal species (Fig.   5b). We found three genes with parallel signatures in a specific amino acid position. Most of these parallel changes presumably led to changes in the physicochemical properties of the expressed protein (Fig.   5a). In *AACS*, we identified six radical amino acid substitutions along the sequence, from an ancestral glycine (non-polar) to a derived arginine (positively charged), alanine (non-polar) to threonine (polar), glycine to serine (polar), alanine to proline (non-polar), serine to proline, and serine to leucine (non-polar) (Fig.   5a). In *ALKBH7*, we found a parallel amino acid substitution from glutamic acid (negatively charged) to lysine (positively charged) and arginine to glutamine (polar). The latter change was found in the Glossophagines and *R. aegyptiacus* (Fig. 5b). Finally, for the gene *UNC-45 B*, which codes for a protein involved in muscle cell development, we identified a substitution from leucine to arginine (Fig. 5a) [36]. Moreover, we calculated the posterior probabilities for each amino acid reconstruction state for each node along the three. For all nodes in the Glossophagini and Pteropodid clades, the probabilities of each amino acid derived state were >80% (Fig.   5c; see Supplementary Table S9).

**Figure 6:**
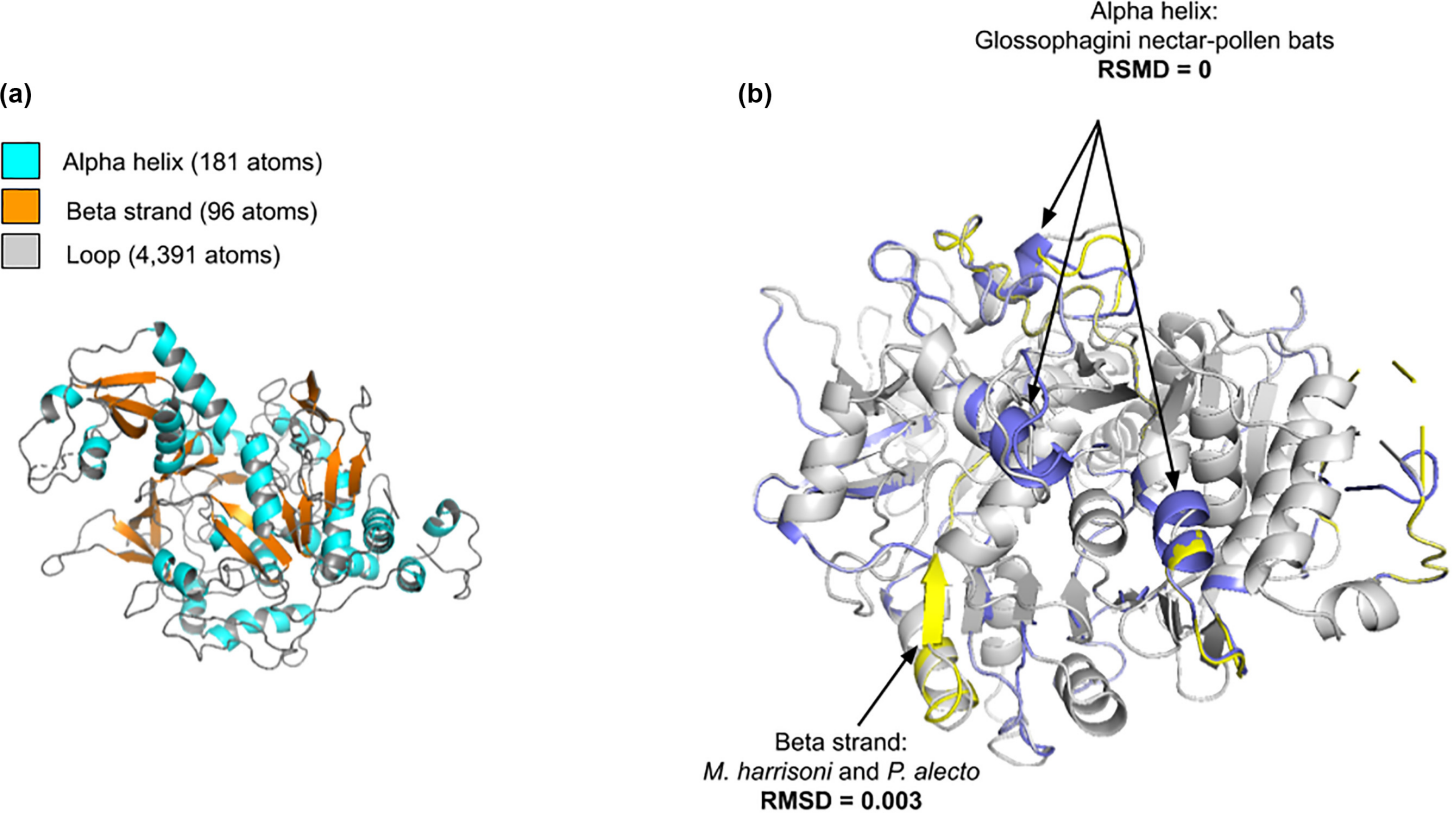
*ACCS* protein structure. (a) 3D structure of *ACCS* protein for *L. yerbabuenae*. (b) In gray: *ACCS* 3D structure consensus (*M. waterhousii* and *D. rotundus*). In blue: α-helix structures shared only for the 3 Glossophagini nectar-pollen feeders (*M. harrisoni, L. nivalis*, and *L. yerbabuenae*). In yellow: β-strand shared only between *M. harrisoni* and *P. alecto* (Pteropodid bat). RMSD score (protein 3D superposition and alignment) between pairs of species (see Supplementary Table S10).

Finally, to evaluate whether the radical amino acid substitutions had affected the protein structure of *acetoacetyl co-enzyme A synthetase* (*ACCS*) , we modelled its 3D protein structure using hidden Markov models, for the nectar feeders *M. harrisoni, L. nivalis*, and *L. yerbabuenae*; the fruit bat *P. alecto*; and *D. rotundus* and *M. waterhousii* (Fig. 6a; see Supplementary Table S10). The *ACCS* protein structure is composed of 662 amino acids, two domains, and 96 atoms of β-strand, 181 atoms of α-helix, and 4,391 loop atoms (Fig.   6a). Moreover, we performed a multi-comparison of the 3D structure for all the species mentioned above (see Supplementary Table S11). We found high similarity in the 3D protein structure for all bats, according with the Root-mean Square Deviation of Atomic Position (RMSD) values from 0 to 0.003. However, we identified three residues of α-helix shared only for the Glossophaginae clade, and a β-strand shared only between *M. harrisoni* and *P. alecto* (Fig. 6b; see Supplementary Table S11).

## Discussion

Our study provides unprecedented knowledge on the genomic signatures behind the dietary diversification and specialization in Phyllostomid bats. Surprisingly, and contrary to our first prediction, we found that many of the genomic characteristics of the ancestral Phyllostomid diet remained functional in all lineages of the family. For example, the *Chitinase* gene was functionally conserved in most genomes (with the exception of the vampire), highlighting the relevance of digestion and nutrient uptake from insects in all lineages, including those that mainly feed on fruit, nectar-pollen, and even in some cases, those that feed only on blood (Figs 3 and 4) [9, 10, 20]. However, as previous studies suggest, we found that *Trehalase* (involved in the digestion of trehalose from insect blood, comprising ∼7% of their dry mass) is missing or a pseudogene for all the blood-, fruit-, and nectar-feeding bats, which may be a result of dietary diversification in the family [37].

Our second prediction was supported: we found unique genomic specializations in bats with obligate and restrictive diets, such as the vampire and the nectar-pollen feeders (Figs 3 and 4, Table 3). The vampire's genome has unique characteristics associated with the ability to consume blood, including genes that play a crucial role in the down-regulation of fibrinolysis and those that control the production of blood cells (Fig. 4) [12, 21, 38]. On the other hand, we also detected strong positive selection in genes crucial for carbohydrate oxidation, ATP production, and in genes involved in ketone metabolism in the three Glossophagini bats. These are associated with the extreme energetic feat of feeding on the wing [14, 30, 39] (Fig. 4). Our analyses also highlight the importance of genes involved in iron storage for animals that feed on iron-deficient sources. These results might be related to the avoidance of metabolic disorders such as anemia (Fig. 4) [28]. The results also help to explain how bats that feed on nectar-pollen can avoid the potentially adverse effects of their peculiar diet. In humans, loss of function due to mutations in some of these genes is associated with nutrient malabsorption and metabolism disorders including diabetes, hyperglycemia, and obesity [30, 39].

In support of our third prediction, we identified signatures of molecular parallel evolution shared by fruit-feeding Pteropodid and nectar-pollen–feeding Glossophagini bats (Fig. 5a and b). The ancestral sequence reconstruction provided us with insights into the mechanisms of molecular adaptation and functional divergence. Signals of parallel evolution and adaptative selection for the proteins *ACCS* and *ALKBH7* shed light on the importance of the storage of fatty fuels necessary to meet the energy demands of an expensive mode of foraging and pollinator ecology of these specialist bats. Protein function is more likely to be affected if genes show many radical substitutions in conserved positions, and signal of positive selection. In spite of the evolutionary changes detected for *ACCS* in the Glossophagini species, their tertiary protein structure exhibited high similarity when we compared it with other bats (see Supplementary Table S11). However, we identified exclusive differences in *α*-helix and *β*-strand regions (Fig. 6b) that may be important in the protein function for the nectar-pollen feeders and the Old World fruit bat (Fig. 6). We consider it very probable that *ACCS* is up- or down-regulated. Future studies must evaluate the expression levels for this gene, and its regulation, including replicating and analysing more tissues, such as the gut and liver [40, 41].

Surprisingly, the protein *UNC-45 B* exhibited the same amino acid substitution between Glossphagini, Pteropodid, and the dolphin. Lee et al. [42] have identified genes involved in muscle skeletal function and movement, with parallel substitutions shared between bats and marine mammals (such as dolphin, whale, and baiji). We suggest that the protein *UNC-45 B* may be implicated in an efficient mobility and superfast muscle physiology for these species [42].

On the other hand, the molecular traits that we infer as the result of parallel evolution were not found in the fruit-eating bat *A. jamaicensis*. We hypothesize that this species should be considered more omnivorous than strictly frugivorous [24, 41]. Omnivory-frugivory might have been an important step in the transition to a more restricted fruit diet and to a nectar-pollen diet (Figs 4 and 5) [5–8]. To explore this hypothesis further, it will be necessary to expand our sample of genomes to include more Phyllostomid species that have more exclusively frugivorous diets than *A. jamaicensis*.

Our findings suggest that parallel evolution due to nectar-feeding dietary specialization is likely a consequence of high metabolic demands required for foraging on flowers and fruits. These results are notable, given that the Pteropodidae and Glossophaginae lineages are separated by >60 million years [8] (see Supplementary Fig. S2) [17]. Moreover, our results shed light on the evolutionary mechanisms and genomic shifts that take place in the transition to novel feeding habits. They also shed light on the genomic changes that take place when animals adopt a diet dominated by sugar consumption, and with low levels of lipids and proteins. The generality of inferences can be tested in other nectar-specialized vertebrate taxa, such as hummingbirds [40].

Finally, we found differences in the evolution of gene families and genes that are not necessarily or only related to diet among Phyllostomid lineages (Fig. 2 and Table 3). These differences are likely associated with other lineage-specific aspects of physiology, ecology (e.g., niche resources, interactions, immune system), and microbiomes [5, 6, 12, 43]. As an example, positive selection in the *IAP* enzyme was only detected in the nectar-feeding bats, which supports a strong relationship between dietary specialization and the bacterial communities that are involved in providing vitamins and aiding digestive processes (Fig. 4) [12]. Future analyses should address the relationship between host diet and intestinal bacterial community, and the evolution of microbiomes across dietary diversification and specialization.

## Methods

### Animal sampling and genome sequencing

An adult male *Leptonycteris yerbabuenae* (NCBI:txid700936) was collected and processed on site at the cave “El Salitre” in Morelos state, Mexico (18 44.467 N, 99 10.767 W). All procedures were carried out in accordance with Federal Mexican Procedures (Guidelines of Secretaría de Medio Ambiente y Recursos Naturales, SEMARNAT), permit SGPA/DGVS/07161/15. The Zoology Museum “Alfonso L. Herrera” (Facultad de Ciencias, UNAM) donated the tissue samples from four leaf-nosed bats: *Macrotus waterhousii* (NCBI:txid124750)*, Artibeus jamaicensis* (NCBI:txid9417), *Leptonycteris nivalis* (NCBI:txid59456), and *Musonycteris harrisoni* (NCBI:txid148053) (Fig. 1; see Supplementary Table S1).

For all leaf-nosed bat species, we isolated their DNA using the Phenol-Chloroform protocol and DNA Blood and Tissue Kit (Qiagen). We used the Illumina HiSeq 4000 150 PE platform to sequence the genomes (see Supplementary Table S1). We paid special attention to *L. yerbabuenae*, in order to use it as a reference to help in the assembly construction of the other genomes. In this species, we performed high-throughput whole-genome sequencing (the DNA sample was sequenced on 2 lanes). Additionally, we used the fresh samples collected from *L. yerbabuenae* to obtain transcriptional evidence for the genome annotation; we extracted the RNA-sequencing (RNA-Seq) from five tissues: brain, pancreas, kidney, lung, and liver (reserved in a buffer storage of RNA stabilization) using the RNeasy Mini Kit (Qiagen). All tissues with RIN values ≥ 8 were sequenced on Illumina HiSeq 4000 150 PE platform.

### *L. yerbabuenae* genome assembly

#### De novo genome assembly

The genome assembly was constructed *de novo* with Platanus (Platanus, RRID:SCR_015531) v. 2.4.3 [44], using a heterozygous value = 0.04 (-u 0.04) and an initial *k*-mer = 32. To optimize and extend the genome assembly, we performed a scaffolding with MeDuSa software [45, 46]. Finally, we used Pilon (Pilon, RRID:SCR_014731) for correcting bases and polishing the genome assembly [47].

We evaluated the genome assembly metrics (total length, number of scaffolds, number of contigs, L50, N50, and others). Moreover, with BUSCO (BUSCO, RRID:SCR_015008) v3 and the Mammalia odb9 database [48] we evaluated the measure for quantitative assessment of the gene content into the genome assembly.

#### Gene prediction

We performed TEdenovo from the REPET package [49] to predict, identify, and annotate the transposable elements (TEs) using the repetitive elements database Repbase [50]. We masked the TEs across the genome using RepeatMasker (RepeatMasker, RRID:SCR_012954) v4.0.7 (see Supplementary Table S3) [51].

We also cleaned, filtered, and assembled the RNA-Seq data of five tissues (brain, pancreas, kidney, liver, and lung) for the same individual with Trinity (Trinity, RRID:SCR_013048) v4.4.7 [52]. On the basis of the transcriptome assembly, we identified open reading frames, coding sequences, and their corresponding proteins [53].

We generated an *ab initio* gene prediction using Augustus (Augustus: Gene Prediction, RRID:SCR_008417) v2.5.5 [54]. To train Augustus, we used the gene structures of *Eptesicus fuscus* bat, and the RNA-Seq evidence (transcripts annotated from *L. yerbabuenae*). We performed a functional annotation by blastp using the UniProtKb SwissProt dababase and InterProScan [55, 56].

### New World leaf-nosed bats reference genome construction (assembly and annotation)

We used the *L. yerbabuenae* assembly as a reference genome to construct the assembly of *M. waterhousii, A. jamaicensis, M. harrisoni*, and *L. nivalis*.

All the raw data were filtered and cleaned (using a Phred score ≥30) (see Supplementary Table S1). We followed the GATK (GATK, RRID:SCR_001876) v2.07 pipeline to identify single-nucleotide variants (SNVs) [57]. First, to find all the SNVs along the genomic information from each Phyllostomid, based on the *L. yerbabuenae* genome assembly, all the high-quality genomic reads of each Phyllosomid were mapped to the *L. yerbabuenae* genome assembly with BWA-MEM (BWA, RRID:SCR_010910) [58]. Second, we used the GATK and Picard (Picard, RRID:SCR_006525) tools to recalibrate the genome mapping and identified the SNVs for each Phyllostomid (SortSam, MarkDuplicates, AddOrReplaceReadGroups, BuildBamIndex and CreateSequenceDictionary, RealignerTargetCreator, IndelRealigner, HaplotypeCaller, VariantFiltration, SelectVariants, BaseRecalibrator, AnalyzeCovariates, PrintReads, and VariantFiltration) (see Supplementary Fig. S3; Supplementary Methods) [56, 59]. We constructed the consensus sequence on the basis of the SNVs identified (with SAMtools, BCFtools, vcfutils.pl, and SeqtK) [60, 61]. We evaluated, for each consensus genome constructed, the assembly metrics and the integrity and gene content with BUSCO.

We predicted, identified, and masked the TEs for each consensus genome using the RepeatMasker v4.0.7 tool [50, 51]. We performed the gene prediction with Augustus. The genomes were annotated with blastp and InterProScan (InterProScan, RRID:SCR_005829) (see Supplementary Methods).

### Phylogenomic and gene family analysis

A total of 132 single-copy orthologous genes (61,331 amino acid sites) across 18 mammals were concatenated to reconstruct a phylogenomic tree (best-fit model distribution JTT, +G +I +I+G, and 80% consensus threshold) using PhyML3 [62] (see Supplementary Methods). We estimated molecular substitution rates with CODEML from the PAML (PAML, RRID:SCR_014932) package [63]. Based on a Bayesian phylogenetic method, with the MCMCtree tool, we estimated the species divergence times using fossil records from the genera *Icaronycteris* (∼50 million years ago) and *Tachypteron* with molecular ages of 64 million years ago [16–18].

We used CAFE [64] to analyse the statistical changes in the gene family sizes using a birth and death estimator (λ and μ). On the basis of the distribution of observed family sizes, we calculated the *P-*values for gene family expansions and contractions. We carried out the gene family annotation with shell and Perl scripts (see Supplementary Methods).

### dN/dS analysis using a branch-site model

#### Orthologous single-copy genes and filtering

We used the proteins annotated of *M. waterhousii, A. jamaicensis, M. harrisoni, L. nivalis*, and *L. yerbabuenae* to create a database, incorporating the complete set of proteins of all Laurasiatheria species available in the ENSEMBL database (35 species) and the protein information of all bat species available in the NCBI database (seven species) (see Supplementary Table S12). Based on a multi-species genome comparison with this database, we inferred the orthologous single-copy genes using the DIAMOND and Proteinortho programs [65, 66]. We extracted all the single-copy genes shared for each Phyllostomid bat, obtaining >9,637 single-copy gene clusters. We checked and removed all potential paralogous sequences and ambiguous amino acids (letter X). Each single-copy gene cluster was composed of from 8 to a maximum of 20 sequences.

Each cluster was aligned with the MAFFT (MAFFT, RRID:SCR_011811) aligner tool [67]. We retained alignment sequences where the length was within 80–120% relative to the human and mouse sequences, and poorly aligned regions were removed by means of a visual inspection. We used the alignments and their corresponding coding sequences to perform a robust conversion of protein multi-alignment into their corresponding codon alignments with PAL2NAL (see Supplementary Methods) [68]. We reconstructed the phylogenetic tree for each cluster with RAxML (parameters -m GTRGAMMA -p 12345) [69].

#### dN/dS test

We used the codon multi-alignment files and their corresponding phylogenetic tree to calculate synonymous sites and nonsynonymous sites (dN/dS) rates, using two bioinformatic tools: CODEML and HyPhy (HyPhy, RRID:SCR_016162) [63, 70].

With CODEML, we used a branch-site model, specifying the *foreground* branch (the species of our interest) and incorporating a null model (that assumes that the background and foreground branches share the same ratio [ω]). We designated each Phyllostomid species as the foreground branch of our interest, and we performed the CODEML analysis independently. For assigning significance, we constructed the likelihood ratio test (LRT) for each Phyllostomid result, using the likelihood values from the null and test model, and calculated the *P-*value ≤ 0.05 under a χ^2^ distribution. We also performed a *P-*value adjustment, using the FDR correction, based on the likelihood ratio. Additionally, we considered under positive selection all those sequence sites with a posterior probability >95% (by Bayes empirical Bayes method).

With HyPhy, we used aBSREL (adaptive branch-site random effects likelihood) [71]. aBSREL infers the optimal number of ω to test whether positive selection has occurred on a proportion of branches. The LRT is performed at each branch and compares the test model and null model. We inferred the optimal ω for all the branches for each single-copy gene cluster (including bats and non-bat species).

We retained and classified those adaptative genes that were identified in both programs (CODEML and HyPhy), with a *P*-value ≤ 0.05.

#### GO enrichment

We performed an enrichment analysis, using the weight01 algorithm with topGO v2.26 package from Bioconductor project in R [72, 73]. We obtained the statistical significance for the GO enrichment terms by performing the Fisher exact test (*P-*value ≤ 0.01).

## Radical amino acid substitution in conserved positions

### Ancestral sequence reconstruction

Based on the previously inferred orthologous genes, we extracted all single-copy genes shared by the Glossophagini: *M. harrisoni, L. nivalis*, and*L. yerbabuenae;* and the Pteropodids: *P. alecto, P. vampyrus*, and *R. aegyptiacus*. Each single-copy gene cluster was composed of between 12 and a maximum of 30 sequences.

We obtained 1,918 clusters of orthologous sequences (including ≥1 Glossophagini and 1 Pteropodid). Each cluster was aligned using PRANK [74], and we constructed their corresponding phylogenetic tree with RAxML (RAxML, RRID:SCR_006086) (parameter -m PROTCATLG) [69]. We checked all alignments for gaps and premature stop codons. We performed an ancestral sequence reconstruction using the protein alignments and phylogenetic trees. To dismiss incorrectly inferred residues and only retain the accurate ones for the reconstructed ancestral sequence, we used two different programs: CODEML, which assumes a Markov process model and calculates a Bayesian empirical likelihood for each character at each sequence position [63]; and FastML, which assumes a continuous time Markov process model and provides the posterior probabilities for each character at each sequence position [75]. Both programs provide the ancestral sequence and the posterior probabilities distribution. For CODEML, we fixed the parameters: model = 2, fix_alpha = 0, alpha = 0.5, and RateAncestor = 1.

On the basis of the ancestral sequence information, we identified pairs of branches for Glossophagini and Pteropodid species that exhibited a parallel amino acid substitution. We also checked the ancestral state at each node for these substitutions. We classified each parallel substitution as a radical amino acid substitution in a conserved position, assuming two criteria: (i) parallel substitution is exclusive in the branches of Glossophagini, Pteropodid, and their corresponding nodes; and (ii) different physicochemical properties between the most frequent amino acid state and the derived (parallel substitution).

We checked the Bayesian empirical likelihood at each ancestral state for all parallel substitution positions. We retained only those parallel substitutions with a posterior probability >85%.

Finally, we constructed a phylogeny using the information of those genes with parallel evolution. We concatenated the amino acid sequence for these three genes. We aligned the sequences with the MAFFT tool. We used ProtTest3 to select the best-fit model of protein evolution [76]. The phylogenetic tree was constructed using a maximum likelihood method with RAxML (-p 12345 -m PROTCATLG).

### Drivers of parallel evolution

In independent branches, mutation and selection can have equal impacts on patterns of parallel substitutions. For those proteins that exhibited parallel evolution, we also evaluated four variables at DNA and protein level: length, GC percent, rates at synonymous sites and nonsynonymous sites (dN/dS), and isoelectric point [77, 78].

## Protein Modelling

To explore the effects of positive selection and the radical amino acid substitutions, we modelled the secondary and tertiary structure of the protein *ACCS* for *M. waterhousii, D. rotundus, M. harrisoni, L. nivalis, L. yerbabuenae*, and *P. alecto*. We used Phyred 2 software [79], which compares the profile of the protein of our interest with a protein database using hidden Markov models and predicting secondary structure for each residue. To identify differences in the protein structure, we compared the secondary and tertiary structure between the nectar-fruit bats and *M. waterhousii* and *D. rotundus*, using the software PyMOL [80, 81]. To calculate the RMSD score, we aligned the Protein Data Bank (PDB) protein model between pairs.

## Availability of Supporting Data and Materials

Supporting data including genome assemblies, genome and protein annotation, TE prediction and annotation, multiFASTA gene families, phylogenomic alignment, ancestral sequence reconstruction, and tertiary protein modelling (PDB) files are available via the *GigaScience* database GigaDB [82].

The whole-genome and RNA-Seq sequence information analysed during the present study are available in the NCBI: whole-genome assembly for *L. yerbabuenae* within BioProject: PRJNA542899 and SRA: SRR9076597. The RNA-Seq data are available within Bioproject: PRJNA543325. Raw genome data of *M. waterhousii, A. jamaicensis, M. harrisonii*, and *L. nivalis* are available in the SRA: SRR908760, SRR9087866, SRR9089318, and SRR9089325, respectively.

## Additional Files

**Supplementary Table S1**. Raw Data Genome and RNA sequencing

**Supplementary Table S2**. *L. yerbabuenae*: de novo genome assembly statistics

**Supplementary Table S3**. TE prediction and annotation

**Supplementary Table S4**. Single Nucleotide Variants and Indels Metrics

**Supplementary Table S5**. Genome-guide statistics for each NW Leaf-nosed bat

**Supplementary Table S6**. LRT construction and *p-value* correction for sensory genes and metabolic enzymes

**Supplementary Table S7**. Genes under positive selection involved in Carbohydrates and lipid metabolism

**Supplementary Table S8**. GO enrichment for those positive selected genes for each Phyllostomid specie (dark gray- Biological Process; light gray- Molecular Function; and white – Celular Component)

**Supplementary Table S9**. Probability at each node at specific position (ancestral reconstruction sequence)

**Supplementary TableS10**. Protein 3D- structure alignments for the protein *ACCS*

**Supplementary Table S11**. 3D-structure model generated by Phyre2

**Supplementary Table S12**. Database: Laurasiatheria and other mammals

**Supplementary Table S13**. Drivers of parallel molecular evolution for the genes: *ACCS, ALKBH7* and *UNC-45 B*, for the fruit-bats (Pteropodids), pollen-nectar feeder bats (Glossophaginae), and other bats

**Supplementary Table S14**. Gene and proteins abbreviations

**Supplementary Figure S1**. a) Venn diagram showing the number of shared and unique Single Nucleotide Variants for each Phyllostomid bat based on *L. yerbabuenae* genome. b) Nucleotide diversity estimated across sliding windows of 1 Mbp for each Phyllostomid, based on *L. yerbabuenae*genome assembly.

**Supplementary Figure S2**. Phylogeny constructed based on 132 single copy genes and calibrated using three fossil records.

**Supplementary Figure S3**. Pipeline for the reference-genome assembly construction.

**Supplementary Figure S4**. *ACCS* 3D protein structure for each bat species.

**Supplementary Methods**.

giaa059_GIGA-D-20-00098_Original_Submission

giaa059_GIGA-D-20-00098_Revision_1

giaa059_GIGA-D-20-00098_Revision_2

giaa059_Response_to_Reviewer_Comments_Original_Submission

giaa059_Response_to_Reviewer_Comments_Revision_1

giaa059_Reviewer_1_Report_Original_Submission Graham Hughes -- 4/16/2020 Reviewed

giaa059_Reviewer_2_Report_Original_Submission Xiuguang Mao -- 4/17/2020 Reviewed

giaa059_Supplemental_Files

## Abbreviations

aBSREL: adaptive branch-site random effects likelihood; ACCS: acetoacetyl co-enzyme A synthetase; ATP: adenosine triphosphate; BLAST: Basic Local Alignment Search Tool; bp: base pairs; BUSCO: Benchmarking Universal Single-Copy Orthologs; BWA: Burrows-Wheeler Aligner; FDR: false discovery rate; GATK: Genome Analysis Toolkit; Gb: gigabase pairs; GC: guanine-cytosine; GO: Gene Ontologies; HyPhy: Hypothesis testing using Phylogenies; kb: kilobase pairs; KEGG: Kyoto Encyclopedia of Genes and Genomes; LRT: likelihood ratio test; MAFFT: Multiple Alignment using Fast Fourier Transform; Mb: megabase pairs; MCMCtree: Markov chain Monte Carlo tree; NCBI: National Center for Biotechnology Information; PAML: Phylogenetic Analysis by Maximum Likelihood; PDB: Protein Data Bank; RAxML: Randomized Axelerated Maximum Likelihood; RNA-Seq: RNA sequencing; SNV: single-nucleotide variant; SRA: Sequence Read Archive; TE: transposable element; RMSD: Root-mean Square Deviation of Atomic Position; UNAM: Universidad Nacional Autónoma de México.

## Ethics Approval and Consent to Participate

The use of animals in this study was performed in accordance with the Federal Mexican Procedures: Guidelines of Secretaría de Medio Ambiente y Recursos Naturales, SEMARNAT, with permit SGPA/DGVS/07161/15.

## Competing Interests

The authors declare that they have no competing interests.

## Funding

The study was supported by a grant from Fronteras de la Ciencia, “Genómica de la Diversidad de Vertebrados 1: *Leptonycteris* y la evolución de la nectarivoría en murciélagos y aves” (CONACyT, project No. 177) to L.E.E.

## Authors' Contributions

Y.T.G.G. and L.E.E. designed and performed research. Y.T.G.G. and L.E.E. wrote the manuscript with contributions from C.M.R., E.A.R., A.U., J.O., and E.A.P. E.I.L. helped with computational resources and bioinformatic analyses. J.B.R. helped with bioinformatic analyses. L.L.P. donated the samples. All authors revised and edited the manuscript.
