# Supplementary material for: Genomic consequences of dietary diversification and parallel evolution due to nectarivory in leaf-nosed bats
Source: Gigascience. 2020 Jun 6;9(6):giaa059. doi: 10.1093/gigascience/giaa059 (PMC7276932; doi:10.1093/gigascience/giaa059)
Supplement: giaa059_GIGA-D-20-00098_Original_Submission [file giaa059_giga-d-20-00098_original_submission.pdf]

GigaScience
Genomic consequences of dietary diversification and parallel evolution due to nectarivory in Leaf-nosed bats.
--Manuscript Draft--

**Manuscript Number:**	GIGA-D-20-00098
**Full Title:**	Genomic consequences of dietary diversification and parallel evolution due to nectarivory in Leaf-nosed bats.
**Article Type:**	Research
**Funding Information:**	Consejo Nacional de Ciencia y Tecnología (177) — Professor Luis E. Eguiarte
**Abstract:**	Background<br><br>The New World Leaf-Nosed bats (Phyllostomids) exhibit a diverse spectrum of feeding habits and innovations in their nutrient acquisition and foraging mechanisms. However, the genomic signatures associated with their distinct diets are unknown.<br><br>Results<br><br>We conducted a genomic comparative analysis to study the evolutionary dynamics related to dietary diversification and specialization. We sequenced, assembled and annotated the genomes of five Phyllostomid species: one insect-feeder (Macrotus waterhousii), one fruit-feeder (Artibeus jamaicensis), and three nectar-feeders from the Glossophaginae subfamily (Leptonycteris yerbabuenae, Leptonycteris nivalis and Musonycteris harrisoni), also including the previously sequenced Desmodus rotundus. Our phylogenomic analysis based on 22,388 gene families displayed differences in expansion and contraction events across the Phyllostomid lineages. Independently of diet, genes relevant for feeding strategies and food intake experienced multiple expansions and signatures of positive selection. We also found adaptation signatures associated with specialized diets: the vampire exhibited traits associated with a blood diet (i.e., coagulation mechanisms), whereas the nectarivore clade shares a group of positively selected genes involved in sugar, lipid, and iron metabolism. Interestingly, in fruit-nectar feeding Phyllostomid and Pteropodids bats, we detected positive selection in two genes: AACS and ALKBH7, which are crucial in sugar and fat metabolism. Moreover, in these two proteins we found parallel amino-acid substitutions in conserved positions exclusive to the tribe Glossophagini and to Pteropodids.<br><br>Conclusions<br><br>Our findings illuminate the genomic and molecular shifts associated with the evolution of nectarivory and shed light on how nectar-feeding bats can avoid the adverse effects of diets with high glucose content.
**Corresponding Author:**	Luis E.Eguiarte<br><br>MEXICO
**Corresponding Author Secondary Information:**	
**Corresponding Author's Institution:**	
**Corresponding Author's Secondary Institution:**	
**First Author:**	Yocelyn T. Gutiérrez-Guerrero, PhD Candidate
**First Author Secondary Information:**	
**Order of Authors:**	Yocelyn T. Gutiérrez-Guerrero, PhD Candidate
	Enrique Ibarra-Laclette, Doctor
	Carlos Martínez del Río, Doctor
	Josué Barrera-Redondo, PhD Candidate

Powered by Editorial Manager® and ProduXion Manager® from Aries Systems Corporation

	Eria A. Rebollar, Doctor
	Jorge Ortega, Doctor
	Livia León-Paniagua, Doctor
	Araxi Urrutia, Doctor
	Erika Aguirre-Planter, Doctor
	Luis E. Eguiarte, Doctor
**Order of Authors Secondary Information:**	
**Additional Information:**	
**Question**	**Response**
Are you submitting this manuscript to a special series or article collection?	No
**Experimental design and statistics**<br><br>Full details of the experimental design and statistical methods used should be given in the Methods section, as detailed in our Minimum Standards Reporting Checklist. Information essential to interpreting the data presented should be made available in the figure legends.<br><br>Have you included all the information requested in your manuscript?	Yes
**Resources**<br><br>A description of all resources used, including antibodies, cell lines, animals and software tools, with enough information to allow them to be uniquely identified, should be included in the Methods section. Authors are strongly encouraged to cite Research Resource Identifiers (RRIDs) for antibodies, model organisms and tools, where possible.<br><br>Have you included the information requested as detailed in our Minimum Standards Reporting Checklist?	Yes

Powered by Editorial Manager® and ProduXion Manager® from Aries Systems Corporation

**Availability of data and materials**<br><br>All datasets and code on which the conclusions of the paper rely must be either included in your submission or deposited in publicly available repositories (where available and ethically appropriate), referencing such data using a unique identifier in the references and in the "Availability of Data and Materials" section of your manuscript.<br><br>Have you have met the above requirement as detailed in our Minimum Standards Reporting Checklist?	Yes

Powered by Editorial Manager® and ProduXion Manager® from Aries Systems Corporation

Manuscript

Click here to download Manuscript GigaScience_Manuscript_2020.docx

Genomic consequences of dietary diversification and parallel evolution due to nectarivory in Leaf-nosed bats.

Yocelyn T. Gutiérrez-Guerrero[1], Enrique Ibarra-Laclette[2], Carlos Martínez del Río[3], Josué Barrera-Redondo[1], Eria A. Rebollar[4], Jorge Ortega[5], Livia León-Paniagua[6], Araxi Urrutia[7], Erika Aguirre-Planter[1] and Luis E. Eguiarte*[1]

[1]Departamento de Ecología Evolutiva, Instituto de Ecología, Universidad Nacional Autónoma de México (UNAM), 04510, Mexico City, Mexico.

[2]Red de Estudios Moleculares Avanzados, Instituto de Ecología AC, 91070, Xalapa, Veracruz, Mexico.

[3]Department of Zoology and Physiology, University of Wyoming, 82071, Wyoming, USA.

[4]Centro de Ciencias Genómicas, Universidad Nacional Autónoma de México, Morelos, Mexico.

[5]Departamento de Zoología, Laboratorio de Bioconservación y Manejo, Posgrado en Ciencias Quimicobiológicas, Instituto Politécnico Nacional-ENCB, 11340, Mexico City, Mexico.

[6]Facultad de Ciencias, Universidad Nacional Autónoma de México, 04510, Mexico City, Mexico

[7]Departamento de Ecología Funcional, Instituto de Ecología, Universidad Nacional Autónoma de México (UNAM), 04510, Mexico City, Mexico.

e-mail addresses: ygutierrez@ecologia.unam.mx; enrique.ibarra@inecol.mx; CmDelRio@uwyo.edu; josue_barrera@comunidad.unam.mx; rebollar@ccg.unam.mx; jortegare@ipn.mx; llp@ciencias.unam.mx; a.urrutia@bath.ac.uk; eaguirre@ecologia.unam.mx; fruns@unam.mx

Corresponding author*: fruns@unam.mx

Abstract

Background

The New World Leaf-Nosed bats (Phyllostomids) exhibit a diverse spectrum of feeding habits and innovations in their nutrient acquisition and foraging mechanisms. However, the genomic signatures associated with their distinct diets are unknown.

Results

We conducted a genomic comparative analysis to study the evolutionary dynamics related to dietary diversification and specialization. We sequenced, assembled and annotated the genomes of five Phyllostomid species: one insect-feeder (*Macrotus waterhousii*), one fruit-feeder (*Artibeus jamaicensis*), and three nectar-feeders from the Glossophaginae subfamily (*Leptonycteris yerbabuenae*, *Leptonycteris nivalis* and *Musonycteris harrisoni*), also including the previously sequenced *Desmodus rotundus*. Our phylogenomic analysis based on 22,388 gene families displayed differences in expansion and contraction events across the Phyllostomid lineages. Independently of diet, genes relevant for feeding strategies and food intake experienced multiple expansions and signatures of positive selection. We also found adaptation signatures associated with specialized diets: the vampire exhibited traits associated with a blood diet (i.e., coagulation mechanisms), whereas the nectarivore clade shares a group of positively selected genes involved in sugar, lipid, and iron metabolism. Interestingly, in fruit-nectar feeding Phyllostomid and Pteropodids bats, we detected positive selection in two genes: *AACS* and *ALKBH7,* which are crucial in sugar and fat metabolism. Moreover, in these two proteins we found parallel amino-acid substitutions in conserved positions exclusive to the tribe Glossophagini and to Pteropodids.

Conclusions

Our findings illuminate the genomic and molecular shifts associated with the evolution of nectarivory and shed light on how nectar-feeding bats can avoid the adverse effects of diets with high glucose content.

Keywords

Adaptation, Comparative genomics, Diet, Parallel evolution, Phyllostomid, Specialization

Background

Evolutionary shifts related to changes in feeding habits are considered one of the most important events in animal evolution (1). Diet changes open new ecological and physiological opportunities (1, 2). These shifts often involve changes in feeding behavior, dramatic innovations in the mechanism by which nutrients are assimilated and metabolized, and sometimes drastic morphological modifications (3). Evolutionary diet shifts are sometimes accompanied by species diversification and adaptive functional trait radiation (4).

The New World leaf-nosed bats (family Phyllostomidae) are one of the most species-rich mammalian taxa, with 216 species in 60 genera (5, 6). Leaf-nosed bats evolved from an insect-feeding common ancestor and now display a large and diverse spectrum of feeding habits that include insectivory, carnivory, frugivory, blood-feeding, nectar-pollen feeding and omnivory (5-7). Moreover, dietary specializations and species diversification seem to be correlated in these bats (7,6).

Although most extant Phyllostomids are insectivorous or omnivorous (6, 7), two lineages have extreme dietary specialization: blood-feeding within the subfamily Desmodotinae (including *Desmodus*, *Diphylla* and *Diademus*) and the nectar-pollen feeding species within the subfamily Glossophaginae (including *Leptonycteris*, *Glossophaga*, *Choeronycteris* and *Musonycteris*), that feed primarily on nectar and pollen (5, 6). Among these nectar-pollen feeding species, *Leptonycteris yerbabuenae* (Lesser long-nosed bat) is notable due to its tight co-evolutionary interactions with plants and seeming specialization to nectarivory/pollinivory (8-10). The blood-feeder *Desmodus rotundus,* and the two other species in the Desmodotinae subfamily have a feeding mode unique among mammals (11). Data on the genome and microbiome of *D. rotundus* have revealed remarkable adaptive changes genes associated with blood diet (12).

Many studies have demonstrated evidence of evolutionary novelties associated with feeding

diversification in leaf-nosed bats (6, 13, 14, 15). These include morphological traits involved in nectar extraction (10), and physiological characteristics related to the processing of a diet high in sugars (13, 14). However, the genomic signatures associated with dietary diversification and specialization during the evolution of Phyllostomid bats from an insect-feeder common ancestor remains largely unknown.

We investigated the genomic and evolutionary dynamics associated with the dietary diversification and nectar-pollen feeding specialization of Phyllostomid bats. We sequenced and assembled the whole genomes of five Phyllostomid bat species, including ecologically and economically important species. We sequenced the genomes of three nectar-pollen feeders *Leptonycteris yerbabuenae*, *Leptonycteris nivalis* and *Musonycteris harrisonii*; the fruit-feeder *Artibeus jamaicensis*; and the insect-feeder *Macrotus waterhousii*. For comparative purposes our analyses incorporated genomic data of the vampire *Desmodus rotundus* (12) and other mammals.

Our research was guided by three sets of predictions. First, we predicted that the dietary diversification from insectivory, which is the ancestral condition in the group, to derived diets would be accompanied by evolutionary changes in relevant genes involved in food uptake and the metabolic pathways associated with the processing of assimilated nutrients. Second, we predicted that the dietary specializations observed in the subfamilies Desmodotidae and Glossophaginae would be correlated with evidence of selection in genes that facilitate the assimilation and metabolism of components of blood and nectar, respectively. More specifically, we expected the nectar-pollen feeder linage to show adaptive signals in genes involved in sugar assimilation and metabolism. Our third prediction was that we should detect convergent evolution between the New World fruit and nectar feeders and the Old World fruit-bats in genes important for carbohydrate metabolism.

We adopted a hierarchical approach: we examined our predictions in deep nodes of the phylogeny, then we identified the nodes that represent dietary transitions and investigated the changes that accompanied these transitions. We conducted a genomic comparative approach

and performed a phylogenomic reconstruction to identify expansions/contractions of gene families across the Phyllostomids lineage. We also evaluated orthologous protein sequences that have been targets of selection and their relation to dietary diversification and specialization. Finally, in order to identify convergent evolutionary signals associated with the diet, we carried out a comparison between the genomes of the nectar-pollen feeders Phyllostomid bats and the Old World fruit-feeder bats (family Pteropodidae), analyzing radical amino-acid substitutions in conserved positions.

Data Description

We sequenced the genome of one adult male Lesser long-nosed bat (*Leptonycteris yerbabuenae*) by a whole high-throughput shotgun strategy and obtained a high quality *de novo* assembly (104 x) (Table 1; see Additional file 1, Table S1, S11). Additionally, we sequenced with medium coverage (~24 – 56 x) the genomes of four Phyllostomid bats: *M. waterhousii* (insect-feeder), *A. jamaicensis* (fruit-feeder), and the nectar-pollen feeders: *M. harrisoni* and *L. nivalis*. (see Additional file 1, Table S1).

The genomic landscape of New World Leaf-nosed bats

The size of *L. yerbabuenae'*s genome was similar to those reported for other bats (2.05 Gb), with an N50 scaffold length of 14, 735,151 bp, and L50 of 38 scaffolds (Table 1). Evaluation of the genome assembly for completeness based on BUSCO identified 94% of complete and 2.5% of fragmented genes from the mammalian database (Mammalia *odb9*). The genome contained 24,074 inferred coding sequences from an *ab initio* prediction, the transcript evidence and homology evidence obtained from a set of proteins of several mammalian species (Table 1; see Additional file 1, Table S2). Approximately 26% of the genome assembly was composed of repetitive elements (547 Mbp length) (see Additional file 1, Table S3).

We constructed a reference guide genome assembly based on *L. yerbabuenae* for the other four Phyllostomid bats, where we annotated from 18,000 to 24,471 coding sequences and

proteins for each Phylllostomid (Table 2; see Additional file 1, Table S4-S5; Additional file 2, Fig. S1).

Analyses

Gene family evolution reflects distinct dietary needs

To understand genomic evolution and to trace changes associated with dietary diversification and specialization, we reconstructed a phylogenomic tree using 132 single-copy orthologous genes (61,331 amino acids sites), which was calibrated using two fossil dates (16-18). Based on the phylogenomic tree, we analyzed the dynamics (expansion and contractions) for 22,388 gene families across the Phyllostomid bat genomes (Fig. 1 and Table 3).

For all the Phyllostomid bats, the significant gene family enrichment functions were related to the cellular repair process and genetic make-up for protein synthesis. Furthermore, across the Phyllostomid bats many gene families exhibited changes with feeding habits, for example, the Phyllostomid node had a contraction related to the lipid metabolism. The blood-feeder lineage had a significant gain on gene families involved in the regulation of appetite and process for nitrogen acquisition, but also this lineage showed many contraction events involved in calcium metabolism (Table 3). The fruit and nectar feeding bats exhibited many expansion events in iron metabolism regulation pathways (Table 3).

Rapidly evolving genes across the whole genome

For all Phyllostomid bats, most of the adaptive genes are related to immune response, DNA repair, inflammatory response, RNA catalytic process and genes that mediate muscle function (such as *Myoblast and PAMR1*) (Fig. 2; see Additional file 1, Table S6-TableS7) (19).

Ecological and feeding behavior adaptations across the Phyllostomid lineages

To understand shifts associated with dietary diversification, we analyzed genes under positive selection involved in the mechanisms of carbohydrate digestion and lipid metabolism in each Phyllostomid species (Fig. 2).

In *M. waterhousii*, an insect-feeder, we found evidence of positive selection in: *Chitinase*, which codes for proteins in the degradation of insect exoskeletons (20) (Fig. 2) Interestingly, for *M. waterhousii*, the *Trehalase* is a partial gene that exhibited signals of positive selection, but for the rest of the Phyllostomid species, *Trehalase* is a pseudogene. This finding is relevant, because *Trehalase* is the principal sugar in insects blood.

The vampire's genome revealed a complex set of genes crucial for maintaining a blood feeding diet under positive selection, including *THBD* (hematopoietic cell pathway) and *A2M* (complement and coagulation cascade pathway) (21). Only in the vampire, we found under positive selection genes involved in feeding and lipid – cholesterol metabolism such as *MGAT2*, *PLAS2G16,* and *GFOD1* (22, 23) (Fig, 2). Interestingly, the vampire was the only genome where the *Trehalase* gene was completely missing (Fig. 2).

In the fruit bat *A. jamaicensis* most of the enzymes analyzed involved in lipid and carbohydrates metabolic pathways showed positive selection pressures (Fig. 2; see Additional file 1, Table S8). The importance of these enzymes for this fruit bat might reflect the diversity of its diet, as *A. jamaicensis* has been documented to eat, besides insects and fruits, seeds and leaves (24).

Every nectar-pollen feeder bat species (*M. harrisoni*, *L. nivalis* and *L. yerbabuenae*) showed positive selection signatures for genes involved in insulin secretion (*UCN 3*) (25), calcium and iron storage (*CALP2, CD248* and *FTL*) (26-27), bone morphogenetic regulation (*Fslt1*) (28), and in *IAP*, the gene coding for the mucosa defense factor involved in proper gut homeostasis (Fig. 2) (29). Interestingly, we found adaptive signatures for genes crucial for carbohydrates and lipid metabolic pathways, such as: pancreatic secretion, glycolysis / gluconeogenesis, glycogen, glycerophospholipid, citrate acid metabolism and ketone metabolism (Fig. 3; see Additional file 1, Table S8) (30-32).

Finally, for the nectar-pollen and fruit bat species, we detected strong selective pressures in four enzymes, *AACS* (which appears to participate in the regulation of lipid metabolism) (32), *ALKBH7* (which codes for a protein that appears to be involved in the regulation of body mass and fat content) (33), *FABP1* (regulates fatty acid trafficking and prevents lipotoxicity) (34) and *AMPK* (major regulator of cellular energy homeostasis) (Fig. 2 and 3) (35).

Adaptation and convergent signatures in fruit and nectar-pollen feeding bats

The sugar and lipid metabolism genes detected under positive selection for the fruit and nectar-pollen feeding Phyllostomid bats, were also analyzed in the genomes of three Old World bat species available in databases, including *Pteropus alecto*, *Pteropus vampyrus* and *Rousettus aegyptiacus* (Family Pteropodidae). We found evidence of positive selection in: *AACS* in *P. alecto*, *P. vampyrus* and *R. aegyptiacus*. In *R. aegyptiacus*, we also found evidence of positive selection in *ALKBH7* (*see* Additional file 1: Table S8).

We analyzed 1,918 orthologous sequences and reconstructed the ancestral sequences states, in order to identify some genes with unique and exclusively parallel substitutions for the Phyllostomid fruit bat, the Glossophagini and Pteropodid lineages, in a conserved position for the rest of the bats and mammal species (Fig. 4b). We found three genes with parallel signatures in a specific amino-acid position. Most of these parallel changes presumably led to changes in the physicochemical properties of the expressed protein (Fig. 4a). In *AACS,* we identified six radical amino-acid substitutions along the sequence, from an ancestral glycine (non-polar) to a derived arginine (+ charger), alanine (non-polar) to threonine (polar), glycine to serine (polar), alanine to proline (non-polar), serine to proline and serine to leucine (non-polar) (Fig. 4a). In *ALKBH7,* we found a parallel amino-acid substitution from glutamic acid (- charger) to lysine (+ charger) and arginine to glutamine (polar). The latter change was found in the Glossophagines and *R. aegyptiacus* (Fig. 4b). Finally, for the gene *UNC-45 B,* which codes for a protein involved in muscle cell development, we identified a substitution from leucine to arginine (Fig. 4a) (36). Moreover, we calculated the posterior probabilities for each amino acid reconstruction state for

each node along the three. For all nodes in the Glossophagini and Pteropodids clades, the probabilities of each amino acid derived state were > 80% (Fig. 4c; see Additional file 1, Table S9).

Finally, in order to evaluate if the radical amino acid substitutions had affected the protein structure of *ACCS,* we modeled its 3D- protein structure using Hidden Markov Models*,* for the nectar-feeders: *M.* harrisoni, *L. nivalis* and *L. yerbabuenae*; the fruit bat *P. alecto*; and *D. rotundus* and *M. waterhousii* (Fig. 5a; see Additional file 1, Table S10). *ACCS* protein structure is composed by 662 amino acids, two domains and 96 atoms of beta strand, 181 atoms of alpha helix and 4,391 loop atoms (Fig. 5a). Moreover, we performed a multi-comparison of the 3D structure for all the species mentioned above (see Additional file 1: Table S11). We found high similarity in the 3D protein structure for all bats (RSMD values from 0 to 0.003). However, we identified three residues of alpha-helix shared only for the Glossophaginae clade, and a beta strand shared only between *M. harrisoni* and *P. alecto* (Fig. 5b; see Additional file 1, Table S11).

Discussion

Our study provides unprecedented knowledge on the genomic signatures behind the dietary diversification and specialization in Phyllostomid bats. Surprisingly, and contrary to our first prediction, we found that many of the genomic characteristics of the ancestral Phyllostomid diet, remained functional in all lineages of the family. For example, the C*hitinase* gene was functionally conserved in most genomes (with the exception of the vampire), highlighting the relevance of digestion and nutrient uptake from insects in all lineages, including those that mainly feed on fruit, nectar-pollen and even in some cases, those than feed only on blood (Fig. 2 and 3) (9, 10, 20). However, as previously studies suggest, we found that *Trehalase* (involved in the digestion of trehalase from insect blood, comprising approximately to 7% of the dry mass) is missing or a pseudogene for all the blood, fruit, and nectar-feeding bats, which may be a result of dietary diversification in the family (37).

Our second prediction was supported: we found unique genomic specializations in bats with obligate and restrictive diets, such as the vampire and the nectar-pollen feeders (Fig. 2 and 3, Table 3). The vampire's genome has unique characteristics associated with the ability to consume blood, including genes that play a crucial role in the down-regulation of fibrinolysis and those that control the production of blood cells (Fig. 3) (12, 21, 38). On the other hand, we also detected strong positive selection in genes crucial for carbohydrate oxidation, ATP production, and in genes involved in ketone metabolism in the three Glossopaghini bats. These are associated with the extreme energetic feat of feeding on the wing (14, 30, 39) (Fig. 3). Our analyses also highlight the importance of genes involved in iron storage for animals that feed on iron deficient sources. These results might be related to the avoidance of metabolic disorders such as anemia (Fig. 3) (28). The results also help to explain how bats that feed on nectar-pollen can avoid the potentially adverse effects of their peculiar diet. In humans, loss-of-function due to mutations in some of these genes are associated with nutrient malabsorption and metabolism disorders including diabetes, hyperglycemia and obesity (30, 39).

In support of our third prediction, we identified signatures of molecular parallel evolution shared by fruit-feeding Pteropodids and nectar-pollen feeding Glossophanigi bats (Fig. 4ab). The ancestral sequence reconstruction provided us with insights into the mechanisms of molecular adaptation and functional divergence. Signals of parallel evolution and adaptative selection for the proteins: *ACCS* and *ALKBH7,* shed light on the importance of the storage of fatty fuels necessary to meet the energy demands of an expensive mode of foraging and pollinator ecology of these specialist bats. Protein function is more likely to be affected if genes show many radical substitutions in conserved positions, and signal of positive selection. In spite of the evolutionary changes detected for *ACCS* in the Glossophagini species, their tertiary protein structure exhibited high similarity when we compared it with other bats (see Additional file 1, Table S11). However, we identified exclusive differences in alpha-helix and beta-strand regions (Fig. 5b), that it may be important in the protein function for the nectar-pollen feeders and the Old World fruit bat (Fig. 5). We consider that it is very probable that *ACCS* is up or down regulated. Future studies must

evaluate the expression levels for this gene, and its regulation, including replicating and analyzing more tissues, such as the gut and liver (40, 41).

Surprisingly, the protein *UNC-45 B,* exhibited the same amino acid substitution between Glossophagini, Pteropodids and the Dolphin. Lee *et al.,* (2018) have identified genes involved in muscle skeletal function and movement, with parallel substitutions shared between bats and marine mammals (such as dolphin, whale and Baiji). We suggest that the protein *UNC-45 B* may be implicated in an efficient mobility and superfast muscle physiology for this species (42).

On the other hand, the molecular traits that we infer as the result of parallel evolution, were not found in the fruit-eating bat *A. jamaicensis*. We hypothesize that this species should be considered more omnivorous than strictly frugivorous (24, 41). Omnivory-frugivory might have been an important step in the transition to a more restricted fruit diet and to a nectar-pollen diet (Fig. 3 and 4) (5-8). To explore this hypothesis further, it will be necessary to expand our sample of genomes to include more Phyllostomid species that have more exclusively frugivorous fruits than *A. jamaicensis*.

Our findings suggest that convergent evolution is likely a consequence of dietary specialization and high metabolic demands required for foraging on flowers and fruits. These results are notable, given that the Pteropodidae and Glossophaginae lineages are separated by over 60 Mya (8) (see Additional file 2, Fig. S2) (17). Moreover, our results shed light on the evolutionary mechanisms and genomic shifts that take place in the transition to novel feeding habits. They also illuminate on the genomic changes that take place when animals adopt a diet dominated by sugar consumption, and with low levels of lipids and proteins. The generality of inferences can be tested in other nectar specialized vertebrate taxa, such as hummingbirds (40).

Finally, we found differences in the evolution of gene families and genes that are not necessarily or only related to diet among Phyllostomid lineages (Fig. 1 and Table 3). These differences are likely associated to other lineage-specific aspects in physiology, ecology (niche resources, interactions, immune system, etc.) and microbiomes (5, 6, 12, 43). As an example, positive selection in *IAP* enzyme was only detected in the nectar feeding bats, which supports a

strong relationship between dietary specialization and the bacterial communities, that are involved in providing vitamins and aiding digestive processes (Fig. 3) (12). Future analyses should address the relationship between host diet-intestinal bacterial community, and the evolution of microbiomes across dietary diversification and specialization.

Methods

Animal sampling and genome sequencing

An adult male *Leptonycteris yerbabuenae* was collected and processed on site at the cave "El Salitre" in Morelos state, Mexico (18°44'28" N, 99°10'46" W). All procedures were carried out in accordance with Federal Mexican Procedures (Guidelines of Secretaría de Medio Ambiente y Recursos Naturales, SEMARNAT), permit SGPA/DGVS/07161/15. The Zoology Museum "Alfonso L. Herrera" (Facultad de Ciencias, UNAM), donated the tissue samples from four leaf-nosed bats: *Macrotus waterhousii, Artibeus jamaicensis*, *Leptonycteris nivalis* and *Musonycteris harrisoni* (see Additional file 1, Table S1).

For all leaf-nosed bats species, we isolated their DNA using Phenol-Chloroform protocol and DNA Blood and Tissue Kit (Qiagen). We used the Illumina HiSeq 4000 150 PE platform to sequence the genomes (see Additional file 1, Table S1). We paid special attention to *L. yerbabuenae,* in order to use it as a reference to help in the assembly construction of the other genomes. In this species, we performed high whole genome sequencing (the DNA sample was sequenced on two lanes). Additionally, we used the fresh samples collected from *L. yerbabuenae* to obtain transcriptional evidence for the genome annotation, we extracted the RNA-Seq from five tissues: brain, pancreas, kidney, lung, and liver (reserved in a buffer storage of RNA stabilization) using the RNeasy Mini Kit (Qiagen). All tissues with RIN values >= 8 were sequenced on Illumina HiSeq 4000 150 PE platform.

***Leptonycteris yerbabuenae* genome assembly**

De novo genome assembly

The genome assembly was constructed *de novo* with Platanus v. 2.4.3 (44), using a heterozygous value = 0.04 (-u 0.04) and a initial kmer=32. To accurate, optimize and extend the genome assembly, we performed a scaffolding with MeDuSa software (45, 46). Finally, we used Pilon for correcting bases and polish the genome assembly (47).

We evaluated the genome assembly metrics (total length, number of scaffolds, number of contings, L50, N50, and others). Moreover, with BUSCO v3 and the Mammalia odb9 database (48) we evaluated the measure for quantitative assessment of the genes content into de genome assembly.

Gene prediction

We performed TEdenovo from the REPET package (49) to predict, identify and annotate the transposable elements (TE) using the repetitive elements database Repbase (50). We masked the TEs across the genome using RepeatMasker v4.0.7 (see Additional file 1, Table S3) (51).

We also cleaned, filtered and assembled the RNA-Seq data of five tissues (brain, pancreas, kidney, liver, and lung) for the same individual with Trinity v4.4.7 (52). Based on the transcriptome assembly, we identified open-reading frames, coding sequences and their corresponding proteins (53).

We generated an *ab initio* gene prediction using Augustus v2.5.5 (54). To train Augustus, we used the gene structures of *E. fuscus* bat, and the RNA-Seq evidence (transcripts annotated from *L. yerbabuenae*). We performed a functional annotation by blastp using the UniProtKb SwissProt dababase and InterProScan (55, 56).

NW Leaf-Nosed bats reference genome construction (assembly and annotation)

We used the *L. yerbabuenae* assembly as a reference genome to construct the assembly of *M. waterhousii*, *A. jamaicensis, M. harrisoni* and *L. nivalis*.

All the raw data were filtered and cleaned (using a PHRED score >= 30) (see Additional file 1, Table S1). We followed the GATK v2.07 pipeline to identify Single Nucleotide Variants (SNVs) (57). First, to find all the SNVs along the genomic information from each Phyllostomid, based on

the *L. yerbabuenae* genome assembly, all the high-quality genomic reads of each Phyllosomid were mapped to *L. yerbabuenae* genome assembly with BWA mem (58). Second, we used the GATK and Picard tools to recalibrate the genome mapping and identified the SNVs for each Phyllostomid (SortSam, MarkDuplicates, AddOrReplaceReadGroups, BuildBamIndex and CreateSequenceDictionary, RealignerTargetCreator, IndelRealigner, HaplotypeCaller, VariantFiltration, SelectVariants, BaseRecalibrator, AnalyzeCovariates, PrintReads, and VariantFiltration) (see Additional file 2, Fig. S3; Additional file 3, Methods) (56, 59). We constructed the consensus sequence based on the SNVs identified (with SAMtools, BCFtools, vcfutils.pl and SeqtK) (60-62). We evaluated for each consensus genome constructed, the assembly metrics and the integrity and gene contend with BUSCO.

We predicted, identified and masked the TEs for each consensus genome using the pipeline of RepeatMasker v4.0.7 (50, 51). We performed the gene prediction with Augustus. The genomes were annotated with blastp and InterProScan (see Additional file 3, Methods).

Phylogenomic and gene family analysis

A total of 132 single-copy orthologous genes (61,331 amino acids sites), across 18 mammals were used to reconstruct a phylogenomic tree (best-fit model distribution JTT, +G +I +I+G and 80% consensus threshold) (see Additional file 3, Methods). We estimated molecular substitution rates with CODEML from the Phylogenetic Analysis by Maximum Likelihood, PAML package (63). Based on a Bayesian phylogenetic method, with MCMCtree tool, we estimated the species divergence times using fossil records from *Icaronycteris* (~50 Mya) and *Tachypteron* taxon with molecular ages of 64 Mya (16-18).

We used CAFE (64) to analyse the statistical changes in the gene family sizes using a birth and death estimator (λ and μ). Based on the distribution of observed family sizes, we calculated the *p-values* for gene family expansion and contractions. We carried out the gene family annotation with shell and PERL scripts (see Additional file 3, Methods).

dN/dS analysis using a branch-site model

Orthologous single copy genes and filtering

We used the proteins annotated information of *M. waterhousii*, *A. jamaicensis*, *M. harrisoni*, *L. nivalis* and *L. yerbabuenae* to create a database, incorporating the complete set of proteins of all Laurasiatheria species available in ENSEMBL database (35 species), and the protein information of all bat species available in NCBI database (7 species) (see Additional file 1, Table S12). Based on a multi-species genome comparison with this database, we inferred the orthologous single copy genes using Diamond and the program Proteinortho (65, 66). We extracted all the single copy genes (scg) shared for each Phyllostomid bat, obtaining more than 9,637 scg clusters. We checked and removed all potential paralogous, sequences with ambiguous amino acids (letter X), and retained sequences where the length is within 80 to 120% relative to the human and mouse sequences. Each single copy gene cluster was composed from 8 to maximum 20 sequences.

Each cluster was aligned with MAFFT aligner tool (67) and poorly aligned regions were removed. We used the alignments and their corresponding coding sequences to perform a robust conversion of protein multi alignment into their corresponding codon alignments with PAL2NAL (see Additional file 3, Methods) (68). We reconstructed the phylogenetic tree for each cluster with RaxML tool (parameters -m GTRGAMMA -p 12345) (69).

dN/dS Test

We used the codon multi-alignment files and their corresponding phylogenetic tree to calculate synonymous sites and nonsynonymous sites (dN/dS) rates, and the average ratio of substitution per site (ω=dN/dS), using two bioinformatic tools: CODEML and HYPHY (63, 70).

With CODEML, we used a branch-site model, specifying the *foreground* branch (the species of our interest) and incorporating a null model [that assumes that background and the foreground branches share the same ratio (ω)]. We designated each Phyllostomid species as the foreground branch of our interest, and we performed independently the CODEML analysis. For assigning significance, we constructed the Likelihood Ratio Test (LRT) for each Phyllostomid result, using the likelihood values from the null and test model, and calculated the *p-value* =< 0.05 under a chi-

square distribution. We also performed a *p-value* adjust, using the False Discovery Rate (FDR) correction, based on the likelihood ratio. Additionally, we considered under positive selection all those sequence sites with a posterior probability > 95% (by Bayes Empirical Bayes method).

With Hyphy, we used aBSREL (adaptive Branch-Site Random Effects Likelihood) (71). aBSREL, infers the optimal number of ω to test if positive selection has occurred on a proportion of branches. The LRT is performed at each branch and compares the test model and null model. We inferred the optimal ω for all the branches for each single copy gene cluster (including bats and non-bats species).

We retained and classified those adaptative genes that were identified in both programs (CODEML and HYPHY), with a *p-value* =< 0.05.

GO enrichment

We performed an enrichment analysis, using the weight01 algorithm with topGO v2.26 package from Bioconductor project in R (72-73). We obtained the statistical significance for the GO enrichment terms by performing the Fisher's exact test (*p-value* =< 0.01).

Radical amino-acid substitution in conserved positions

Ancestral sequence reconstruction

Based on the previously inferred orthologous genes, we extracted all single copy genes shared by the Glossophagini (Glsp): *M. harrisoni*, *L. nivalis*, *L. yerbabuenae;* and the Pteropodids (Ptrp): *P. alecto*, *P. vampyrus* and *R. aegyptiacus.* Each single copy gene cluster was composed from 12 to maximum 30 sequences.

We obtained 1,918 clusters of orthologous sequences (including at least one Glsp and one Ptrp). Each cluster was aligned using PRANK (74) and we constructed their corresponding phylogenetic tree with RAxML (parameter -m PROTCATLG) (69). We checked all alignments for gaps and premature stop codons. We performed an ancestral sequence reconstruction using the protein alignments and phylogenetic trees. To dismiss incorrectly inferred residues and only retain the accurate for the reconstructed ancestral sequence, we used two different program:

CODEML, that assumes a Markov process model and calculates a Bayesian empirical likelihood for each character at each sequence position (63), and FastML, that assumes a continuous time Markov process model and provides the posterior probabilities for each character at each sequence position (75). Both programs provide the ancestral sequence and the posterior probabilities distribution. For CODEML, we fixed the parameters: model =2, fix_alpha = 0, alpha = 0.5 and RateAncestor = 1.

Based on the ancestral sequence information, we identified pairs of branches for Glsp and Ptrp species that exhibited a parallel amino acid substitution. We also checked the ancestral state at each node for these substitutions. We classified each parallel substitution as a radical amino acid substitution in a conserved position, assuming two criteria: 1) parallel substitution is exclusive in the branches of Glsp, Ptrp and their corresponding nodes; and 2) different physicochemical properties between the most frequent amino acid state and the derived (parallel substitution).

Finally, we checked the Bayesian empirical likelihood at each ancestral state for all parallel substitution position. We retained only those parallel substitution with a posterior probability > 85%.

Drivers of parallel evolution

In independently branches, mutation and selection can have equal impacts on patterns of parallel substitutions. For those proteins that exhibited parallel evolution, we also evaluated four variables at DNA and protein level: length, GC percent, rates at synonymous sites and nonsynonymous sites (dN/dS) and isoelectric point (76-77).

Phylogenetic tree

We constructed a phylogeny using the information of *ACCS, ALKBH7* and *UNC-45 B.* We concatenated the amino acid sequence for these three genes. We aligned the sequences with MAFFT tool. We used ProtTest3 to select the best-fit model of protein evolution (79). The phylogenetic tree was constructed using a Maximum Likelihood method with RAxML.

Protein modeling

We modeled the second and tertiary structure of the protein Acetoacetyl CoA Synthetase (ACCS) for *M. waterhousii*, *D. rotundus*, *M. harrisoni*, *L. nivalis*, *L. yerbabuenae* and *P. alecto*. We used Phyred 2 software (78), which compares the profile of the protein of our interest, with a protein database, using Hidden Markov Models and predicting secondary structure for each residue. To identify differences in the protein structure, we compared the secondary and tertiary structure between the nectar- fruit bats and *M. waterhousii* and *D. rotundus*, using the software PyMOL (80-81). To calculate the RSMD score, we aligned the PDB protein model between pairs.

Availability of data and materials

The datasets generated and analysed during the current study are available in the National Center for Biotechnology Information (NCBI): whole genome assembly for *L. yerbabuenae* within BioProject: PRJNA542899 and SRA: SRR9076597. The RNA-Seq data is available within Bioproject: PRJNA543325 and SRA: SRR9087861-SRR908765. Raw genome data of *M. waterhousii, A. jamaicensis*, *M. harrisonii* and *L. nivalis* are available in the SRA: SRR908760, SRR9087866, SRR9089318 and SRR9089325.

Declarations

Ethics approval and consent to participate

The use of animals in this study was performed in accordance with the Federal Mexican Procedures: Guidelines of Secretaría de Medio Ambiente y Recursos Naturales, SEMARNAT, with permit SGPA/DGVS/07161/15.

Consent for publication

Not applicable.

Competing interests

The authors declare that they have no competing interests.

Funding

Y.T.GG is supported by a doctoral scholarship from the Comisión Nacional de Ciencia y Tecnología (CONACyT, Beca Mixta grant no. 291250). The study was supported by a grant from Fronteras de la Ciencia, “Genómica de la Diversidad de Vertebrados 1: *Leptonycteris* y la evolución de la nectarivoría en murciélagos y aves” (CONACyT, project no. 177) to L.E.E.

Author’s Contributions

YT.G.G and L.E.E designed and performed research. YT.G.G and L.E.E wrote the paper with contributions from C.M.R, E.A.R, A.U, J.O and E.A.P. E.I.L. helped with computational resources and bioinformatic analyses. J.B.R. helped with bioinformatic analyses. L.L.P. donated the samples. All authors revised and edited the manuscript.

Acknowledgments

This manuscript constitutes part of the doctoral project of the first author, who thanks the Posgrado en Ciencias Biomédicas (Universidad Nacional Autónoma de México, UNAM) and acknowledges the doctoral scholarship supported by Comisión Nacional de Ciencia y Tecnología (CONACyT, grant no. 580116; Beca Mixta grant no. 291250), and the grant Fronteras de la Ciencia (CONACyT, project no. 177). The authors wish to acknowledge the Instituto de Ecología (UNAM), Comisión Nacional para el Conocimiento de la Biodiversidad (CONABIO) and Instituto de Ecología A.C. (INECOL, A.C) for computing resources. Special thanks to MSc. R. Trejo-Salazar, MSc. O, Gaona, Biol. A, Galicia for their assistance during fieldwork in the El Salitre cave. We thank Dra. L. Espinosa Asuar, Dra. V. Souza, S. Barrientos and all the Lab. Evolución Molecular y Experimental (UNAM) for their help in lab work. To MSc. E. Villafán, Ing. Rodrigo García, and Mat. E. Campos for their computational assistance. We gratefully acknowledge Dr. S. Ramírez-Barahona, C. Keenan and Dr. R. A. Medellin-Legorreta, for their constructive comments on the manuscript.

References

1. Hunter JP. Key innovation and ecology of macroevolution. *Trends Ecol Evol.* 19981;3:31–36.
2. Yoder JB, Clancey E, Des Roches S, Eastman JM, Gentry L, et al. Ecological opportunity and the origin of adaptive radiations. *J Evol Biol.* 2010;23:1581–1596.
3. Palm W, Thompson CB. Nutrient acquisition strategies of mammalian cells. *Nature.* 2017;546:234–242.
4. Borstein SR, Fordyce JA, O'Meara BC, Wainwright PC, McGee MD. Reef fish functional traits evolve fastest at trophic extremes. *Nat Ecol Evol.* 2018;3-191-199.
5. Rojas D, Vale A, Ferrero V, Navarro L. When did plants become important to leaf-nosed bats? Diversification of feeding habits in the family Phyllostomidae. *Mol Ecol.* 2011;20:2217-28.
6. Rossoni DM, Assis APA, Giannini NP, Marroig G. Intense natural selection preceded the invasion of new adaptive zones during the radiation of New World leaf-nosed bats. *Sci Rep.* 2017;7:1–11.
7. Yohe LR, Velazco PM, Rojas D, Gerstner BE, Simmons NB, Dávalos LM. Bayesian hierarchical models suggest oldest known plant-visiting bat was omnivorous. *Biol Lett.* 2015;11:20150501.
8. Fleming TH, Geiselman C, Kress WJ. The evolution of bat pollination: a phylogenetic perspective. *Ann Bot.* 2009;104:1017–1043.
9. Cole FR, Wilson DE. *Leptonycteris yerbabuenae*. *Mamm Species.* 2006;797:1–7.
10. Gonzalez-Terrazas TP, Medellin RA, Knörnschild M, Tschapka M. Morphological specialization influences nectar extraction efficiency of sympatric nectar-feeding bats. *J Exp Biol.* 2012;215:3989–3996.
11. Hong W, Zhao H. Vampire bats exhibit evolutionary reduction of bitter taste receptor genes common to other bats. *Proc R Soc B Biol Sci.* 2014;281:20141079.

12. Zepeda Mendoza ML, Xiong Z, Escalera-Zamudio M, Runge AK, Thézé J, et al. Hologenomic adaptations underlying the evolution of sanguivory in the common vampire bat. *Nat Ecol Evol.* 2018;2:659–668.

13. Ayala-Berdon AJ, Schondube JE. A physiological perspective on nectar-feeding adaptation in Phyllostomid bats. *Physiol Biochem Zool.* 2015;84:661541.

14. Schondube JE, Herrera-M, LG, Martínez del Rio C. Diet and the evolution of digestion and renal function in phyllostomid bats. *Zoology (Jena).* 2001;104:59–73.

15. Zhao H, Zhou Y, Pinto CM, Charles-Dominique P, Galindo-González J, et al. Evolution of the Sweet Taste Receptor Gene Tas1r2 in Bats Research article. *Mol Biol Evol.* 2010;27:2642–2650.

16. Simmons NB, Geisler JH. Phylogenetic relationships of *Icaronycteris*, *Archaeonycteris*, *Hassianycteris*, and *Palaeochiropteryx* to extant bat lineages, with comments on the evolution of echolocation and foraging strategies in Microchiroptera. In: Bull Am Museum Nat Hist. 1998. http://hdl.handle.net/2246/1629. Accessed 01 Sep 2019.

17. Teeling EC, Springer MS, Madsen O, Bates P, O'brien SJ, et al. A molecular phylogeny for bats illuminates biogeography and the fossil record. *Science.* 2005;307:580-4.

18. Simmons NB, Seymour KL, Habersetzer J, Gunnell GF. Primitive Early Eocene bat from Wyoming and the evolution of flight and echolocation. *Nature*. 2008;451:818-821.

19. Chen EH. Invasive Podosomes and Myoblast Fusion. *Curr Top Membr.* 2011;68:235-258.

20. Nardelli A, Vecchi M, Mandrioli M, Manicardi GC. The evolutionary history and functional divergence of trehalase (*treh*) genes in insects. *Front Physiol.* 2019:00062.

21. Okamoto T, Tanigami H, Suzuki K, Shimaoka M. Thrombomodulin: A Bifunctional Modulator of Inflammation and Coagulation in Sepsis. *Crit Care Res Pract.* 2012(Dic):1–10.

22. Tsuchida T, Fukuda S, Aoyama H, Taniuchi N, Ishihara T, et al. MGAT2 deficiency ameliorates high-fat diet-induced obesity and insulin resistance by inhibiting intestinal fat absorption in mice. *Lipids Health Dis.* 2012;11:1–10.

23. Xiong S, Tu H, Kollareddy M, Pant V, Li Q, et al. Pla2g16 phospholipase mediates gain-of-function activities of mutant p53. *Proc Natl Acad Sci USA*. 2014,30:11145-11150.

24. Kunz TH, Díaz CA. Folivory in Fruit-eating Bats, with New Evidence from *Artibeus jamaicensis* (Chiroptera: Phyllostomidae). *Biotropica.* 1995;27:106–120.

25. Li C, Chen P, Vaughan J, Lee K-F, Vale W. Urocortin 3 regulates glucose-stimulated insulin secretion and energy homeostasis. *Proc Natl Acad Sci USA.* 2007;104:4206–4211.

26. Teicher B. CD248: A therapeutic target in cancer and fibrotic diseases. *Oncotarget*. 2019,10:993-1009.

27. Drysdale J, Arosio P, Invernizzi R, Cazzola M, Volz A, et al. Mitochondrial ferritin: A new player in iron metabolism. *Blood Cells Mol Dis.* 2002;29:376–383.

28. Geng Y, Dong Y, Yu M, Zhang L, Yan X, et al. Follistatin-like 1 (Fstl1) is a bone morphogenetic protein (BMP) signaling antagonist in controlling mouse lung development. *Prot Natl Acad Sci USA.* 2011,17:7058-7063.

29. Lallès JP. Intestinal alkaline phosphatase: Multiple biological roles in maintenance of intestinal homeostasis and modulation by diet. *Nutr Rev.* 2010;68:323–332.

30. Sylow L, Kleinert M, Richter EA, Jensen TE. Exercise-stimulated glucose uptake - regulation and implications for glycaemic control. *Nat Rev Endocrinol*. 2016;13:133-148.

31. Griffin BA. Lipid metabolism. *Surgery (Oxford).* 2013;31:267-272.

32. Shi L, Tu PB. Acetyl-CoA and the regulation of metabolism: mechanisms and consequences. *Curr Opin Cell Biol.* 2015;33:125-131.

33. Solberg A, Robertson AB, Aronsen JM, Rognmo O, Sjaastad I, et al. Deletion of mouse Alkbh7 lead to obesity. *J Mol Cell Biol.* 2013;5:194-203.

34. Guzmán C, Benet M, Pisonero-Vaquero S, Moya M, García-Mediavilla M, et al. The human liver fatty acid binding protein (FABP1) gene is activated by FOXA1 and PPAra; and repressed by C/EBPa: Implications in FABP1 down-regulation in nonalcoholic fatty liver diseae. *BBA Mol Cell Biol L.*2013,4:803-818.

35. Srivastava R, Pinkosky SL, Filippov S, Hanselman JC, Cramer CT, et al. AMP-activated protein kinase: an emerging drug target to regulate imbalances in lipid and carbohydrate metabolism to treat cardio-metabolic diseases. *J Lipids Res.* 2012,53:2490-2514.

36. Kachur TM, Pilgrim DB. Myosin assembly, maintenance and degradation in muscle: Role of the chaperone UNC-45 in myosin thick filament dynamics. *Int J Mol Sci.* 2008,9:1863-1875.

37. Jiao H, Zhang L, Xie HW, Simmons NB, Lui H, et al. Trehalase gene as a molecular signature of dietary diversification in mammals. *Mol Biol Evol.* 2019;10:2171-2183.

38. Qu J, Ko CW, Tso P, Bhargava A. Apolipoprotein A-IV: A multifunctional protein involved in protection against atherosclerosis and diabetes. *Cells.* 2019,4:319.

39. Jensen J, Rustad PI, Kolnes AJ, Lai YC. The role of skeletal muscle glycogen breakdown for regulation of insulin sensitivity by exercise. *Front Physiol.* 2011;2:1–11.

40. Workman RE, Myrka M, Wong GM, Tseng E, Welch KC Jr. et al. Single-molecule, full length transcript sequencing provides insight into the extreme metabolism of the ruby-throated hummingbird *Alchilochus colubris*. *GigasScience.* 2018,3:1-12.

41. Moreno-Santillán DD, Machain-Williams C, Hernández-Montes G, Ortega J. De Novo Transcriptome Assembly and Functional Annotation in Five Species of Bats. *Sci Rep.* 2019;9:6222.

42. Lee JH, Lewis KM, Moural TW, Kirilenko B, Borgonovo B, et al. Molecular parallelism in fast-twitch muscle proteins in echolocating mammals. *Science*. 2018;4:eaat9660.

43. Song SJ, Sanders JG, Delsuc F, Metcalf J, Amato K, et al. Comparative analyses of vertebrates gut microbiomes reveal convergence between birds and bats. *M*B*io.* 2020;1:e02901-19.

44. Kaijitani R, Toshimoto K, Noguchi H, Toyoda A, Ogura Y, et al. Efficient de novo assembly of highly heterozygous genomes from whole-genome shotgun short reads. *Genome Res.* 2014;24:1384–1395.

45. Lowe T. tRNAscan-SE: a program for improved detection of transfer RNA genes in genomic sequence. *Nucleic Acids Res.* 1997;25:955–964.

46. Bosi E, Donati B, Galardini M, Brunetti S, Sagot MF, et al. MeDuSa: A multi-draft based scaffolder. *Bioinformatics.* 2015;31:2443–2451.

47. Walker BJ, Abeel T, Shea T, Priest M, Abouelliel A, et al. Pilon: An integrated tool for comprehensive microbial variant detection and genome assembly improvement. *PLoS One*. 2014;0112963.

48. Simão FA, Waterhouse RM, Ioannidis P, Kriventseva EV, Zdobnov EM. BUSCO: Assessing genome assembly and annotation completeness with single-copy orthologs. *Bioinformatics.* 2015;31:3210–3212.

49. Flutre T, Duprat E, Feuillet C, Quesneville H. Considering transposable element diversification in de novo annotation approaches. *PLoS One.* 2011;6:0016526.

50. Bao W, Kojima KK, Kohany O. Repbase Update, a database of repetitive elements in eukaryotic genomes. *Mob DNA.* 2015;6:4–9.

51. Tarailo-Graovac, Chen N. Using RpeatMasker to identify repetitive elements in genomic sequences. *Curr Protoc Bioinformatics*. 2009;4:bi0410s25.

52. Haas BJ, Papanicolaou A, Yassour M, Grabherr M, Blood PD, et al. *De novo* transcript sequence reconstruction from RNA-seq using the Trinity platform for reference generation and analysis. *Nat Protoc.* 2013;8:1494–1512.

53. Evans T, Loose M. AlignWise: a tool for identifying protein-coding sequence and correcting frame-shifts. *BMC Bioinformatics.* 2015;1:376.

54. Stanke M, Morgenstern B. AUGUSTUS: A web server for gene prediction in eukaryotes that allows user-defined constraints. *Nucleic Acids Res.* 2005;33:465–467.

55. The UniProt Consortium. UniProt: The universal protein knowledgebase. *Nucleic Acids Res.* 2018;46:2699.

56. Jones P, Binns D, Chang HY, Fraser M, Li W, et al., InterProScan 5: Genome-scale protein function classification. *Bioinformatics.* 2014;30:1236–1240.

57. Van der Auwera GA, Carneiro MO, Hartl C, Poplin R, Del Angel G, et al. From fastQ data to high-confidence variant calls: The genome analysis toolkit best practices pipeline. *Curr Protoc Bioinformatics.* 2013;43:11.10.1-33.

58. Li H, Durbin R. Fast and accurate short read alignment with Burrows-Wheeler transform. *Bioinformatics.* 2009;14:1754-1760.

59. Broad Institute. Picard Tools. Broad Institute, Github repository. https://github.com/broadinstitute/picard/issues/808.

60. Li H, Handsaker B, Wysoker A, Fennell T, Ruan J, et al. The Sequence Alignment/Map format and SAMtools. *Bioinformatics.* 2009;16:2078–2079.

61. Danecek P, Auton A, Abecasis G, Alberts CA, Banks E, et al. The variant call format and VCFtools. *Bioinformatics*. 2011;15:2156–2158.

62. Quinlan AR, Hall IM. BEDTools: A flexible suite of utilities for comparing genomic features. *Bioinformatics.* 2010;6:841–842.

63. Yang Z. PAML 4 : Phylogenetic Analysis by Maximum Likelihood. *Mol Biol Evol.* 2007;24:1586–1591.

64. De Bie T, Cristianini N, Demuth JP, Hahn MW. CAFE: A computational tool for the study of gene family evolution. *Bioinformatics.* 2006;22:1269–1271.

65. Lechner M, Findeib SS, Steiner L, Marz M, Stadler PF. Proteinortho: detection of (co-)orthologs in large-scale analysis. *BMC Bioinformatics.* 2011;12:124.

66. Buchfink B, Xie C, Huson DH. Fast and sensitive protein alignment using DIAMOND. *Nat Methods*. 2015;1:59-60.

67. Katoh K, Misawa K, Kuma K, Miyata T. MAFFT: a novel method for rapid multiple sequence alignment based on fast Fourier transform. *Nucleic Acids Res.* 2002;14:3059-3066.

68. Suyama M, Torrents D, Bork P. PAL2NAL: robust conversion of protein sequence alignments into the corresponding codon alignments. *Nucleic Acids Res.* 2006;34:W609-W612.

69. Stamatakis A. RaxML version 8: a tool for phylogenetic analysis and post-analysis of large phylogenies. *Bioinformatics.* 2014;30:1312-1313.

70. Pond SL, Frost SD, Muse SV. HyPhy: hypothesis testing using phylogenies. *Bioinformatics*. 2005;5:676-9.

71. Smith MD, Wertheim JO, Weaver S, Murrell B, Scheffler K. et al. Less is More: An adaptive branch-site random effects model for efficient detection of episodic diversifying selection. *Mol Biol Evol.* 2015;5:132-1353.

72. Alexa A, Rahnenführer J, Lengauer T. Improved scoring of functional groups from gene expression data by decorrelating GO graph structure. *Bioinformatics*. 2006;22:1600-7.

73. R Development Core Team. R: A language and environment for statistical computing. R Foundation for Statistical Computing, Vienna, Austria. 2008. http://www.R-project.org.

74. Löytynoja A. Phylogeny-aware alignment with PRANK. *Methods Mol Biol.* 2014;1079:155-70.

75. Ashkenazy H, Penn O, Doron-Faigenboim A, Cohen O, Cannarozzi G, et al. FastML: a web server for probabilistic reconstruction of ancestral sequences. *Nucleic Acids Res.* 2012;40:W580-4.

76. Kozlowski LP. IPC – Isolectric Point Calculator. *Biol Direct.* 2016;11:55.

77. Bailey SF, Guo Q, Bataillon T. Identifying drivers of parallel evolution: A regression model approach. *Genome Biol Evol.* 2018; 10:2801-2812.

78. Kelley LA, Mezulis S, Yates CM, Wass MN, Sternberg MJE. The Phyre2 web portal for protein modeling, prediction and analysis. *Nat Protoc.* 2015;10:845-858.

79. Darriba D, Taboada GL, Doallo R, Posada D. ProtTest3: fast selection of best-fit models of protein evolution. *Bioinformatics*. 2011;8:1164-1165.

80. DeLano WL. PyMOL: An open-source molecular graphics tool. *CCP4 Newsletter On Protein Crystallography*. 2002;40:82-92.

81. The PyMOL Molecular Graphics System, Version 2, Schrödinger, LLC. https://pymol.org/2/

Figures

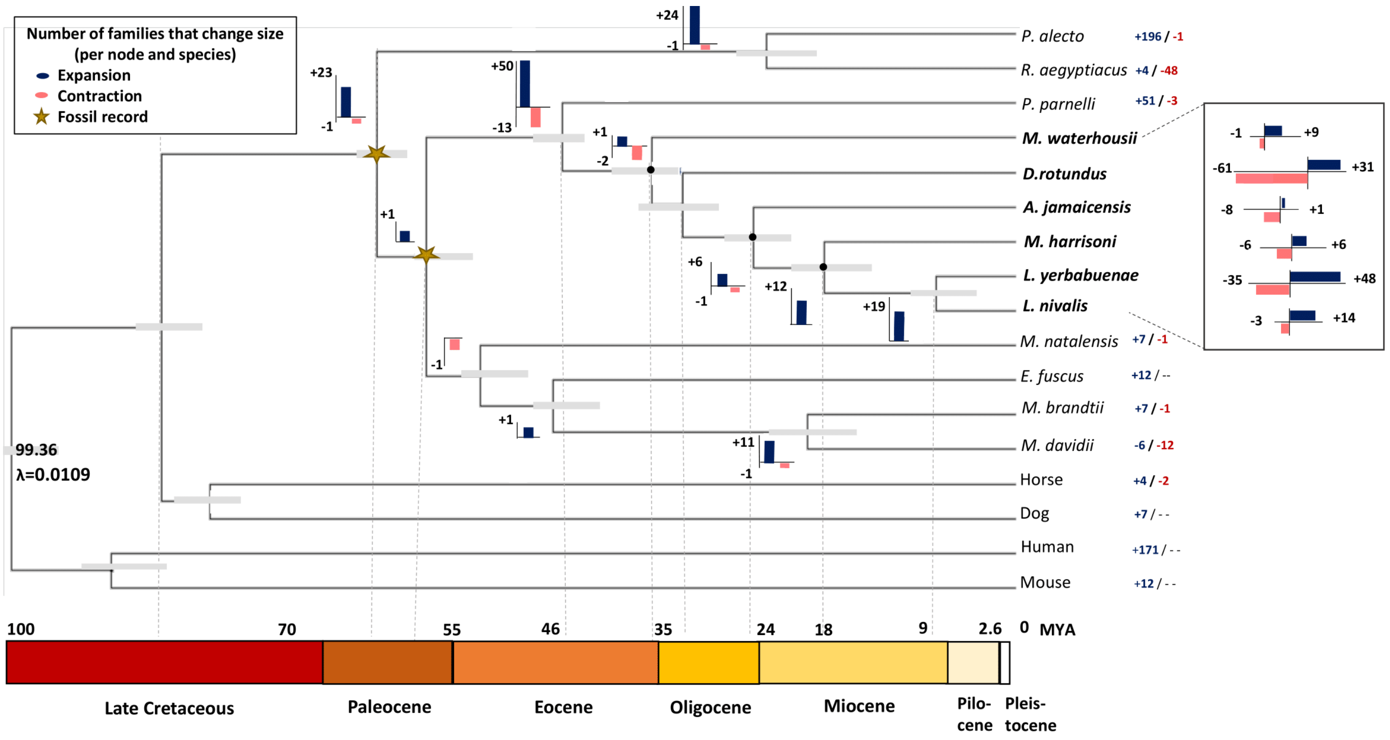

Figure 1. Phylogenetic tree constructed with 132 single copy genes and estimates of divergence times based on two fossil records (yellow stars) (see Methods). Based on 22,388 gene families we analyzed the number of orthologous families expanded (+ blue) and contracted (- red) across the phylogeny: per node (bars) and per species branch (right), with a *p-value* =< 0.01. Gray bars reflect the divergence time interval based on *95% HPD*.

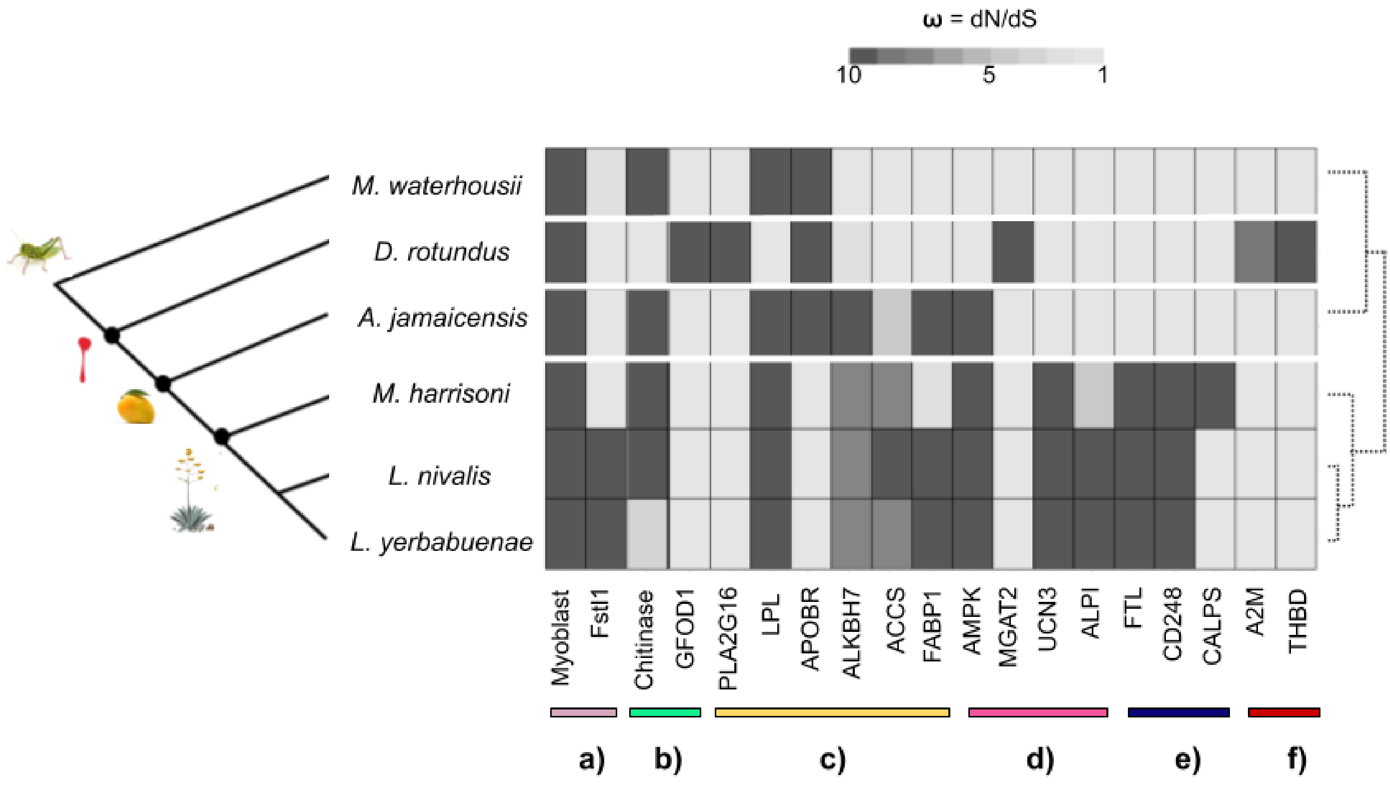

Figure 2. Positive selection in genes and proteins across the phylogeny of Phyllostomid bats, in comparison to the insect-feeder bat *Macrotus waterhousii*. Most of positive selected genes likely contribute to the regulation and processing of: a) muscle and bone development; b) carbohydrates; c) lipids; d) nutrients and food uptake; e) iron storage and calcium sources; and f) blood regulation (see gene and proteins abbreviations in Additional file 1, Table S14*)*.

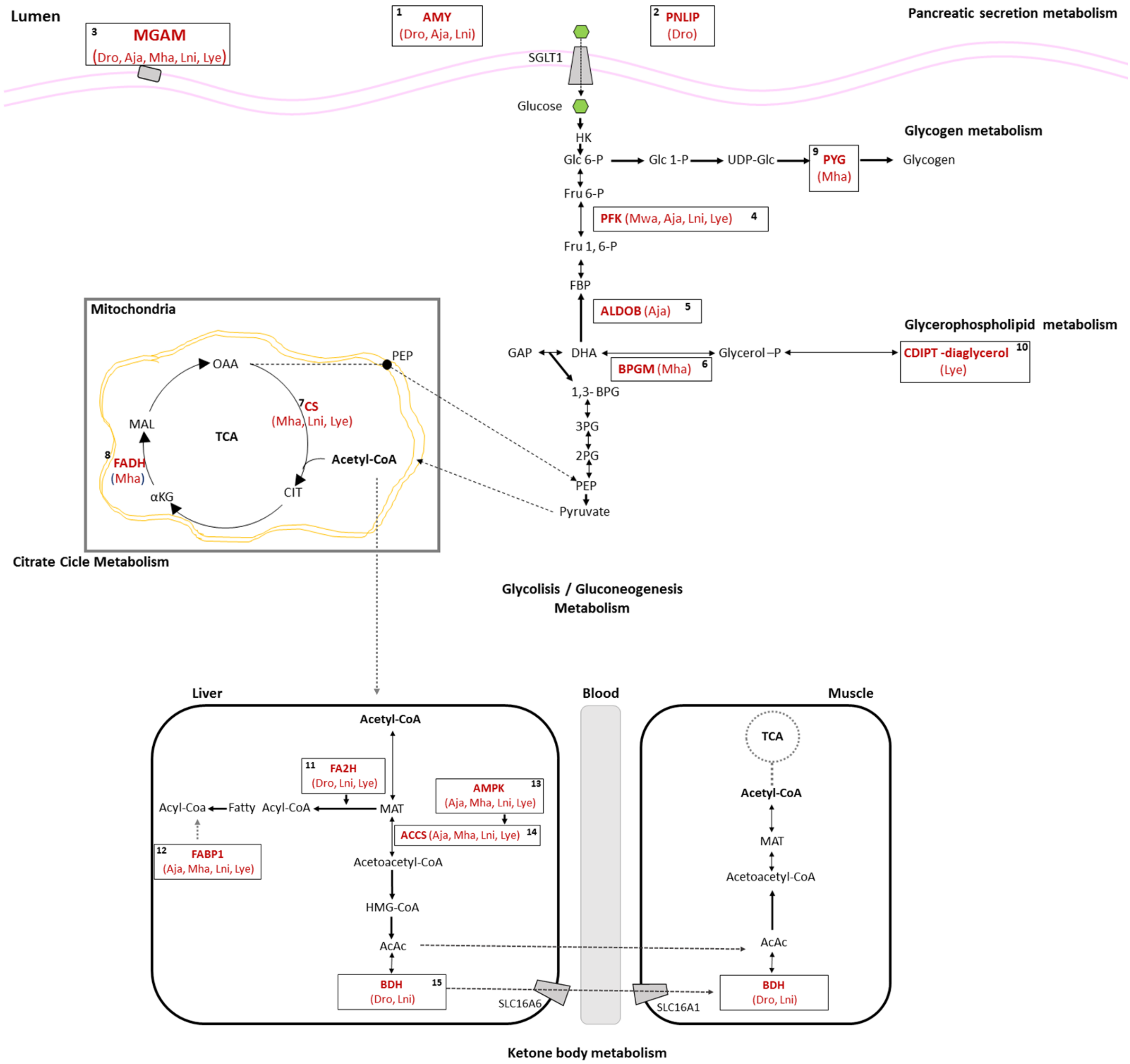

Figure 3. A subset of genes under positive selection (in red bold) that are involved in glucose and ketogen in the frugivorous (Aja: *A. jamaicensis*) and nectar-pollen bats (Mha: *M. harrisoni*, Lni: *L. nivalis*, and Lye: *L. yerbabuenae*). The diagram also identifies adaptative signals for some genes in the vampire *D. rotundus* (Dro) and the insectivore *M. waterhousii* (Mwa). The diagram is based on the KEGG metabolic pathways database and a review of the literature (see gene and proteins abbreviations in Additional file 1, Table S8).

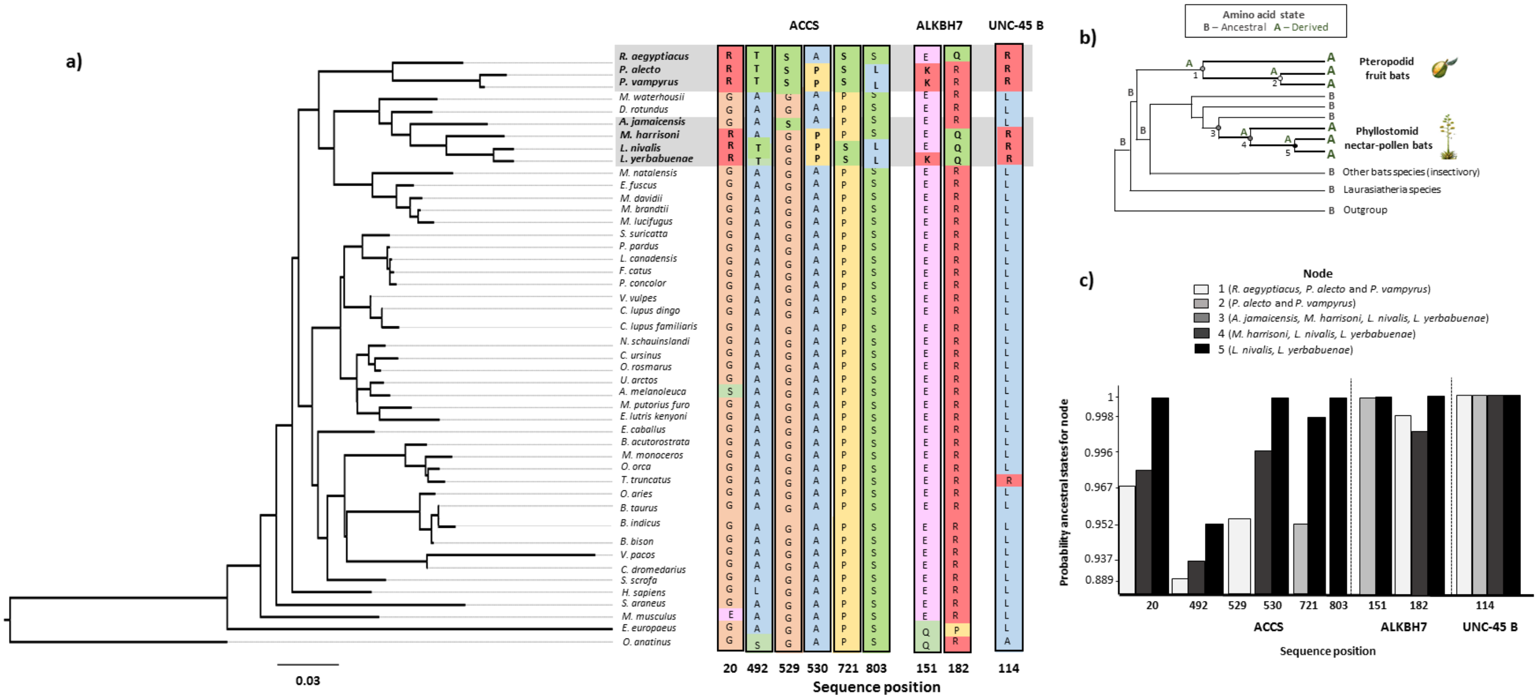

Figure 4. Parallel molecular evolution between Pteropodids (Old World) and Glossophagini (New World) bats, in three genes: Aceotacetyl CoA Synthetase (*AACS*); Alpha-Ketoglutarate-Dependent Dioxygenase Homolog 7, mitochondrial (*ALBKH7*); and UNC-45 homolog B, mitochondrial (*UNC-45 B*). a) Phylogeny reconstruction for these three genes by Maximum likelihood (using 1,827 amino acids), for 47 mammal species. b) Ancestral sequence reconstruction (for branches and nodes) to infer parallel substitutions in conserved positions for the three genes. c) Probability of replacement at each ancestral state node for each sequence position. Amino acid abbreviations: A – Alanine (non-polar); T – Threonine (polar); Q – Glutamine (polar); R – Arginine (basic-charged); K – Lysine (basic-charged); E – Glutamic acid (acidic + charged); S – Serine (polar) and L – Leucine (non-polar).

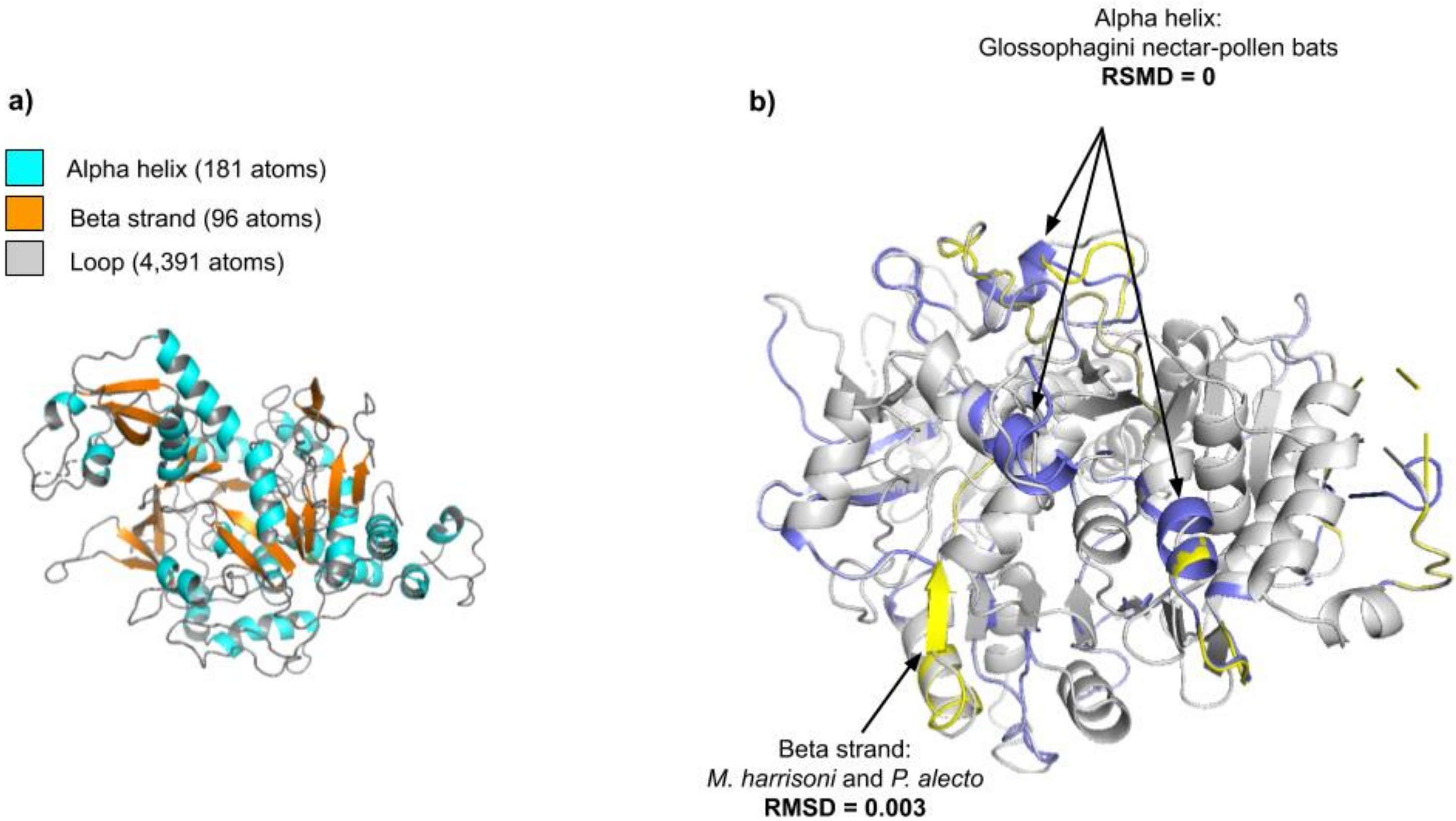

Figure 5. *ACCS* protein structure. a) 3D- structure of *ACCS* protein for *L. yerbabuenae.* b) In gray: *ACCS* 3D- structure consensus (*M. waterhousii* and *D. rotundus*). In blue: alpha-helix structures shared only for the three Glossophagini nectar-pollen feeders (*M. harrisoni*, *L. nivalis* and *L. yerbabuenae*). In yellow: beta-strand shared only between *M. harrisoni* and *P. alecto* (Pteropodid bat). RSMD score (protein 3D superposition and alignment) between pairs of species (see Additional file 1, Table S10).

Tables

Table 1. Global statistics for the nectar-pollen feeding bat *L. yerbabuenae* genome assembly.

i) Sequencing	Total raw data (Gb)	Number Reads > PHRED 30	Coverage (x)
	254.4	690,759,531	103.6
ii) Assembly	N50 (Kb) - L50	Number – Longest (Mb)	Total Size (Gb)
Contig	69.49 - 8,805	78,626 – 0.55	2.05
Scaffold	14,735.1 – 38	34,419 – 70.81	2.05
BUSCO	Completed	Fragmented	Missing
	3,864 (94.1%)	103 (2.5%)	141 (3.4%)
iii) Annotation	Number	Length (Mb)	Percent
Exons	119,036	- -	- -
CDS / Proteins	24,074	- -	- -
Repeats	3,010,348	547.05	26.64%

Table 2. Mapping statistics and SNP identification in Phyllostomid bat genomes (based on *L. yerbabuenae* genome assembly).

	**Phyllostomidae**			
**Species**	*Leptonycteris nivalis*	*Musonycteris harrisoni*	*Artibeus jamaicensis*	*Macrotus waterhousii*
**Diet**	Nectar-pollen	Nectar-pollen	Fruits	Insects
**Total data (Gb)**	131.4	69.2	56.6	128.4
**Coverage (x)**	54.8	30.45	25	56.34
**BUSCO**<br>**Complete**<br>**Fragmented**<br>**Missing**	90.9%<br>4.8%<br>4.3%	90.8%<br>5.0%<br>4.2%	89.3%<br>6.5%<br>4.2%	90.3%<br>4.5%<br>5.2%
**CDS/Proteins**	24,471	20,135	18,756	19,171
**Nucleotide diversity (¶)**	0.014	0.05	0.058	0.056

Table 3. GO enrichment for significant gene families per node and habit food. Gene Ontologies (GO) annotations involved in metabolism and diet are in bold (**+** Gene families expansions; **-** contractions)

Specie and Nodes	Function and Metabolic Pathway	GO	*p-value < 0.01*
Phyllostomid node (expansions)	+ Structural constituent of ribosome	GO:0003735	<1e-30
	+ Translation	GO:0006412	<1e-30
Phyllostomid node (contractions)	- Hydrolase activity	GO:0016788	0.00099
	**- Lipid metabolic process**	**GO:0006629**	**0.001**
	- Aspartic-type endopeptidase activity	GO:0004190	1.8e-06
D. rotundus (expansions)	+ Response to biotic stimulus	GO:0009607	5.7e-12
	+ Defense response	GO:0006952	7.8e-12
	+ Signal transduction	GO:0007165	1.3e-08
	**+ Nitrate assimilation**	**GO:0042128**	**8.8e-08**
	**+ Regulation of appetite**	**GO:0032098**	**0.00034**
	+ Protein glycosylation in Golgi	GO:0033578	0.00313
	+ GTPase activity	GO:0003924	< 1e-30
	+ Molybdenum ion binding	GO:0030151	4.5e-05
(contractions)	- Translation	GO:0006412	1e-30
	**- Calcium ion transmembrane**	**GO:0070588**	**1.3e-16**
	**- Cellular calcium ion homeostasis**	**GO:0006874**	**3.5e-09**
	- Neuron development	GO:0048666	4.2e-09
	- Microtubule-based process	GO:0007017	5.5e-08
	- Homophilic cell adhesion via plasma	GO:0007156	1.7e-07
	- Peptidyl-prolyl cis-trans isomerase	GO:0003755	< 1e-30
	- Ephrin receptor activity	GO:0005003	2.7e-21
	- Ryanodine-sensitive calcium channel	GO:0005219	6.2e-21
	- Inorganic anion exchanger activity	GO:0005452	2.1e-20
	- Ionotropic glutamate receptor activity	GO:0004970	6.3e-20
	- Voltage-gated calcium channel activity	GO:0005245	5.6e-16
A. jamaicensi, *M.harrisoni*, *L. yerbabuenae* and *L. nivalis* (expansions)	+ Translation	GO:0006412	<1e-30
	+ Integral component of membrane	GO:0016021	6.7e-06
	+ Immune response 458	GO:0006955	1.1e-05
	**+ Iron ion import membrane**	**GO:0098711**	**0.00018**
	**+ HFE-transferrin receptor complex**	**GO:1990712**	**5.7e-05**
	**+ Transferrin receptor binding**	**GO:1990459**	**7.3e-05**
(contractions)	- Protein peptidyl-prolyl isomerization	GO:0000413	< 1e-30
M. harrisoni, *L. yerbabuenae* and *L. nivalis* (expansions)	**+ Protein deubiquitination**	**GO:0016579**	**2.8e-09**
	+ Virion assembly 24	GO:0019068	0.00027
	+ Structural constituent of ribosome	GO:0003735	< 1e-30
	+ Thiol-dependent ubiquitinyl hydrolase	GO:0036459	3.6e-11
	**+ Transferrin receptor binding**	**GO:1990459**	**0.0099**
	**+ HFE-transferrin receptor**	**GO:1990712**	**0.0099**
	**+ Iron ion import membrane**	**GO:0098711**	**0.0056**

Additional file

Additional file 1. Supplementary tables. Tables S1-S14 (PDF).

Additional file 2. Supplementary figure. Figures S1-S4 (PDF).

Additional file 3. Supplementary Methods. Methods supporting the manuscript (PDF)

Click here to download Table Table1.docx

Table 1. Global statistics for the nectar-pollen feeding bat *L. yerbabuenae* genome assembly.

i) Sequencing	Total raw data (Gb)	Number Reads > PHRED 30	Coverage (x)
	254.4	690,759,531	103.6
ii) Assembly	N50 (Kb) - L50	Number – Longest (Mb)	Total Size (Gb)
Contig	69.49 - 8,805	78,626 – 0.55	2.05
Scaffold	14,735.1 – 38	34,419 – 70.81	2.05
BUSCO	Completed	Fragmented	Missing
	3,864 (94.1%)	103 (2.5%)	141 (3.4%)
iii) Annotation	Number	Length (Mb)	Percent
Exons	119,036	- -	- -
CDS / Proteins	24,074	- -	- -
Repeats	3,010,348	547.05	26.64%

Click here to download Table Table2.docx

Table 2. Mapping statistics and SNP identification in Phyllostomid bat genomes (based on *L. yerbabuenae* genome assembly).

	**Phyllostomidae**			
**Species**	*Leptonycteris nivalis*	*Musonycteris harrisoni*	*Artibeus jamaicensis*	*Macrotus waterhousii*
**Diet**	Nectar-pollen	Nectar-pollen	Fruits	Insects
**Total data (Gb)**	131.4	69.2	56.6	128.4
**Coverage (x)**	54.8	30.45	25	56.34
**BUSCO**<br>**Complete**<br>**Fragmented**<br>**Missing**	 90.9%<br>4.8%<br>4.3%	 90.8%<br>5.0%<br>4.2%	 89.3%<br>6.5%<br>4.2%	 90.3%<br>4.5%<br>5.2%
**CDS/Proteins**	24,471	20,135	18,756	19,171
**Nucleotide diversity (¶)**	0.014	0.05	0.058	0.056

Click here to download Table Table3.docx

Table 3. GO enrichment for significant gene families per node and habit food. Gene Ontologies (GO) annotations involved in metabolism and diet are in bold (**+** Gene families expansions; **-** contractions)

Specie and Nodes	Function and Metabolic Pathway	GO	*p-value < 0.01*
Phyllostomid node (expansions)	+ Structural constituent of ribosome<br>+ Translation	GO:0003735<br>GO:0006412	<1e-30<br><1e-30
Phyllostomid node (contractions)	- Hydrolase activity<br>**- Lipid metabolic process**<br>- Aspartic-type endopeptidase activity	GO:0016788<br>**GO:0006629**<br>GO:0004190	0.00099<br>**0.001**<br>1.8e-06
D. rotundus (expansions)	+ Response to biotic stimulus<br>+ Defense response<br>+ Signal transduction<br>**+ Nitrate assimilation**<br>**+ Regulation of appetite**<br>+ Protein glycosylation in Golgi<br>+ GTPase activity<br>+ Molybdenum ion binding	GO:0009607<br>GO:0006952<br>GO:0007165<br>**GO:0042128**<br>**GO:0032098**<br>GO:0033578<br>GO:0003924<br>GO:0030151	5.7e-12<br>7.8e-12<br>1.3e-08<br>**8.8e-08**<br>**0.00034**<br>0.00313<br>< 1e-30<br>4.5e-05
(contractions)	- Translation<br>**- Calcium ion transmembrane**<br>**- Cellular calcium ion homeostasis**<br>- Neuron development<br>- Microtubule-based process<br>- Homophilic cell adhesion via plasma<br>- Peptidyl-prolyl cis-trans isomerase<br>- Ephrin receptor activity<br>- Ryanodine-sensitive calcium channel<br>- Inorganic anion exchanger activity<br>- Ionotropic glutamate receptor activity<br>- Voltage-gated calcium channel activity	GO:0006412<br>**GO:0070588**<br>**GO:0006874**<br>GO:0048666<br>GO:0007017<br>GO:0007156<br>GO:0003755<br>GO:0005003<br>GO:0005219<br>GO:0005452<br>GO:0004970<br>GO:0005245	1e-30<br>**1.3e-16**<br>**3.5e-09**<br>4.2e-09<br>5.5e-08<br>1.7e-07<br>< 1e-30<br>2.7e-21<br>6.2e-21<br>2.1e-20<br>6.3e-20<br>5.6e-16
A. jamaicensi, *M.harrisoni*, *L. yerbabuenae* and *L. nivalis* (expansions)	+ Translation<br>+ Integral component of membrane<br>+ Immune response 458<br>**+ Iron ion import membrane**<br>**+ HFE-transferrin receptor complex**<br>**+ Transferrin receptor binding**	GO:0006412<br>GO:0016021<br>GO:0006955<br>**GO:0098711**<br>**GO:1990712**<br>**GO:1990459**	<1e-30<br>6.7e-06<br>1.1e-05<br>**0.00018**<br>**5.7e-05**<br>**7.3e-05**
(contractions)	- Protein peptidyl-prolyl isomerization	GO:0000413	< 1e-30
M. harrisoni, *L. yerbabuenae* and *L. nivalis* (expansions)	**+ Protein deubiquitination**<br>+ Virion assembly 24<br>+ Structural constituent of ribosome<br>+ Thiol-dependent ubiquitinyl hydrolase<br>**+ Transferrin receptor binding**<br>**+ HFE-transferrin receptor**<br>**+ Iron ion import membrane**	**GO:0016579**<br>GO:0019068<br>GO:0003735<br>GO:0036459<br>**GO:1990459**<br>**GO:1990712**<br>**GO:0098711**	**2.8e-09**<br>0.00027<br>< 1e-30<br>3.6e-11<br>**0.0099**<br>**0.0099**<br>**0.0056**

Figure1

Click here to download Figure Figure1.png

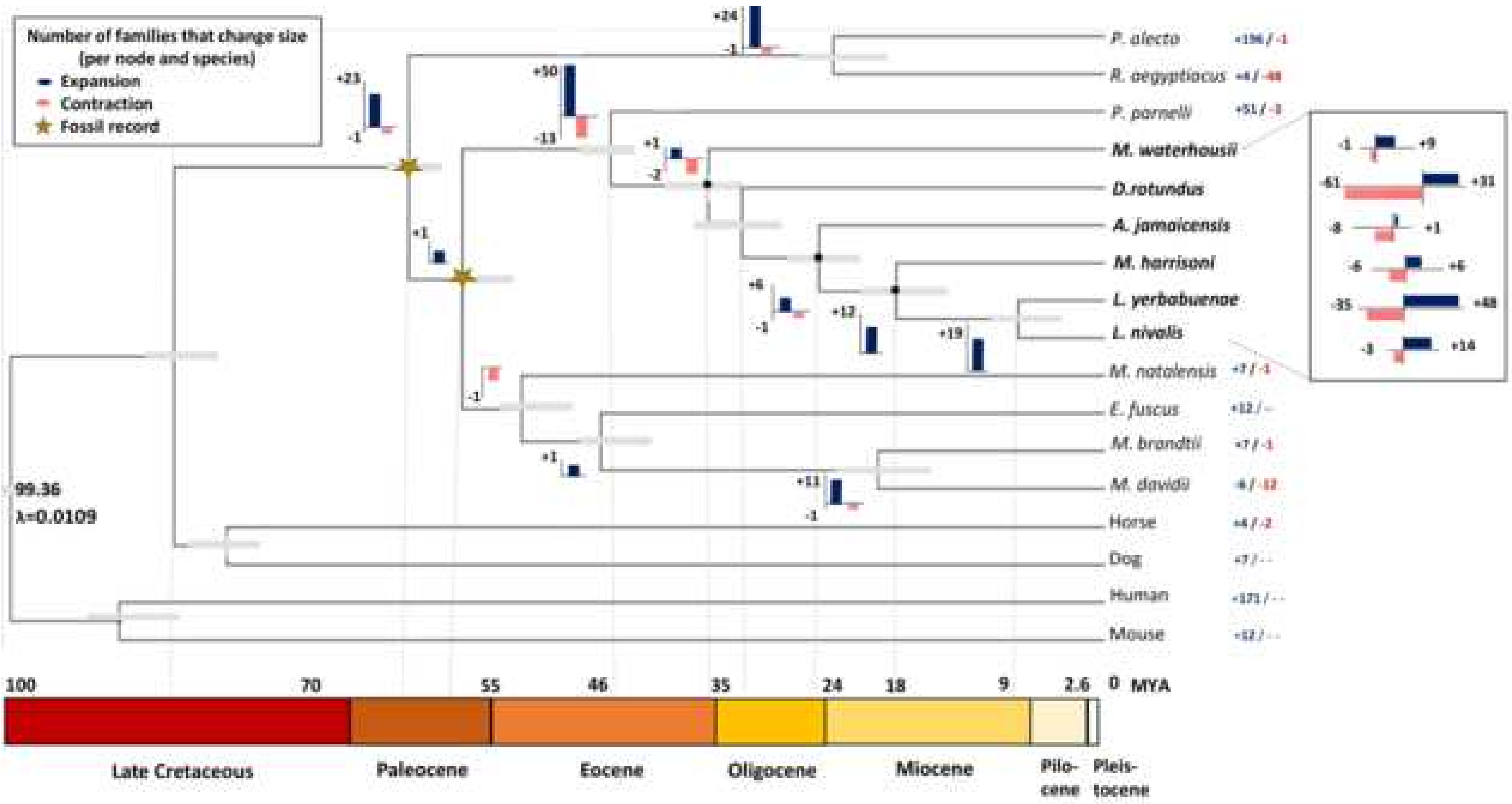

Figure2

Click here to download Figure Figure2.png

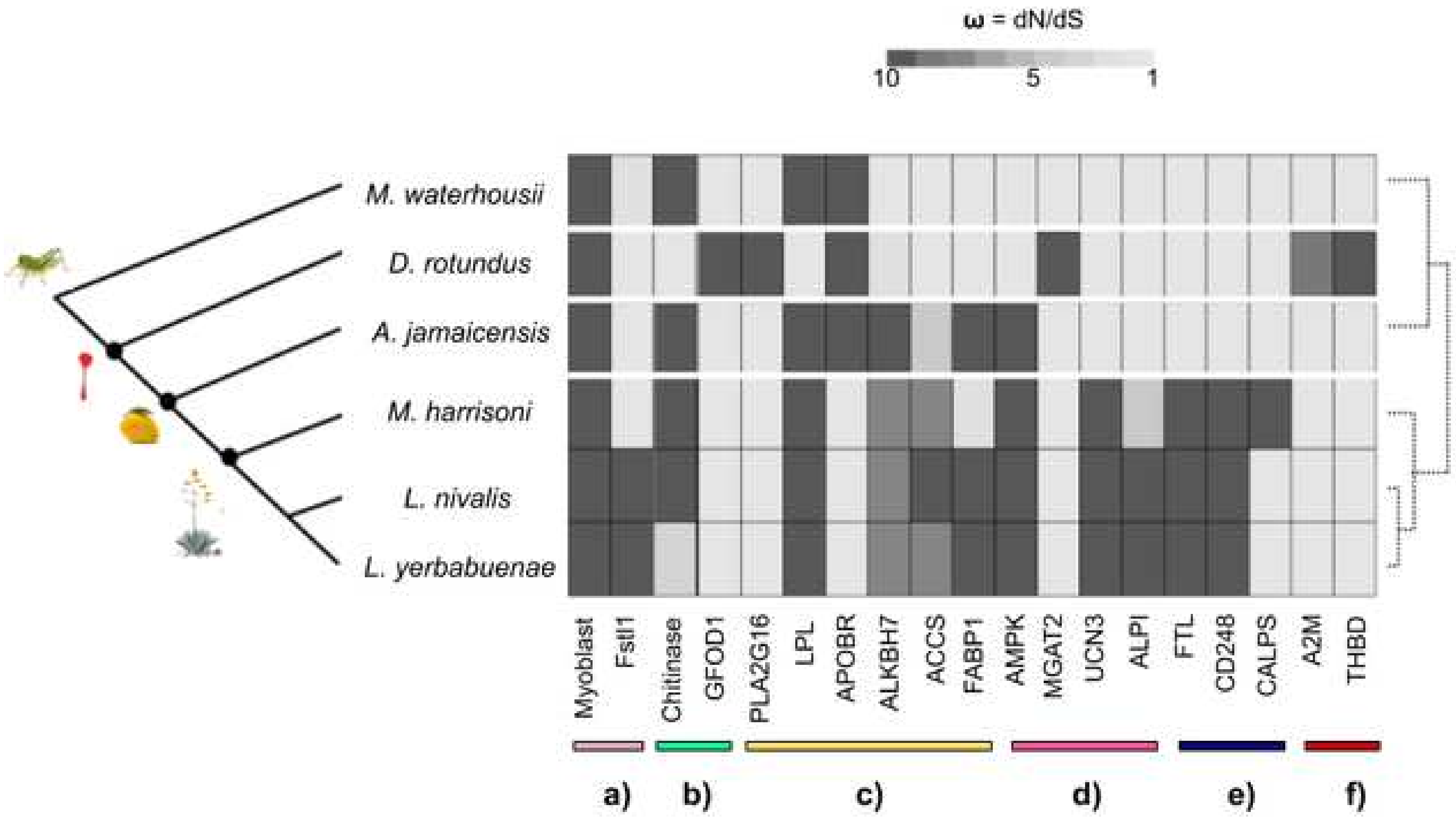

Figure3

Click here to download Figure Figure3.png

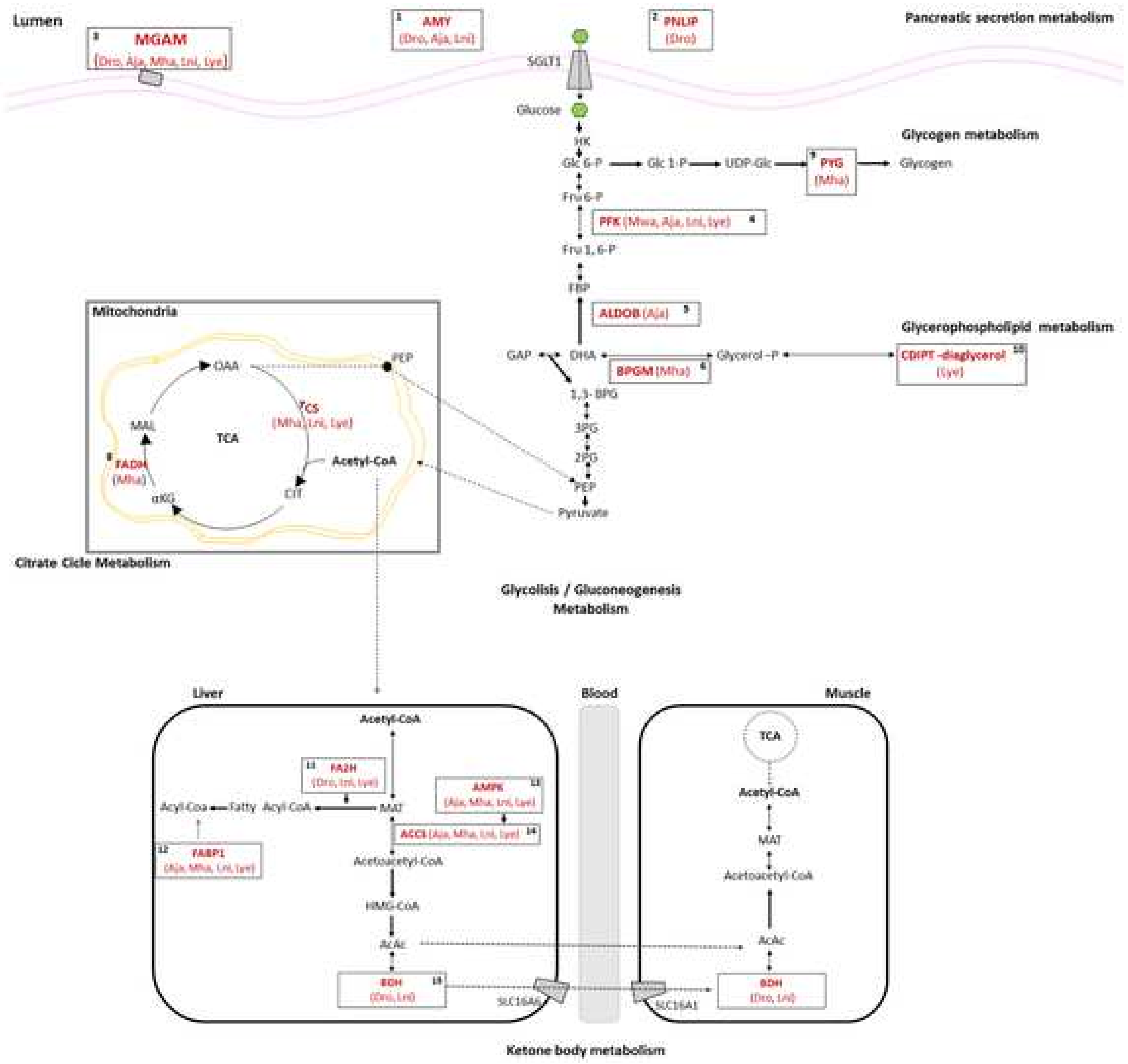

Figure4

Click here to download Figure Figure4.png

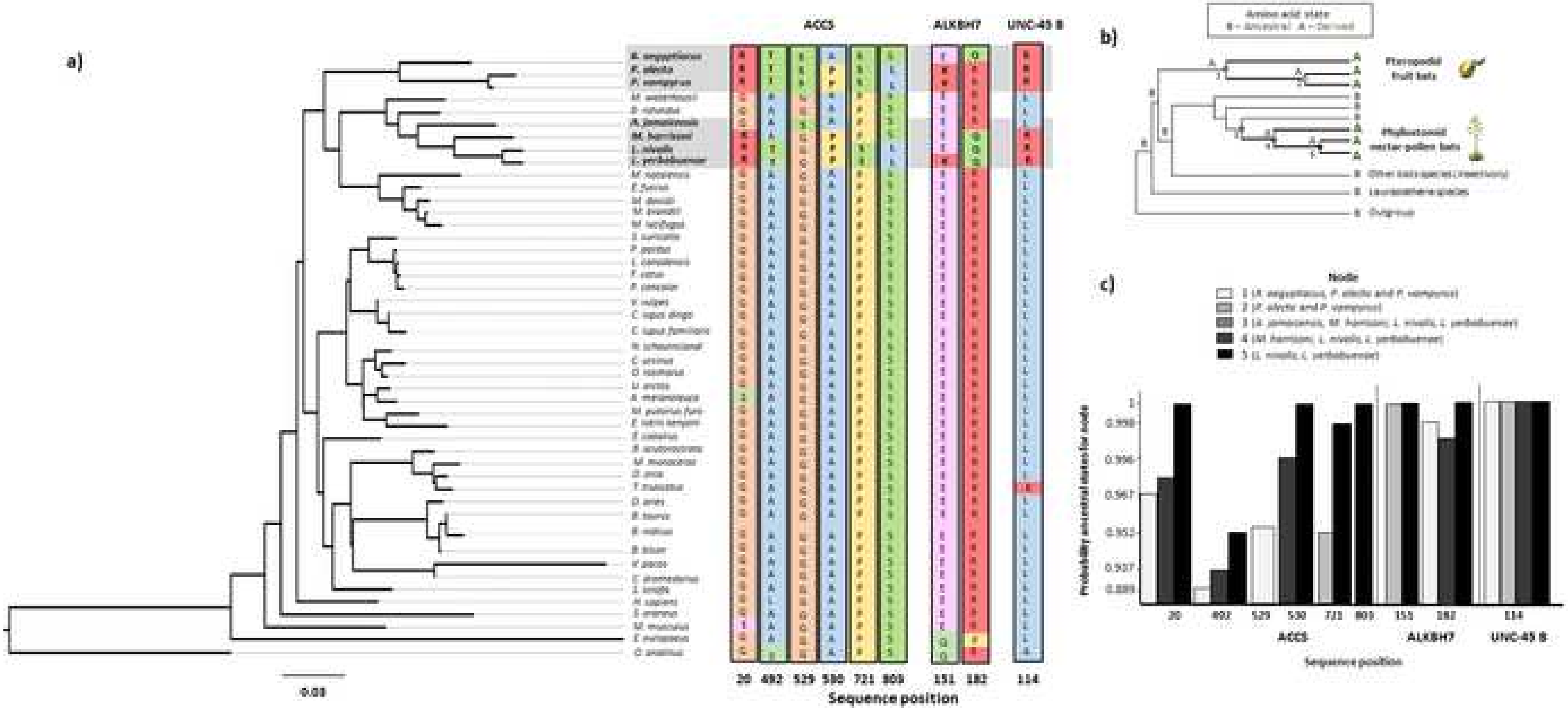

Figure5

Click here to download Figure Figure5.png

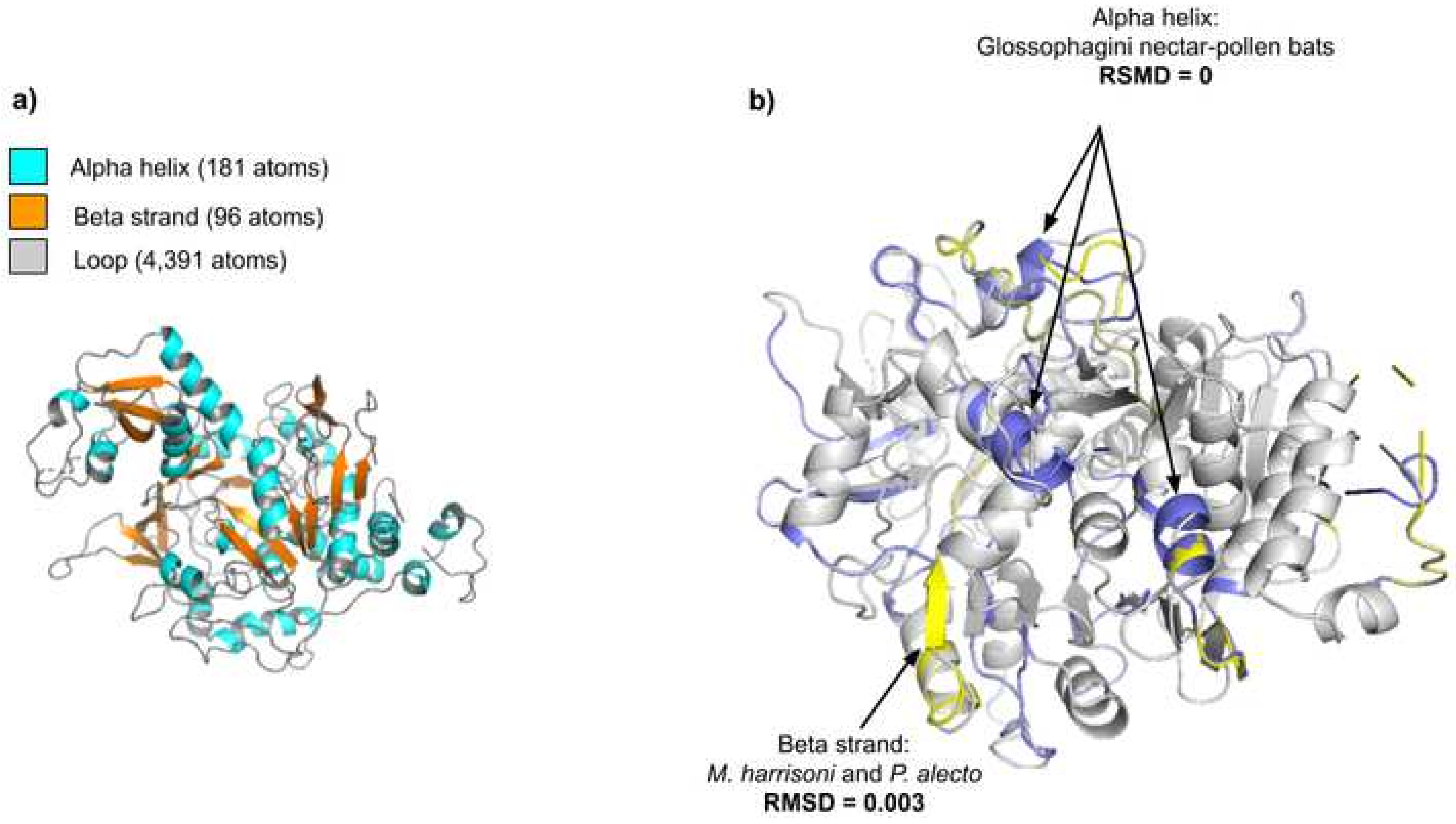

Additional File 1

Click here to access/download

Supplementary Material

AdditionalFile1.pdf

Additional File 2

Click here to access/download
Supplementary Material
AdditionalFile2.pdf

Additional File 3

Click here to access/download
Supplementary Material
AdditionalFile3.pdf

Click here to download Personal Cover CoverLetter_GigaScience.docx

April 2, 2020

Dr. Lauri Goodman
Editor-In-Chief
GigaScience
Beijing Genomics Institute,
China

Dear Dr. Lauri Goodman and Dr. Hans Zauner,

We wish to resubmit the enclosed manuscript for consideration in *GigaScience.*

Our manuscript entitled: "*Genomic consequences of dietary diversification and parallel evolution due to nectarivory in Leaf-nosed bats*", describes the genomic changes associated with the remarkably divergent feeding modes in the large adaptive radiation occurring in the Phyllostomid bat diets, which range from blood to nectar and pollen. We sequenced and assembled genomes from several species of nectar/pollen feeders, as well as from an insectivore, and a fruit-eater. We also used previously published genomic data from a vampire to assess the genomic evolutionary changes associated with different diets. We identified the expansions and contractions in gene families associated with dietary shifts and identified genes under selection. In short, we described in this paper, the genomic changes that accompany evolutionary shifts in these bats and that underlay some of the physiological mechanisms that permit these bats to subsist on such contrasting and extreme diets. Importantly, our analyses also identified convergent genomic changes between Phyllostomid nectar/pollen feeding bats, which are found only in the New World, and Old World frugivorous Pteropodids. Thus, we documented not only the divergence associated with shifts in diet, but convergence in genomic traits between bats with similar feeding habits but independent evolutionary origins.

We believe that our study is of interest to the broad readership of your journal because 1) we

document for the first time the full genomes of bats in one of the largest mammalian radiations, and these bats have undoubtedly some of the most extreme diets among mammals; 2) we provide a striking example of convergence at the genomic level.

We have carefully reviewed all the comments made by the reviewer and approached all of their concerns. In particular, we performed additional methods to detected genes under positive selection, to identify parallel molecular evolution, and we modeled an important protein for all the Phyllostomid species sequenced and other bats species. Also, we have described in detail all the used methodologies, including an additional file with the most important commands performed in this research.

The detailed and specific answer to each comment is attached in the answer to reviewer document.

We want to point out that co-authors have scrutinized a final draft and approved submission.

The manuscript includes 18 pages, 3 tables, 5 figures and three files of supplementary material (one file: tables; second file: figures and third file: methodology section (with code and scripts). We followed the author's guidelines and submission policies. This manuscript has not been previously published and the authors declare no conflicts of interest. We hope that you will find our manuscript interesting and relevant for publication in *GigaScience* and we thank you in advance for considering our study.

Please do not hesitate to contact us if you require any additional information.

Sincerely yours,

Dr. Luis E. Eguiarte

Professor

Lab. Evolución Molecular y Experimental,
Departamento de Ecología Evolutiva,
Instituto de Ecología,
Universidad Nacional Autónoma de México
fruns@unam.mx

Biol. Yocelyn T. Gutiérrez-Guerrero

Ph. D student

Lab. Evolución Molecular y Experimental,
Departamento de Ecología Evolutiva,
Instituto de Ecología,
Universidad Nacional Autónoma de México
yoss_279@comunidad.unam.mx

Click here to download Personal Cover ReviewersComments2019.docx

Reviewer's Comments (answer are in bold)

Reviewer #1:

This study has sequenced and assembled both the genomes of a number of bats showing diverse feeding strategies, and their transcriptomes. The authors explore some potential diet-related genes for evidence of evolution at the genomic level. However, their methods are incredible sparse, so I had difficulty really following how they carried out each step.

A: Thank you by the general remark on our original methods presentation.

In this version, we made a careful and detailed effort to expand all the methodologies, so the readers can replicate any analyses or follow our methodology, if they need it.

We also have proviede a new detailed methodological section in additional file 3, with the most importante scripts, programs and parameters to perform our analysis.

In addition to this, I think there are a number of possible aspects to their selection analyses that may have been overlooked, such that false-positives cannot be ruled out. I have given special attention to the codeml work in this paper, as a large number of the author's conclusions rest on it.

A: We carefully reconsidered this aspect of the analysis.
We performed a *p-value* adjust with a False Discovery Rate (FDR) correction, and we also used an additional program to detect selection, as we explain below.

I believe it is possible to address each of these issues, however I am afraid I cannot recommend this manuscript for publication until then.

Major points

1. While I think the authors have carried out a thorough assembly and analyses, I found the methods section rather light on information. There are a number of instances where the authors can and should provide details of the software used. I think that some details may need to be provided to allow other research groups follow the methodology. A large number of details are present in the supplemental information, but even that is quite sparse. I think another researcher may have difficulty recapitulating the results, even with the supplemental information.

A: Thank you.

In this new version, we have explained in detail each of the used methods. We also have incorporated into the additional file 3, a new methodology section, that includes the main scripts and parameters used to perform and conduct a specific analysis of our research.

2. The authors claim to find evidence of selection in diet-specific genes. Are any of these genes up/down regulated in the transcriptomic data? If not, do the authors have a hypothesis as to why not?

A: An interesting idea, that we will explore formally in the near future, but at this moment our data are not adequate for a robust, scientifically sound answer for this. This is because in the design of our study, the objective when we sequenced the RNA from different tissues (brain, pancreas, kidney, liver and lung), was to provide the most complete transcriptomic evidence for the largest number of gene prediction for the bat *L. yerbabuenae*.

Because of our objective, we did not include RNA-Seq replicates in our research.

However, to start exploring your question, we performed a differential gene expression analysis, comparing the five tissues with RSEM and DESeq2 packages (method blind, log fold change >= 2 and false discovery rate *pva*l < 0.05).

We identified that the liver was the tissue with the highest expression levels, and that many genes involved in carbohydrate and lipid metabolism showed high differential expression levels.

We did not include this section in the main manuscript, because we need more samples and biological replicates to perform properly the RNA-Seq analysis. But we included a table with these results at the end of file (Table for Reviewers. Differential expression analysis for the RNA-Seq data of *L. yerbabuenae*)

We hypothesized in the new version (lines 260-262) that many genes important in metabolism and food uptake, do not necessarily exhibit signals of positive selection. Indeed, we consider that it is very probable that those genes are up or down regulated. We agree that future research must carry out a transcriptome analysis, incorporating replicates and including more tissues, such as the gut and muscle.

3. The likelihood ratio test (LRT) changes from LRT to LTR throughout. Please be consistent.

A: We apoligize for the mistake. We have carefully checked all the manuscript and the supplementary sections, and changed to Likelihood Ratio Test (LRT).

4. Have the authors used some form of correction for multiple testing? Generally, selection studies using codeml require FDR correction or something analogous, to remove the risk of false positive instances of selection. FDR should be applied to each different foreground branch to rule out false-positives.

A: Thank you for the comments and suggestion.

We performed a *p-value* adjust, using the False Discovery Rate (FDR) correction, based on the likelihood ratio for our results (CODEML and Hyphy absrel). Additionally, for CODEML,

we considered only those genes with a Bayes Empirical Bayes >= 99% as positive selected genes.

[Lines 387-402 (Methods), Additional file 3].

5. In their selection studies, The authors include 3 other bats, all Pteropoididae, and hence frugivorous. However, to solidify their results, they need to look for selection for the same genes, designating some insectivorous bat species as forgeound, to show that selection is not simply an artefact found in all bats for that gene. Until this is done, they cannot explicitly state that they have found genomic evidence of dietary shifts. The authors should also designate some non-bat foreground branches to see if they happen to find selection in the same genes for other taxa that are neither bats nor frugivorous or nectivorous.

A: Thank you.

We agree that this is a very relevant comment, and we paid special attention to solve it. To identify positive selection for each Phyllostomid bat species, we considered each Phyllostomid bat as our foreground branch (for CODEML). Then we performed this analysis for all the orthologous single-copy gene clusters (each cluster included from 12 to 30 sequences of different mammals species). This way, we identified genes under positive selection exclusive for a specific Phyllosotmid species. But also, we detected some genes under positive selection shared by two o more species.

[Lines 390-394 (Methods), Additional file 3].

Based on these results, we classified these genes using two criteria: 1) Genes involved with metabolism (lipids, carbohydrates, iron, calcium and food uptake); and 2) Genes related to a specific diet. For example, the vampire showed a signal of positive selection for genes involved in coagulation pathways.

[Lines 154-186 (Analysis)].

On the other hand, we conducted a Hyphy aBSREL analysis to test if positive selection has ocurred in all or in a portion of branches, testing all the branches (including bats and non-bats).

[Lines 401-403 (Methods), Additional file 3].

6. The species representation in their selection tests are incredibly saturated by bat taxa. I realise this is a study specifically relating to bats, however the authors have only one laurasiatherian outgroup in their data (Specifically the dog). Therefore, there will be a stark difference in the likelihood-based ancestral reconstruction of codons at the internal nodes between bats and other taxa. In order to prove their findings are not simply an artefact of poor non-bat representation, they should consider adding more non-bat laurasiatherian taxa.

A: Thank you.

As in the previous point, to answer this question, we used many other taxa. In this version, we included in a new analysis of parallel evolution and of the ancestral reconstruction sequence state for a total of 49 mammals species.

[Lines 370-373 (Methods), Additional file 1, Table S12].

In these new analyses, we used the protein information for a total of 46 Laurasiatheria species, composed by 15 bats species (including our Phyllostomids samples), and 3 non-laurasiatheria mammals: human, mouse and *Ornithorhyncus*.

[Additional file 1, Table S12].

7. Can the authors explore the 3D structure of the protein sequences in some of their genes showing selection? As it stands, they are relying on a significant p-value in selection tests to indicate that there is diet-specific evolution happening. They mention radical amino acid substitutions, do nothing to further explore or validate this.

A: Thank you for this idea.

We modelled the secondary and tertiary structure of the protein: Acetoacetyl CoA Synthetase (*ACCS*), for *M. waterhousii, D. Rotundus, M. harrisoni, L. nivalis, L. yerbabuenae, P. alecto* and *H. armirger* (insectivorous bat from the Old World), with Phyred 2 software.
This is detailed in the Methods section.

[Lines 449-452 (Methods), Additional file 1, Table S11, Additional file 2, Fig. S4].

Additionally, to identify differences in the protein structure, we compared the secondary and tertiary structure between the nectar-feeder bat *L. yerbabuenae* and the other bats, using the software PyMOL. We calculated the RSMD score between pairs (the nectar-fruit bats and *M. waterhousii* and *D. rotundus*)

[Lines 452-455 (Methods), Additional file 1, Table S10].

We found that tertiary protein structure exhibited high similarity in all of our comparisons. However, we identified little differences in an alpha-helix region , that it may be important in the protein function for the nectar-pollen feeders *L. yerbabuenae, L. nivalis* and *M. harrisoni*; and a beta strand region shared only between *M. harrisoni* and *P. alecto*.

[Lines 217-220, Figure 5 (Analysis)].

We also suggest that future studies must evaluate the regulation of this gene, and performed a RNA-Seq experiment with biological samples to identify if this gene is down or up-regulated.

[Lines 260-262 (Discussion)].

8. The authors state that they have found convergent evolution, based on finding selection in the same genes. However, Can they rule out the possibility of an ancestral mutation being maintained, rather than convergent evolution? For example, in Figure 4, sequence position 181 is "Q in, *R. aegyptiacus* and a number of the author's target species. However, simply by looking at the position, one cannot tell if: (1) *R. aegyptiacus* and the other species with "Q" have retained the ancestral amino acid, and in fact the other bat species have evolved and converged, (2) If true convergence has happened, or (3) it is an artefact of drift; especially given that this site doesn't show up as having a significant BEB score in their branch-site tables in the supplemental. Perhaps reconstructing the ancestral sequence for these genes at each internal node might help shed some light on this? Until then, unfortunately I must remain sceptical about the author's instances of "convergence", based purely on observations of amino acids in the alignments. Perhaps Clade model C can help identify convergent evolution for these data?

A: We agree that this is a complicated issue that warranted a better analysis. To tackle it, we performed a new computational approach, specifically to identify parallel amino acid substitutions between pairs of bat lineages from Glossophaginae bats (*L. yerbabuenae*, *L. nivalis* and *M.* harrisoni), and from fruit-nectar feeding Pteropodids (*R. aegyptiacus, P. alecto* and *P. vampyrus*).

First, we obtained all orthologous single-copy genes for at least one Glossophagini and one Pteropodid, using a database including sequences from 12 to 30 mammals species.

[Lines 413-16 (Methods)].

Then, we aligned all those orthologous single-copy genes groups, with PERL and BASH scripts. We looked for exclusives amino acids substitutions shared in at least one Glossophaginii and Pteropodid species (putative parallel sequences), as described in Additional file 3.

For all those putative parallel sequences, we reconstructed their ancestral sequences state, using two different algorithms: CODEML and FASTML. Both programs reconstruct the ancestral sequence state at each internal node.

[Lines 417-427 (Methods)].

Finally, based on the information of each internal node at the parallel substitution position, we classified as a parallel substitution those sites with: 1) a radical amino acid change, exclusively for these bat lineages; 2) if the internal node exhibited the same parallel change; and 3) we considered as a significant amino acid substitution, those internal nodes with a reconstruction probability > 85%.

[Lines 198-200 (Results), 428-437 (Methods), Figure 4].

9. There are a number of instances in the paper where the English is not very good.

A: We followed your suggestions, and we have carefully checked the grammar and corrected the grammar mistakes.

Minor points

Data descriptions
Line 113: genome => genomes

A: We changed it.

[Line 115].

Analyses

Line 137 – 144. The authors make sweeping broad statements about their results. I think that it would be interesting to give one or two examples to back up these statements.

A: We included the following examples (lines 141-147):

"Furthermore, across the Phyllostomid bats many gene families exhibited changes with feeding habits, for example, the Phyllostomid node had a contraction related to the lipid metabolism. The blood-feeder lineage had a significant gain on gene families involved in the regulation of appetite and process for nitrogen acquisition, but also this lineage showed many contraction events involved in calcium metabolism (Table 3). The fruit and nectar feeding bats exhibited many expansion events in iron metabolism regulation pathways (Table 3). "

Methods
Line 279 "and RNA-seq" is shoe-horned In here. The authors should elaborate a litte more on the specifics of this. Do the author mean transcriptome (I also refer to line 281 in the text too). Can the authors add in a single line here re-iterating why special attention as given to *L. yerbabuenae* over the other species, with respect to RNA-seq analyses?

A: Thank you, we changed the ms. as suggested.

[Lines 304-307].

Line 281/282: The authors begin 2 consecutive sentences with "and/also we sequenced", perhaps they can use something different?

A: Thank you.

[Line 304].

Line 287: assembly genome => genome assembly.

A: We made the change.

[Line 315].

Line 287: I'm assuming these steps where applied to all species? Please state xplicitly if it was.

A: We detailed this section, thank you.

[Lines 350-354].

Line 287: Can the authors please state the *k*-mer length used. Presumably there is one, given a *de novo* assembly program.

A: We detailed this.

[Line 316].

Line 288: In one sentence, the author have given 3 different programs to do 3 different things. Could they elaborate on what each software does, and any important parameters used? I don't think a reader would be able to replicate these steps without specific knowledge. Same goes for BUSCO.

A: We are sorry about out original compact explanations. We state now (lines 315-322):

"The genome assembly was constructed *de novo* with Platanus v. 2.4.3 (44), using a heterozygous value = 0.04 (-u 0.04) and a initial kmer=32. To accurate, optimize and extend the genome assembly, we performed a scaffolding with MeDuSa software (45, 46). Finally, we used Pilon for correcting bases and polish the genome assembly (47).
We evaluated the genome assembly metrics (total length, number of scaffolds, number of contings, L50, N50, and others). Moreover, with BUSCO v3 and the Mammalia odb9 database (48) we evaluated the measure for quantitative assessment of the genes content into de genome assembly. "

Line 293: The authors should explicitly state "TE" as the acronym after the "transposable elements" in the previous line.

A: Thank you, we have explained this in line 325.

Line 303: Are SNPs only identified in L. yerbabuenae? I found this section difficult to follow!

A: We detailed, and also we explained this issue in the Additional file 3.

[Lines 337-354, Additional file 2, Fig. S3 and Additional File 3].

Line 310: How were these single copy orthologs chosen? Where they from OrthDB?

A: We modified this section, because we created a big database with the protein information of 49 species. To obtain the orthologous sequences using this database, we used the program Proteinortho. This program has a fast and efficient algorithm to search orthologous sequences for big databases.

[Line: 375-376].

Line 322: The authors mention two-ratio branch model, yet their supplemental methods mention branch site. I think based on their results that they have carried out branch site, therefore this line is categorically incorrect.

A: This is correctly stated now (line 369):

dN/dS analysis using a branch-site model

Line 325: Can the authors state what alignments software they used?

A: We mentioned the aligner tools in the lines 380 and 419.

Line 328: The authors alternate between "CODEML" and "Codeml", can they please be consistent?

A: We have carefully checked and changed to CODEML in all the manuscript.

Line 345: What software did the authors use?

A: This is explained now in lines 414-420:

"Based on the previously inferred orthologous genes, we extracted all single copy genes shared by the Glossophagini (Glsp): *M. harrisoni*, *L. nivalis*, *L. yerbabuenae;* and the Pteropodids (Ptrp): *P. alecto*, *P. vampyrus* and *R. aegyptiacus.* Each single copy gene cluster was composed from 12 to maximum 30 sequences. We obtained 1,918 clusters of orthologous sequences (including at least one Glsp and one Ptrp). Each cluster was aligned using PRANK (74) and we constructed their corresponding phylogenetic tree with RAxML (parameter -m PROTCATLG) (69)."

Reviewer #2:

Based on multiple full genomes from bats with different diets, this comparative genomics study aims to identify genomic changes associated with these extreme diets in a large adaptive Phyllostomid group. And by including published genomes, the authors also examine the genomic convergence between Phyllostomid nectar/pollen feeding bats and old world frugivorous Old World Pteropodids which have similar diets but independent evolutionary origins. The authors found some interesting genes associated with diversification of diets and convergence.

A: Thank you for your comments.

The logic of this whole MS is very good and centered by three predictions in Introduction, Results and Discussion.

A: Thank you again for the kind comment.

However, most of their predictions or conclusions are based on positive selection analysis using PAML with branch-site model. This is my major concern because results from PAML are not always reliable. I suggest that authors should conduct other selection analyses (at least two) to get consistent results.

A: Thank you for your comment.

We have used an extra program to identify signatures of positive selection: Hyphy aBSREL. We have classified as positive selected genes those that had a *p-value* =< 0.05 in both programs: CODEML and Hyphy aBSREL.

[Lines 400 – 406 (Methods)].

In addition, for convergence analysis at the genomic level, I suggest to use some formally statistic methods, such as methods in Parker et al. Genome-wide signatures of convergent evolution in echolocating mammals. Nature. 2013, 502, 228.).

A: Thank you for your concerns.

We reviewed recent studies and their methodologies to identify signals of parallel evolution in mammals and plants.
For example:

Lee J, Lewis KM, Moural TW, et al. Molecular parallelism in fast-twitch muscle proteins in echolocating mammals. *Science*. 2018;4:eaat9660.

Xu S, He Z, Guo Z, et al. Genome-Wide convergence during evoluton of mangroves from woody plants. *Mol Biol Evol*. 2017;34:1008-1015.

Bailey SF, Guo Q, Bataillon T. Identifying drivers of parallel evolution: a regression model approach. *Genome Biol Evol*. 2018; 10:2801-2812.

Based on the Methods and Results of these manuscripts, we have taken into account the importance of obtaining an accurate ancestral sequence reconstrunction and signals of positive selection. These two issues are the most important to identify molecular parallelims or molecular convergence.

To improve our methodology, we performed and followed three criteria:

1) Analysis to detect positive selection (using two programs and a *p-value* correction by FDR) [Lines 405-406].

2) Ancestral sequence reconstruction state in internal nodes, using two programs. We also calculated the probability of amino-acid substitution in the internal nodes (Fig. 4C, Additional file 1, Table S9).

3) We performed an analysis of possible drivers of parallel evolution, evaluating gene and protein length, %GC, *dN/dS* rate and isoelectric point (Additional file 1, Table S13).

Finally, I can not get the idea in "gene family analysis". I wonder whether this analysis is based on 132 genes which was used to build the phylogenetic tree ? (Maybe I miss something here?).

A: We apologize for our original explanations. We detailed these results.

[Lines 135-139 (Analyses)]:

"To understand genomic evolution and to trace changes associated with dietary diversification and specialization, we reconstructed a phylogenomic tree using 132 single-copy orthologous genes (61,331 amino acids sites), which was calibrated using two fossil dates (16-18). Based on the phylogenomic tree, we analyzed the dynamics (expansion and contractions) for 22,388 gene families across the Phyllostomid bat genomes."

Table for Reviewers. Differential expression analysis for the RNA-Seq data of *L. yerbabuenae*

Id	Log fold change	*p-value*	FDR	TPM	Function
			**Brain**		
unigen_4901	-7.251990571	0.000216782	0.02356759	172.424	Solute carrier family 1 member 3 (SLC1A3)
unigen_10595	-9.3213293415	0.000431228	0.035833573	80.719	Glutamate ionotropic receptor kainate type subunit 2 (GRIK2)
unigen_15295	-10.2271671512	8.68E-05	0.012835147	149.824	Glutamate ionotropic receptor AMPA type subunit 2 (GRIA2)
unigen_22229	-9.3568982466	0.000405169	0.034825529	82.387	Glutamate ionotropic receptor delta type subunit 1 (GRID1)
unigen_3215	-5.8792261643	0.000420342	0.035356255	3631.939	Aldolase, fructose-bisphosphate C (ALDOC)
unigen_96	-7.4961722171	6.2544E-05	0.009996393	385.022	Lactate dehydrogenase B (LDHB)
			**Pancreas**		
unigen_11846	6.9046387437	0.000318162	0.033014784	163.536	B-cell scaffold protein with ankyrin repeats 1 (BANK1)
			**Kidney**		
unigen_12548	9.6127149411	0.000257906	0.037880397	101.35	Solute carrier family 5 member 12 (SLC5A12)
unigen_15693	6.4710622278	0.000361756	0.048638025	201.069	Solute carrier family 7 member 11 (SLC7A11)
unigen_11573	6.6969496854	0.000165244	0.028760449	345.986	Solute carrier family 2 member 5 (SLC2A5)
unigen_3216	7.9440626366	1.05E-05	0.021021083	1826	Aldolase, fructose-bisphosphate B (ALDOB)
unigen_5105	9.2227329979	5.90E-06	0.026932024	713.195	Phosphoenolpyruvate carboxykinase 1 (PCK1)
unigen_96	6.6527908257	0.000259383	0.037880397	228.196	Lactate dehydrogenase B (LDHB)
			**Liver**		
unigen_305	9.6046284378	0.000264718	0.026896527	97.147	Solute carrier family 2 member 2 (SLC2A2)
unigen_10221	6.1682218476<br>-7.7247788625	0.0003217093<br>5.37967302346053E-005	0.0308314061<br>0.0094967252	367.14	Insulin like growth factor binding protein 2 (IGFBP2)
unigen_10535	6.8255755692<br>-10.1482691829	0.000469337<br>0.0001017612	0.0377459484<br>0.0150926693	127.221	Aldehyde dehydrogenase 1 family member L1
unigen_1072	10.8984351101<br>-6.3460572414	0.000026329<br>0.0003644543	0.0054449563<br>0.0487147513	237.617	Pyruvate kinase, liver and RBC (PKLR)
unigen_12621	12.3672690676	1.84E-06	0.000971083	659.05	Glucose-6-phosphatase catalytic subunit 2 (G6PC2)
unigen_14961	9.7188199196	0.000216371	0.02356759	104.878	ATP binding cassette subfamily C member 6 (ABCC6)
unigen_15951	9.9350731545	0.000147491	0.018608906	121.881	Acyl-CoA synthetase long-chain family member 5 (ACSL5)
unigen_17577	-9.8603053353	0.000171549	0.021773582	104.185	Regulator of G-protein signaling 16 (RGS16)
unigen_17591	-6.6813433458	0.000347882	0.035053723	177.917	Solute carrier family 16 member 14 (SLC16A14)
unigen_18670	10.6548081624	4.08E-05	0.007285091	201.109	Aquaporin 9 (AQP9)
unigen_20513	6.9029061128	0.000409629	0.034990234	134.46	Phosphoenolpyruvate carboxykinase 2
unigen_22362	6.4736318871<br>-7.4765405336	0.0003918874<br>0.0001592441	0.0345020499<br>0.0286989491	188.239	Solute carrier family 41 member 2 (SLC41A2)
unigen_2242	8.1121792838	4.71E-05	0.008161434	309.718	Solute carrier family 27 member 6 (SLC27A6)
unigen_3637	-10.8869951495	2.72E-05	0.005986135	212.303	Solute carrier family 6 member 11 (SLC6A11
unigen_3658	-9.9146956321	0.000153863	0.020108817	108.319	Solute carrier family 38 member 3 (SLC38A3)
unigen_38603	13.6753969476	1.71E-07	0.000225979	1631.77	Fructose-bisphosphatase 1 (FBP1)
unigen_4275	-9.5178339857	0.0003113	0.03287324	81.574	Solute carrier family 22 member 9-like
unigen_4648	10.7041660734	3.73E-05	0.006952204	208.035	Solute carrier family 37 member 4 (SLC37A4)
unigen_6395	-11.4353569201	6.4E-08	0.000187836	4805.82	Solute carrier organic anion transporter family member 1B3-like
