# Supplementary material for: Genomic consequences of dietary diversification and parallel evolution due to nectarivory in leaf-nosed bats
Source: Gigascience. 2020 Jun 6;9(6):giaa059. doi: 10.1093/gigascience/giaa059 (PMC7276932; doi:10.1093/gigascience/giaa059)
Supplement: giaa059_GIGA-D-20-00098_Revision_2 [file giaa059_giga-d-20-00098_revision_2.pdf]

GigaScience

Genomic consequences of dietary diversification and parallel evolution due to nectarivory in Leaf-nosed bats.

--Manuscript Draft--

**Manuscript Number:**	GIGA-D-20-00098R2
**Full Title:**	Genomic consequences of dietary diversification and parallel evolution due to nectarivory in Leaf-nosed bats.
**Article Type:**	Research
**Funding Information:**	Consejo Nacional de Ciencia y Tecnología (177) — Professor Luis E. Eguiarte
**Abstract:**	Background<br><br>The New World Leaf-Nosed bats (Phyllostomids) exhibit a diverse spectrum of feeding habits and innovations in their nutrient acquisition and foraging mechanisms. However, the genomic signatures associated with their distinct diets are unknown.<br><br>Results<br><br>We conducted a genomic comparative analysis to study the evolutionary dynamics related to dietary diversification and specialization. We sequenced, assembled and annotated the genomes of five Phyllostomid species: one insect-feeder (Macrotus waterhousii), one fruit-feeder (Artibeus jamaicensis), and three nectar-feeders from the Glossophaginae subfamily (Leptonycteris yerbabuenae , Leptonycteris nivalis and Musonycteris harrisoni), also including the previously sequenced vampire Desmodus rotundus . Our phylogenomic analysis based on 22,388 gene families displayed differences in expansion and contraction events across the Phyllostomid lineages. Independently of diet, genes relevant for feeding strategies and food intake experienced multiple expansions and signatures of positive selection. We also found adaptation signatures associated with specialized diets: the vampire exhibited traits associated with a blood diet (i.e., coagulation mechanisms), whereas the nectarivore clade shares a group of positively selected genes involved in sugar, lipid, and iron metabolism. Interestingly, in fruit-nectar feeding Phyllostomid and Pteropodids bats, we detected positive selection in two genes: AACS and ALKBH7, which are crucial in sugar and fat metabolism. Moreover, in these two proteins we found parallel amino-acid substitutions in conserved positions exclusive to the tribe Glossophagini and to Pteropodids.<br><br>Conclusions<br><br>Our findings illuminate the genomic and molecular shifts associated with the evolution of nectarivory and shed light on how nectar-feeding bats can avoid the adverse effects of diets with high glucose content.
**Corresponding Author:**	Luis E.Eguiarte<br><br>MEXICO
**Corresponding Author Secondary Information:**	
**Corresponding Author's Institution:**	
**Corresponding Author's Secondary Institution:**	
**First Author:**	Yocelyn T. Gutiérrez-Guerrero, PhD Candidate
**First Author Secondary Information:**	
**Order of Authors:**	Yocelyn T. Gutiérrez-Guerrero, PhD Candidate

Powered by Editorial Manager® and ProduXion Manager® from Aries Systems Corporation

	Enrique Ibarra-Laclette, Doctor
	Carlos Martínez del Río, Doctor
	Josué Barrera-Redondo, PhD Candidate
	Eria A. Rebollar, Doctor
	Jorge Ortega, Doctor
	Livia León-Paniagua, Doctor
	Araxi Urrutia, Doctor
	Erika Aguirre-Planter, Doctor
	Luis E. Eguiarte, Doctor
**Order of Authors Secondary Information:**	
**Response to Reviewers:**	Reviewer's Comments<br><br>This study has sequenced and assembled the genomes of a number of bats showing diverse feeding strategies, and explored the evolutionary adaptations underpinning dietary niche specialization. The authors have fully addressed my initial concerns regarding selection tests, have provided a wealth of data to support their findings and have also provided an incredibly thorough guide on their methods that I think will benefit many researchers. I have some small, largely trivial issues below. Most these are concerned with the written English rather than the methods used. I am therefore happy to support this paper for publication, pending these minor changes.<br><br><br>We appreciate your support and comments.<br>We are certain that your suggestions have been crucial to improve and make more accurate our manuscript.<br>In particular, we appreciate your remark on the changes in description of the methods, that we hope will be useful.<br><br>Minor points<br><br>Line 163: insects -> insect's<br><br>Thank you for your correction, we made the change.<br>Line 165<br><br>Line 163: I feel like trehalOse should be the sugar in insect blood, rather than trehalase, if trehalase is the enzyme that degrades it.<br><br>Thank you for your observation, we are talking about trehalose, we are sorry for the mistake.<br>Line 165<br><br>Line 231: those than -> those that<br><br>Thank you, we changed it.<br>Line 232<br><br>Line 233: This relates to my comment on line 163. Does trehalase digest trehalase in insects, such that one enzyme degrades another enzyme, or does trehalase degrade the trehalose sugar?<br><br>We appreciate your comment, as it is very relevant. Most of the vertebrates have the capacity to digest dietary trehalose with the membrane bound intestinal enzyme trehalase.<br>We have modified this section.<br>Lines 234-235<br><br>Line 233: Do the authors have any ideas as to why the ability to digest insects may be

maintained in bats, nit not the ability to digest the trehalase/trehalose sugar/enzyme in insect blood?

This issue is really interesting, as a parallel change seem to have happened in birds. Even those specialist bats, such as hematophagous and nectar-feeding species have the capacity to digest insects exoskeletal chitin. We consider two possibilities for the loss of trehalase. One is that the main dietary value of the insects is for lipids and proteins, and energy (as sugars) would be less important, and once the ability to digest trehalose is loss, there is no way they can recuperate it. On the other hand, we suggest that gut microbiome plays an important role to digest trehalose. The microbiome role is discussed in line 291-296.

Line 260: that it may -> that may

Thank you.
Line 262

Line 277: When the authors mention convergent evolution here, do they mean specifically dietary genes or the genome and physiology of the bat as a whole? Please clarify.

Thank you. We meant specifically parallel evolution due to nectar-feeding dietary specialization.

"Our findings suggest that parallel evolution due to nectar-feeding dietary specialization is likely a consequence of high metabolic demands required for foraging on flowers and fruits."
Lines 279-280

Line 318: I have not seen 'accurate' used in the context the authors use it here. Perhaps another word such as 'validate'can be used instead?

We apologize for the mistake.

" To optimize and extend the genome assembly"
Lines 321-322

Line 354 Perhaps consider "Repeatmasker pipeline" rather than "pipeline of repeatmasker"

We appreciate your suggestion.
Line 358

Line 373: I think "proteins" should be "protein's"

Thank you.
Line 377

Line 373: DIAMOND is also a program, so consider saying "programs DIAMOND and Proteinortho"

Thank you, we made the change.
Line 381

Line 380: "paralogous, sequences" -> "paralogous sequences"

Thank you, we made the change.
Line 383

Line 384: Were the poorly aligned regions removed based on a visual inspection or something like Gblocks?

We carried out a visual inspection and calculated the alignment length with a bash script.

Powered by Editorial Manager® and ProduXion Manager® from Aries Systems Corporation

“Each cluster was aligned with MAFFT aligner tool (67), we retained alignment sequences where the length is within 80 to 120% relative to the human and mouse sequences, and poorly aligned regions were removed by a visual inspection. ”
Line 386-388

Line 387: I think “RAxML tool” can just be “RaxML”

We appreciate your suggestion.
Line 391
Line 390: The authors describe how they calculated “synonymous sites and nonsynonymous sites (dN/dS) rates, and the average ratio of substitution per site (ω=dN/dS)”, however I would have assumed that these were essentially the same things, and don’t need to be stated twice as it is written, at least as far as dN/dS and w=dN/dS is concerned.
Thank you for your observation, we estimated the ratio of substitution per site.
Line 395
Line 402: No need for the “,” after the word aBSREL.

Thank you.
Line 407

Line 417: “was composed from 12 to maximum 30” -> “was composed of between 12 and a maximum 30” perhaps?

Thank you for your suggestion.
Lines 422-423

Line 424: “the accurate” –> “ the accurate ones”

Thank you.
Line 429

Line 424: “program” -> “programs”

Thank you.
Line 430

Line 442: “Independantly” –> “Independent”

Thank you.
Line 453

Line 446: The phylogenetic tree section seems out of context here, as trees have been generated throughout the methods up to this point. The authors should consider moving this section or being explicit as to the function of the tree generated in this section.

Thank you, we re-ordenized this section.
Lines 446-450

Line 451: The authors should consider adding one line at the start to give context for the reasoning behind modelling, for example “To explore the effects of selected sites on the protein 3D structure..” or something similar.

We appreciate your suggestion.

“To explore the effects of positive selection and the radical amino acid substitutions, we modeled the second and tertiary structure of the protein Acetoacetyl CoA Synthetase (ACCS) for M. waterhousii, D. rotundus, M. harrisoni, L. nivalis, L. yerbabuenae and P. alecto. ”
Lines 459-461

Table 2: there was an odd symbol in the brackets under nucleotide diversity on my

screen. Double check that it is not an error!

Thank you, we modified this section.

Figure 1:Purely out of curiosity, do the major expansion events correlate with known climate events occurring in the various geological epochs?

This is an interesting question., but we have not formally explored this. In the case of the nectar-pollen feeder clade, we found an important gene family expansion event. This is interesting, because the divergence of the Glossophagini bats started in the Mid-Miocene from 21 to 7 Mya, coinciding with some environmental changes and the increase of food resources at the “Climatic Optimum” period.

On the other hand, the major gene family expansion was detected at the Microchiroptera node, in the Eocene period, where the Earth responded to higher levels of carbon dioxide and an increment in the temperature, warmer than today.

We will analyze in detail these gene families expansions in a future manuscript, incorporating some analysis such as phylostratigraphy and gene family calibration. Thank you for the comment.

Additional File 1, Table S1-6: Some numbers have “,” in them, others don’t. Please ensure they all do.

We apologize for the mistake, we made the change.

Table S6: please change LTR to LRT. Are these p-values corrected for multiple testing? It would also be helpful to highlight significant ones with a “*” or something similar.

We included a column with the p-values adjust by FDR and we highlighted those significant genes.

Reviewer #2: The authors made a great effort to make changes in this revision based on reviewers' comments. I generally agree with the authors for their responses to my previous comments. However, as I look through the whole MS, I found many minor errors which can be avoided if authors are meticulous during writing. So I strongly recommend the authors to reread the whole MS carefully to correct possible minor errors.

We appreciate your support and comments. We have read carefully all the manuscript, and double-checked.

Below are some examples.

In "Rapidly evolving genes across the whole genome", the authors did not provide the specific total number of positively selected genes, and also some words about enrichment analysis.

We appreciate your observation, we have incorporated more information.
Lines 151-155.

“For all Phyllostomid bats, we identified 42 genes with robust signals of positive selection (FDR $p < 0.05$). According with the enrichment analysis, most of the adaptive genes are related to immune response, DNA repair, inflammatory response, RNA catalytic process and genes that mediate muscle function (such as Myoblast and PAMR1) (Fig. 2; see Additional file 1, Table S6-TableS8) (19).“

In Table S6, LTR is still used (another reviewer had pointed out this mistake).

We deeply apologize for this repeated mistake.
We changed LTR to LRT.

Powered by Editorial Manager® and ProduXion Manager® from Aries Systems Corporation

	In Additional file 1, "Table S7" was wrote as "Table S8", so there are two "Table S8".<br><br>We are sorry and we changed the number of this figures.<br><br>"Table S6. LTR construction and ω ratio", I did not see results aboutω ratio, but just P values.<br><br>We appreciate your observation.<br>We have incorporated the p-value correction and highlighted those significant genes.<br><br>Table S7 "GO enrichment for those positive selected genes for each Phyllostomid specie", the last word should be "species"<br><br>Thank you, we modified it.<br><br>Line 231, "than" should be "that"<br><br>We apologize for the mistake, we change it.<br>Line 232<br><br>Line 359, what software was used to construct the phylogeny based a total of 132 genes? I find it in the additional file 3, PhyML3. I think that the authors should mention this in the main text. In addition, the authors did not mention that whether these 132 genes are concatenated or not in building the tree.<br><br>Thank you, we included the information in the main text.<br><br>"A total of 132 single-copy orthologous genes (61,331 amino acids sites), across 18 mammals were concatenated to reconstruct a phylogenomic tree (best-fit model distribution JTT, +G +I +I+G and 80% consensus threshold) using PhyML3 (62) (see Additional file 3, Methods). "<br>Lines 362-364<br><br>Line 387, RaxML<br><br>Thank you, we changed RaxML to RAxML.<br>Line 391<br><br>Line 442, "independently" should be "independent"<br><br>Thank you, we modified it.<br>Line 453<br><br>Line 450, no parameters are provided for RAxML analysis.<br><br>Thank you, we included the parameters used in the analysis<br><br>" The phylogenetic tree was constructed using a Maximum Likelihood method with RAxML (-p 12345 -m PROTCATLG)."<br>Lines 449-450<br><br>Line 707, genes<br><br>We apologize for this mistake.<br>Line 721
**Additional Information:**	
**Question**	**Response**
Are you submitting this manuscript to a special series or article collection?	No
**Experimental design and statistics**	Yes

Powered by Editorial Manager® and ProduXion Manager® from Aries Systems Corporation

Full details of the experimental design and statistical methods used should be given in the Methods section, as detailed in our Minimum Standards Reporting Checklist. Information essential to interpreting the data presented should be made available in the figure legends.<br><br>Have you included all the information requested in your manuscript?	
**Resources**<br><br>A description of all resources used, including antibodies, cell lines, animals and software tools, with enough information to allow them to be uniquely identified, should be included in the Methods section. Authors are strongly encouraged to cite Research Resource Identifiers (RRIDs) for antibodies, model organisms and tools, where possible.<br><br>Have you included the information requested as detailed in our Minimum Standards Reporting Checklist?	Yes
**Availability of data and materials**<br><br>All datasets and code on which the conclusions of the paper rely must be either included in your submission or deposited in publicly available repositories (where available and ethically appropriate), referencing such data using a unique identifier in the references and in the "Availability of Data and Materials" section of your manuscript.<br><br>Have you have met the above requirement as detailed in our Minimum Standards Reporting Checklist?	Yes

Manuscript

Click here to access/download;Manuscript;Bats_Manuscript_2020for_producti

Genomic consequences of dietary diversification and parallel evolution due to nectarivory in leaf-nosed bats.

Yocelyn T. Gutiérrez-Guerrero[1], Enrique Ibarra-Laclette[2], Carlos Martínez del Río[3], Josué Barrera-Redondo[1], Eria A. Rebollar[4], Jorge Ortega[5], Livia León-Paniagua[6], Araxi Urrutia[7], Erika Aguirre-Planter[1] and Luis E. Eguiarte*[1]

[1]Departamento de Ecología Evolutiva, Instituto de Ecología, Universidad Nacional Autónoma de México (UNAM), 04510, Mexico City, Mexico.

[2]Red de Estudios Moleculares Avanzados, Instituto de Ecología AC, 91070, Xalapa, Veracruz, Mexico.

[3]Department of Zoology and Physiology, University of Wyoming, 82071, Wyoming, USA.

[4]Centro de Ciencias Genómicas, Universidad Nacional Autónoma de México, Morelos, Mexico.

[5]Departamento de Zoología, Laboratorio de Bioconservación y Manejo, Posgrado en Ciencias Quimicobiológicas, Instituto Politécnico Nacional-ENCB, 11340, Mexico City, Mexico.

[6]Facultad de Ciencias, Universidad Nacional Autónoma de México, 04510, Mexico City, Mexico

[7]Departamento de Ecología Funcional, Instituto de Ecología, Universidad Nacional Autónoma de México (UNAM), 04510, Mexico City, Mexico.

e-mail addresses: ygutierrez@ecologia.unam.mx; enrique.ibarra@inecol.mx; CmDelRio@uwyo.edu; josue_barrera@comunidad.unam.mx; rebollar@ccg.unam.mx; jortegare@ipn.mx; llp@ciencias.unam.mx; a.urrutia@bath.ac.uk; eaguirre@ecologia.unam.mx; fruns@unam.mx

Corresponding author*: fruns@unam.mx

Abstract

Background

The New World Leaf-Nosed bats (Phyllostomids) exhibit a diverse spectrum of feeding habits and innovations in their nutrient acquisition and foraging mechanisms. However, the genomic signatures associated with their distinct diets are unknown.

Results

We conducted a genomic comparative analysis to study the evolutionary dynamics related to dietary diversification and specialization. We sequenced, assembled and annotated the genomes of five Phyllostomid species: one insect-feeder (*Macrotus waterhousii*), one fruit-feeder (*Artibeus jamaicensis*), and three nectar-feeders from the Glossophaginae subfamily (*Leptonycteris yerbabuenae*, *Leptonycteris nivalis* and *Musonycteris harrisoni*), also including the previously sequenced vampire *Desmodus rotundus*. Our phylogenomic analysis based on 22,388 gene families displayed differences in expansion and contraction events across the Phyllostomid lineages. Independently of diet, genes relevant for feeding strategies and food intake experienced multiple expansions and signatures of positive selection. We also found adaptation signatures associated with specialized diets: the vampire exhibited traits associated with a blood diet (i.e., coagulation mechanisms), whereas the nectarivore clade shares a group of positively selected genes involved in sugar, lipid, and iron metabolism. Interestingly, in fruit-nectar feeding Phyllostomid and Pteropodids bats, we detected positive selection in two genes: *AACS* and *ALKBH7,* which are crucial in sugar and fat metabolism. Moreover, in these two proteins we found parallel amino-acid substitutions in conserved positions exclusive to the tribe Glossophagini and to Pteropodids.

Conclusions

Our findings illuminate the genomic and molecular shifts associated with the evolution of nectarivory and shed light on how nectar-feeding bats can avoid the adverse effects of diets with high glucose content.

Keywords

Adaptation, Comparative genomics, Diet, Parallel evolution, Phyllostomid, Specialization

Background

Evolutionary shifts related to changes in feeding habits are considered one of the most important events in animal evolution (1). Diet changes open new ecological and physiological opportunities (1, 2). These shifts often involve changes in feeding behavior, dramatic innovations in the mechanism by which nutrients are assimilated and metabolized, and sometimes drastic morphological modifications (3). Evolutionary diet shifts are sometimes accompanied by species diversification and adaptive functional trait radiation (4).

The New World leaf-nosed bats (family Phyllostomidae) are one of the most species-rich mammalian taxa, with 216 species in 60 genera (5, 6). Leaf-nosed bats evolved from an insect-feeding common ancestor and now display a large and diverse spectrum of feeding habits that include insectivory, carnivory, frugivory, blood-feeding, nectar-pollen feeding and omnivory (5-7). Moreover, dietary specializations and species diversification seem to be correlated in these bats (7,6).

Although most extant Phyllostomids are insectivorous or omnivorous (6, 7), two lineages have extreme dietary specialization: blood-feeding within the subfamily Desmodotinae (including *Desmodus*, *Diphylla* and *Diademus*) and the nectar-pollen feeding species within the subfamily Glossophaginae (including *Leptonycteris*, *Glossophaga*, *Choeronycteris* and *Musonycteris*), that feed primarily on nectar and pollen (5, 6). Among these nectar-pollen feeding species, *Leptonycteris yerbabuenae* (Lesser long-nosed bat) is notable due to its tight co-evolutionary

interactions with plants and seeming specialization to nectarivory/pollinivory (8-10). The blood-feeder *Desmodus rotundus,* and the two other species in the Desmodotinae subfamily have a feeding mode unique among mammals (11). Data on the genome and microbiome of *D. rotundus* have revealed remarkable adaptive changes genes associated with blood diet (12).

Many studies have demonstrated evidence of evolutionary novelties associated with feeding diversification in leaf-nosed bats (6, 13, 14, 15). These include morphological traits involved in nectar extraction (10), and physiological characteristics related to the processing of a diet high in sugars (13, 14). However, the genomic signatures associated with dietary diversification and specialization during the evolution of Phyllostomid bats from an insect-feeder common ancestor remains largely unknown.

We investigated the genomic and evolutionary dynamics associated with the dietary diversification and nectar-pollen feeding specialization of Phyllostomid bats. We sequenced and assembled the whole genomes of five Phyllostomid bat species, including ecologically and economically important species. We sequenced the genomes of three nectar-pollen feeders *Leptonycteris yerbabuenae*, *Leptonycteris nivalis* and *Musonycteris harrisonii*; the fruit-feeder *Artibeus jamaicensis*; and the insect-feeder *Macrotus waterhousii* (Fig. 1). For comparative purposes our analyses incorporated genomic data of the vampire *Desmodus rotundus* (12) and other mammals.

Our research was guided by three sets of predictions. First, we predicted that the dietary diversification from insectivory, which is the ancestral condition in the group, to derived diets would be accompanied by evolutionary changes in relevant genes involved in food uptake and the metabolic pathways associated with the processing of assimilated nutrients. Second, we predicted that the dietary specializations observed in the subfamilies Desmodotidae and Glossophaginae would be correlated with evidence of selection in genes that facilitate the assimilation and metabolism of components of blood and nectar, respectively. More specifically, we expected the nectar-pollen feeder linage to show adaptive signals in genes involved in sugar assimilation and metabolism. Our third prediction was that we should detect convergent evolution

between the New World fruit and nectar feeders and the Old World fruit-bats in genes important for carbohydrate metabolism.

We adopted a hierarchical approach: we examined our predictions in deep nodes of the phylogeny, then we identified the nodes that represent dietary transitions and investigated the changes that accompanied these transitions. We conducted a genomic comparative approach and performed a phylogenomic reconstruction to identify expansions/contractions of gene families across the Phyllostomids lineage. We also evaluated orthologous protein sequences that have been targets of selection and their relation to dietary diversification and specialization. Finally, in order to identify convergent evolutionary signals associated with the diet, we carried out a comparison between the genomes of the nectar-pollen feeders Phyllostomid bats and the Old World fruit-feeder bats (family Pteropodidae), analyzing radical amino-acid substitutions in conserved positions.

Data Description

We sequenced the genome of one adult male Lesser long-nosed bat (*Leptonycteris yerbabuenae*) by a whole high-throughput shotgun strategy and obtained a high quality *de novo* assembly (104 x) (Table 1; see Additional file 1, Table S1, S11). Additionally, we sequenced with medium coverage (~24 – 56 x) the genomes of four Phyllostomid bats: *M. waterhousii* (insect-feeder), *A. jamaicensis* (fruit-feeder), and the nectar-pollen feeders: *M. harrisoni* and *L. nivalis*. (Fig. 1; see Additional file 1, Table S1).

The genomic landscape of New World Leaf-nosed bats

The size of *L. yerbabuenae'*s genome was similar to those reported for other bats (2.05 Gb), with an N50 scaffold length of 14, 735,151 bp, and L50 of 38 scaffolds (Table 1). Evaluation of the genome assembly for completeness based on BUSCO identified 94% of complete and 2.5% of fragmented genes from the mammalian database (Mammalia *odb9*). The genome contained 24,074 inferred coding sequences from an *ab initio* prediction, the transcript evidence and

homology evidence obtained from a set of proteins of several mammalian species (Table 1; see Additional file 1, Table S2). Approximately 26% of the genome assembly was composed of repetitive elements (547 Mbp length) (see Additional file 1, Table S3).

We constructed a reference guide genome assembly based on *L. yerbabuenae* for the other four Phyllostomid bats, where we annotated from 18,000 to 24,471 coding sequences and proteins for each Phylllostomid (Table 2; see Additional file 1, Table S4-S5; Additional file 2, Fig. S1).

Analyses

Gene family evolution reflects distinct dietary needs

To understand genomic evolution and to trace changes associated with dietary diversification and specialization, we reconstructed a phylogenomic tree using 132 single-copy orthologous genes (61,331 amino acids sites), which was calibrated using two fossil dates (16-18). Based on the phylogenomic tree, we analyzed the dynamics (expansion and contractions) for 22,388 gene families across the Phyllostomid bat genomes (Fig. 2 and Table 3).

For all the Phyllostomid bats, the significant gene family enrichment functions were related to the cellular repair process and genetic make-up for protein synthesis. Furthermore, across the Phyllostomid bats many gene families exhibited changes with feeding habits, for example, the Phyllostomid node had a contraction related to the lipid metabolism. The blood-feeder lineage had a significant gain on gene families involved in the regulation of appetite and process for nitrogen acquisition, but also this lineage showed many contraction events involved in calcium metabolism (Table 3). The fruit and nectar feeding bats exhibited many expansion events in iron metabolism regulation pathways (Table 3).

Rapidly evolving genes across the whole genome

For all Phyllostomid bats, we identified 42 genes with robust signals of positive selection (FDR $p < 0.05$). According with the enrichment analysis, most of the adaptive genes are related to

immune response, DNA repair, inflammatory response, RNA catalytic process and genes that mediate muscle function (such as *Myoblast and PAMR1*) (Fig. 3; see Additional file 1, Table S6-TableS8) (19).

Ecological and feeding behavior adaptations across the Phyllostomid lineages

To understand shifts associated with dietary diversification, we analyzed genes under positive selection involved in the mechanisms of carbohydrate digestion and lipid metabolism in each Phyllostomid species (Fig. 3).

In *M. waterhousii*, an insect-feeder, we found evidence of positive selection in *Chitinase*, which codes for proteins in the degradation of insect exoskeleton (20) (Fig. 3) Interestingly, for *M. waterhousii*, the *Trehalase* is a partial gene that exhibited signals of positive selection, but for the rest of the Phyllostomid species, *Trehalase* is a pseudogene. This finding is relevant, because trehalose is the principal sugar in insect's blood.

The vampire's genome revealed a complex set of genes crucial for maintaining a blood feeding diet under positive selection, including *THBD* (hematopoietic cell pathway) and *A2M* (complement and coagulation cascade pathway) (21). Only in the vampire we found under positive selection genes involved in feeding and lipid – cholesterol metabolism such as *MGAT2*, *PLAS2G16,* and *GFOD1* (22, 23) (Fig, 3). Interestingly, the vampire was the only genome where the *Trehalase* gene was completely missing.

In the fruit bat *A. jamaicensis* most of the enzymes analyzed involved in lipid and carbohydrates metabolic pathways showed positive selection pressures (Fig. 3; see Additional file 1, Table S8). The importance of these enzymes for this fruit bat might reflect the diversity of its diet, as *A. jamaicensis* has been documented to eat, besides insects and fruits, seeds and leaves (24).

Every nectar-pollen feeder bat species (*M. harrisoni*, *L. nivalis* and *L. yerbabuenae*) showed positive selection signatures for genes involved in insulin secretion (*UCN 3*) (25), calcium and iron storage (*CALP2, CD248* and *FTL*) (26-27), bone morphogenetic regulation (*Fslt1*) (28), and

in *IAP*, the gene coding for the mucosa defense factor involved in proper gut homeostasis (Fig. 3) (29). Interestingly, we found adaptive signatures for genes crucial for carbohydrates and lipid metabolic pathways, such as pancreatic secretion, glycolysis / gluconeogenesis, glycogen, glycerophospholipid, citrate acid metabolism and ketone metabolism (Fig. 4; see Additional file 1, Table S8) (30-32).

Finally, for the nectar-pollen and fruit bat species, we detected strong selective pressures in four enzymes, *AACS* (which appears to participate in the regulation of lipid metabolism) (32), *ALKBH7* (which codes for a protein that appears to be involved in the regulation of body mass and fat content) (33), *FABP1* (regulates fatty acid trafficking and prevents lipotoxicity) (34) and *AMPK* (major regulator of cellular energy homeostasis) (Fig. 3 and 4) (35).

Adaptation and convergent signatures in fruit and nectar-pollen feeding bats

The sugar and lipid metabolism genes detected under positive selection for the fruit and nectar-pollen feeding Phyllostomid bats, were also analyzed in the genomes of three Old World bat species available in databases, including *Pteropus alecto*, *Pteropus vampyrus* and *Rousettus aegyptiacus* (Family Pteropodidae). We found evidence of positive selection in *AACS* in *P. alecto*, *P. vampyrus* and *R. aegyptiacus*. In *R. aegyptiacus*, we also found evidence of positive selection in *ALKBH7* (*see* Additional file 1: Table S8).

We analyzed 1,918 orthologous sequences and reconstructed the ancestral sequences states, in order to identify some genes with unique and exclusively parallel substitutions for the Phyllostomid fruit bat, the Glossophagini and Pteropodid lineages, in a conserved position for the rest of the bats and mammal species (Fig. 5b). We found three genes with parallel signatures in a specific amino-acid position. Most of these parallel changes presumably led to changes in the physicochemical properties of the expressed protein (Fig. 5a). In *AACS,* we identified six radical amino-acid substitutions along the sequence, from an ancestral glycine (non-polar) to a derived arginine (+ charged), alanine (non-polar) to threonine (polar), glycine to serine (polar), alanine to proline (non-polar), serine to proline and serine to leucine (non-polar) (Fig. 5a). In *ALKBH7,* we

found a parallel amino-acid substitution from glutamic acid (- charged) to lysine (+ charged) and arginine to glutamine (polar). The latter change was found in the Glossophagines and *R. aegyptiacus* (Fig. 5b). Finally, for the gene *UNC-45 B,* which codes for a protein involved in muscle cell development, we identified a substitution from leucine to arginine (Fig. 5a) (36). Moreover, we calculated the posterior probabilities for each amino acid reconstruction state for each node along the three. For all nodes in the Glossophagini and Pteropodids clades, the probabilities of each amino acid derived state were > 80% (Fig. 5c; see Additional file 1, Table S9).

Finally, in order to evaluate if the radical amino acid substitutions had affected the protein structure of *ACCS,* we modeled its 3D- protein structure using Hidden Markov Models, for the nectar-feeders: *M.* harrisoni, *L. nivalis* and *L. yerbabuenae*; the fruit bat *P. alecto*; and *D. rotundus* and *M. waterhousii* (Fig. 6a; see Additional file 1, Table S10). *ACCS* protein structure is composed by 662 amino acids, two domains and 96 atoms of beta strand, 181 atoms of alpha helix and 4,391 loop atoms (Fig. 6a). Moreover, we performed a multi-comparison of the 3D structure for all the species mentioned above (see Additional file 1: Table S11). We found high similarity in the 3D protein structure for all bats (RSMD values from 0 to 0.003). However, we identified three residues of alpha-helix shared only for the Glossophaginae clade, and a beta strand shared only between *M. harrisoni* and *P. alecto* (Fig. 6b; see Additional file 1, Table S11).

Discussion

Our study provides unprecedented knowledge on the genomic signatures behind the dietary diversification and specialization in Phyllostomid bats. Surprisingly, and contrary to our first prediction, we found that many of the genomic characteristics of the ancestral Phyllostomid diet, remained functional in all lineages of the family. For example, the C*hitinase* gene was functionally conserved in most genomes (with the exception of the vampire), highlighting the relevance of digestion and nutrient uptake from insects in all lineages, including those that mainly feed on fruit, nectar-pollen and even in some cases, those that feed only on blood (Fig. 3 and 4) (9, 10, 20).

However, as previously studies suggest, we found that *Trehalase* (involved in the digestion of trehalose from insect blood, comprising approximately to 7% of their dry mass) is missing or a pseudogene for all the blood, fruit, and nectar-feeding bats, which may be a result of dietary diversification in the family (37).

Our second prediction was supported: we found unique genomic specializations in bats with obligate and restrictive diets, such as the vampire and the nectar-pollen feeders (Fig. 3 and 4, Table 3). The vampire's genome has unique characteristics associated with the ability to consume blood, including genes that play a crucial role in the down-regulation of fibrinolysis and those that control the production of blood cells (Fig. 4) (12, 21, 38). On the other hand, we also detected strong positive selection in genes crucial for carbohydrate oxidation, ATP production, and in genes involved in ketone metabolism in the three Glossopaghini bats. These are associated with the extreme energetic feat of feeding on the wing (14, 30, 39) (Fig. 4). Our analyses also highlight the importance of genes involved in iron storage for animals that feed on iron deficient sources. These results might be related to the avoidance of metabolic disorders such as anemia (Fig. 4) (28). The results also help to explain how bats that feed on nectar-pollen can avoid the potentially adverse effects of their peculiar diet. In humans, loss-of-function due to mutations in some of these genes are associated with nutrient malabsorption and metabolism disorders including diabetes, hyperglycemia and obesity (30, 39).

In support of our third prediction, we identified signatures of molecular parallel evolution shared by fruit-feeding Pteropodids and nectar-pollen feeding Glossophanigi bats (Fig. 5ab). The ancestral sequence reconstruction provided us with insights into the mechanisms of molecular adaptation and functional divergence. Signals of parallel evolution and adaptative selection for the proteins *ACCS* and *ALKBH7,* shed light on the importance of the storage of fatty fuels necessary to meet the energy demands of an expensive mode of foraging and pollinator ecology of these specialist bats. Protein function is more likely to be affected if genes show many radical substitutions in conserved positions, and signal of positive selection. In spite of the evolutionary changes detected for *ACCS* in the Glossophagini species, their tertiary protein structure exhibited

high similarity when we compared it with other bats (see Additional file 1, Table S11). However, we identified exclusive differences in alpha-helix and beta-strand regions (Fig. 6b), that may be important in the protein function for the nectar-pollen feeders and the Old World fruit bat (Fig. 6). We consider that it is very probable that *ACCS* is up or down regulated. Future studies must evaluate the expression levels for this gene, and its regulation, including replicating and analyzing more tissues, such as the gut and liver (40, 41).

Surprisingly, the protein *UNC-45 B,* exhibited the same amino acid substitution between Glossophagini, Pteropodids and the dolphin. Lee *et al.,* (2018) have identified genes involved in muscle skeletal function and movement, with parallel substitutions shared between bats and marine mammals (such as dolphin, whale and Baiji). We suggest that the protein *UNC-45 B* may be implicated in an efficient mobility and superfast muscle physiology for this species (42).

On the other hand, the molecular traits that we infer as the result of parallel evolution, were not found in the fruit-eating bat *A. jamaicensis*. We hypothesize that this species should be considered more omnivorous than strictly frugivorous (24, 41). Omnivory-frugivory might have been an important step in the transition to a more restricted fruit diet and to a nectar-pollen diet (Fig. 4 and 5) (5-8). To explore this hypothesis further, it will be necessary to expand our sample of genomes to include more Phyllostomid species that have more exclusively frugivorous fruits than *A. jamaicensis*.

Our findings suggest that parallel evolution due to nectar-feeding dietary specialization is likely a consequence of high metabolic demands required for foraging on flowers and fruits. These results are notable, given that the Pteropodidae and Glossophaginae lineages are separated by over 60 Mya (8) (see Additional file 2, Fig. S2) (17). Moreover, our results shed light on the evolutionary mechanisms and genomic shifts that take place in the transition to novel feeding habits. They also illuminate on the genomic changes that take place when animals adopt a diet dominated by sugar consumption, and with low levels of lipids and proteins. The generality of inferences can be tested in other nectar specialized vertebrate taxa, such as hummingbirds (40).

Finally, we found differences in the evolution of gene families and genes that are not necessarily or only related to diet among Phyllostomid lineages (Fig. 2 and Table 3). These differences are likely associated to other lineage-specific aspects in physiology, ecology (niche resources, interactions, immune system, etc.) and microbiomes (5, 6, 12, 43). As an example, positive selection in *IAP* enzyme was only detected in the nectar feeding bats, which supports a strong relationship between dietary specialization and the bacterial communities, that are involved in providing vitamins and aiding digestive processes (Fig. 4) (12). Future analyses should address the relationship between host diet-intestinal bacterial community, and the evolution of microbiomes across dietary diversification and specialization.

Methods

Animal sampling and genome sequencing

An adult male *Leptonycteris yerbabuenae* (NCBI txid: 700936) was collected and processed on site at the cave “El Salitre” in Morelos state, Mexico (18°44'28'' N, 99°10'46'' W). All procedures were carried out in accordance with Federal Mexican Procedures (Guidelines of Secretaría de Medio Ambiente y Recursos Naturales, SEMARNAT), permit SGPA/DGVS/07161/15. The Zoology Museum “Alfonso L. Herrera” (Facultad de Ciencias, UNAM), donated the tissue samples from four leaf-nosed bats: *Macrotus waterhousii* (NCBI txid: 124750), *Artibeus jamaicensis* (NCBI txid: 9417), *Leptonycteris nivalis* (NCBI txid: 59456) and *Musonycteris harrisoni* (NCBI txid: 148053) (Fig. 1; see Additional file 1, Table S1).

For all leaf-nosed bats species, we isolated their DNA using Phenol-Chloroform protocol and DNA Blood and Tissue Kit (Qiagen). We used the Illumina HiSeq 4000 150 PE platform to sequence the genomes (see Additional file 1, Table S1). We paid special attention to *L. yerbabuenae,* in order to use it as a reference to help in the assembly construction of the other genomes. In this species, we performed high whole genome sequencing (the DNA sample was sequenced on two lanes). Additionally, we used the fresh samples collected from *L. yerbabuenae* to obtain transcriptional evidence for the genome annotation, we extracted the RNA-Seq from five

tissues: brain, pancreas, kidney, lung, and liver (reserved in a buffer storage of RNA stabilization) using the RNeasy Mini Kit (Qiagen). All tissues with RIN values >= 8 were sequenced on Illumina HiSeq 4000 150 PE platform.

Leptonycteris yerbabuenae genome assembly

De novo genome assembly

The genome assembly was constructed *de novo* with Platanus (Platanus, RRID:SCR_015531) v. 2.4.3 (44), using a heterozygous value = 0.04 (-u 0.04) and an initial kmer=32. To optimize and extend the genome assembly, we performed a scaffolding with MeDuSa software (45, 46). Finally, we used Pilon (Pilon, RRID:SCR_014731) for correcting bases and polish the genome assembly (47).

We evaluated the genome assembly metrics (total length, number of scaffolds, number of contigs, L50, N50, and others). Moreover, with BUSCO (BUSCO, RRID:SCR_015008) v3 and the Mammalia odb9 database (48) we evaluated the measure for quantitative assessment of the genes content into the genome assembly.

Gene prediction

We performed TEdenovo from the REPET package (49) to predict, identify and annotate the transposable elements (TE) using the repetitive elements database Repbase (50). We masked the TEs across the genome using RepeatMasker (RepeatMasker, RRID:SCR_012954) v4.0.7 (see Additional file 1, Table S3) (51).

We also cleaned, filtered and assembled the RNA-Seq data of five tissues (brain, pancreas, kidney, liver, and lung) for the same individual with Trinity (Trinity, RRID:SCR_013048) v4.4.7 (52). Based on the transcriptome assembly, we identified open-reading frames, coding sequences and their corresponding proteins (53).

We generated an *ab initio* gene prediction using Augustus (Augustus: Gene Prediction, RRID:SCR_008417) v2.5.5 (54). To train Augustus, we used the gene structures of *E. fuscus* bat, and the RNA-Seq evidence (transcripts annotated from *L. yerbabuenae*). We performed a

functional annotation by blastp using the UniProtKb SwissProt dababase and InterProScan (55, 56).

NW Leaf-Nosed bats reference genome construction (assembly and annotation)

We used the *L. yerbabuenae* assembly as a reference genome to construct the assembly of *M. waterhousii*, *A. jamaicensis, M. harrisoni* and *L. nivalis*.

All the raw data were filtered and cleaned (using a PHRED score >= 30) (see Additional file 1, Table S1). We followed the GATK (GATK , RRID:SCR_001876) v2.07 pipeline to identify Single Nucleotide Variants (SNVs) (57). First, to find all the SNVs along the genomic information from each Phyllostomid, based on the *L. yerbabuenae* genome assembly, all the high-quality genomic reads of each Phyllosomid were mapped to *L. yerbabuenae* genome assembly with BWA mem (BWA, RRID:SCR_010910) (58). Second, we used the GATK and Picard (Picard, RRID:SCR_006525) tools to recalibrate the genome mapping and identified the SNVs for each Phyllostomid (SortSam, MarkDuplicates, AddOrReplaceReadGroups, BuildBamIndex and CreateSequenceDictionary, RealignerTargetCreator, IndelRealigner, HaplotypeCaller, VariantFiltration, SelectVariants, BaseRecalibrator, AnalyzeCovariates, PrintReads, and VariantFiltration) (see Additional file 2, Fig. S3; Additional file 3, Methods) (56, 59). We constructed the consensus sequence based on the SNVs identified (with SAMtools, BCFtools, vcfutils.pl and SeqtK) (60-61). We evaluated for each consensus genome constructed, the assembly metrics and the integrity and gene contend with BUSCO.

We predicted, identified and masked the TEs for each consensus genome using RepeatMasker v4.0.7 tool (50, 51). We performed the gene prediction with Augustus. The genomes were annotated with blastp and InterProScan (InterProScan, RRID:SCR_005829) (see Additional file 3, Methods).

Phylogenomic and gene family analysis

A total of 132 single-copy orthologous genes (61,331 amino acids sites), across 18 mammals were concatenated to reconstruct a phylogenomic tree (best-fit model distribution JTT, +G +I +I+G and 80% consensus threshold) using PhyML3 (62) (see Additional file 3, Methods). We estimated molecular substitution rates with CODEML from the Phylogenetic Analysis by Maximum Likelihood, PAML (PAML, RRID:SCR_014932) package (63). Based on a Bayesian phylogenetic method, with MCMCtree tool, we estimated the species divergence times using fossil records from *Icaronycteris* (~50 Mya) and *Tachypteron* taxon with molecular ages of 64 Mya (16-18).

We used CAFE (64) to analyse the statistical changes in the gene family sizes using a birth and death estimator (λ and μ). Based on the distribution of observed family sizes, we calculated the *p-values* for gene family expansions and contractions. We carried out the gene family annotation with shell and PERL scripts (see Additional file 3, Methods).

dN/dS analysis using a branch-site model

Orthologous single copy genes and filtering

We used the proteins annotated of *M. waterhousii*, *A. jamaicensis*, *M. harrisoni*, *L. nivalis* and *L. yerbabuenae* to create a database, incorporating the complete set of protein's of all Laurasiatheria species available in the ENSEMBL database (35 species), and the protein information of all bat species available in NCBI database (7 species) (see Additional file 1, Table S12). Based on a multi-species genome comparison with this database, we inferred the orthologous single copy genes using DIAMOND and Proteinortho programs (65, 66). We extracted all the single copy genes (scg) shared for each Phyllostomid bat, obtaining more than 9,637 scg clusters. We checked and removed all potential paralogous sequences, and ambiguous amino acids (letter X). Each single copy gene cluster was composed from 8 to maximum 20 sequences.

Each cluster was aligned with MAFFT (MAFFT, RRID:SCR_011811) aligner tool (67), we retained alignment sequences where the length is within 80 to 120% relative to the human and

mouse sequences, and poorly aligned regions were removed by a visual inspection. We used the alignments and their corresponding coding sequences to perform a robust conversion of protein multi alignment into their corresponding codon alignments with PAL2NAL (see Additional file 3, Methods) (68). We reconstructed the phylogenetic tree for each cluster with RAxML (parameters -m GTRGAMMA -p 12345) (69).

dN/dS Test

We used the codon multi-alignment files and their corresponding phylogenetic tree to calculate synonymous sites and nonsynonymous sites (dN/dS) rates, using two bioinformatic tools: CODEML and HYPHY (HYPHY, RRID:SCR_016162) (63, 70).

With CODEML, we used a branch-site model, specifying the *foreground* branch (the species of our interest) and incorporating a null model [that assumes that background and the foreground branches share the same ratio (ω)]. We designated each Phyllostomid species as the foreground branch of our interest, and we performed independently the CODEML analysis. For assigning significance, we constructed the Likelihood Ratio Test (LRT) for each Phyllostomid result, using the likelihood values from the null and test model, and calculated the *p-value* =< 0.05 under a chi-square distribution. We also performed a *p-value* adjust, using the False Discovery Rate (FDR) correction, based on the likelihood ratio. Additionally, we considered under positive selection all those sequence sites with a posterior probability > 95% (by Bayes Empirical Bayes method).

With Hyphy, we used aBSREL (adaptive Branch-Site Random Effects Likelihood) (71). aBSREL infers the optimal number of ω to test if positive selection has occurred on a proportion of branches. The LRT is performed at each branch and compares the test model and null model. We inferred the optimal ω for all the branches for each single copy gene cluster (including bats and non-bats species).

We retained and classified those adaptative genes that were identified in both programs (CODEML and HYPHY), with a *p-value* =< 0.05.

GO enrichment

We performed an enrichment analysis, using the weight01 algorithm with topGO v2.26 package

from Bioconductor project in R (72-73). We obtained the statistical significance for the GO enrichment terms by performing the Fisher's exact test (*p-value* =< 0.01).

Radical amino-acid substitution in conserved positions

Ancestral sequence reconstruction

Based on the previously inferred orthologous genes, we extracted all single copy genes shared by the Glossophagini (Glsp): *M. harrisoni*, *L. nivalis*, *L. yerbabuenae;* and the Pteropodids (Ptrp): *P. alecto*, *P. vampyrus* and *R. aegyptiacus.* Each single copy gene cluster was composed of between 12 and a maximum 30 sequences.

We obtained 1,918 clusters of orthologous sequences (including at least one Glsp and one Ptrp). Each cluster was aligned using PRANK (74) and we constructed their corresponding phylogenetic tree with RAxML (RAxML, RRID:SCR_006086) (parameter -m PROTCATLG) (69). We checked all alignments for gaps and premature stop codons. We performed an ancestral sequence reconstruction using the protein alignments and phylogenetic trees. To dismiss incorrectly inferred residues and only retain the accurate ones for the reconstructed ancestral sequence, we used two different programs CODEML, that assumes a Markov process model and calculates a Bayesian empirical likelihood for each character at each sequence position (63), and FastML, that assumes a continuous time Markov process model and provides the posterior probabilities for each character at each sequence position (75). Both programs provide the ancestral sequence and the posterior probabilities distribution. For CODEML, we fixed the parameters: model =2, fix_alpha = 0, alpha = 0.5 and RateAncestor = 1.

Based on the ancestral sequence information, we identified pairs of branches for Glsp and Ptrp species that exhibited a parallel amino acid substitution. We also checked the ancestral state at each node for these substitutions. We classified each parallel substitution as a radical amino acid substitution in a conserved position, assuming two criteria: 1) parallel substitution is exclusive in the branches of Glsp, Ptrp and their corresponding nodes; and 2) different physicochemical properties between the most frequent amino acid state and the derived (parallel

substitution).

We checked the Bayesian empirical likelihood at each ancestral state for all parallel substitution position. We retained only those parallel substitution with a posterior probability > 85%.

Finally, we constructed a phylogeny using the information of those genes with parallel evolution. We concatenated the amino acid sequence for these three genes. We aligned the sequences with MAFFT tool. We used ProtTest3 to select the best-fit model of protein evolution (76). The phylogenetic tree was constructed using a Maximum Likelihood method with RAxML (-p 12345 -m PROTCATLG).

Drivers of parallel evolution

In independent branches, mutation and selection can have equal impacts on patterns of parallel substitutions. For those proteins that exhibited parallel evolution, we also evaluated four variables at DNA and protein level: length, GC percent, rates at synonymous sites and nonsynonymous sites (dN/dS) and isoelectric point (77-78).

Protein modeling

To explore the effects of positive selection and the radical amino acid substitutions, we modeled the second and tertiary structure of the protein *Acetoacetyl CoA Synthetase* (ACCS) for *M. waterhousii*, *D. rotundus*, *M. harrisoni*, *L. nivalis*, *L. yerbabuenae* and *P. alecto*. We used Phyred 2 software (79), which compares the profile of the protein of our interest, with a protein database, using Hidden Markov Models and predicting secondary structure for each residue. To identify differences in the protein structure, we compared the secondary and tertiary structure between the nectar- fruit bats and *M. waterhousii* and *D. rotundus*, using the software PyMOL (80-81). To calculate the RSMD score, we aligned the PDB protein model between pairs.

Availability of data and materials

Supporting data including genome assemblies, genome and protein annotation, TE prediction and annotation, multifasta gene families, phylogenomic alignment, ancestral sequence reconstruction, and tertiary protein modelling (PDB) files, are available via the *GigaScience* database GigaDB (82).

The whole genome and RNA-Seq sequence information analysed during the current study are available in the National Center for Biotechnology Information (NCBI): whole genome assembly for *L. yerbabuenae* within BioProject: PRJNA542899 and SRA: SRR9076597. The RNA-Seq data is available within Bioproject: PRJNA543325. Raw genome data of *M. waterhousii, A. jamaicensis*, *M. harrisonii* and *L. nivalis* are available in the SRA: SRR908760, SRR9087866, SRR9089318 and SRR9089325.

Declarations

Ethics approval and consent to participate

The use of animals in this study was performed in accordance with the Federal Mexican Procedures: Guidelines of Secretaría de Medio Ambiente y Recursos Naturales, SEMARNAT, with permit SGPA/DGVS/07161/15.

Consent for publication

Not applicable.

Competing interests

The authors declare that they have no competing interests.

Funding

Y.T.GG is supported by a doctoral scholarship from the Comisión Nacional de Ciencia y Tecnología (CONACyT, Beca Mixta grant no. 291250). The study was supported by a grant from Fronteras de la Ciencia, “Genómica de la Diversidad de Vertebrados 1: *Leptonycteris* y la

evolución de la nectarivoría en murciélagos y aves” (CONACyT, project no. 177) to L.E.E.

Author’s Contributions

YT.G.G and L.E.E designed and performed research. YT.G.G and L.E.E wrote the paper with contributions from C.M.R, E.A.R, A.U, J.O, E.A.P and E.I.L helped with computational resources and bioinformatic analyses. J.B.R helped with bioinformatic analyses. L.L.P donated the samples. All authors revised and edited the manuscript.

Acknowledgments

This manuscript constitutes part of the doctoral project of the first author, who thanks the Posgrado en Ciencias Biomédicas (Universidad Nacional Autónoma de México, UNAM) and acknowledges the doctoral scholarship supported by Comisión Nacional de Ciencia y Tecnología (CONACyT, grant no. 580116; Beca Mixta grant no. 291250), and the grant Fronteras de la Ciencia (CONACyT, project no. 177). The authors wish to acknowledge the Instituto de Ecología (UNAM), Comisión Nacional para el Conocimiento de la Biodiversidad (CONABIO) and Instituto de Ecología A.C. (INECOL, A.C) for computing resources. Special thanks to MSc. R. Trejo-Salazar, MSc. O, Gaona, Biol. A, Galicia for their assistance during fieldwork in the El Salitre cave. We thank Dra. L. Espinosa Asuar, Dra. V. Souza, S. Barrientos and all the Lab. Evolución Molecular y Experimental (UNAM) for their help in lab work. To MSc. E. Villafán, Ing. Rodrigo García and Mat. E. Campos for their computational assistance. We gratefully acknowledge Dr. S. Ramírez-Barahona and C. Keenan for their constructive comments on the manuscript. We acknowledge MSc. D. Zamora-Mejías, R. Medellín-Legorreta and Biol. Melissa E. Rodríguez for providing us pictures of Phyllostomid bat species. We gratefully acknowledge BioCurator Chris Hunter for his technical support to prepare our GigaDB dataset.

References

1. Hunter JP. Key innovation and ecology of macroevolution. *Trends Ecol Evol.* 19981;3:31–36.

2. Yoder JB, Clancey E, Des Roches S, Eastman JM, Gentry L, et al. Ecological opportunity and the origin of adaptive radiations. *J Evol Biol.* 2010;23:1581–1596.

3. Palm W, Thompson CB. Nutrient acquisition strategies of mammalian cells. *Nature.* 2017;546:234–242.

4. Borstein SR, Fordyce JA, O'Meara BC, Wainwright PC, McGee MD. Reef fish functional traits evolve fastest at trophic extremes. *Nat Ecol Evol.* 2018;3-191-199.

5. Rojas D, Vale A, Ferrero V, Navarro L. When did plants become important to leaf-nosed bats? Diversification of feeding habits in the family Phyllostomidae. *Mol Ecol.* 2011;20:2217-28.

6. Rossoni DM, Assis APA, Giannini NP, Marroig G. Intense natural selection preceded the invasion of new adaptive zones during the radiation of New World leaf-nosed bats. *Sci Rep.* 2017;7:1–11.

7. Yohe LR, Velazco PM, Rojas D, Gerstner BE, Simmons NB, Dávalos LM. Bayesian hierarchical models suggest oldest known plant-visiting bat was omnivorous. *Biol Lett.* 2015;11:20150501.

8. Fleming TH, Geiselman C, Kress WJ. The evolution of bat pollination: a phylogenetic perspective. *Ann Bot.* 2009;104:1017–1043.

9. Cole FR, Wilson DE. *Leptonycteris yerbabuenae*. *Mamm Species.* 2006;797:1–7.

10. Gonzalez-Terrazas TP, Medellin RA, Knörnschild M, Tschapka M. Morphological specialization influences nectar extraction efficiency of sympatric nectar-feeding bats. *J Exp Biol.* 2012;215:3989–3996.

11. Hong W, Zhao H. Vampire bats exhibit evolutionary reduction of bitter taste receptor genes common to other bats. *Proc R Soc B Biol Sci.* 2014;281:20141079.

12. Zepeda Mendoza ML, Xiong Z, Escalera-Zamudio M, Runge AK, Thézé J, et al. Hologenomic adaptations underlying the evolution of sanguivory in the common vampire bat. *Nat Ecol Evol.* 2018;2:659–668.

13. Ayala-Berdon AJ, Schondube JE. A physiological perspective on nectar-feeding adaptation in Phyllostomid bats. *Physiol Biochem Zool.* 2015;84:661541.

14. Schondube JE, Herrera-M, LG, Martínez del Rio C. Diet and the evolution of digestion and renal function in phyllostomid bats. *Zoology (Jena).* 2001;104:59–73.

15. Zhao H, Zhou Y, Pinto CM, Charles-Dominique P, Galindo-González J, et al. Evolution of the Sweet Taste Receptor Gene Tas1r2 in Bats Research article. *Mol Biol Evol.* 2010;27:2642–2650.

16. Simmons NB, Geisler JH. Phylogenetic relationships of *Icaronycteris*, *Archaeonycteris*, *Hassianycteris*, and *Palaeochiropteryx* to extant bat lineages, with comments on the evolution of echolocation and foraging strategies in Microchiroptera. In: Bull Am Museum Nat Hist. 1998. http://hdl.handle.net/2246/1629. Accessed 01 Sep 2019.

17. Teeling EC, Springer MS, Madsen O, Bates P, O'brien SJ, et al. A molecular phylogeny for bats illuminates biogeography and the fossil record. *Science.* 2005;307:580-4.

18. Simmons NB, Seymour KL, Habersetzer J, Gunnell GF. Primitive Early Eocene bat from Wyoming and the evolution of flight and echolocation. *Nature*. 2008;451:818-821.

19. Chen EH. Invasive Podosomes and Myoblast Fusion. *Curr Top Membr.* 2011;68:235-258.

20. Nardelli A, Vecchi M, Mandrioli M, Manicardi GC. The evolutionary history and functional divergence of trehalase (*treh*) genes in insects. *Front Physiol.* 2019:00062.

21. Okamoto T, Tanigami H, Suzuki K, Shimaoka M. Thrombomodulin: A Bifunctional Modulator of Inflammation and Coagulation in Sepsis. *Crit Care Res Pract.* 2012(Dic):1–10.

22. Tsuchida T, Fukuda S, Aoyama H, Taniuchi N, Ishihara T, et al. MGAT2 deficiency ameliorates high-fat diet-induced obesity and insulin resistance by inhibiting intestinal fat absorption in mice. *Lipids Health Dis.* 2012;11:1–10.

23. Xiong S, Tu H, Kollareddy M, Pant V, Li Q, et al. Pla2g16 phospholipase mediates gain-of-function activities of mutant p53. *Proc Natl Acad Sci USA*. 2014,30:11145-11150.

24. Kunz TH, Díaz CA. Folivory in Fruit-eating Bats, with New Evidence from *Artibeus jamaicensis* (Chiroptera: Phyllostomidae). *Biotropica.* 1995;27:106–120.

25. Li C, Chen P, Vaughan J, Lee K-F, Vale W. Urocortin 3 regulates glucose-stimulated insulin secretion and energy homeostasis. *Proc Natl Acad Sci USA.* 2007;104:4206–4211.

26. Teicher B. CD248: A therapeutic target in cancer and fibrotic diseases. *Oncotarget*. 2019,10:993-1009.

27. Drysdale J, Arosio P, Invernizzi R, Cazzola M, Volz A, et al. Mitochondrial ferritin: A new player in iron metabolism. *Blood Cells Mol Dis.* 2002;29:376–383.

28. Geng Y, Dong Y, Yu M, Zhang L, Yan X, et al. Follistatin-like 1 (Fstl1) is a bone morphogenetic protein (BMP) signaling antagonist in controlling mouse lung development. *Prot Natl Acad Sci USA.* 2011,17:7058-7063.

29. Lallès JP. Intestinal alkaline phosphatase: Multiple biological roles in maintenance of intestinal homeostasis and modulation by diet. *Nutr Rev.* 2010;68:323–332.

30. Sylow L, Kleinert M, Richter EA, Jensen TE. Exercise-stimulated glucose uptake - regulation and implications for glycaemic control. *Nat Rev Endocrinol*. 2016;13:133-148.

31. Griffin BA. Lipid metabolism. *Surgery (Oxford).* 2013;31:267-272.

32. Shi L, Tu PB. Acetyl-CoA and the regulation of metabolism: mechanisms and consequences. *Curr Opin Cell Biol.* 2015;33:125-131.

33. Solberg A, Robertson AB, Aronsen JM, Rognmo O, Sjaastad I, et al. Deletion of mouse Alkbh7 lead to obesity. *J Mol Cell Biol.* 2013;5:194-203.

34. Guzmán C, Benet M, Pisonero-Vaquero S, Moya M, García-Mediavilla M, et al. The human liver fatty acid binding protein (FABP1) gene is activated by FOXA1 and PPAra; and repressed by C/EBPa: Implications in FABP1 down-regulation in nonalcoholic fatty liver diseae. *BBA Mol Cell Biol L.*2013,4:803-818.

35. Srivastava R, Pinkosky SL, Filippov S, Hanselman JC, Cramer CT, et al. AMP-activated protein kinase: an emerging drug target to regulate imbalances in lipid and carbohydrate metabolism to treat cardio-metabolic diseases. *J Lipids Res.* 2012,53:2490-2514.

36. Kachur TM, Pilgrim DB. Myosin assembly, maintenance and degradation in muscle: Role of the chaperone UNC-45 in myosin thick filament dynamics. *Int J Mol Sci.* 2008,9:1863-1875.

37. Jiao H, Zhang L, Xie HW, Simmons NB, Lui H, et al. Trehalase gene as a molecular signature of dietary diversification in mammals. *Mol Biol Evol.* 2019;10:2171-2183.

38. Qu J, Ko CW, Tso P, Bhargava A. Apolipoprotein A-IV: A multifunctional protein involved in protection against atherosclerosis and diabetes. *Cells.* 2019,4:319.

39. Jensen J, Rustad PI, Kolnes AJ, Lai YC. The role of skeletal muscle glycogen breakdown for regulation of insulin sensitivity by exercise. *Front Physiol.* 2011;2:1–11.

40. Workman RE, Myrka M, Wong GM, Tseng E, Welch KC Jr. et al. Single-molecule, full length transcript sequencing provides insight into the extreme metabolism of the ruby-throated hummingbird *Alchilochus colubris*. *GigasScience.* 2018,3:1-12.

41. Moreno-Santillán DD, Machain-Williams C, Hernández-Montes G, Ortega J. De Novo Transcriptome Assembly and Functional Annotation in Five Species of Bats. *Sci Rep.* 2019;9:6222.

42. Lee JH, Lewis KM, Moural TW, Kirilenko B, Borgonovo B, et al. Molecular parallelism in fast-twitch muscle proteins in echolocating mammals. *Science*. 2018;4:eaat9660.

43. Song SJ, Sanders JG, Delsuc F, Metcalf J, Amato K, et al. Comparative analyses of vertebrates gut microbiomes reveal convergence between birds and bats. *M*B*io.* 2020;1:e02901-19.

44. Kaijitani R, Toshimoto K, Noguchi H, Toyoda A, Ogura Y, et al. Efficient de novo assembly of highly heterozygous genomes from whole-genome shotgun short reads. *Genome Res.* 2014;24:1384–1395.

45. Lowe T. tRNAscan-SE: a program for improved detection of transfer RNA genes in genomic sequence. *Nucleic Acids Res.* 1997;25:955–964.

46. Bosi E, Donati B, Galardini M, Brunetti S, Sagot MF, et al. MeDuSa: A multi-draft based scaffolder. *Bioinformatics.* 2015;31:2443–2451.

47. Walker BJ, Abeel T, Shea T, Priest M, Abouelliel A, et al. Pilon: An integrated tool for comprehensive microbial variant detection and genome assembly improvement. *PLoS One*. 2014;0112963.

48. Simão FA, Waterhouse RM, Ioannidis P, Kriventseva EV, Zdobnov EM. BUSCO: Assessing genome assembly and annotation completeness with single-copy orthologs. *Bioinformatics.* 2015;31:3210–3212.

49. Flutre T, Duprat E, Feuillet C, Quesneville H. Considering transposable element diversification in de novo annotation approaches. *PLoS One.* 2011;6:0016526.

50. Bao W, Kojima KK, Kohany O. Repbase Update, a database of repetitive elements in eukaryotic genomes. *Mob DNA.* 2015;6:4–9.

51. Tarailo-Graovac, Chen N. Using RpeatMasker to identify repetitive elements in genomic sequences. *Curr Protoc Bioinformatics*. 2009;4:bi0410s25.

52. Haas BJ, Papanicolaou A, Yassour M, Grabherr M, Blood PD, et al. *De novo* transcript sequence reconstruction from RNA-seq using the Trinity platform for reference generation and analysis. *Nat Protoc.* 2013;8:1494–1512.

53. Evans T, Loose M. AlignWise: a tool for identifying protein-coding sequence and correcting frame-shifts. *BMC Bioinformatics.* 2015;1:376.

54. Stanke M, Morgenstern B. AUGUSTUS: A web server for gene prediction in eukaryotes that allows user-defined constraints. *Nucleic Acids Res.* 2005;33:465–467.

55. The UniProt Consortium. UniProt: The universal protein knowledgebase. *Nucleic Acids Res.* 2018;46:2699.

56. Jones P, Binns D, Chang HY, Fraser M, Li W, et al., InterProScan 5: Genome-scale protein function classification. *Bioinformatics.* 2014;30:1236–1240.

57. Van der Auwera GA, Carneiro MO, Hartl C, Poplin R, Del Angel G, et al. From fastQ data to high-confidence variant calls: The genome analysis toolkit best practices pipeline. *Curr Protoc Bioinformatics.* 2013;43:11.10.1-33.

58. Li H, Durbin R. Fast and accurate short read alignment with Burrows-Wheeler transform. *Bioinformatics.* 2009;14:1754-1760.

59. Broad Institute. Picard Tools. Broad Institute, Github repository. https://github.com/broadinstitute/picard/issues/808.

60. Li H, Handsaker B, Wysoker A, Fennell T, Ruan J, et al. The Sequence Alignment/Map format and SAMtools. *Bioinformatics.* 2009;16:2078–2079.

61. Danecek P, Auton A, Abecasis G, Alberts CA, Banks E, et al. The variant call format and VCFtools. *Bioinformatics*. 2011;15:2156–2158.

62. Guindon S, Gascuel O. PhyML: “A simple, fast and accurate algorithm to estimate large phylogenies by maximum likelihood”. *Syst Biol.* 2003:52:696-704.

63. Yang Z. PAML 4 : Phylogenetic Analysis by Maximum Likelihood. *Mol Biol Evol.* 2007;24:1586–1591.

64. De Bie T, Cristianini N, Demuth JP, Hahn MW. CAFE: A computational tool for the study of gene family evolution. *Bioinformatics.* 2006;22:1269–1271.

65. Lechner M, Findeib SS, Steiner L, Marz M, Stadler PF. Proteinortho: detection of (co-)orthologs in large-scale analysis. *BMC Bioinformatics.* 2011;12:124.

66. Buchfink B, Xie C, Huson DH. Fast and sensitive protein alignment using DIAMOND. *Nat Methods*. 2015;1:59-60.

67. Katoh K, Misawa K, Kuma K, Miyata T. MAFFT: a novel method for rapid multiple sequence alignment based on fast Fourier transform. *Nucleic Acids Res.* 2002;14:3059-3066.

68. Suyama M, Torrents D, Bork P. PAL2NAL: robust conversion of protein sequence alignments into the corresponding codon alignments. *Nucleic Acids Res.* 2006;34:W609-W612.

69. Stamatakis A. RAxML version 8: a tool for phylogenetic analysis and post-analysis of large phylogenies. *Bioinformatics.* 2014;30:1312-1313.

70. Pond SL, Frost SD, Muse SV. HyPhy: hypothesis testing using phylogenies. *Bioinformatics*. 2005;5:676-9.

71. Smith MD, Wertheim JO, Weaver S, Murrell B, Scheffler K. et al. Less is More: An adaptive branch-site random effects model for efficient detection of episodic diversifying selection. *Mol Biol Evol.* 2015;5:132-1353.

72. Alexa A, Rahnenführer J, Lengauer T. Improved scoring of functional groups from gene expression data by decorrelating GO graph structure. *Bioinformatics*. 2006;22:1600-7.

73. R Development Core Team. R: A language and environment for statistical computing. R Foundation for Statistical Computing, Vienna, Austria. 2008. http://www.R-project.org.

74. Löytynoja A. Phylogeny-aware alignment with PRANK. *Methods Mol Biol.* 2014;1079:155-70.

75. Ashkenazy H, Penn O, Doron-Faigenboim A, Cohen O, Cannarozzi G, et al. FastML: a web server for probabilistic reconstruction of ancestral sequences. *Nucleic Acids Res.* 2012;40:W580-4.

76. Darriba D, Taboada GL, Doallo R, Posada D. ProtTest3: fast selection of best-fit models of protein evolution. *Bioinformatics*. 2011;8:1164-1165.

77. Kozlowski LP. IPC – Isolectric Point Calculator. *Biol Direct.* 2016;11:55.

78. Bailey SF, Guo Q, Bataillon T. Identifying drivers of parallel evolution: A regression model approach. *Genome Biol Evol.* 2018; 10:2801-2812.

79. Kelley LA, Mezulis S, Yates CM, Wass MN, Sternberg MJE. The Phyre2 web portal for protein modeling, prediction and analysis. *Nat Protoc.* 2015;10:845-858.

80. DeLano WL. PyMOL: An open-source molecular graphics tool. *CCP4 Newsletter On Protein Crystallography*. 2002;40:82-92.

81. The PyMOL Molecular Graphics System, Version 2, Schrödinger, LLC. https://pymol.org/2/

82. Gutiérrez-Guerrero YT, Ibarra-Laclette E, Martínez del Río C, Barrera-Redondo J, Rebollar EA; Ortega J, León-Paniagua L, Urrutia A, Aguirre-Planter E, Eguiarte LE. Supporting data for “Genomic consequences of dietary diversification and parallel evolution due to nectarivory in Leaf-nosed bats”. *GigaScience Databas*e. 2020. http://dx.doi.org/10.5524/100746.

Figures

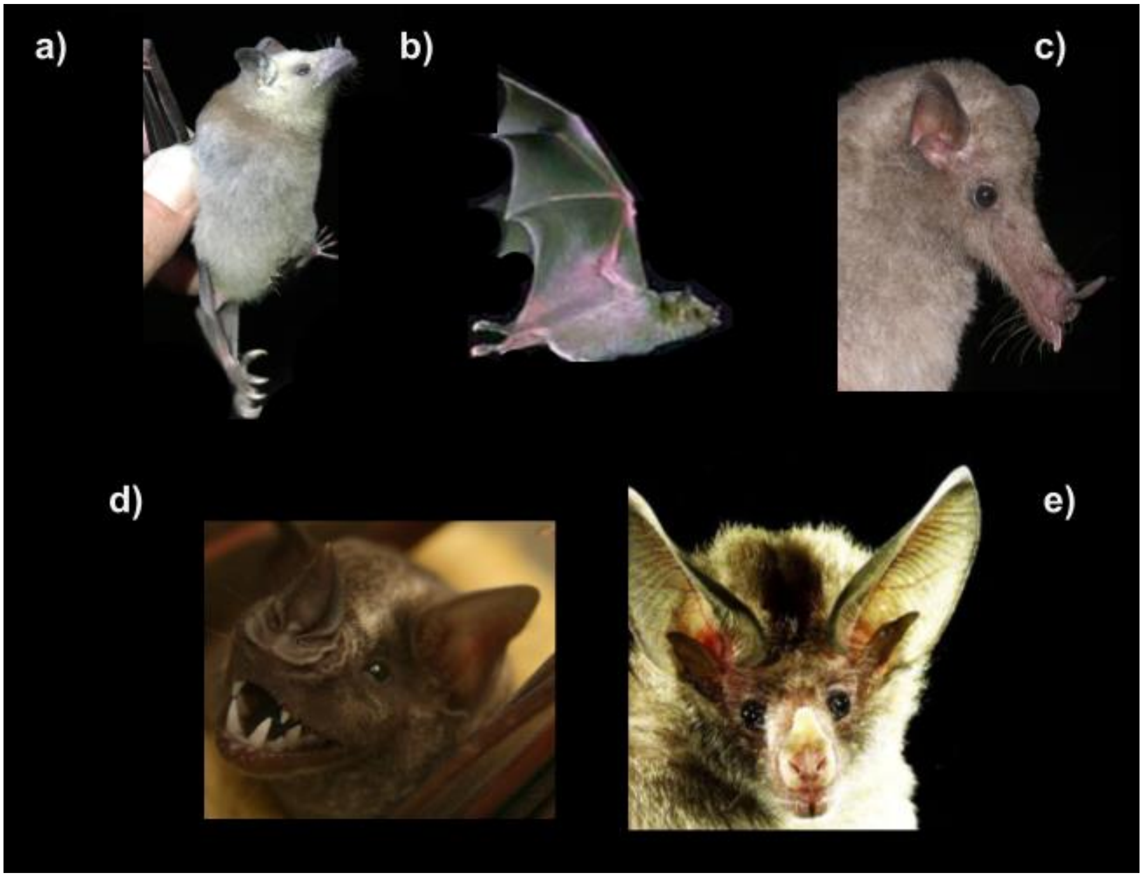

Figure 1. Phyllostomid species. a) *Leptonycteris yerbabuenae* , b) *Leptonycteris nivalis,* c) *Musonycteris harrisoni*, d) *Macrotus waterhousii,* and e) *Artibeus jamaicensis.* Photo credits: a) Daniel Zamora-Mejías, c) Rodrigo Medellín-Legorreta, d) Melissa E. Rodríguez, and b – e) Wikimedia, public domain.

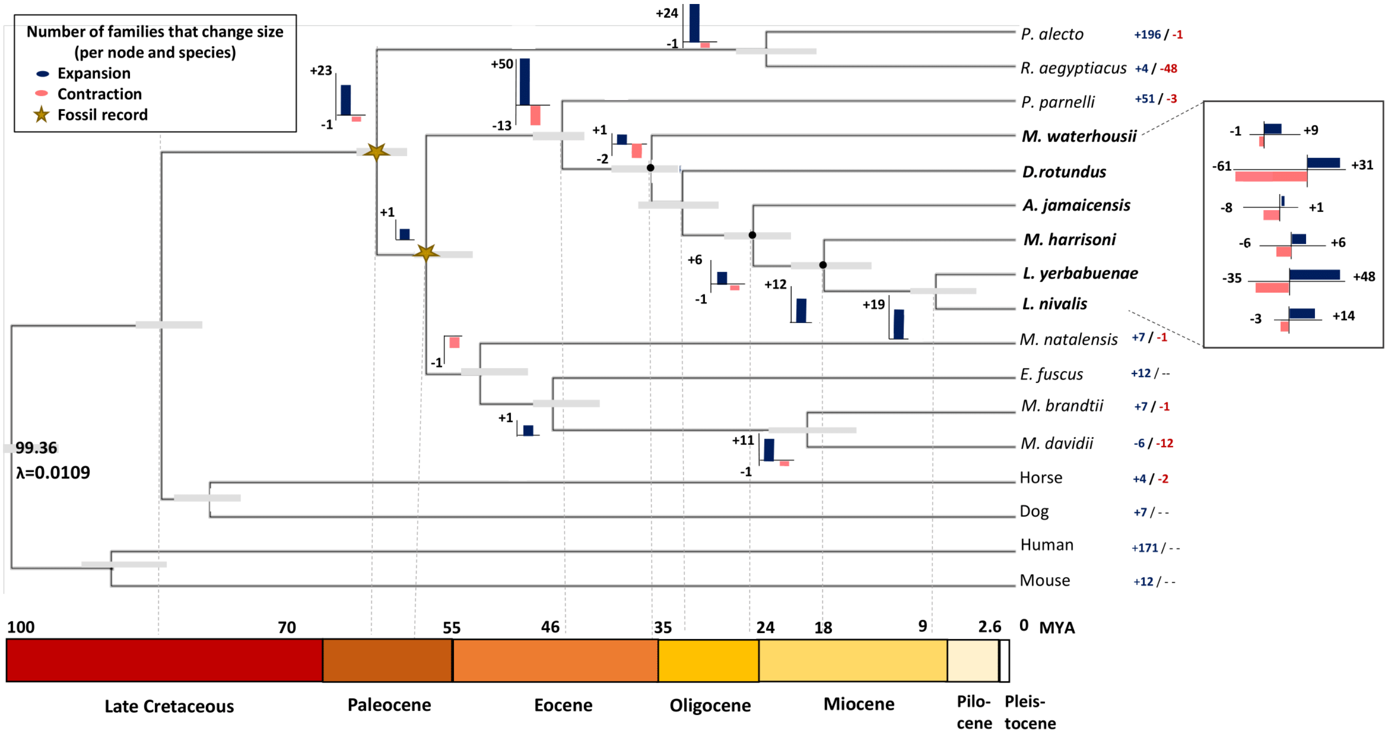

Figure 2. Phylogenetic tree constructed with 132 single copy genes and estimates of divergence times based on two fossil records (yellow stars) (see Methods). Based on 22,388 gene families we analyzed the number of orthologous families expanded (+ blue) and contracted (- red) across the phylogeny: per node (bars) and per species branch (right), with a *p-value* =< 0.01. Gray bars reflect the divergence time interval based on *95% HPD*.

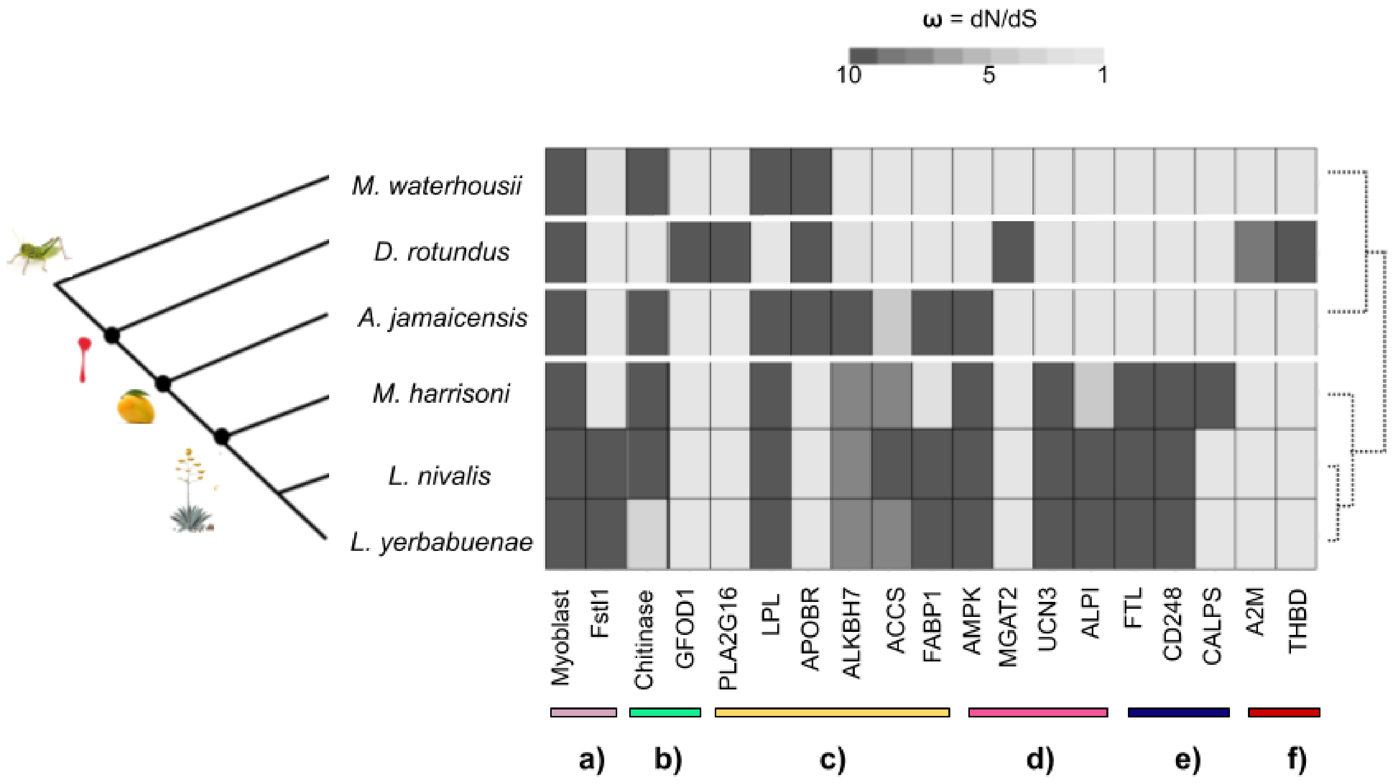

737 **Figure 3.** Positive selection in genes and proteins across the phylogeny of Phyllostomid bats, in comparison to the insect-feeder bat *Macrotus waterhousii*. Most of positive selected genes likely contribute to the regulation and processing of: a) muscle and bone development; b) carbohydrates; c) lipids; d) nutrients and food uptake; e) iron storage and calcium sources; and f) blood regulation (see genes and proteins abbreviations in Additional file 1, Table S14*)*.

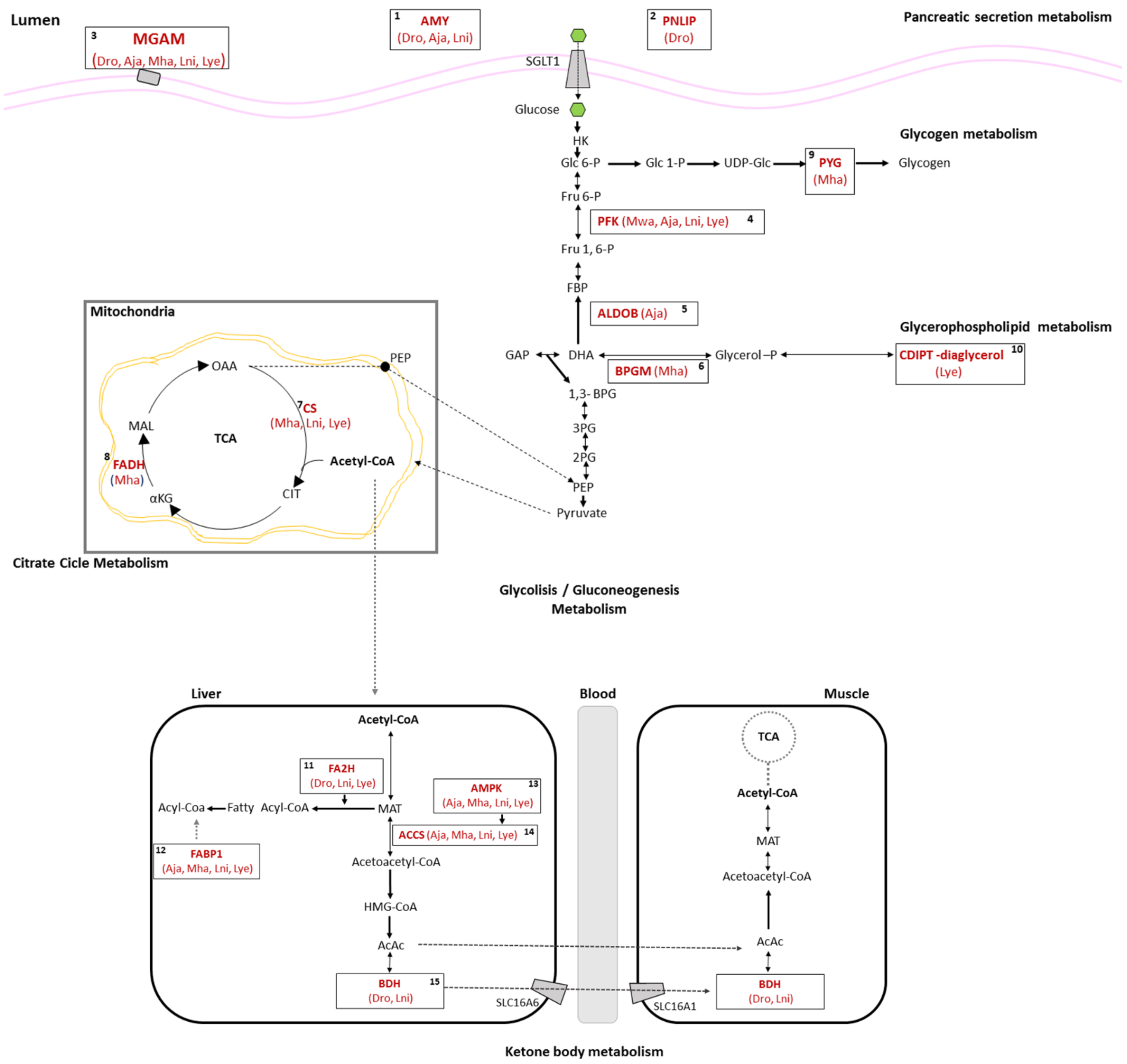

Figure 4. A subset of genes under positive selection (in red bold) that are involved in glucose and ketogen in the frugivorous (Aja: *A. jamaicensis*) and nectar-pollen bats (Mha: *M. harrisoni*, Lni: *L. nivalis*, and Lye: *L. yerbabuenae*). The diagram also identifies adaptative signals for some genes in the vampire *D. rotundus* (Dro) and the insectivore *M. waterhousii* (Mwa). The diagram is based on the KEGG metabolic pathways database and a review of the literature (see gene and proteins abbreviations in Additional file 1, Table S8).

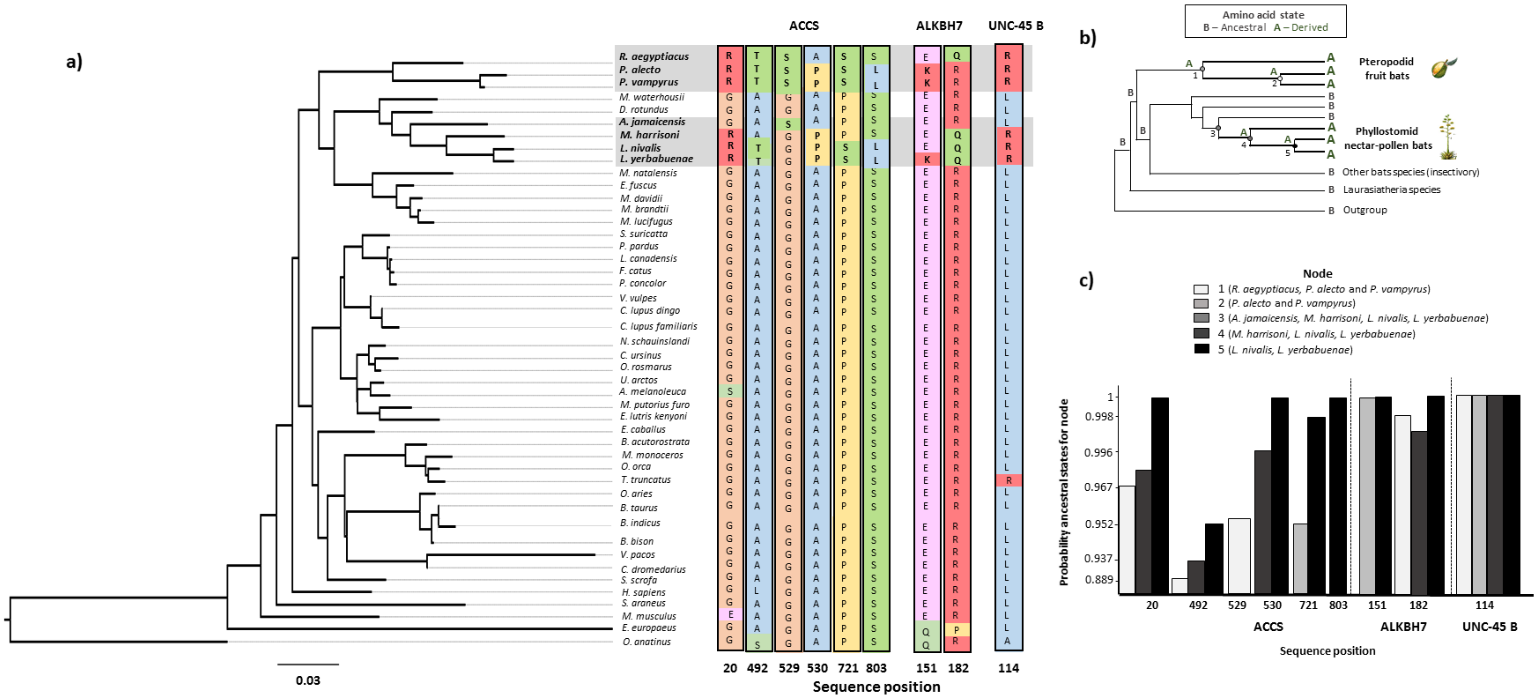

Figure 5. Parallel molecular evolution between Pteropodids (Old World) and Glossophagini (New World) bats, in three genes: Aceotacetyl CoA Synthetase (*AACS*); Alpha-Ketoglutarate-Dependent Dioxygenase Homolog 7, mitochondrial (*ALBKH7*); and UNC-45 homolog B, mitochondrial (*UNC-45 B*). a) Phylogeny reconstruction for these three genes by Maximum likelihood (using 1,827 amino acids), for 47 mammal species. b) Ancestral sequence reconstruction (for branches and nodes) to infer parallel substitutions in conserved positions for the three genes. c) Probability of replacement at each ancestral state node for each sequence position. Amino acid abbreviations: A – Alanine (non-polar); T – Threonine (polar); Q – Glutamine (polar); R – Arginine (basic-charged); K – Lysine (basic-charged); E – Glutamic acid (acidic + charged); S – Serine (polar) and L – Leucine (non-polar).

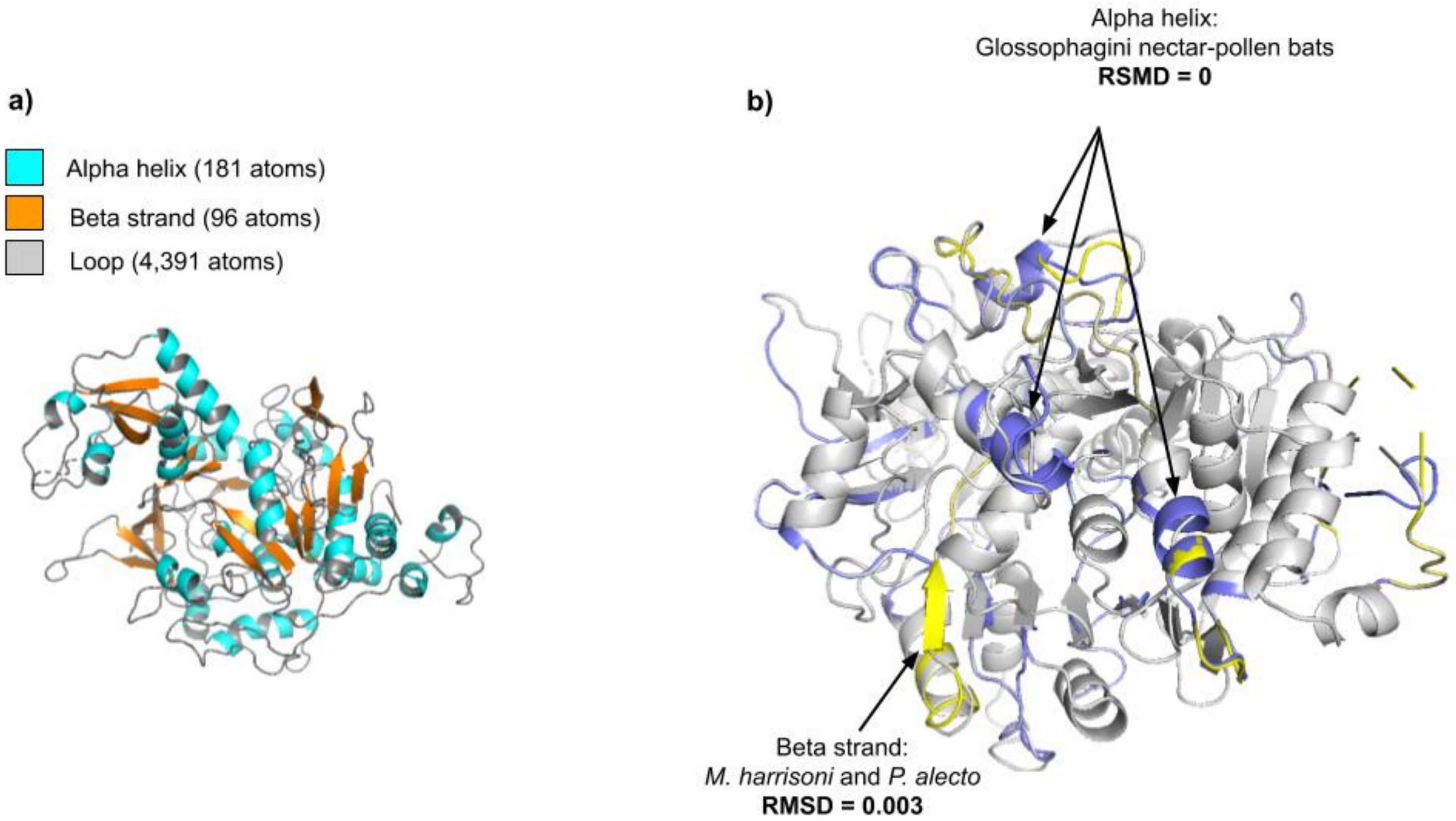

Figure 6. *ACCS* protein structure. a) 3D- structure of *ACCS* protein for *L. yerbabuenae.* b) In gray: *ACCS* 3D- structure consensus (*M. waterhousii* and *D. rotundus*). In blue: alpha-helix structures shared only for the three Glossophagini nectar-pollen feeders (*M. harrisoni*, *L. nivalis* and *L. yerbabuenae*). In yellow: beta-strand shared only between *M. harrisoni* and *P. alecto* (Pteropodid bat). RSMD score (protein 3D superposition and alignment) between pairs of species (see Additional file 1, Table S10).

Tables

Table 1. Global statistics for the nectar-pollen feeding bat *L. yerbabuenae* genome assembly.

i) Sequencing	Total raw data (Gb)	Number Reads > PHRED 30	Coverage (x)
	254.4	690,759,531	103.6
ii) Assembly	N50 (Kb) - L50	Number – Longest (Mb)	Total Size (Gb)
Contig	69.49 - 8,805	78,626 – 0.55	2.05
Scaffold	14,735.1 – 38	34,419 – 70.81	2.05
BUSCO	Completed	Fragmented	Missing
	3,864 (94.1%)	103 (2.5%)	141 (3.4%)
iii) Annotation	Number	Length (Mb)	Percent
Exons	119,036	- -	- -
CDS / Proteins	24,074	- -	- -
Repeats	3,010,348	547.05	26.64%

Table 2. Mapping statistics and SNP identification in Phyllostomid bat genomes (based on *L. yerbabuenae* genome assembly).

	Phyllostomidae			
**Species**	*Leptonycteris nivalis*	*Musonycteris harrisoni*	*Artibeus jamaicensis*	*Macrotus waterhousii*
**Diet**	Nectar-pollen	Nectar-pollen	Fruits	Insects
**Total data (Gb)**	131.4	69.2	56.6	128.4
**Coverage (x)**	54.8	30.45	25	56.34
**BUSCO**<br>**Complete**<br>**Fragmented**<br>**Missing**	93.7%<br>2.8%<br>3.5%	94.0%<br>2.4%<br>3.6%	93.0%<br>3.5%<br>3.5%	93.3%<br>3.4%<br>3.3%
**CDS/Proteins**	24,471	20,135	18,756	19,171

Table 3. GO enrichment for significant gene families per node and habit food. Gene Ontologies (GO) annotations involved in metabolism and diet are in bold (**+** Gene families expansions; **-** contractions)

**Specie and Nodes**	**Function and Metabolic Pathway**	**GO**	***p-value < 0.01***
Phyllostomid node (expansions)	+ Structural constituent of ribosome	GO:0003735	<1e-30
	+ Translation	GO:0006412	<1e-30
Phyllostomid node (contractions)	- Hydrolase activity	GO:0016788	0.00099
	**- Lipid metabolic process**	**GO:0006629**	**0.001**
	- Aspartic-type endopeptidase activity	GO:0004190	1.8e-06
D. rotundus (expansions)	+ Response to biotic stimulus	GO:0009607	5.7e-12
	+ Defense response	GO:0006952	7.8e-12
	+ Signal transduction	GO:0007165	1.3e-08
	**+ Nitrate assimilation**	**GO:0042128**	**8.8e-08**
	**+ Regulation of appetite**	**GO:0032098**	**0.00034**
	+ Protein glycosylation in Golgi	GO:0033578	0.00313
	+ GTPase activity	GO:0003924	< 1e-30
	+ Molybdenum ion binding	GO:0030151	4.5e-05
(contractions)	- Translation	GO:0006412	1e-30
	**- Calcium ion transmembrane**	**GO:0070588**	**1.3e-16**
	**- Cellular calcium ion homeostasis**	**GO:0006874**	**3.5e-09**
	- Neuron development	GO:0048666	4.2e-09
	- Microtubule-based process	GO:0007017	5.5e-08
	- Homophilic cell adhesion via plasma	GO:0007156	1.7e-07
	- Peptidyl-prolyl cis-trans isomerase	GO:0003755	< 1e-30
	- Ephrin receptor activity	GO:0005003	2.7e-21
	- Ryanodine-sensitive calcium channel	GO:0005219	6.2e-21
	- Inorganic anion exchanger activity	GO:0005452	2.1e-20
	- Ionotropic glutamate receptor activity	GO:0004970	6.3e-20
	- Voltage-gated calcium channel activity	GO:0005245	5.6e-16
A. jamaicensi, *M.harrisoni*, *L. yerbabuenae* and *L. nivalis* (expansions)	+ Translation	GO:0006412	<1e-30
	+ Integral component of membrane	GO:0016021	6.7e-06
	+ Immune response 458	GO:0006955	1.1e-05
	**+ Iron ion import membrane**	**GO:0098711**	**0.00018**
	**+ HFE-transferrin receptor complex**	**GO:1990712**	**5.7e-05**
	**+ Transferrin receptor binding**	**GO:1990459**	**7.3e-05**
(contractions)	- Protein peptidyl-prolyl isomerization	GO:0000413	< 1e-30
M. harrisoni, *L. yerbabuenae* and *L. nivalis* (expansions)	**+ Protein deubiquitination**	**GO:0016579**	**2.8e-09**
	+ Virion assembly 24	GO:0019068	0.00027
	+ Structural constituent of ribosome	GO:0003735	< 1e-30
	+ Thiol-dependent ubiquitinyl hydrolase	GO:0036459	3.6e-11
	**+ Transferrin receptor binding**	**GO:1990459**	**0.0099**
	**+ HFE-transferrin receptor**	**GO:1990712**	**0.0099**
	**+ Iron ion import membrane**	**GO:0098711**	**0.0056**

Additional file

Additional file 1. Supplementary tables. Tables S1-S14 (PDF).

Additional file 2. Supplementary figure. Figures S1-S4 (PDF).

Additional file 3. Supplementary Methods. Methods supporting the manuscript (PDF)

Table 1. Global statistics for the nectar-pollen feeding bat *L. yerbabuenae* genome assembly.

i) Sequencing	Total raw data (Gb)	Number Reads > PHRED 30	Coverage (x)
	254.4	690,759,531	103.6
ii) Assembly	N50 (Kb) - L50	Number – Longest (Mb)	Total Size (Gb)
Contig	69.49 - 8,805	78,626 – 0.55	2.05
Scaffold	14,735.1 – 38	34,419 – 70.81	2.05
BUSCO	Completed	Fragmented	Missing
	3,864 (94.1%)	103 (2.5%)	141 (3.4%)
iii) Annotation	Number	Length (Mb)	Percent
Exons	119,036	- -	- -
CDS / Proteins	24,074	- -	- -
Repeats	3,010,348	547.05	26.64%

Table3 Click here to access/download;Table;Table3.docx

Table 3. GO enrichment for significant gene families per node and habit food. Gene Ontologies (GO) annotations involved in metabolism and diet are in bold (**+** Gene families expansions; **-** contractions)

**Specie and Nodes**	**Function and Metabolic Pathway**	**GO**	***p-value < 0.01***
Phyllostomid node (expansions)	+ Structural constituent of ribosome	GO:0003735	<1e-30
	+ Translation	GO:0006412	<1e-30
Phyllostomid node (contractions)	- Hydrolase activity	GO:0016788	0.00099
	**- Lipid metabolic process**	**GO:0006629**	**0.001**
	- Aspartic-type endopeptidase activity	GO:0004190	1.8e-06
D. rotundus (expansions)	+ Response to biotic stimulus	GO:0009607	5.7e-12
	+ Defense response	GO:0006952	7.8e-12
	+ Signal transduction	GO:0007165	1.3e-08
	**+ Nitrate assimilation**	**GO:0042128**	**8.8e-08**
	**+ Regulation of appetite**	**GO:0032098**	**0.00034**
	+ Protein glycosylation in Golgi	GO:0033578	0.00313
	+ GTPase activity	GO:0003924	< 1e-30
	+ Molybdenum ion binding	GO:0030151	4.5e-05
(contractions)	- Translation	GO:0006412	1e-30
	**- Calcium ion transmembrane**	**GO:0070588**	**1.3e-16**
	**- Cellular calcium ion homeostasis**	**GO:0006874**	**3.5e-09**
	- Neuron development	GO:0048666	4.2e-09
	- Microtubule-based process	GO:0007017	5.5e-08
	- Homophilic cell adhesion via plasma	GO:0007156	1.7e-07
	- Peptidyl-prolyl cis-trans isomerase	GO:0003755	< 1e-30
	- Ephrin receptor activity	GO:0005003	2.7e-21
	- Ryanodine-sensitive calcium channel	GO:0005219	6.2e-21
	- Inorganic anion exchanger activity	GO:0005452	2.1e-20
	- Ionotropic glutamate receptor activity	GO:0004970	6.3e-20
	- Voltage-gated calcium channel activity	GO:0005245	5.6e-16
A. jamaicensi, *M.harrisoni*, *L. yerbabuenae* and *L. nivalis* (expansions)	+ Translation	GO:0006412	<1e-30
	+ Integral component of membrane	GO:0016021	6.7e-06
	+ Immune response 458	GO:0006955	1.1e-05
	**+ Iron ion import membrane**	**GO:0098711**	**0.00018**
	**+ HFE-transferrin receptor complex**	**GO:1990712**	**5.7e-05**
	**+ Transferrin receptor binding**	**GO:1990459**	**7.3e-05**
(contractions)	- Protein peptidyl-prolyl isomerization	GO:0000413	< 1e-30
M. harrisoni, *L. yerbabuenae* and *L. nivalis* (expansions)	**+ Protein deubiquitination**	**GO:0016579**	**2.8e-09**
	+ Virion assembly 24	GO:0019068	0.00027
	+ Structural constituent of ribosome	GO:0003735	< 1e-30
	+ Thiol-dependent ubiquitinyl hydrolase	GO:0036459	3.6e-11
	**+ Transferrin receptor binding**	**GO:1990459**	**0.0099**
	**+ HFE-transferrin receptor**	**GO:1990712**	**0.0099**
	**+ Iron ion import membrane**	**GO:0098711**	**0.0056**

Table2 Click here to access/download;Table;Table2.docx

Table 2. Mapping statistics and SNP identification in Phyllostomid bat genomes (based on *L. yerbabuenae* genome assembly).

	Phyllostomidae			
**Species**	*Leptonycteris nivalis*	*Musonycteris harrisoni*	*Artibeus jamaicensis*	*Macrotus waterhousii*
**Diet**	Nectar-pollen	Nectar-pollen	Fruits	Insects
**Total data (Gb)**	131.4	69.2	56.6	128.4
**Coverage (x)**	54.8	30.45	25	56.34
**BUSCO**<br>**Complete**<br>**Fragmented**<br>**Missing**	93.7%<br>2.8%<br>3.5%	94.0%<br>2.4%<br>3.6%	93.0%<br>3.5%<br>3.5%	93.3%<br>3.4%<br>3.3%
**CDS/Proteins**	24,471	20,135	18,756	19,171

Figure4 Click here to access/download;Figure;Figure4.png

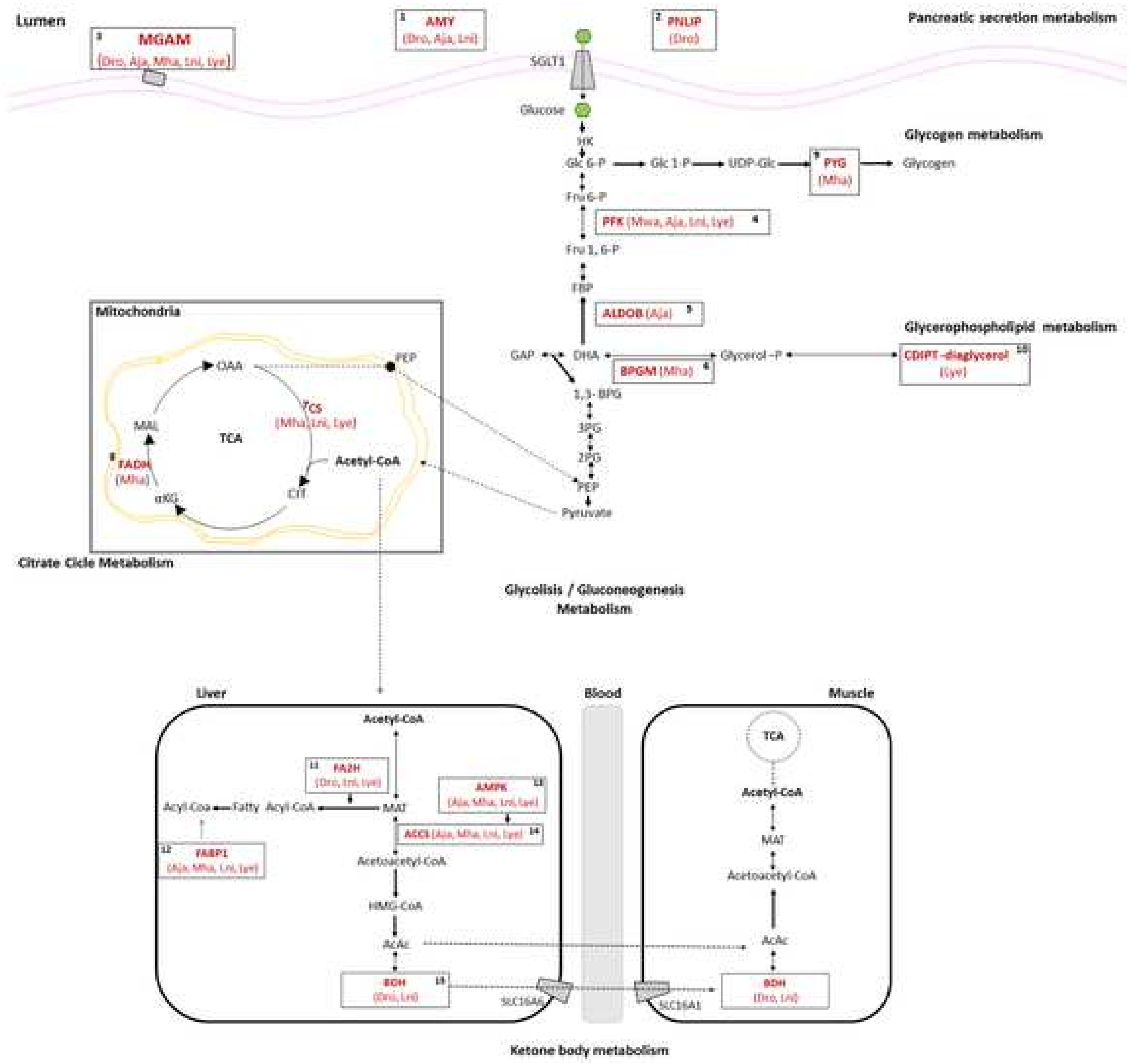

Figure5

Click here to access/download;Figure;Figure5.png

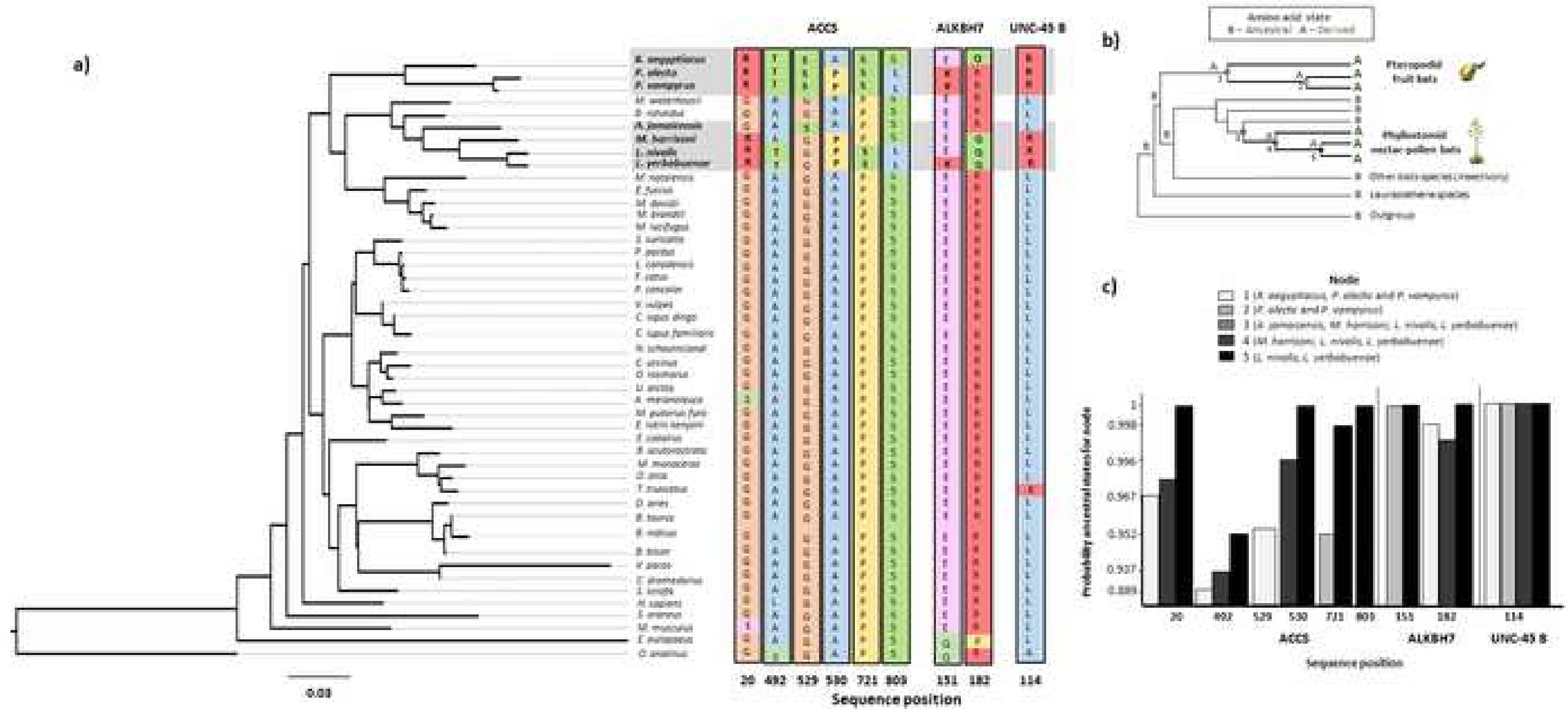

 Click here to access/download;Figure;Figure6.png

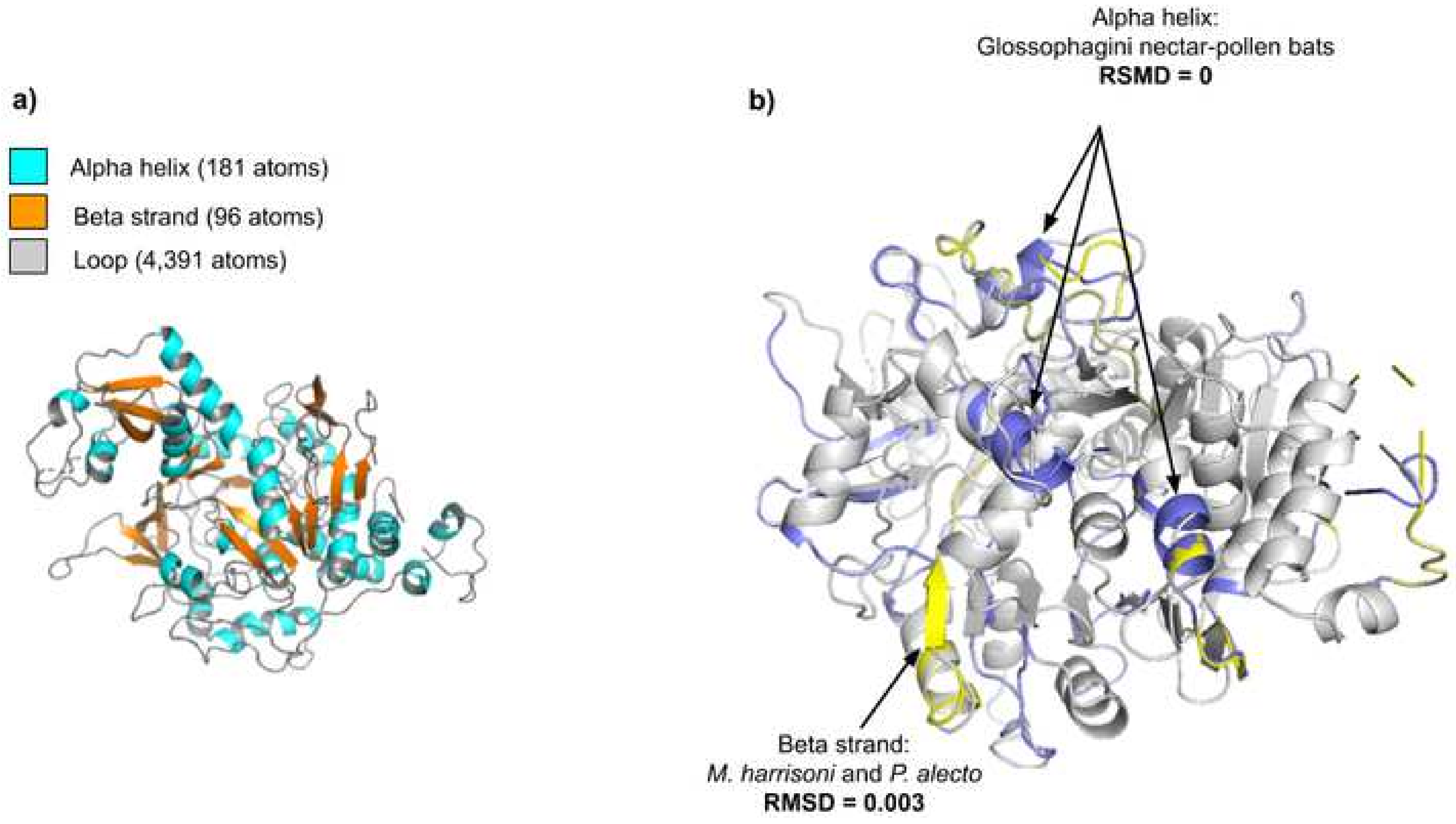

 Click here to access/download;Figure;Figure_1.png

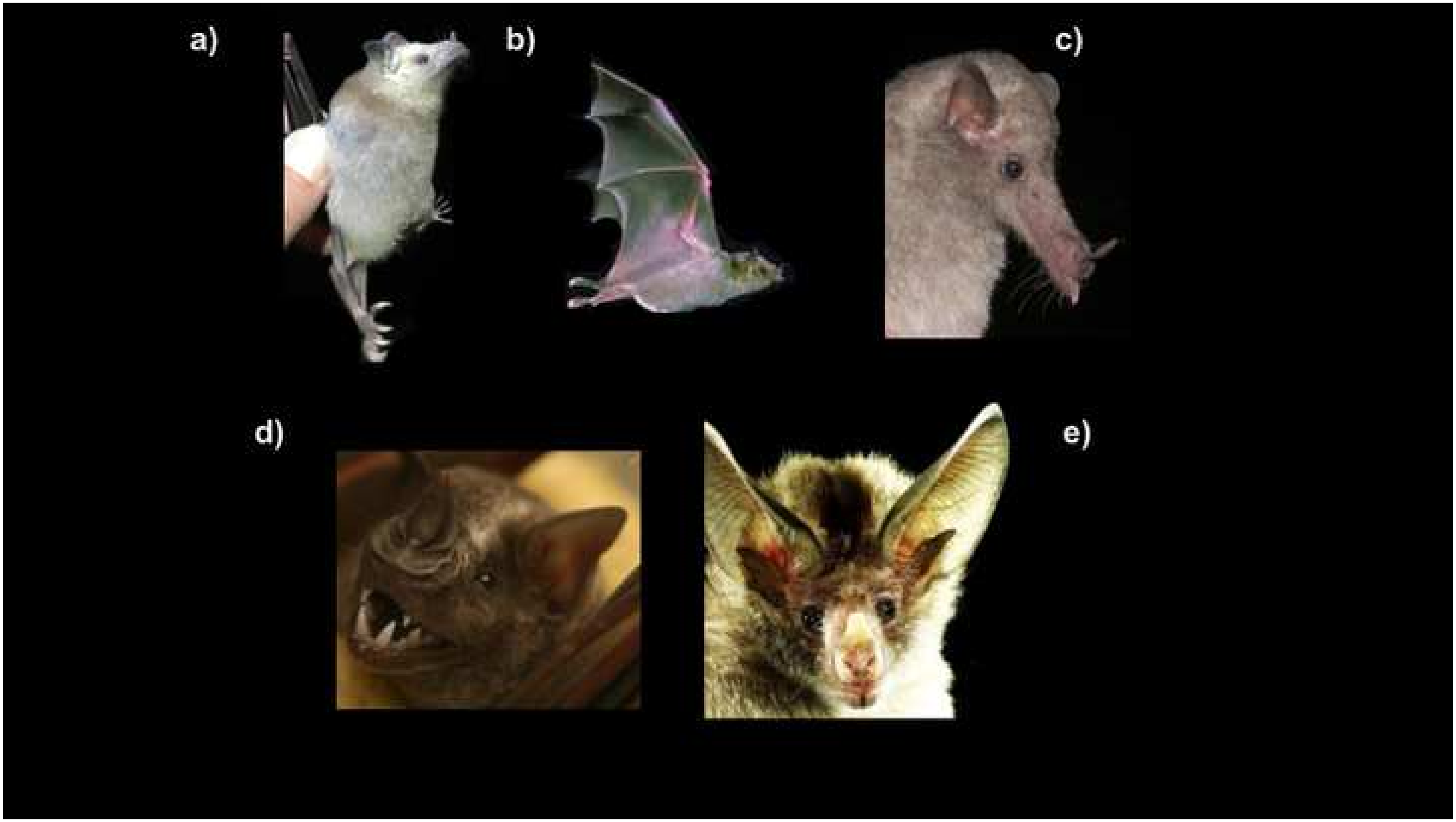

Figure2 Click here to access/download;Figure;Figure2.png

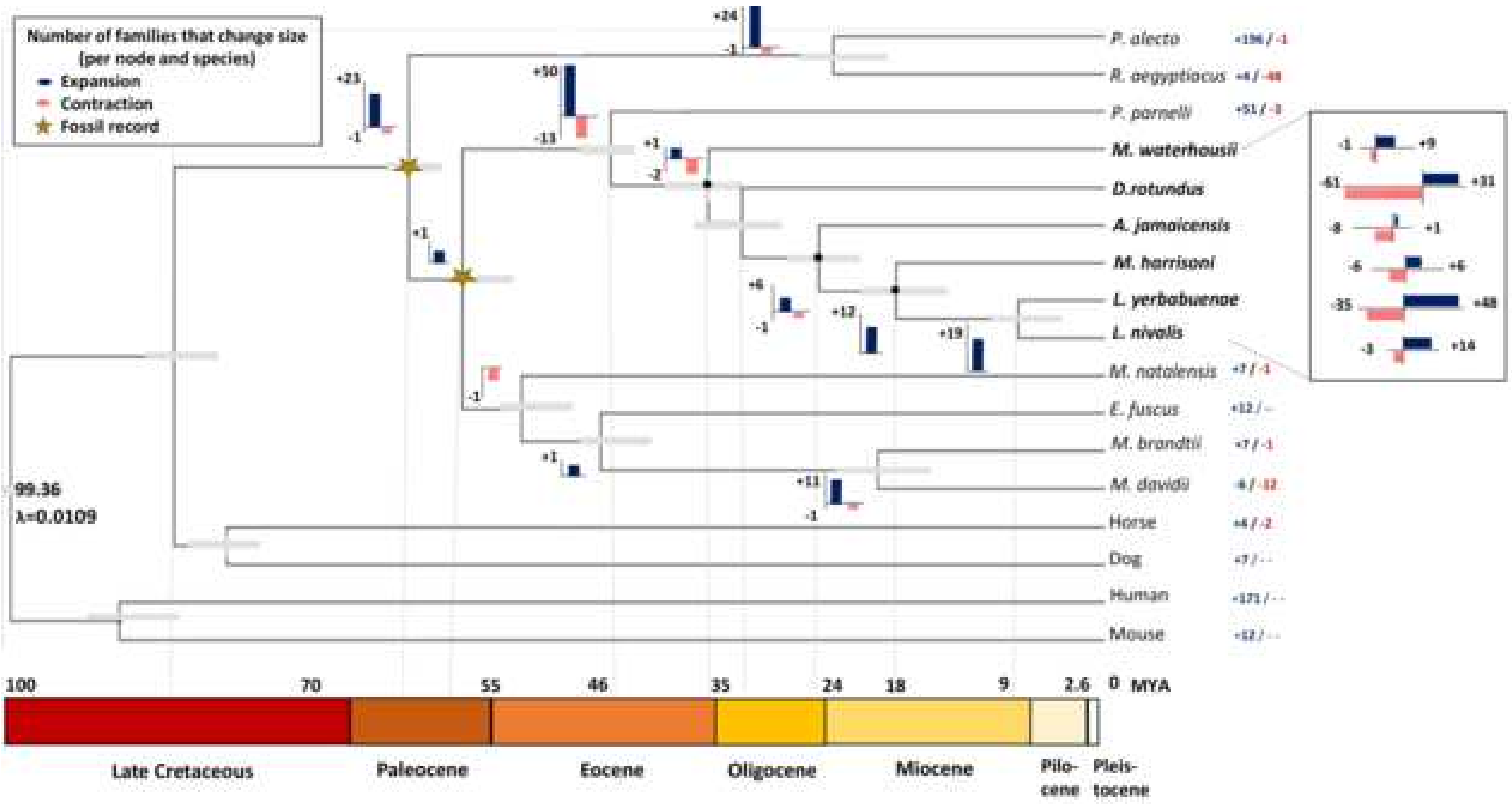

Figure3

Click here to access/download;Figure;Figure3.png

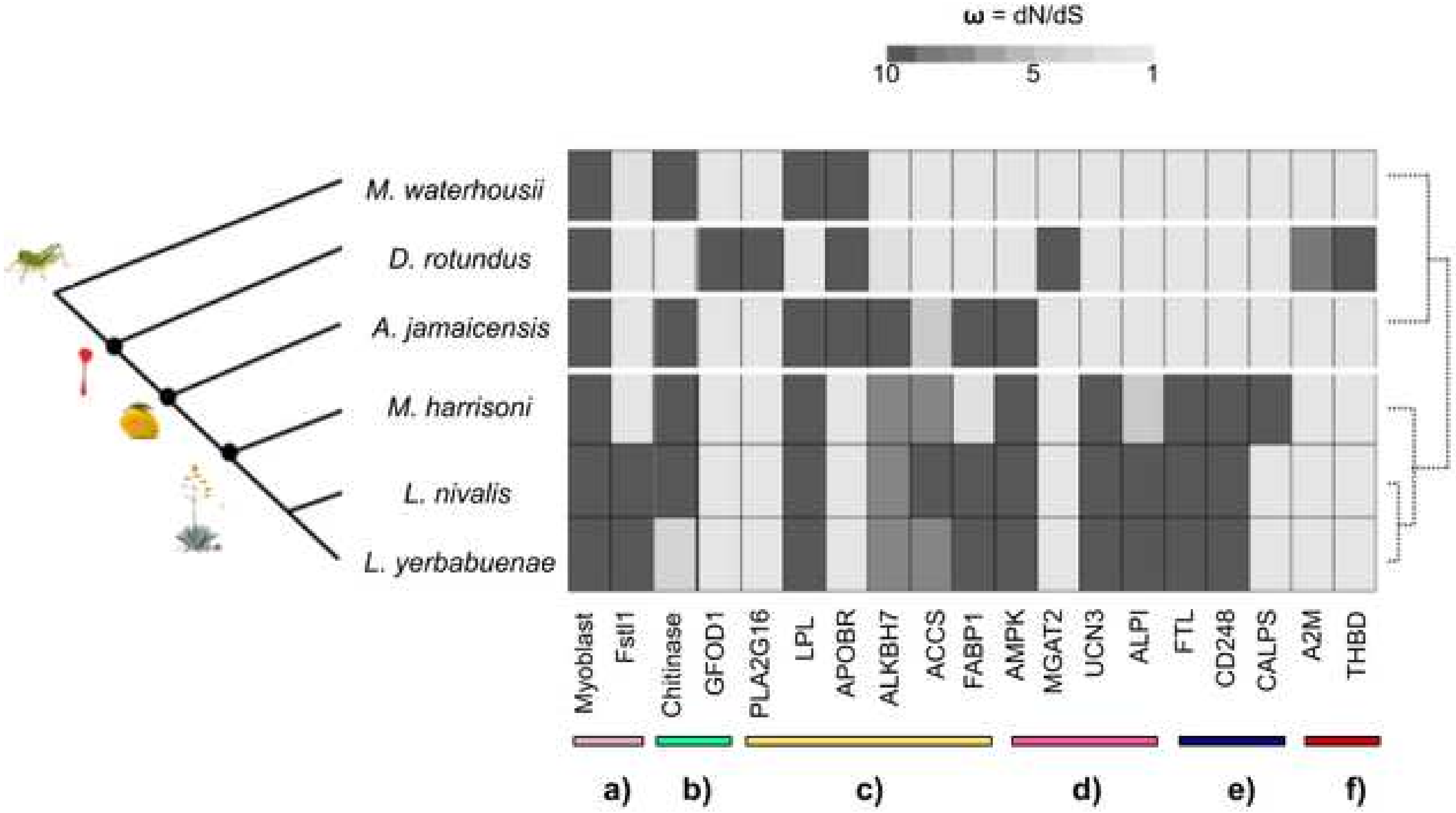

Additional File 2

Click here to access/download
Supplementary Material
AdditionalFile2.pdf

Additional File 3

Click here to access/download
Supplementary Material
AdditionalFile3.pdf

Additional File 1

Click here to access/download
Supplementary Material
Additional_File1.pdf

 Click here to access/download;Personal Cover;Reviewers_Comments_2020.docx

Reviewer's Comments

This study has sequenced and assembled the genomes of a number of bats showing diverse feeding strategies, and explored the evolutionary adaptations underpinning dietary niche specialization. The authors have fully addressed my initial concerns regarding selection tests, have provided a wealth of data to support their findings and have also provided an incredibly thorough guide on their methods that I think will benefit many researchers. I have some small, largely trivial issues below. Most these are concerned with the written English rather than the methods used. I am therefore happy to support this paper for publication, pending these minor changes.

Answers are in bold

We appreciate your support and comments.
We are certain that your suggestions have been crucial to improve and make more accurate our manuscript.
In particular, we appreciate your remark on the changes in description of the methods, that we hope will be useful.

Minor points

Line 163: insects -> insect's

Thank you for your correction, we made the change.
Line 165

Line 163: I feel like trehalOse should be the sugar in insect blood, rather than trehalase, if trehalase is the enzyme that degrades it.

Thank you for your observation, we are talking about trehalose, we are sorry for the mistake.
Line 165

Line 231: those than -> those that

Thank you, we changed it.
Line 232

Line 233: This relates to my comment on line 163. Does *trehalase* digest trehalase in insects, such that one enzyme degrades another enzyme, or does *trehalase* degrade the trehalose sugar?

We appreciate your comment, as it is very relevant. Most of the vertebrates have the capacity to digest dietary trehalose with the membrane bound intestinal enzyme trehalase.
We have modified this section.
Lines 234-235

Line 233: Do the authors have any ideas as to why the ability to digest insects may be maintained in bats, nit not the ability to digest the trehalase/trehalose sugar/enzyme in insect blood?

This issue is really interesting, as a parallel change seem to have happened in birds. Even those specialist bats, such as hematophagous and nectar-feeding species have the capacity to digest insects exoskeletal chitin. We consider two possibilities for the loss of *trehalase*. One is that the main dietary value of the insects is for lipids and proteins, and energy (as sugars) would be less important, and once the ability to digest trehalose is loss, there is no way they can recuperate it. On the other hand, we suggest that gut microbiome plays an important role to digest trehalose. The microbiome role is discussed in line 291-296.

Line 260: that it may -> that may

Thank you.
Line 262

Line 277: When the authors mention convergent evolution here, do they mean specifically dietary genes or the genome and physiology of the bat as a whole? Please clarify.

Thank you. We meant specifically parallel evolution due to nectar-feeding dietary specialization.

"Our findings suggest that parallel evolution due to nectar-feeding dietary specialization is likely a consequence of high metabolic demands required for foraging on flowers and fruits."
Lines 279-280

Line 318: I have not seen 'accurate' used in the context the authors use it here. Perhaps another word such as 'validate'can be used instead?

We apologize for the mistake.

" To optimize and extend the genome assembly"
Lines 321-322

Line 354 Perhaps consider "Repeatmasker pipeline" rather than "pipeline of repeatmasker"

We appreciate your suggestion.
Line 358

Line 373: I think "proteins" should be "protein's"

Thank you.
Line 377

Line 373: DIAMOND is also a program, so consider saying "programs DIAMOND and Proteinortho"

Thank you, we made the change.

Line 381

Line 380: “paralogous, sequences” -> “paralogous sequences”

Thank you, we made the change.
Line 383

Line 384: Were the poorly aligned regions removed based on a visual inspection or something like Gblocks?

We carried out a visual inspection and calculated the alignment length with a bash script.

“Each cluster was aligned with MAFFT aligner tool (67), we retained alignment sequences where the length is within 80 to 120% relative to the human and mouse sequences, and poorly aligned regions were removed by a visual inspection. ”
Line 386-388

Line 387: I think “RAxML tool” can just be “RaxML”

We appreciate your suggestion.
Line 391

Line 390: The authors describe how they calculated “synonymous sites and nonsynonymous sites (dN/dS) rates, and the average ratio of substitution per site (ω=dN/dS)”, however I would have assumed that these were essentially the same things, and don’t need to be stated twice as it is written, at least as far as dN/dS and w=dN/dS is concerned.

Thank you for your observation, we estimated the ratio of substitution per site.
Line 395

Line 402: No need for the “,” after the word aBSREL.

Thank you.
Line 407

Line 417: “was composed from 12 to maximum 30” -> “was composed of between 12 and a maximum 30” perhaps?

Thank you for your suggestion.
Lines 422-423

Line 424: “the accurate” –> “ the accurate ones”

Thank you.
Line 429

Line 424: “program” -> “programs”

Thank you.

Line 430

Line 442: “Independantly” –> “Independent”

Thank you.
Line 453

Line 446: The phylogenetic tree section seems out of context here, as trees have been generated throughout the methods up to this point. The authors should consider moving this section or being explicit as to the function of the tree generated in this section.

Thank you, we re-ordenized this section.
Lines 446-450

Line 451: The authors should consider adding one line at the start to give context for the reasoning behind modelling, for example “To explore the effects of selected sites on the protein 3D structure..” or something similar.

We appreciate your suggestion.

“To explore the effects of positive selection and the radical amino acid substitutions, we modeled the second and tertiary structure of the protein *Acetoacetyl CoA Synthetase* (ACCS) for *M. waterhousii*, *D. rotundus*, *M. harrisoni*, *L. nivalis*, *L. yerbabuenae* and *P. alecto*. ”
Lines 459-461

Table 2: there was an odd symbol in the brackets under nucleotide diversity on my screen. Double check that it is not an error!

Thank you, we modified this section.

Figure 1:Purely out of curiosity, do the major expansion events correlate with known climate events occurring in the various geological epochs?

This is an interesting question., but we have not formally explored this. In the case of the nectar-pollen feeder clade, we found an important gene family expansion event. This is interesting, because the divergence of the Glossophagini bats started in the Mid-Miocene from 21 to 7 Mya, coinciding with some environmental changes and the increase of food resources at the “Climatic Optimum” period.

On the other hand, the major gene family expansion was detected at the Microchiroptera node, in the Eocene period, where the Earth responded to higher levels of carbon dioxide and an increment in the temperature, warmer than today.

We will analyze in detail these gene families expansions in a future manuscript, incorporating some analysis such as phylostratigraphy and gene family calibration. Thank you for the comment.

Additional File 1, Table S1-6: Some numbers have “,” in them, others don’t. Please ensure they all do.

We apologize for the mistake, we made the change.

Table S6: please change LTR to LRT. Are these p-values corrected for multiple testing? It would also be helpful to highlight significant ones with a “*” or something similar.

We included a column with the *p-values* adjust by FDR and we highlighted those significant genes.

Reviewer #2: The authors made a great effort to make changes in this revision based on reviewers' comments. I generally agree with the authors for their responses to my previous comments. However, as I look through the whole MS, I found many minor errors which can be avoided if authors are meticulous during writing. So I strongly recommend the authors to reread the whole MS carefully to correct possible minor errors.

We appreciate your support and comments. We have read carefully all the manuscript, and double-checked.

Below are some examples.

In "Rapidly evolving genes across the whole genome", the authors did not provide the specific total number of positively selected genes, and also some words about enrichment analysis.

We appreciate your observation, we have incorporated more information.
Lines 151-155.

“For all Phyllostomid bats, we identified 42 genes with robust signals of positive selection (FDR $p < 0.05$). According with the enrichment analysis, most of the adaptive genes are related to immune response, DNA repair, inflammatory response, RNA catalytic process and genes that mediate muscle function (such as *Myoblast and PAMR1*) (Fig. 2; see Additional file 1, Table S6-TableS8) (19).“

In Table S6, LTR is still used (another reviewer had pointed out this mistake).

We deeply apologize for this repeated mistake.
We changed LTR to LRT.

In Additional file 1, "Table S7" was wrote as "Table S8", so there are two "Table S8".

We are sorry and we changed the number of this figures.

"Table S6. LTR construction and ω ratio", I did not see results aboutω ratio, but just P values.

We appreciate your observation.
We have incorporated the *p-value* correction and highlighted those significant genes.

Table S7 "GO enrichment for those positive selected genes for each Phyllostomid specie", the last word should be "species"

Thank you, we modified it.

Line 231, "than" should be "that"

We apologize for the mistake, we change it.
Line 232

Line 359, what software was used to construct the phylogeny based a total of 132 genes? I find it in the additional file 3, PhyML3. I think that the authors should mention this in the main text. In addition, the authors did not mention that whether these 132 genes are concatenated or not in building the tree.

Thank you, we included the information in the main text.

"*A total of 132 single-copy orthologous genes (61,331 amino acids sites), across 18 mammals were concatenated to reconstruct a phylogenomic tree (best-fit model distribution JTT, +G +I +I+G and 80% consensus threshold) using PhyML3 (62) (see* Additional file 3, Methods*).* "
Lines 362-364

Line 387, RaxML

Thank you, we changed RaxML to RAxML.
Line 391

Line 442, "independently" should be "independent"

Thank you, we modified it.
Line 453

Line 450, no parameters are provided for RAxML analysis.

Thank you, we included the parameters used in the analysis

" The phylogenetic tree was constructed using a Maximum Likelihood method with RAxML (-p 12345 -m PROTCATLG)."
Lines 449-450

Line 707, genes

We apologize for this mistake.
Line 721

Personal Cover Click here to access/download;Personal Cover;CoverLetter_GigaScience.docx

April 2, 2020

Dr. Lauri Goodman
Editor-In-Chief
GigaScience
Beijing Genomics Institute,
China

Dear Dr. Lauri Goodman and Dr. Hans Zauner,

We wish to resubmit the enclosed manuscript for consideration in *GigaScience.*

Our manuscript entitled: "*Genomic consequences of dietary diversification and parallel evolution due to nectarivory in Leaf-nosed bats*", describes the genomic changes associated with the remarkably divergent feeding modes in the large adaptive radiation occurring in the Phyllostomid bat diets, which range from blood to nectar and pollen. We sequenced and assembled genomes from several species of nectar/pollen feeders, as well as from an insectivore, and a fruit-eater. We also used previously published genomic data from a vampire to assess the genomic evolutionary changes associated with different diets. We identified the expansions and contractions in gene families associated with dietary shifts and identified genes under selection. In short, we described in this paper, the genomic changes that accompany evolutionary shifts in these bats and that underlay some of the physiological mechanisms that permit these bats to subsist on such contrasting and extreme diets. Importantly, our analyses also identified convergent genomic changes between Phyllostomid nectar/pollen feeding bats, which are found only in the New World, and Old World frugivorous Pteropodids. Thus, we documented not only the divergence associated with shifts in diet, but convergence in genomic traits between bats with similar feeding habits but independent evolutionary origins.

1

We believe that our study is of interest to the broad readership of your journal because 1) we

document for the first time the full genomes of bats in one of the largest mammalian radiations, and these bats have undoubtedly some of the most extreme diets among mammals; 2) we provide a striking example of convergence at the genomic level.

We have carefully reviewed all the comments made by the reviewer and approached all of their concerns. In particular, we performed additional methods to detected genes under positive selection, to identify parallel molecular evolution, and we modeled an important protein for all the Phyllostomid species sequenced and other bats species. Also, we have described in detail all the used methodologies, including an additional file with the most important commands performed in this research.

The detailed and specific answer to each comment is attached in the answer to reviewer document.

We want to point out that co-authors have scrutinized a final draft and approved submission.

The manuscript includes 18 pages, 3 tables, 6 figures and three files of supplementary material (one file: tables; second file: figures and third file: methodology section (with code and scripts). We followed the author's guidelines and submission policies. This manuscript has not been previously published and the authors declare no conflicts of interest. We hope that you will find our manuscript interesting and relevant for publication in *GigaScience* and we thank you in advance for considering our study.

Please do not hesitate to contact us if you require any additional information.

Sincerely yours,

Dr. Luis E. Eguiarte

Professor

Lab. Evolución Molecular y Experimental,
Departamento de Ecología Evolutiva,
Instituto de Ecología,
Universidad Nacional Autónoma de México
fruns@unam.mx

Biol. Yocelyn T. Gutiérrez-Guerrero

Ph. D student

Lab. Evolución Molecular y Experimental,
Departamento de Ecología Evolutiva,
Instituto de Ecología,
Universidad Nacional Autónoma de México
yoss_279@comunidad.unam.mx
