# Supplementary material for: Genomic consequences of dietary diversification and parallel evolution due to nectarivory in leaf-nosed bats
Source: Gigascience. 2020 Jun 6;9(6):giaa059. doi: 10.1093/gigascience/giaa059 (PMC7276932; doi:10.1093/gigascience/giaa059)
Supplement: giaa059_Supplemental_Files [file giaa059_supplemental_files.zip › AdditionalFile2.pdf]

Genomic consequences of dietary diversification and parallel evolution due to nectarivory in Leaf-nosed bats

Yocelyn T. Gutiérrez-Guerrero[1], Enrique Ibarra-Laclette[2], Carlos Martínez del Río[3], Josué Barrera-Redondo[1], Eria A. Rebollar[4], Jorge Ortega[5], Livia León-Paniagua[6], Araxi Urrutia[7], Erika Aguirre-Planter[1] and Luis E. Eguiarte*[1]

Additional File 2.

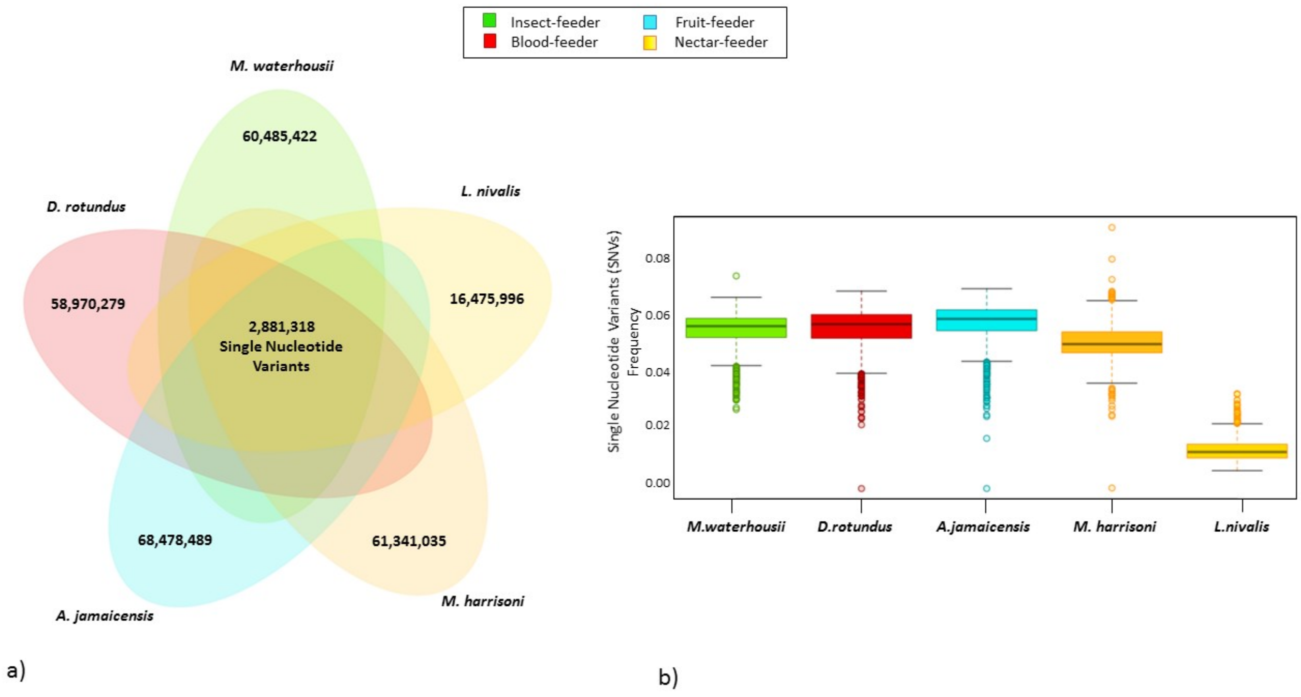

Figure S1. a) Venn diagram showing the number of shared and unique *Single Nucleotide Variants* for each Phyllostomid bat based on *L. yerbabuenae* genome. b) Nucleotide diversity estimated across sliding windows of 1 Mbp for each Phyllostomid, based on *L. yerbabuenae* genome assembly.

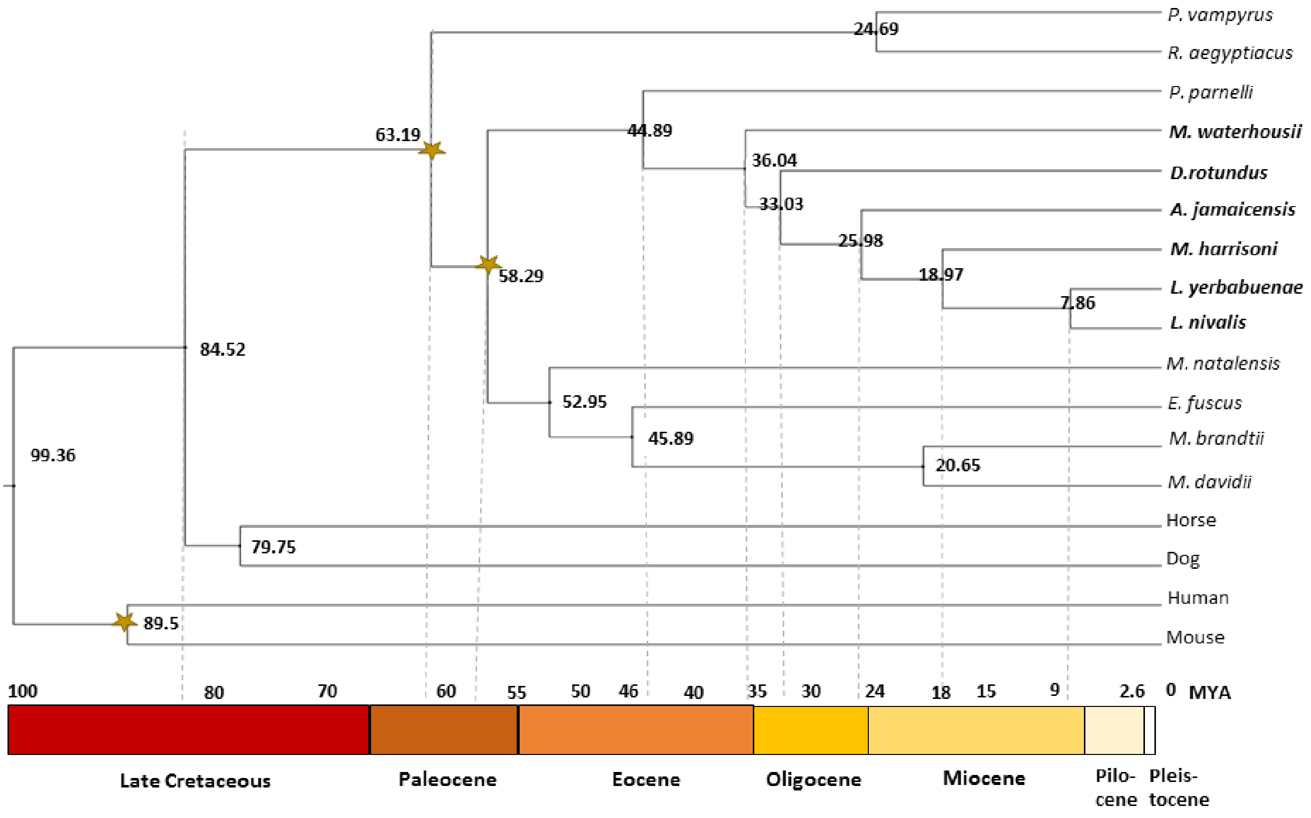

Figure S2. Phylogeny constructed based on 132 single copy genes and calibrated using three fossil records.

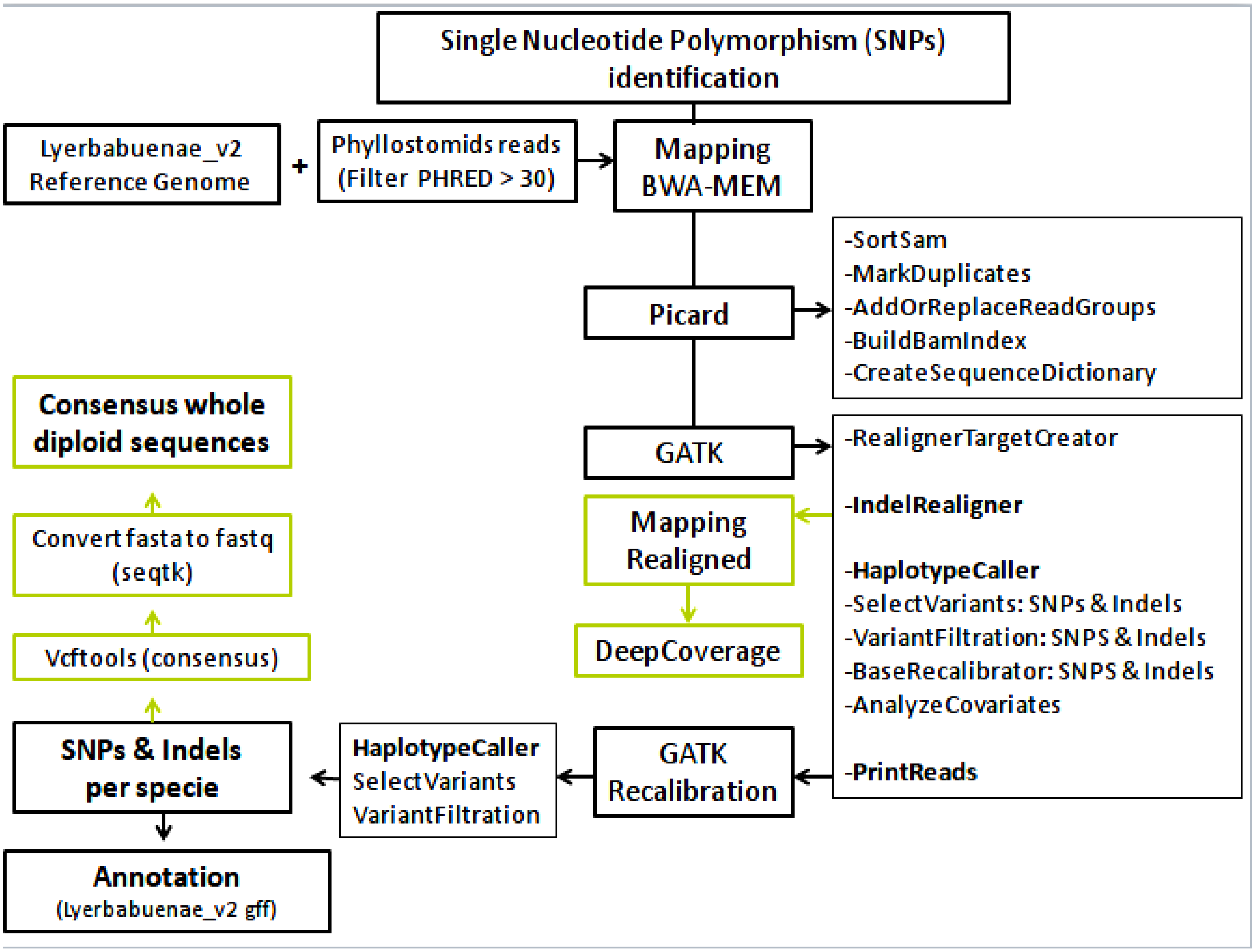

Figure S3. Pipeline for the reference-genome assembly construction.

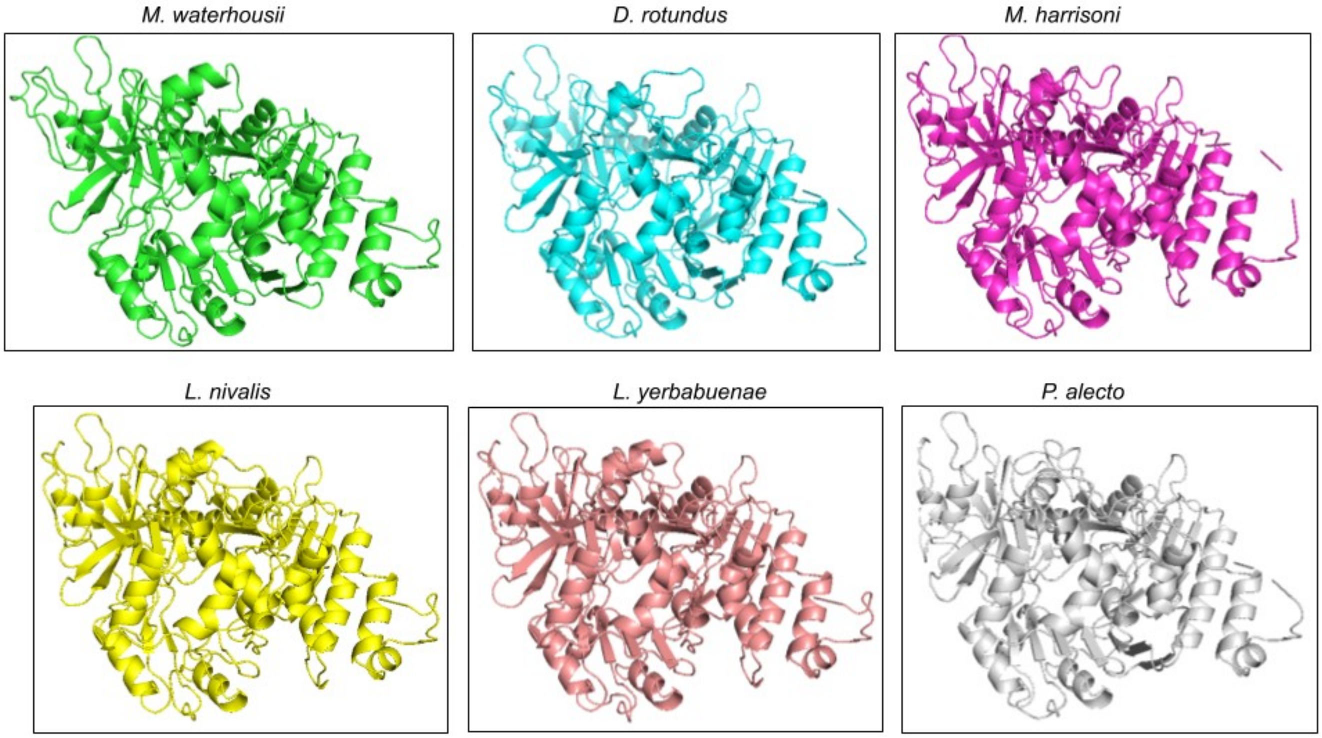

Figure S4. *ACCS* 3D protein structure for each bat species.
