# Supplementary material for: Genomic consequences of dietary diversification and parallel evolution due to nectarivory in leaf-nosed bats
Source: Gigascience. 2020 Jun 6;9(6):giaa059. doi: 10.1093/gigascience/giaa059 (PMC7276932; doi:10.1093/gigascience/giaa059)
Supplement: giaa059_Supplemental_Files [file giaa059_supplemental_files.zip › AdditionalFile3.pdf]

Genomic consequences of dietary diversification and parallel evolution due to nectarivory in Leaf-nosed bats

Additional File 3.

Supplementary Methods

Section SI 1

Animal sampling and genome sequencing An adult male *Leptonycteris yerbabuenae* was collected and processed onsite at the cave “El Salitre” in Morelos state, Mexico (18°44’28’‘N, 99°10’46’’ W). All procedures were carried out in accordance with Federal Mexican Procedures (Guidelines of Secretaria de Medio Ambiente y Recursos Naturales, SEMARNAT), permit: SGPA/DGVS/07161/15.
The Zoology Museum “Alfonso L. Herrera” (Facultad de Ciencias, UNAM), donated the tissues samples from four leaf-nosed bats: *Macrotus waterhousii* (insectivore, catalog sample: 12465), *Artibeus jamaicensis* (frugivore, catalog sample: 12380), *Leptonycteris nivalis* and *Musonycteris harrisonii* (nectarivores, catalog sample: 6432 and 6218).

For all leaf-nosed bats species, we isolated their DNA using Phenol-Chloroform protocol and DNA Blood and Tissue Kit (Qiagen). We performed high whole genome sequencing for *L. yerbabuenae* using Illumina HiSeq 4000 150 PE platform (the genomic sample sequenced on two lanes). Additionally, for the same sample we sequenced the RNA-Seq from five tissues (brain, pancreas, kidney, lung, and liver).

We sequenced the whole genome of *M. waterhousii, L. nivalis, M. harrisoni* and *A. jamaicensis* by Illumina HiSeq 4000 150 PE (Vincent J. Coates Genomics Sequencing Laboratory, qb3 Berkeley, USA).

Section SI 2

Denovo genome assembly

To assembly the whole genome and transcriptome of *L. yerbabuenae*, genomic reads were filtering and cleaning. The assembly genome was constructed de novo using Platanus v. 2.4.3, with the following parameters: heterozygosis u= 0.4 and initial kmer= 32.

Additionally, we performed two iterations with Pilon for correcting bases. With MeDuSa, we performed a scaffolding on the *L. yerbabuenae* assembly (Lyerbabuebae_v1) using the assembly genome of the big brown bat: *Eptesicus fuscus* (Bioproject accession: PRJNA232522). We evaluated the global genome assembly metrics and measure for quantitative assessment of the genes content with BUSCO v3 software and the Mammalia odb9 database.

```
##Platanus
platanus assemble -t 20 -f *.fastq -o Lyerbabuena_assembly -m 500 -u 0.4 1> Lyerbabuenae
_assembly_out.txt 2> Lyerbabuenae_assembly_log.txt

platanus scaffold -o Lyerbabuenae_scaffolding -c Lyerbabuena_assembly_contig.fa -b Lyerb
abuena_assembly_contigBubble.fa -IP1 ./Trimmed_R1.fastq  ./Trimmed_R2.fastq -u 0.4 -t 40
2> Lyerbabuenae_scaffolding.log

platanus gap_close -o Lyerbabuenae_ensambleFinal.fa -c Lyerbabuenae_scaffolding_scaffol
d.fa -IP1 ./Trimmed_R1.fastq  ./Trimmed_R2.fastq -t 40 2> Lyerbabuenae_gapclose.log
```

```
#Pilon
bwa index Lyerbabuenae_assemblyFinal500.fa

bwa mem -t 20 Lyerbabuenae_assemblyFinal500.fa Lyerbabuenae_R1.fastq Lyerbabuenae_R2.fas
tq > Lyerbabuenae.sam

samtools view -Su Lyerbabuenae.sam > Lyerbabuenae.bam

samtools sort Lyerbabuenae.bam -o Lyerbabuenae_sorted.bam

samtools index Lyerbabuenae_sorted.bam

java -Xmx250000m -jar pilon-1.21.jar --genome Lyerbabuenae_assemblyFinal500.fa --frags L
yerbabuenae.sorted.bam --output iteracion_1 --outdir salida_iteraciones --diploid --thre
ads 10 > log1_pilon
```

```
#MeDuSa

java -jar medusa.jar -f reference_genomes/ -i Lyerbabuenae.fasta -o Lyerbabuenae_medusa.
fna -threads 12 -v
```

Repeat identification and annotation

We used TEdenovo from REPET package to predict, identify and annotate the transposable elements using the repetitive elements database: Repbase, for the de novo genome assembly. We used the RepeatMasker v4.0.7 software (https://github.com/rmhubley/RepeatMasker (https://github.com/rmhubley/RepeatMasker)) to generated the masking of TEs across the genome. Repetitive elements were annotated by blastn against the complete virus database of nucleotide sequences from RefSeq. Additionally, we used the GyDB database to perform a second annotation by blastx.

```
#RepeatMasker

RepeatMasker -pa 10 -lib lyer_refTEs_v2.fa -x -gff Lyerbabuenae_medusa.fna
```

Transcriptomic data

We assembled the RNA-Seq data of five tissues (brain, pancreas, kidney, liver, and lung) with Trinity v4.4.7. The transcriptome assembly was cleaned with SeqClean and DeconSeq tool. We obtained the coding sequences (CDS) and their corresponding translation to proteins with the software AlingWise.

We performed the annotation by Blastp using the protein information from RefSeq database of the mammals: *Myotis brandtii* (ASM41265v1), *Myotis lucifugus* (Myoluc2.0), *Miniopterus natalensis* (Mnat.v1), *Rousettus aegyptiacus* (Raegyp2.0), *Pteropus vampyrus* (Pvam_2.0), *Cannis lupus familiaris* (CamFam3.1), *Equus caballus* (EquCab3.0), *Homo sapiens* (GRCh38.p12), and *Mus musculus* (GRCm38.p6).

```
cat BrainR1.fastq LiverR1.fastq KidneyR1.fastq PancreasR1.fastq LungR1.fastq > Lyerbabuena_R1.fastq

cat BrainR2.fastq LiverR2.fastq KidneyR2.fastq PancreasR2.fastq LungR2.fastq > Lyerbabuena_R2.fastq

time Trinity --seqType fq -max_memory 500G --left /home/ygutierrez/Assembly/TrinityAssembler/Lyerbabuena_R1.fastq --right /home/ygutierrez/Assembly/TrinityAssembler/Lyerbabuena_R2.fastq --CPU 20 --full_cleanup
```

Section SI 3

NW Leaf-Nosed bats reference genome assembly

Mapping and Variants identification For this analysis, we also included the raw genomic information of *D. rotundus* (accession number: SRA619672), and the Moormopidae: *Pteronotus parnelli* (accession number: SRR924359).

To identify Single-Nucleotide Variants (SNVs), all the high-quality genomic reads of each phyllosomid and moormopid bat were mapped to Lyerbabuena_v2 with Burrow-Wheller alignment (BWA-MEM) tool. We employed Picard (https://broadinstitute.github.io/picard/ (https://broadinstitute.github.io/picard/)) and GATK v2.07 (https://github.com/broadinstitute/gatk (https://github.com/broadinstitute/gatk)) pipeline and followed the GATK best practices and recommendations. We recalibrated the genomic alignment and identified the single nucleotide variants (SNVs) for each bat. With Picard, we used SortSam, MarkDuplicates, AddOrReplaceReadGroups, BuildBamIndex and CreateSequenceDictionary tools. With GATK we performed RealignerTargetCreator, IndelRealigner, HaplotypeCaller, VariantFiltration, SelectVariants, BaseRecalibrator, AnalyzeCovariates, PrintReads, and VariantFiltration.

```
#Mapping

bwa index Lyerbabuenae_medusa.fna
bwa mem -M Lyerbabuenae_medusa.fna Mwaterhousii_R1.fastq Mwaterhousii_R2.fastq -t 10 > align_Mwaterhousii.sam
bwa mem -M Lyerbabuenae_medusa.fna Lnivalis_R1.fastq Lnivalis_R2.fastq -t 10 > align_Lnivalis.sam
bwa mem -M Lyerbabuenae_medusa.fna Mharrisonii_R1.fastq Mharrisonii_R2.fastq -t 10 > align_Mharrisonii.sam
bwa mem -M Lyerbabuenae_medusa.fna Ajamaisensis_R1.fastq Ajamaisensis_R2.fastq -t 10 > align_Ajamaicensis.sam
bwa mem -M Lyerbabuenae_medusa.fna Pparnelli_R1.fastq Pparnelli_R2.fastq -t 10 > align_Pparnelli.sam

#-M flag causes BWA to mark shorter split hits as secondary (essential for Picard compatibility).

# SAM order

java -jar picard.jar SortSam I= align_Mwaterhousii.sam O= sorted_MwaterhousiiPE.bam SORT_ORDER=coordinate
java -jar picard.jar SortSam I= align_Lnivalis.sam O= sorted_LnivalisPE.bam SORT_ORDER=coordinate
java -jar picard.jar SortSam I= align_Mharrisonii.sam O= sorted_MharrisoniiPE.bam SORT_ORDER=coordinate
java -jar picard.jar SortSam I= align_Ajamaicensis.sam O= sorted_AjamaicensisPE.bam SORT_ORDER=coordinate
java -jar picard.jar SortSam I= align_Pparnelli.sam O= sorted_PparnelliPE.bam SORT_ORDER=coordinate

#Mark duplicates

java -jar picard.jar MarkDuplicates I= sorted_MwaterhousiiPE.bam O= dedup_Mwaterhousii.bam M= metrics_Mwaterhousii.txt MAX_FILE_HANDLES_FOR_READ_ENDS_MAP=1000
java -jar picard.jar MarkDuplicates I= sorted_LnivalisPE.bam O= dedup_Lnivalis.bam M= metrics_Lnivalis.txt MAX_FILE_HANDLES_FOR_READ_ENDS_MAP=1000
java -jar picard.jar MarkDuplicates I= sorted_MharrisoniiPE.bam O= dedup_Mharrisonii.bam M= metrics_Mharrisonii.txt MAX_FILE_HANDLES_FOR_READ_ENDS_MAP=1000
java -jar picard.jar MarkDuplicates I= sorted_AjamaicensisPE.bam O= dedup_Ajamaicensis.bam M= metrics_Ajamaicensis.txt MAX_FILE_HANDLES_FOR_READ_ENDS_MAP=1000
java -jar picard.jar MarkDuplicates I= sorted_PparnelliPE.bam O= dedup_Pparnelli.bam M= metrics_Pparnelli.txt MAX_FILE_HANDLES_FOR_READ_ENDS_MAP=1000

#AddOrReplaceReadGroups

java -jar picard.jar AddOrReplaceReadGroups I= dedup_Mwaterhousii.bam O= dedup2_Mwaterhousii.bam RGID=1 RGLB=lib1 RGPL=illumina RGPU=TAGCTT RGSM=Mwaterhousii
java -jar picard.jar AddOrReplaceReadGroups I= dedup_Lnivalis.bam O= dedup2_Lnivalis.bam RGID=1 RGLB=lib1 RGPL=illumina RGPU=ATGTCA RGSM=Lnivalis
java -jar picard.jar AddOrReplaceReadGroups I= dedup_Mharrisonii.bam O= dedup2_Mharrisonii.bam RGID=1 RGLB=lib1 RGPL=illumina RGPU=GGCTAC RGSM=Mharrisonii
java -jar picard.jar AddOrReplaceReadGroups I= dedup_Ajamaicensis.bam O= dedup2_Ajamaicensis.bam RGID=1 RGLB=lib1 RGPL=illumina RGPU=CACCGG RGSM=Ajamaicensis
```

```
java -jar picard.jar AddOrReplaceReadGroups I= dedup_Pparnelli.bam O= dedup2_Pparnelli.bam RGID=1 RGLB=lib1 RGPL=illumina RGPU=TAGCTTAT RGSM=Pparnelli


#BIULD INDEX

java -jar picard.jar BuildBamIndex I= dedup2_Mwaterhousii.bam
java -jar picard.jar BuildBamIndex I= dedup2_Lnivalis.bam
java -jar picard.jar BuildBamIndex I= dedup2_Mharrisonii.bam
java -jar picard.jar BuildBamIndex I= dedup2_Ajamaicensis.bam
java -jar picard.jar BuildBamIndex I= dedup2_Pparnelli.bam

#REALIGNMENT

samtools faidx Lyerbabuenae_medusa.fna

#Create list
java -jar GenomeAnalysisTK.jar -T RealignerTargetCreator -R Lyerbabuenae_medusa.fna -I dedup2_Mwaterhousii.bam -o realignment_targetsMwaterhousii.list
java -jar GenomeAnalysisTK.jar -T RealignerTargetCreator -R Lyerbabuenae_medusa.fna -I dedup2_Lnivalis.bam -o realignment_targetsLnivalis.list
java -jar GenomeAnalysisTK.jar -T RealignerTargetCreator -R Lyerbabuenae_medusa.fna -I dedup2_Mharrisonii.bam -o realignment_targetsMharrisonii.list
java -jar GenomeAnalysisTK.jar -T RealignerTargetCreator -R Lyerbabuenae_medusa.fna -I dedup2_Ajamaicensis.bam -o realignment_targetsAjamaicensis.list
java -jar GenomeAnalysisTK.jar -T RealignerTargetCreator -R Lyerbabuenae_medusa.fna -I dedup2_Pparnelli.bam -o realignment_targetsPparnelli.list

#Realign indels identified

java -jar GenomeAnalysisTK.jar -T IndelRealigner -R Lyerbabuenae_medusa.fna -I dedup2_Mwaterhousii.bam -targetIntervals realignment_targetsMwaterhousii.list -o realigned_readsMwaterhousii.bam
java -jar GenomeAnalysisTK.jar -T IndelRealigner -R Lyerbabuenae_medusa.fna -I dedup2_Lnivalis.bam -targetIntervals realignment_targetsLnivalis.list -o realigned_readsLnivalis.bam
java -jar GenomeAnalysisTK.jar -T IndelRealigner -R Lyerbabuenae_medusa.fna -I dedup2_Mharrisonii.bam -targetIntervals realignment_targetsMharrisonii.list -o realigned_readsMharrisonii.bam
java -jar GenomeAnalysisTK.jar -T IndelRealigner -R Lyerbabuenae_medusa.fna -I dedup2_Ajamaicensis.bam -targetIntervals realignment_targetsAjamaicensis.list -o realigned_readsAjamaicensis.bam
java -jar GenomeAnalysisTK.jar -T IndelRealigner -R Lyerbabuenae_medusa.fna -I dedup2_Pparnelli.bam -targetIntervals realignment_targetsPparnelli.list -o realigned_readsPparnelli.bam

java -jar picard.jar CreateSequenceDictionary R=Lyerbabuenae_medusa.fna O=Lyerbabuenae_medusa.fna.dict
java -jar GenomeAnalysisTK.jar -T HaplotypeCaller -R Lyerbabuenae_medusa.fna -I dedup2_Mwaterhousii.bam --genotyping_mode DISCOVERY -o raw_variantsMwaterhousii.vcf

#Variants identification
java -jar GenomeAnalysisTK.jar -T HaplotypeCaller -R Lyerbabuenae_medusa.fna -I realigned_readsMwaterhousii.bam -o raw_variantsMwa.vcf
```

```
java -jar GenomeAnalysisTK.jar -T HaplotypeCaller -R Lyerbabuenae_medusa.fna -I realigne
d_readsLnivalis.bam -o raw_variantsLni.vcf
java -jar GenomeAnalysisTK.jar -T HaplotypeCaller -R Lyerbabuenae_medusa.fna -I realigne
d_readsMharrisonii.bam -o raw_variantsMha.vcf
java -jar GenomeAnalysisTK.jar -T HaplotypeCaller -R Lyerbabuenae_medusa.fna -I realigne
d_readsAjamaicensis.bam -o raw_variantsAja.vcf
java -jar GenomeAnalysisTK.jar -T HaplotypeCaller -R Lyerbabuenae_medusa.fna -I realigne
d_readsPparnelli.bam -o raw_variantsPpa.vcf

#SNVs
java -jar GenomeAnalysisTK.jar -T VariantFiltration -R Lyerbabuenae_medusa.fna -V raw_va
riantsMwa.vcf --filterExpression 'QD < 2.0 || FS > 60.0 || MQ < 40.0 || MQRankSum < -12.
5 || ReadPosRankSum < -8.0 || SOR > 4.0' --filterName "basic_snp_filter" -o filtered_snp
sMwa.vcf
java -jar GenomeAnalysisTK.jar -T VariantFiltration -R Lyerbabuenae_medusa.fna -V raw_va
riantsLni.vcf --filterExpression 'QD < 2.0 || FS > 60.0 || MQ < 40.0 || MQRankSum < -12.
5 || ReadPosRankSum < -8.0 || SOR > 4.0' --filterName "basic_snp_filter" -o filtered_snp
sLni.vcf
java -jar GenomeAnalysisTK.jar -T VariantFiltration -R Lyerbabuenae_medusa.fna -V raw_va
riantsMha.vcf --filterExpression 'QD < 2.0 || FS > 60.0 || MQ < 40.0 || MQRankSum < -12.
5 || ReadPosRankSum < -8.0 || SOR > 4.0' --filterName "basic_snp_filter" -o filtered_snp
sMha.vcf
java -jar GenomeAnalysisTK.jar -T VariantFiltration -R Lyerbabuenae_medusa.fna -V raw_va
riantsAja.vcf --filterExpression 'QD < 2.0 || FS > 60.0 || MQ < 40.0 || MQRankSum < -12.
5 || ReadPosRankSum < -8.0 || SOR > 4.0' --filterName "basic_snp_filter" -o filtered_snp
sAja.vcf
java -jar GenomeAnalysisTK.jar -T VariantFiltration -R Lyerbabuenae_medusa.fna -V raw_va
riantsPpa.vcf --filterExpression 'QD < 2.0 || FS > 60.0 || MQ < 40.0 || MQRankSum < -12.
5 || ReadPosRankSum < -8.0 || SOR > 4.0' --filterName "basic_snp_filter" -o filtered_snp
sPpa.vcf

##INDELs

java -jar GenomeAnalysisTK.jar -T VariantFiltration -R Lyerbabuenae_medusa.fna -V raw_va
riantsMwa.vcf --filterExpression 'QD < 2.0 || FS > 200.0 || ReadPosRankSum < -20.0 || SO
R > 10.0' --filterName "basic_indel_filter" -o filtered_indelsMwa.vcf
java -jar GenomeAnalysisTK.jar -T VariantFiltration -R Lyerbabuenae_medusa.fna -V raw_va
riantsLni.vcf --filterExpression 'QD < 2.0 || FS > 200.0 || ReadPosRankSum < -20.0 || SO
R > 10.0' --filterName "basic_indel_filter" -o filtered_indelsLni.vcf
java -jar GenomeAnalysisTK.jar -T VariantFiltration -R Lyerbabuenae_medusa.fna -V raw_va
riantsMha.vcf --filterExpression 'QD < 2.0 || FS > 200.0 || ReadPosRankSum < -20.0 || SO
R > 10.0' --filterName "basic_indel_filter" -o filtered_indelsMha.vcf
java -jar GenomeAnalysisTK.jar -T VariantFiltration -R Lyerbabuenae_medusa.fna -V raw_va
riantsAja.vcf --filterExpression 'QD < 2.0 || FS > 200.0 || ReadPosRankSum < -20.0 || SO
R > 10.0' --filterName "basic_indel_filter" -o filtered_indelsAja.vcf
java -jar GenomeAnalysisTK.jar -T VariantFiltration -R Lyerbabuenae_medusa.fna -V raw_va
riantsPpa.vcf --filterExpression 'QD < 2.0 || FS > 200.0 || ReadPosRankSum < -20.0 || SO
R > 10.0' --filterName "basic_indel_filter" -o filtered_indelsPpa.vcf
```

Linked Desequilibrium sites

We used vcftool and PLINK to estimate linked disequilibrium sites (LD). For biallelic markers, we calculated the squared coefficient of correlation (r^2) within sliding windows of 5 Mbp. Additionally, we employed a filtering threshold on Minor Allele Frequency (MAF) of 0.05. We filtered the nucleotide variants that were in close proximity

(based on r^2 coefficient > 0.7) with VCFtools.

Genomic diversity

The percent of heterozygous and homozygous SNVs, and the nucleotide diversity measure were calculated across each phyllostomid vcf file, using sliding windows of 5 Mbp. We calculated the nucleotide diversity dividing the heterozygous single nucleotide variants by the genome length.

Reference genome assembly and annotation

With the GAKT output (BAM files recalibrated), we constructed the consensus sequence for: *Artibeus jamaicensis, Leptonycteris nivalis, Musonycteris harrisoni* and *Macrotus waterhousii* and the Moormopid: *Pteronotus parnelli* (SAMtools, BCFtools, vcfutils.pl and SeqtK tools) (https://samtools.github.io/bcftools/ (https://samtools.github.io/bcftools/)). For each consensus sequences in fasta format, the coding sequences regions and their corresponding protein translation were predicted with Augustus.

```
samtools mpileup -uf ../Lyerbabuenae_medusa.fna ../recalibration_readsMwaterhousii.bam |
bcftools call -c | vcfutils.pl vcf2fq -d 10 -D 100 > Mwaterhousii_consensus_recal.fq

samtools mpileup -uf ../Lyerbabuenae_medusa.fna ../recalibration_readsAjamaicensis.bam |
bcftools call -c | vcfutils.pl vcf2fq -d 10 -D 100 > Ajamaicensis_consensus_recal.fq

samtools mpileup -uf ../Lyerbabuenae_medusa.fna ../recalibration_readsMharrisonii.bam |
bcftools call -c | vcfutils.pl vcf2fq -d 10 -D 100 > Mharrisonii_consensus_recal.fq

samtools mpileup -uf ../Lyerbabuenae_medusa.fna ../recalibration_readsLnivalis.bam | bcf
tools call -c | vcfutils.pl vcf2fq -d 10 -D 100 > Lnivalis_consensus_recal.fq
```

We constructed a protein database including the UniProtKb/SwissProt, the protein sequences of *Leptonycteris yerbabueae*, and the sequences retrieved from RefSeq: *Desmodus rotundus, Eptesicus fuscus*, and *Miniopterus natalensis* bats. We used the database to performed the functional annotation by blastp for each proteomic file. Additionally, we performed InterProScan to annotated all the Phyllostomid proteins files.

Section SI 5

Phylogenomic history of New World (NW) Leaf-nosed bats

We reconstructed a phylogenomic tree based on 132 single-copy orthologous genes (61,331 amino acids sites), which was calibrated using several fossil dates. The divergence time between the Chiroptera order and the horse (as a sister taxon) was 63.19 million years ago (Mya) (60.27 – 64.47 Mya, 95% HPD). The NW Leaf-Nosed bats diverged in the transition of Eocene/Oligocene between 44 - 36.4 Mya (34.63 – 45.8 Mya, 95% HPD). Furthermore, nectar-feeding bats (Glossophaginae subfamily), evolved relatively recently in comparison to other nectarivorous vertebrates. The divergence of the glossophagini bats started in the Mid-Miocene from 21 to 7 Mya, coinciding with some environmental changes and the increase of food resources at the Climatic Optimum period.

```
prottest3 -i all_scp_aling.phy -o all_scp_aling_AIC_BIC.prottest -t1 -t2 -tc 0.8 -AIC -B
IC -S 2

PhyML3 -i alignment_sp_scg.phy -d aa -m JTT -c 4 -a e -boot_progress_display 1000000
```

Gene family analyses across Leaf-nosed bats lineages

We used CAFE, to analyse the statistical changes in the gene family sizes using a birth and death estimator (λ and μ). Based on the distribution of observed family sizes, we calculated the p-values for gene family expansion and contractions.

We estimated the birth-dead parameter (λ) to know the probability to any gene gain or lost across the phylogeny. The global-λ value = 0.00970874 was using to evaluate the expansions and contractions of families with large numbers of gene copies. Additionally, we tested two models global-λ and multiple-λ by a likelihood ratio test. The probability of global-λ is fitting by chance is higher than a multiple-λ for our data. Finally, we evaluated the error associated with the genome assembly and annotation over the gene family evolution. According with our results error in the genome assembly and annotation could inflate the rate of gene family evolution.

```
#CAFE

#!cafe

load -i filtered_cafe_input.txt -t 4 -l reports/log_run1.txt
tree (((Clupus:79.745528,Ecaballus:79.745528):4.77879,((Pvampyrus:24.694838,Raegyptiacu
s:24.694838):38.496546,((Ppar:44.885057,(Mwaterhousii:36.043301,(Drotundus:33.006268,(Aj
amaicensis:25.979402,(Mharrisoni:18.967049,(Lnivalis:7.863679,Lyerbabuenae:7.863679):11.
103369):7.012353):7.026867):3.037032):8.841756):13.409139,(Mnatalensis:52.952397,(Efus:4
5.838966,(Mbrandtii:20.648309,Mdavidii:20.648309):25.190657):7.11343):5.341799):4.8971
9):21.332933):14.835391,(Hsapiens:89.496652,Mmusculus:89.496652):9.863057);
lambda -s -t (((1,1)1,((1,1)1,((1,(1,(1,(1,(1,(1,1)1)1)1)1)1)1,(1,(1,(1,1)1)1)1)1)1)1,
(1,1)1)
report reports/report_run1

#Step 2

#!cafe

load -i large_filtered_cafe_input_cafe1.txt -t 15 -l reports/log_cafe1_run2.txt
tree ((Hsapiens:88.464846,(Clupus:76.756963,(Ecaballus:73.745437,((Pvampyrus:23.019102,R
aegyptiacus:23.019102):42.097932,((Pparnelli:38.979498,(Mwaterhousii:33.115163,(Drotundu
s:30.683813,(Ajamaicensis:26.275739,(Mharrisoni:21.576576,(Lnivalis:11.591220,Lyerbabuen
ae:11.591220):9.985356):4.699163):4.408074):2.431349):5.864335):17.594998,(Mnatalensis:4
7.881537,(Mbrandtii:20.547808,Mdavidii:20.547808):27.333729):8.692959):8.542538):8.62840
3):3.011526):11.707883):15.053281,Mmusculus:103.518127);
lambda -l 0.00970873786400 -t ((1,(1,(1,((1,1)1,((1,(1,(1,(1,(1,(1,1)1)1)1)1)1)1,(1,(1,
1)1)1)1)1)1)1)1,1)
report reports/report_cafe1_run2

#Step 3

#!cafe

load -i filtered_cafe_input.txt -t 4 -l reports/log_run3.txt
tree (((Clupus:79.745528,Ecaballus:79.745528):4.77879,((Pvampyrus:24.694838,Raegyptiacu
s:24.694838):38.496546,((Ppar:44.885057,(Mwaterhousii:36.043301,(Drotundus:33.006268,(Aj
amaicensis:25.979402,(Mharrisoni:18.967049,(Lnivalis:7.863679,Lyerbabuenae:7.863679):11.
103369):7.012353):7.026867):3.037032):8.841756):13.409139,(Mnatalensis:52.952397,(Efus:4
5.838966,(Mbrandtii:20.648309,Mdavidii:20.648309):25.190657):7.11343):5.341799):4.8971
9):21.332933):14.835391,(Hsapiens:89.496652,Mmusculus:89.496652):9.863057);
lambda -s -t (((4,4)4,((3,3)3,((2,(1,(1,(1,(1,(1,1)1)1)1)1)1)1,(2,(2,(2,2)2)2)2)2)2)2,
(4,4)4)
report reports/report_run3

#Step 4

#!cafe

load -i filtered_cafe_input.txt -t 4 -l reports/log_run4.txt
tree (((Clupus:79.745528,Ecaballus:79.745528):4.77879,((Pvampyrus:24.694838,Raegyptiacu
s:24.694838):38.496546,((Ppar:44.885057,(Mwaterhousii:36.043301,(Drotundus:33.006268,(Aj
amaicensis:25.979402,(Mharrisoni:18.967049,(Lnivalis:7.863679,Lyerbabuenae:7.863679):11.
```

```
103369):7.012353):7.026867):3.037032):8.841756):13.409139,(Mnatalensis:52.952397,(Efus:4
5.838966,(Mbrandtii:20.648309,Mdavidii:20.648309):25.190657):7.11343):5.341799):4.8971
9):21.332933):14.835391,(Hsapiens:89.496652,Mmusculus:89.496652):9.863057);
lambda -l 0.0109298
genfamily tutorial_genfamily/rnd -t 100
lhtest -d tutorial_genfamily -t (((4,4)4,((3,3)3,((2,(1,(1,(1,(1,(1,1)1)1)1)1)1)1,(2,(2,
(2,2)2)2)2)2)2)2,(4,4)4) -l  0.0109298 -o reports/lhtest_result.txt

#Step 5

#!cafe

load -i filtered_cafe_input.txt -t 4 -l reports/log_run5.txt
tree (((Clupus:79.745528,Ecaballus:79.745528):4.77879,((Pvampyrus:24.694838,Raegyptiacu
s:24.694838):38.496546,((Ppar:44.885057,(Mwaterhousii:36.043301,(Drotundus:33.006268,(Aj
amaicensis:25.979402,(Mharrisoni:18.967049,(Lnivalis:7.863679,Lyerbabuenae:7.863679):11.
103369):7.012353):7.026867):3.037032):8.841756):13.409139,(Mnatalensis:52.952397,(Efus:4
5.838966,(Mbrandtii:20.648309,Mdavidii:20.648309):25.190657):7.11343):5.341799):4.8971
9):21.332933):14.835391,(Hsapiens:89.496652,Mmusculus:89.496652):9.863057);
lambdamu -s -t (((1,1)1,((1,1)1,((1,(1,(1,(1,(1,(1,1)1)1)1)1)1)1,(1,(1,(1,1)1)1)1)1)1)1,
(1,1)1)
report reports/report_run5

#Step 6

#!cafe

load -i filtered_cafe_input.txt -t 4 -l reports/log_run6.txt
tree (((Clupus:79.745528,Ecaballus:79.745528):4.77879,((Pvampyrus:24.694838,Raegyptiacu
s:24.694838):38.496546,((Ppar:44.885057,(Mwaterhousii:36.043301,(Drotundus:33.006268,(Aj
amaicensis:25.979402,(Mharrisoni:18.967049,(Lnivalis:7.863679,Lyerbabuenae:7.863679):11.
103369):7.012353):7.026867):3.037032):8.841756):13.409139,(Mnatalensis:52.952397,(Efus:4
5.838966,(Mbrandtii:20.648309,Mdavidii:20.648309):25.190657):7.11343):5.341799):4.8971
9):21.332933):14.835391,(Hsapiens:89.496652,Mmusculus:89.496652):9.863057);
lambda -s -t (((1,1)1,((1,1)1,((1,(1,(1,(1,(1,(1,1)1)1)1)1)1)1,(1,(1,(1,1)1)1)1)1)1)1,
(1,1)1)
report reports/report_run6

#Step 7

#!/bin/bash

python caferror.py -i cafe_run6.sh -d reports/run6_caferror_files -v 0 -f 1

#Step 8

#!cafe

load -i filtered_cafe_input.txt -t 4 -l reports/log_run7.txt
tree (((Clupus:79.745528,Ecaballus:79.745528):4.77879,((Pvampyrus:24.694838,Raegyptiacu
s:24.694838):38.496546,((Ppar:44.885057,(Mwaterhousii:36.043301,(Drotundus:33.006268,(Aj
amaicensis:25.979402,(Mharrisoni:18.967049,(Lnivalis:7.863679,Lyerbabuenae:7.863679):11.
103369):7.012353):7.026867):3.037032):8.841756):13.409139,(Mnatalensis:52.952397,(Efus:4
5.838966,(Mbrandtii:20.648309,Mdavidii:20.648309):25.190657):7.11343):5.341799):4.8971
```

```
9):21.332933):14.835391,(Hsapiens:89.496652,Mmusculus:89.496652):9.863057);
errormodel -model reports/run6_caferror_files/cafe_errormodel_0.161865234375.txt -all
lambda -s -t (((4,4)4,((3,3)3,((2,(1,(1,(1,(1,(1,1)1)1)1)1)1)1,(2,(2,(2,2)2)2)2)2)2)2,
(4,4)4)
report reports/report_run7
```

Section SI 5

dN/dS analysis using a branch-site model

Orthologous single copy genes and filtering

Based on a multi-species genome comparison with this database, we inferred the orthologous single copy genes using Diamond and two different programs Proteinortho and OrthoVenn.

Each cluster were aligned with MAFFT aligner tool and poorly aligned regions were removed. We used the alignments and their corresponding coding sequences to perform a robust conversion of protein multi alignment into their corresponding codon alignments with PAL2NAL. We reconstructed the phylogenetic tree for each cluster with RaxML tool (parameters -m GTRGAMMA -p 12345).

```
ls *_seq.faa > test.txt

cat test.txt | while read seq ; do

        mafft --anysymbol --parttree --retree 1 --thread 8 ${seq} > alin_${seq}

    perl pal2nal.pl alin_${seq} ${seq}.fasta -output paml -nogap -codontable 1 > paml_
${seq}.fasta

    raxmlHPC-PTHREADS -f a -p 12345 -m PROTGAMMAAUTO -s alin_${seq} -n ${seq}.tree -p 12
345 -# 10000 -T 10 -x 10000

done
```

dN/dS Test

CODEML, we used a branch-site model, specifying the foreground branch (the phyllostomid species of our interest) and incorporating a null model. For assigning significance, we constructed the Likelihood Ratio Test (LRT), using the likelihood values from the null and test model, and calculated the *p-value* =< 0.05 under a chi-square distribution. We considered under positive selection all those sequence sites with a posterior probability > 95% (by Bayes Empirical Bayes method).

Test model parameters: model = 2; fix_omega = 0; omega = 1 Null model parameters: model = 2; fix_omega = 1; omega = 1

```
#CODEML test model

ls *.fasta > list_paml

cat list_paml | while read paml; do

    sed -r "s/[0-9]+.fasta/${paml}/g" codeml_atl.ctl | sed -r "s/[0-9]+.tree/${paml}.tree/g"  | sed 's/.fasta.tree/.tree/g' | sed -r "s/[0-9]+_alt.mcl/${paml}_alt.mcl/g" | sed 's/.fasta_alt.mcl/_alt.mcl/g' > codeml_${paml}_alt.ctl
    codeml codeml_${paml}_alt.ctl
    grep "lnL(ntime:" ${paml}_alt.mcl | sed "s/lnL/${paml}\tlnL/g" >> Paml_alt_out
    rm codeml_${paml}_alt.ctl

done

mv *.mcl Codeml_out/
```

```
#CODEML Null model

ls *.tree > list_tree

mkdir Paml_null

cd Paml_null/
touch Paml_null_out
mkdir Codeml_null_out
cp ../../codeml.ctl .

ln -s ../../genes/* .

cat ../list_tree | while read tree; do

    ln -s ../$tree .

done

rm ../list_tree

cat ../list_paml | while read paml; do

    sed -r "s/[0-9]+.fasta/${paml}/g" codeml.ctl | sed -r "s/[0-9]+.tree/${paml}.tree/g" | sed 's/.fasta.tree/.tree/g' | sed -r "s/[0-9]+_null.mcl/${paml}_null.mcl/g" | sed 's/.fasta_null.mcl/_null.mcl/g'  > codeml_${paml}_null.ctl

    codeml codeml_${paml}_null.ctl

    grep "lnL(ntime:" ${paml}_null.mcl | sed "s/lnL/${paml}\tlnL/g" >> Paml_null_out

    mv ${paml}_null.mcl Codeml_null_out/

done
```

```
#Likelihood Ratio Test (LRT) construction

cd Codeml_out/
grep "lnL(ntime:" *mcl | sed 's/_alt.mcl://g' | sed "s/lnL/\tlnL/g" >> ../Paml_alt_out

cd Paml_null/
grep "lnL(ntime:" *.mcl | sed 's/_null.mcl://g' | sed "s/lnL/\tlnL/g" >> ../Paml_null_ou
t

paste -d "\t" Paml_alt_out Paml_null_out | awk '{print$1"\t"$5"\t"$6"\t"$12"\t"$13}' | a
wk '{print$1"\t"$3"\t"$5}' > paml_lnL.txt
```

```
#setwd(Dir="")
lnl <- read.table(file = "paml_lnL.txt", header = FALSE)
lnl$Ln <- lnl$V2-lnl$V3
lnl$LRT <- lnl$Ln*2
write.table(lnl, file="1_LTR")
```

```
# Chi square

awk '{print$NF}' 1_LTR | sed -s '1d' > list_pval

cat list_pval | while read pval ; do

    chi2 1 $pval | sed -e "s/df/$pval\tdf/g" >> result_pval.txt

done

awk '{print$NF"\t"$2}' 1_LTR | sed '1d' | sed 's/"//g' > list_name

awk 'NR == FNR{a[$1] = $0;next}; {print $1, $1 in a?a[$1]: "NA"}'  result_pval.txt list_
pval | grep -v "NA" | grep -v "prob = 1.000000000" | grep -v "invalid" |  awk '{$1=""}1'
| sed 's/^.//g' > pval_paml.txt

awk 'NR == FNR{a[$1] = $0;next}; {print $1, $1 in a?a[$1]: "NA"}' list_name pval_paml.tx
t | awk '{print$NF}' > list_seq

awk '{print$NF}' 1_LTR | sed -s '1d' > list_pval

cat list_pval | while read pval ; do

    chi2 1 $pval | sed -e "s/df/$pval\tdf/g" >> result_pval.txt

done

awk '{print$NF"\t"$2}' 1_LTR | sed '1d' | sed 's/"//g' > list_name

awk 'NR == FNR{a[$1] = $0;next}; {print $1, $1 in a?a[$1]: "NA"}'  result_pval.txt list_
pval | grep -v "NA" | grep -v "prob = 1.000000000" | grep -v "invalid" |  awk '{$1=""}1'
| sed 's/^.//g' > pval_paml.txt

awk 'NR == FNR{a[$1] = $0;next}; {print $1, $1 in a?a[$1]: "NA"}' list_name pval_paml.tx
t | awk '{print$NF}' > list_seq

paste -d "\t" list_seq pval_paml.txt | sed 's/df/\tdf/g' | sed 's/1 prob/\t1 prob/g' | a
wk '{print$1"\t"$2"\t"$NF}' > codeml_out_pval.txt
```

```
#False Discovery Rate

code <- read.table(file="codeml_out_pval.txt", header = FALSE, sep = "\t")

code$FDR <- p.adjust(code$V3, method = "fdr")

write.table(code, file="FDR_codeml_pval.txt", quote=FALSE)
```

```
#Filter by pvalue

cat FDR_codeml_pval.txt | awk '{if ($4<=0.05) print$0}' > PositiveSel_genes_pval.txt
```

```
#Sites under positive selection based on Bayesian Empirical Probability

awk '{print$1}' PositiveSel_genes_pval.txt | sed 's/$/_alt.mcl/g' > list.mcl

cd Codeml_out/
cat ../list.mcl | while read beb ; do

    awk '/Bayes Empirical Bayes/,/The grid/' $beb | grep "*" | perl -pe 's/\n/, / unless eof' | sed "s/^./$beb /" >> ../../PositiveSelec_genes_BEB.txt
```

Hyphy, we used aBSREL (adaptive Branch-Site Random Effects Likelihood).

```
#HYPHY

#!/bin/bash
#Dependencies: HyPhy
#USAGE: ./run_busted

for abs in $(cat list_scg);
do

  aBSREL.sh genes/nuc_genes/${abs}.fasta genes/phylo_genes/${abs}.nwk > genes_batout/${abs}.bf

  mpiexec -n 10 HYPHYMP genes_batout/${abs}.bf  > aBSREL_genes/${abs}_aBSREL.res

  mpiexec -n 10 HYPHYMP absrel --alignment scg/nuc_scg/${abs}.fasta --tree scg/tree_scg/${abs}.nwk --output aBSREL_scg/${abs}.absrel.res > ${abs}.out

done
```

Section SI 6

Radical amino-acid substitution in conserved positions

Each single copy gene cluster was aligned using PRANK.We constructed their corresponding phylogenetic tree with RAxML (parameter -m PROTCATLG). To accurate the ancestral sequence, we used two different programs:

CODEML: assumes a Markov process model and calculates a bayesian empirical likelihood for each character at each sequence position.

FastML: assumes a continuous time Markov process model and provides the posterior probabilities for each character at each sequence position

For CODEML, we fixed the parameters: model =2, fix_alpha = 0, alpha = 0.5 and RateAncestor = 1.

```
#Scripts to identify parallel changes

#CODEML

#!/bin/bash
mkdir evol_rates
mkdir ancestral_seq

ls *.faa > list_seq

cat list_seq | while read paml; do

    sed -r "s/[a-z]+.faa/${paml}/g" codeml.ctl | sed -r "s/[a-z]+.tree/${paml}.tree/g" | sed 's/.faa.tree/.tree/g' | sed -r "s/[a-z]+.mcl/${paml}.mcl/g" | sed 's/.faa.mcl/.mcl/g' > codeml_${paml}.ctl
    codeml codeml_${paml}.ctl
    mv rst rst_${paml}
done

mv *.mcl evol_rates/
mv rst* ancestral_seq/
```

```
#Look into rst file (ancestral reconstruction)
#!/bin/bash

ls rst* > ancestral.list

cat ancestral.list | while read anc ;
do
    awk '/List of extant and reconstructed sequences/,/Overall accuracy/' ${anc} | sed '1,4d' | sed '$d' | sed '$d' | sed '$d' | awk '{gsub("node #" , "node")}1' > reconstruct.txt
    sed -i 's/^/>/g' reconstruct.txt
    grep -E '>Mha|>Lye|>Lni|>Pal|>PVA|>Rae' reconstruct.txt | awk '{print$1}' | sed 's/>//g' > id_nect
    grep -Ev '>Mha|>Lye|>Lni|>Pal|>PVA|>Rae|>node' reconstruct.txt | awk '{print$1}' | sed 's/>//g' > id_out
    python ../extract_seq.py reconstruct.txt id_nect
    mv output.fasta nectar.fasta
    python ../extract_seq.py reconstruct.txt id_out
    cat output.fasta >> nectar.fasta
    rm output.fasta id_out reconstruct.txt
    sed -i 's/>//g' nectar.fasta
    awk '{$1=""; print$0}' nectar.fasta | sed -e 's/\(.\)/\1 /g' | sed 's/^..//g' > temp_recons.txt
    awk '{print$1}' nectar.fasta | awk -F"_" '{print$1}' > id_temp
    paste -d "\t" id_temp temp_recons.txt > ${anc}_reconstruct_table.txt
    rm id_nect id_temp nectar.fasta temp_recons.txt
    ls *table* > list.txt
    ./traspose.sh
    rm reconstruct_table.txt
done
```

```
# Transpose script "transpose.sh"

#!/bin/bash

cat list.txt | while read t ; do

        awk '
        {
                for (i=1; i<=NF; i++) {
                        a[NR,i] = $i
                }
        }
        NF>p { p = NF }
        END {
                for(j=1; j<=p; j++) {
                        str=a[1,j]
                        for(i=2; i<=NR; i++){
                                str=str" "a[i,j];
                        }
                        print str
                }
        }' ${t} > t_${t}

done
```

```
#Identify parallel sites (including ancestral resconstruction state in internal nodes)

#!/bin/bash

ls t_* > list_t.txt

cat list_t.txt | while read p ; do

        awk '{for (i=2; i<=3; i++) {if ($1!=$i){ next}}; for (i=4; i<=NF; i++) {if ($1==
$i) {next}}};1' t_reconstruct_table.txt $p > ${p}.txt
done
```

References

1. Kaijitani R, Toshimoto K, Noguchi H, Toyoda A, Ogura Y, et al. Efficient de novo assembly of highly heterozygous genomes from whole-genome shotgun short reads. Genome Res. 2014;24:1384–1395.

2. Lowe T. tRNAscan-SE: a program for improved detection of transfer RNA genes in genomic sequence. Nucleic Acids Res. 1997;25:955–964.

3. Bosi E, Donati B, Galardini M, Brunetti S, Sagot MF, et al. MeDuSa: A multi-draft based scaffolder. Bioinformatics. 2015;31:2443–2451.

4. Walker BJ, Abeel T, Shea T, Priest M, Abouelliel A, et al. Pilon: An integrated tool for comprehensive microbial variant detection and genome assembly improvement. PLoS One. 2014;0112963.

5. Simão FA, Waterhouse RM, Ioannidis P, Kriventseva EV, Zdobnov EM. BUSCO: Assessing genome assembly and annotation completeness with single-copy orthologs. Bioinformatics. 2015;31:3210–3212.

6. Flutre T, Duprat E, Feuillet C, Quesneville H. Considering transposable element diversification in de novo annotation approaches. PLoS One. 2011;6:0016526.

7. Bao W, Kojima KK, Kohany O. Repbase Update, a database of repetitive elements in eukaryotic genomes. Mob DNA. 2015;6:4–9.

8. Tarailo-Graovac, Chen N. Using RpeatMasker to identify repetitive elements in genomic sequences. Curr Protoc Bioinformatics. 2009;4:bi0410s25.

9. Haas BJ, Papanicolaou A, Yassour M, Grabherr M, Blood PD, et al. De novo transcript sequence reconstruction from RNA-seq using the Trinity platform for reference generation and analysis. Nat Protoc. 2013;8:1494–1512.

10. Evans T, Loose M. AlignWise: a tool for identifying protein-coding sequence and correcting frame-shifts. BMC Bioinformatics. 2015;1:376.

11. Stanke M, Morgenstern B. AUGUSTUS: A web server for gene prediction in eukaryotes that allows user-defined constraints. Nucleic Acids Res. 2005;33:465–467.

12. The UniProt Consortium. UniProt: The universal protein knowledgebase. Nucleic Acids Res. 2018;46:2699.

13. Jones P, Binns D, Chang HY, Fraser M, Li W, et al., InterProScan 5: Genome-scale protein function classification. Bioinformatics. 2014;30:1236–1240.

14. Van der Auwera GA, Carneiro MO, Hartl C, Poplin R, Del Angel G, et al. From fastQ data to high-confidence variant calls: The genome analysis toolkit best practices pipeline. Curr Protoc Bioinformatics. 2013;43:11.10.1-33.

15. Li H, Durbin R. Fast and accurate short read alignment with Burrows-Wheeler transform. Bioinformatics. 2009;14:1754-1760.

16. Broad Institute. Picard Tools. Broad Institute, Github repository. https://github.com/broadinstitute/picard/issues/808 (https://github.com/broadinstitute/picard/issues/808)

17. Li H, Handsaker B, Wysoker A, Fennell T, Ruan J, et al. The Sequence Alignment/Map format and SAMtools. Bioinformatics. 2009;16:2078–2079.

18. Danecek P, Auton A, Abecasis G, Alberts CA, Banks E, et al. The variant call format and VCFtools. Bioinformatics. 2011;15:2156–2158.

19. Quinlan AR, Hall IM. BEDTools: A flexible suite of utilities for comparing genomic features. Bioinformatics. 2010;6:841–842.

20. Yang Z. PAML 4 : Phylogenetic Analysis by Maximum Likelihood. Mol Biol Evol. 2007;24:1586–1591.

21. De Bie T, Cristianini N, Demuth JP, Hahn MW. CAFE: A computational tool for the study of gene family evolution. Bioinformatics. 2006;22:1269–1271.

22. Wang Y, Coleman-Derr D, Chen G, Gu YQ. OrthoVenn: a web server for genome wide comparison and annotation of orthologous clusters across multiple species. Nucleic Acids Res. 2015;43:W78-W84.

23. Lechner M, Findeib SS, Steiner L, Marz M, Stadler PF. Proteinortho: detection of (co-)orthologs in large-scale analysis. BMC Bioinformatics. 2011;12:124.

24. Katoh K, Misawa K, Kuma K, Miyata T. MAFFT: a novel method for rapid multiple sequence alignment based on fast Fourier transform. Nucleic Acids Res. 2002;14:3059-3066.

25. Suyama M, Torrents D, Bork P. PAL2NAL: robust conversion of protein sequence alignments into the corresponding codon alignments. Nucleic Acids Res. 2006;34:W609-W612.

26. Stamatakis A. RaxML version 8: a tool for phylogenetic analysis and post-analysis of large phylogenies. Bioinformatics. 2014;30:1312-1313.

27. Pond SL, Frost SD, Muse SV. HyPhy: hypothesis testing using phylogenies. Bioinformatics. 2005;5:676-9.

28. Smith MD, Wertheim JO, Weaver S, Murrell B, Scheffler K. et al. Less is More: An adaptive branch-site random effects model for efficient detection of episodic diversifying selection. Mol Biol Evol. 2015;5:132-1353.

29. Löytynoja A. Phylogeny-aware alignment with PRANK. Methods Mol Biol. 2014;1079:155-70.

30. Ashkenazy H, Penn O, Doron-Faigenboim A, Cohen O, Cannarozzi G, et al. FastML: a web server for probabilistic reconstruction of ancestral sequences. Nucleic Acids Res. 2012;40:W580-4.
