# Supplementary material for: Genomic consequences of dietary diversification and parallel evolution due to nectarivory in leaf-nosed bats
Source: Gigascience. 2020 Jun 6;9(6):giaa059. doi: 10.1093/gigascience/giaa059 (PMC7276932; doi:10.1093/gigascience/giaa059)
Supplement: giaa059_Supplemental_Files [file giaa059_supplemental_files.zip › Additional_File1.pdf]

Genomic consequences of dietary diversification and parallel evolution due to nectarivory in Leaf-nosed bats

Yocelyn T. Gutiérrez-Guerrero[1], Enrique Ibarra-Laclette[2], Carlos Martínez del Río[3], Josué Barrera-Redondo[1], Eria A. Rebollar[4], Jorge Ortega[5], Livia León-Paniagua[6], Araxi Urrutia[7], Erika Aguirre-Planter[1] and Luis E. Eguiarte*[1]

Additional File 1.

Raw Data Genome and RNA sequencing

Illumina HiSeq 4000 150PE

Specie	Sequence	Tissue	Total reads	Reads PHRED >30	Single-End (R1-R2)
L. yerbabuenae	Genome	Muscle	697032826	690759531(99.1%)	125695210 (18%)
	Transcriptome	Brain	57843563	57695949 (99.97%)	41191232 (71.3%)
	Transcriptome	Pancreas	67115811	67027977(99.86%)	55525185 (82.8%)
	Transcriptome	Kidney	69843684	69697427 (99.79%)	53201672 (76.3%)
	Transcriptome	Lung	58711578	58556306 (99.74%)	42000519 (71.7%)
	Transcriptome	Liver	60067046	59990327 (99.87%)	49829078 (83.1%)

Specie	Sequence	Tissue	Total reads	Reads PHRED >30
L. nivalis	Genome	Muscle	365708658	346437915 (94.7%)
M. harrisonii	Genome	Muscle	203048998	192616065 (94.8%)
A. jamaisensis	Genome	Muscle	165368915	155925581(94.2%)
M. waterhousii	Genome	Muscle	375623209	357259663 (95.11%)

MUSEUM DATA

Specie	Colect	#Catalog	Locality
L. nivalis	005TCM	6432	Hidalgo, San Agustin Tlaxiaca, San Francisco Tecajique
M. harrisonii	GHC216	16153	Guerrero, Tecpan de Galeana, Palma del Cayaco
A. jamaisensis	GHC051	12380	Chiapas, Cacahoatan, La Unión
M. waterhousii	GHC033	12465	Guerrero,Taxco de Alarcón, Jale Minero “El Fraile”

L. yerbabuenae: *De Novo* genome assembly statistics

Genome assembly metrics for first (Lyerbauenae_v1) and second (Lyerbabuena_v2) assembly

	***Leptonycteris yerbabuenae* assembly v1**			***Leptonycteris yerbabuenae* assembly v2**	
	**Contig**	**Scaffold**	**GapClosing**	**Contig**	**Scaffold**
**Total size length**	2325728430	2130311516	2130088700	2050309067	2053581484
**Number**	2011529	686214	686214	78626	34419
**N50**	7723	73651	73623	69496	14735151
**L50**	82448	7760	7763	8805	38

BUSCO Summarized

**Genome**	**Total BUSCO**	**Complete**	**Complete – S**	**Complete – D**	**Fragmented**	**Missing**
Lyerbabuenaev1	4104	3132(76.3%)	3108(75.7%)	24(0.6%)	595(14.5%)	377(9.2%)
Lyerbabuenaev2	4104	3860 (94.1%)	3837 (93.5%)	23(0.6%)	103(2.5%)	141(3.4%)

Augustus Gene Prediction

Training Set	CDS	Gene	Intron	Start	Stop
L. yerbabuenae (self trainir	124479	21971	103228	21530	21558

TE prediction and annotation

**Specie**	**Total TE leng**	**Seq Putative Potential Host**	**Seq TE Anno**	**% TE in Genome**	
Lyerbabuenae	9966767 bp	229	327	26.00%	55626.00%
		229	327	26.00%	

ELEMENT ANNOTATED

MATCH	FAMILY	SYSTEM	Class
ASSBSV	Retroviridae	LTR retroelements	Class 1
BAEVM	Retroviridae	LTR retroelements	Class 1
CAEV	Retroviridae	LTR retroelements	Class 2
CfSCAND3	Element	Unclassified	
FeLV	Retroviridae	LTR retroelements	Class 1
GALV	Retroviridae	LTR retroelements	Class 1
HERV-E	Retroviridae	LTR retroelements	Class 1
HERV-K10	Retroviridae	LTR retroelements	Class 2
HFV	Retroviridae	LTR retroelements	Class 3
HTLV-2	Retroviridae	LTR retroelements	Class 2
JSRV	Retroviridae	LTR retroelements	Class 2
K-HERV	Retroviridae	LTR retroelements	Class 2
KoRV	Retroviridae	LTR retroelements	Class 1
LPDV	Retroviridae	LTR retroelements	Class 2
MdEV	Retroviridae	LTR retroelements	Class 1
MMTV	Retroviridae	LTR retroelements	Class 2
MPMV	Retroviridae	LTR retroelements	Class 2
MuLV	Retroviridae	LTR retroelements	Class 1
PERV-MSL	Retroviridae	LTR retroelements	Class 1
PvSCAND3	Element	Unclassified	
PyERV	Retroviridae	LTR retroelements	Class 2
RCHO-K1	Retroviridae	LTR retroelements	Class 1
REV	Retroviridae	LTR retroelements	Class 1
RSV	Retroviridae	LTR retroelements	Class 2
RTVL-Ia	Retroviridae	LTR retroelements	Class 1
SERV	Retroviridae	LTR retroelements	Class 2
SMRV-H	Retroviridae	LTR retroelements	Class 2
SRV-1	Retroviridae	LTR retroelements	Class 2
VMV	Retroviridae	LTR retroelements	Class 2
WdSV	Retroviridae	LTR retroelements	Class 1
XEN1	Retroviridae	LTR retroelements	Class 1
Yoyo	*Ty3/Gypsy*	LTR retroelements	Branch 2
ZFERV	Retroviridae	LTR retroelements	Class 1
ZFERV-2	Retroviridae	LTR retroelements	Class 2

Single Nucleotide Variants and Indels Metrics

SNVs and INDELs identified for each leaf-nosed bat and P. parnelli (mapped to reference genome Lyerbabuenae_2)

Specie	No. reads mapp	No. Bp	SNPS	SNP Heterozy	SNP (½)	SNP Homozygous	Rate He/Ho
L. nivalis	687389680	2051172625	29678506	6485536	72127	23121294	0.283583
M. harrisonii	666527225	2045633862	105386551	2401366	83033	102882877	0.0241433
A. jamaicensis	290391248	2043298805	117591198	8766294	443558	108363473	0.0849764
D. rotundus	2347045591	2047984644	113437016	8091088	445181	104891464	0.0813747
M. waterhousii	666527225	2040084780	112514030	2704911	145943	109656595	0.0246671073

Specie	P_He(1/2)	P_He	P_Ho	Mean Sliding windows 1Mb
L. nivalis	0.24302773192	8.18868E-07	77.9058555	0.019
M. harrisonii	0.07878899083	7.47619E-08	97.62429458	0.0032
A. jamaicensis	0.37720340259	3.20775E-07	92.15270772	0.003
D. rotundus	0.39244773505	3.45961E-07	92.46669888	0.0024
M. waterhousii	0.12971093472	1.15284E-07	97.4603745	0.0012

Genome-guide statistics for each NW Leaf-nosed bat

Genomic consensus sequence based on L.yerbabuenae reference-genome

Specie	No. contigs	CDS	Proteins	Proteins >30aa	Blastp	InterProScan	KEGG
L. yerbabuenae						16261	14569
L. nivalis	34031	84094	84094	66003	28748	22819	13020
M. harrisonii	32288	71229	71229	51292	20831	16849	11855
A. jamaicensis	31503	57748	57748	35730	17213	14094	10674
M. waterhousii	30112	59039	59039	39447	17805	14559	11295

LRT construction and *p-value* correction for sensory genes and metabolic enzymes

Gene	Specie	LRT	*p-value*	FDR	
acecoa	*A. jamaicensis*	46.46467	9.329E-12	8.3E-11	*
acecoa	*M. harrisoni*	11.45761	0.000712	0.00132	*
acecoa	*L. nivalis*	60.11579	8.993E-15	2E-13	*
acecoa	*L. yerbabuenae*	34.78877	3.675E-09	1.72E-08	*
aldob	*A. jamaicensis*	60.58401	7.105E-15	2E-13	*
aldob	*L. nivalis*	0.120858	0.7281	0.94533	
alkphos	*M. waterhousii*	6E-06	0.998	0.9984	
alkphos	*D. rotundus*	8.36814	0.003819	0.00607	*
alkphos	*M. harrisoni*	24.58403	7.114E-07	2.34E-06	*
alkphos	*L. yerbabuenae*	16.00388	6.321E-05	0.00014	*
apoa2	*M. waterhousii*	38.38333	5.813E-10	3.45E-09	*
apoa2	*D. rotundus*	37.48247	9.224E-10	5.13E-09	*
apoa2	*A. jamaicensis*	23.08263	1.552E-06	4.76E-06	*
apoa2	*M. harrisoni*	52.73992	3.808E-13	4.84E-12	*
apoa2	*L. yerbabuenae*	21.01473	4.558E-06	1.27E-05	*
b-hydro	*M. waterhousii*	26.87043	2.176E-07	8.8E-07	*
b-hydro	*D. rotundus*	8.952286	0.002771	0.00484	*
b-hydro	*A. jamaicensis*	5.659676	0.01736	0.02452	
b-hydro	*L. yerbabuenae*	0.075916	0.7829	0.96775	
chia	*M. waterhousii*	3.44952	0.06327	0.08798	
chia	*M. harrisoni*	5.829654	0.01576	0.02262	
chitinased	*M. waterhousii*	72.4141	0	0	*
chitinased	*D. rotundus*	12.25654	0.0004636	0.0009	*
chitinased	*A. jamaicensis*	22.3227	2.305E-06	6.62E-06	*
chitinased	*L. nivalis*	4E-06	0.9984	0.9984	
chitinased	*L. yerbabuenae*	47.76433	4.807E-12	4.75E-11	*
citrate	*M. waterhousii*	6.44388	0.01113	0.01651	*
citrate	*D. rotundus*	6.590114	0.01025	0.01546	*
citrate	*L. nivalis*	9.82998	0.001717	0.00306	*
citrate	*L. yerbabuenae*	0.000444	0.9832	0.9984	
fbp1	*M. waterhousii*	17.5987	2.728E-05	6.94E-05	*
fbp1	*L. yerbabuenae*	4E-06	0.9984	0.9984	
fbp1	*L. yerbabuenae*	4E-06	0.9984	0.9984	
frucliv	*A. jamaicensis*	34.64849	3.949E-09	1.76E-08	*
frucmus	*M. waterhousii*	0.09753	0.7548	0.94616	
gfod1	*D. rotundus*	8.174652	0.004248	0.00663	*
glucogen	*M. waterhousii*	11.53642	0.0006825	0.00129	*
glucogen	*D. rotundus*	24.96057	5.851E-07	2.08E-06	*
glucogen	*A. jamaicensis*	8.546818	0.003461	0.0057	*
glucogen	*M. harrisoni*	8.780016	0.003045	0.00521	*
glut1	*M. waterhousii*	13.59617	0.0002266	0.00045	*

glut1	*M. harrisoni*	17.28703	3.214E-05	7.95E-05	*
glut1	*L. yerbabuenae*	1.2E-05	0.9972	0.9984	
glut1	*L. yerbabuenae*	1.2E-05	0.9972	0.9984	
glut2	*M. waterhousii*	16.58112	4.661E-05	0.0001	*
glut2	*L. nivalis*	2.2E-05	0.9963	0.9984	
glut2	*L. yerbabuenae*	3E-05	0.9956	0.9984	
glut3	*M. waterhousii*	6E-06	0.998	0.9984	
glut3	*D. rotundus*	4E-06	0.9984	0.9984	
glut3	*M. harrisoni*	4E-06	0.9984	0.9984	
glut4	*M. waterhousii*	38.47176	5.555E-10	3.45E-09	*
glut4	*M. harrisoni*	16.79426	4.166E-05	9.76E-05	*
glut5	*M. waterhousii*	36.30353	1.689E-09	8.84E-09	*
glut5	*D. rotundus*	26.48618	2.654E-07	1E-06	*
glut5	*A. jamaicensis*	18.16234	2.029E-05	5.31E-05	*
glut5	*M. harrisoni*	14.05766	0.0001773	0.00037	*
hexokinase1	*D. rotundus*	32.72959	1.059E-08	4.49E-08	*
hexokinase1	*A. jamaicensis*	22.41214	2.2E-06	6.53E-06	*
hexokinase	*M. waterhousii*	0.06581	0.7975	0.97229	
hexokinase	*L. nivalis*	8.506186	0.003539	0.00573	*
hexokinase	*L. yerbabuenae*	6.945342	0.008404	0.0129	*
insur2	*M. waterhousii*	34.83399	3.59E-09	1.72E-08	*
insur2	*D. rotundus*	40.82768	1.663E-10	1.14E-09	*
insur2	*A. jamaicensis*	2E-05	0.9964	0.9984	
insur2	*L. nivalis*	9.923646	0.001632	0.00296	*
insur2	*L. yerbabuenae*	0.00643	0.9361	0.9984	
lipali	*D. rotundus*	23.72939	1.109E-06	3.53E-06	*
lipali	*A. jamaicensis*	8.73168	0.003127	0.00525	*
lipali	*M. harrisoni*	17.10744	3.532E-05	8.5E-05	*
lipoli	*M. waterhousii*	2.2E-05	0.9963	0.9984	
lipoli	*L. yerbabuenae*	1.098838	0.2945	0.3912	
lipoli	*M. harrisoni*	14.5195	0.0001387	0.00029	*
lipoli	*L. yerbabuenae*	1.098838	0.2945	0.3912	
maltase	*D. rotundus*	25.3319	4.827E-07	1.79E-06	*
maltase	*M. harrisoni*	43.77396	3.686E-11	3E-10	*
maltase	*L. yerbabuenae*	5.956854	0.01466	0.02139	
pancreatic_am	*M. waterhousii*	24.61107	7.015E-07	2.34E-06	*
pancreatic_am	*D. rotundus*	13.74737	0.0002091	0.00042	*
pancreatic_am	*M. harrisoni*	16.57303	4.681E-05	0.0001	*
pancreatic_am	*L. nivalis*	20.93805	4.744E-06	1.28E-05	*
phosphofruck	*M. waterhousii*	0.098044	0.7542	0.94616	
phosphofruck	*M. harrisoni*	0.11646	0.7329	0.94533	
phosphofruck	*L. yerbabuenae*	65.69854	5.551E-16	2.47E-14	*
sglt1	*D. rotundus*	2.839626	0.09197	0.12593	

sglt1	*A. jamaicensis*	42.0472	8.91E-11	6.61E-10	*
tas1r2	*M. waterhousii*	49.68295	1.807E-12	2E-11	*
tas1r2	*A. jamaicensis*	57.08934	4.163E-14	7.41E-13	*
tas1r2	*M. harrisoni*	54.02113	1.984E-13	2.94E-12	*
tas1r2	*L. nivalis*	1.2E-05	0.9972	0.9984	

Table S7

Genes under positive selection involved in Carbohydrates and lipid metabolism

Gene	Name	Metabolic Pathway	Enzyme number	Species	*p-value*
CDIPT	CDP-diacylglycerol--inositol 3-phosphatidyltransferase	Glycerophospholipid / Inositol phosphate	2.7.8.11	*L. yerbabuenae*	0.001
BPGM	bisphosphoglycerate/ phosphoglycerate mutase	Glycolysis / Gluconeogenesis	5.4.2.4<br>5.4.2.11	*Mharrisoni*	0.002
NADHUFV2	NADH dehydrogenase (Ubiquinone)	Oxidative phosphorylation	7.1.1.2 1.6.99.3	*Lyerbabuenae*	0.0051
ALDO	fructose-bisphosphate aldolase, class I	Glycolysis / Gluconeogenesis	4.1.2.13	*A. jamaicensis*	0.002
PGLS	6-phosphogluconolactonase	Pentose phosphate	3.1.1.31	*L. nivalis*	0.002
FADH1	acylpyruvate hydrolase	TCA	3.7.1.5	*A.jamaicensis*	0.0004
MARS	methionyl-tRNA synthetase	Aminoacyl-tRNA biosynthesis	6.1.1.10	*D. rotundus*	3.6e-11
ETNK	ethanolamine kinase	Glycerophospholipid	2.7.1.82	*A. jamaicensis*	0.0004
NduFa12	NADH:ubiquinone oxidoreductase subunit A12	Glycolysis / Gluconeogenesis			
PFK	6-phosphofructokinase 1	Glycolysis / Gluconeogenesis / Fructose and Manose	2.7.1.11	*M. waterhousii*<br>*A. jamaicensis*<br>*L. yerbabuenae*	5.2e-08<br>0<br>4.4e-12
AMPK	5'-AMP-activated protein kinase	Insulin signaling		*A. jamaicensis*<br>*M.harrisoni*<br>*L. nivalis*<br>*L. yerbabuenae*	0.001<br>1.3e-07<br>7.9e-06<br>0.001
PYG	glycogen phosphorylase	Insulin signaling<br>Starch and sucrose<br>Glucagon signaling	2.4.1.1	*M. harrisoni*	0.002
CS	citrate synthase	Citrate cycle (TCA cycle)	2.3.3.1	*M. waterhousii*<br>*L. nivalis*	0<br>0.007
SLC2A1 / GLUT1	MFS transporter, SP family, solute carrier family 2 (facilitated glucose transporter), member 1	Insulin secretion<br>Glucagon		*A. jamaicensis*<br>*L. nivalis*	0<br>0
AMY	alpha-amylase	Carbohydrate digestion and	3.2.1.1	*D. rotundus*<br>*A. jamaicensis*	0<br>0

		absorption / Starch and Sucrose		*L. nivalis*	0
PNLIP	pancreatic triacylglycerol lipase	Pancreatic secretion<br><br>Fat digestion and absorption	3.1.1.3	*D. rotundus*	0.00001
FABP	fatty acid-binding protein	Fat digestion and absorption		*A. jamaicensis*<br>*M. harrisoni*<br>*L. nivalis*<br>*L. yerbabauenae*	5.5e-08<br>0.0002<br>0<br>0
FA2H	Fatty acid 2-hydroxylase	Fat digestion and absorption		*D. rotundus*<br>*L. nivalis*<br>*L. yerbabuenae*	1.3e-14<br>0.01<br>0.01
ACCS	acetoacetyl-CoA synthetase	Keto metabolism	6.2.1.16	*A. jamaicensis*<br>*M. harrisoni*<br>*L. nivalis*<br>*L. yerbabuenae*<br><br>*R. aegyptiacus*<br>*P. alecto*<br>*P. vampyrus*	0<br>0<br>0<br>0.0007<br><br>0<br>0<br>0
ALKBH7	Alpha-Ketoglutarate-Dependent Dioxygenase AlkB Homolog 7, Mitochondrial	Keto metabolism		*A. jamaicensis*<br>*M. harrisoni*<br>*L. nivalis*<br>*L. yerbabuenae*<br><br>*R. aegyptiacus*<br>*P. alecto*<br>*P. vampyrus*	0.0002<br>0<br>0<br>0<br><br>0.00001<br>0.005<br>0
BDH	3-hydroxybutyrate dehydrogenase	Synthesis of Ketone bodies	1.1.1.30	*D. rotundus*<br>*L. nivalis*	3e-12<br>2e-07

Table S8

GO enrichment for those positive selected genes for each Phyllostomid specie (dark gray- Biological Process; light gray- Molecular Function; and white – Celular Component)

Species	Function	GO	*p-value*
M. waterhousii (insect-feeder)	Negative regulation of Ras protein signal	GO:0046580	0.0035
	RNA processing	GO:0006396	0.0108
	DNA repair	GO:0006281	0.0205
	Cellular response to calcium ion	GO:0071277	0.0233
	snRNA processing	GO:0016180	0.0233
	tRNA splicing, via endonucleolytic	GO:0006388	0.0233
	Retinol metabolic process	GO:0042572	0.0233
	Immune response	GO:0006955	0.0276
	Endoplasmic reticulum unfolded protein	GO:0030968	0.0278
	Positive regulation of GTPase activity	GO:0043547	0.0278
	Chemotaxis	GO:0006935	0.0489
	ATP binding	GO:0005524	0.0014
	3',5'-cyclic-nucleotide phosphodiesteras	GO:0004114	0.0126
	Malate dehydrogenase (decarboxylating)	GO:0004471	0.0230
	Protein binding	GO:0005515	0.0248
	Quinone binding	GO:0048038	0.0275
	cAMP response element binding	GO:0035497	0.0275
	GTPase activator activity	GO:0005096	0.0338
	Cytokine receptor activity	GO:0004896	0.0484
	Rho guanyl-nucleotide exchange factor	GO:0005089	0.0486
	Metallopeptidase activity	GO:0008237	0.0487
	Proteasome core complex, alpha-subunit	GO:0019773	0.020
	Integrator complex	GO:0032039	0.020
D. rotundus (blood)	Mismatch repair	GO:0006298	0.00086
	RNA 3'-end processing	GO:0031123	0.00094
	rRNA processing	GO:0006364	0.00468
	Platelet activation	GO:0030168	0.00789
	Mitotic cell cycle	GO:0000278	0.00861
	Glutathione catabolic process	GO:0006751	0.01283
	Fatty acid beta-oxidation	GO:0006635	0.02564
	Gamma-aminobutyric acid catabolic process	GO:0009450	0.03726
	Lysosomal transport	GO:0007041	0.03726
	Fc receptor signaling pathway	GO:0038093	0.03726
	Cyclic nucleotide catabolic process	GO:0009214	0.03726
	Cytoplasmic microtubule organization	GO:0031122	0.03726
	Glucosylceramide catabolic process	GO:0006680	0.03726
	Mitotic spindle organization	GO:0007052	0.03726
	Ionotropic glutamate receptor activity	GO:0004970	5.8e-06
	Extracellularly glutamate-gated ion change	GO:0005234	5.8e-06
	[Heparan sulfate]-glucosamine	GO:0015016	5.9e-05

	Endopeptidase inhibitor activity<br>Protein-glutamine gamma-glutamyltransferase<br>Polynucleotide adenylyltransferase activity<br>Elongation factor-2 kinase activity<br>DNA-directed DNA polymerase activity<br>G protein-coupled receptor activity<br>Binding<br>Glycogen (starch) synthase activity<br>Tubulin N-acetyltransferase activity<br>ATP binding<br>Phospholipase A2 activity<br>acyl-CoA oxidase activity<br>Transporter activity<br>Motor activity<br>Heme binding<br>Platelet-derived growth factor beta-receptor<br>Succinate-semialdehyde dehydrogenase<br>Glucosylceramidase activity<br>Cytokine binding<br>SUMO binding<br>O-phospho-L-serine:2-oxoglutarate<br>Peptidoglycan binding<br>2',3'-cyclic-nucleotide 3'-phosphodiesterase<br>Leukotriene-A4 hydrolase activity<br>acetyl-CoA transmembrane transporter activity<br>Methylenetetrahydrofolate reductase<br>Thymidylate synthase activity<br>Interleukin-12 receptor binding<br>Acetyl-CoA:L-glutamate N-acetyltransferase<br>Nucleoside binding	GO:0004866<br>GO:0003810<br>GO:0004652<br>GO:0004686<br>GO:0003887<br>GO:0004930<br>GO:0005488<br>GO:0004373<br>GO:0019799<br>GO:0005524<br>GO:0004623<br>GO:0003997<br>GO:0005215<br>GO:0003774<br>GO:0020037<br>GO:0005019<br>GO:0009013<br>GO:0004348<br>GO:0019955<br>GO:0032183<br>GO:0004648<br>GO:0042834<br>GO:0004113<br>GO:0004463<br>GO:0008521<br>GO:0004489<br>GO:0004799<br>GO:0005143<br>GO:0004042<br>GO:0001882	6.7e-05<br>0.00011<br>0.00092<br>0.00136<br>0.00137<br>0.00230<br>0.00233<br>0.00398<br>0.00398<br>0.01146<br>0.01343<br>0.01849<br>0.02275<br>0.03121<br>0.03532<br>0.03693<br>0.03693<br>0.03693<br>0.03693<br>0.03693<br>0.03693<br>0.03693<br>0.03693<br>0.03693<br>0.03693<br>0.03693<br>0.03693<br>0.03693<br>0.03693<br>0.03696
	Nuclear pore<br>Microtubule<br>Fibrinogen complex<br>Peroxisome<br>Connexin complex<br>DNA replication factor C complex<br>Ciliary base<br>Nuclear condensin complex<br>Membrane	GO:0005643<br>GO:0005874<br>GO:0005577<br>GO:0005777<br>GO:0005922<br>GO:0005663<br>GO:0097546<br>GO:0000799<br>GO:0016020	1.2e-06<br>0.00076<br>0.00688<br>0.03040<br>0.03477<br>0.03477<br>0.03477<br>0.03477<br>0.04069
A. jamaicensis (frugivore)	rRNA processing<br>Mismatch repair<br>Vascular endothelial growth factor recep...<br>Flagellated sperm motility<br>Hippo signaling<br>tRNA thio-modification<br>Lipid storage<br>Phosphatidylinositol dephosphorylation	GO:0006364<br>GO:0006298<br>GO:0048010<br>GO:0030317<br>GO:0035329<br>GO:0034227<br>GO:0019915<br>GO:0046856	0.0065<br>0.0133<br>0.0243<br>0.0243<br>0.0243<br>0.0243<br>0.0243<br>0.0295

	Phospholipid catabolic process<br>Ribosome biogenesis<br>Fatty acid beta-oxidation	GO:0009395<br>GO:0042254<br>GO:0006635	0.0295<br>0.0408<br>0.0422
	Protein binding<br>ATP binding<br>Thiamine pyrophosphate binding<br>Phospholipase activity<br>Metal ion binding<br>Mismatched DNA binding<br>Serine-type exopeptidase activity<br>Arginyltransferase activity<br>snoRNA binding<br>Vascular endothelial growth factor-activ...<br>Oxoglutarate dehydrogenase (succinyl-tra...<br>ATP-dependent DNA helicase activity<br>tRNA binding<br>Calcium ion binding	GO:0005515<br>GO:0005524<br>GO:0030976<br>GO:0004620<br>GO:0046872<br>GO:0030983<br>GO:0070008<br>GO:0004057<br>GO:0030515<br>GO:0005021<br>GO:0004591<br>GO:0004003<br>GO:0000049<br>GO:0005509	0.00049<br>0.00252<br>0.00350<br>0.00608<br>0.00656<br>0.01240<br>0.02305<br>0.02307<br>0.02307<br>0.02307<br>0.02307<br>0.02751<br>0.04888<br>0.04953
	Plasma membrane<br>Centrosome<br>Condensin complex<br>Mismatch repair complex<br>Meiotic nuclear membrane microtubule	GO:0005886<br>GO:0005813<br>GO:0000796<br>GO:0032300<br>GO:0034993	0.018<br>0.018<br>0.021<br>0.021<br>0.024
M. harrisoni (nectar-pollen)	RNA processing<br>Metabolic process<br>Protein phosphorylation<br>Pseudouridine synthesis<br>Chromosome segregation<br>Protein deubiquitination<br>Protein transport<br>Phosphate ion transport<br>Platelet activation<br>Spindle assembly<br>Inflammatory response<br>Sulfate transport	GO:0006396<br>GO:0008152<br>GO:0006468<br>GO:0001522<br>GO:0007059<br>GO:0016579<br>GO:0015031<br>GO:0006817<br>GO:0030168<br>GO:0051225<br>GO:0006954<br>GO:0008272	0.00091<br>0.00109<br>0.00184<br>0.00312<br>0.00532<br>0.01661<br>0.02539<br>0.03051<br>0.03051<br>0.03051<br>0.04025<br>0.04342
	Protein binding<br>Binding<br>RNA binding<br>ATP binding<br>Protein kinase activity<br>Serine-type endopeptidase activity<br>3'-5' exonuclease activity<br>DNA-directed 5'-3' RNA polymerase activity<br>Growth factor activity<br>Translation release factor activity<br>NAD(P)+-protein-arginine ADP-ribosyltranferase<br>Oxidoreductase activity	GO:0005515<br>GO:0005488<br>GO:0003723<br>GO:0005524<br>GO:0004672<br>GO:0004252<br>GO:0008408<br>GO:0003899<br>GO:0008083<br>GO:0003747<br>GO:0003956<br>GO:0016491	0.00012<br>0.00036<br>0.00178<br>0.00271<br>0.00316<br>0.00400<br>0.00496<br>0.00711<br>0.01413<br>0.01732<br>0.01732<br>0.01867

	Nucleotide binding<br>Cytokine activity<br>Interleukin-1 receptor binding<br>Insulin receptor substrate binding<br>Phosphatidylinositol 3-kinase binding<br>Inorganic phosphate transmembrane transport<br>Peptide-methionine (S)-S-oxide reductase<br>Ubiquitin-protein transferase activity<br>Catalytic activity<br>Magnesium ion binding<br>Solute:proton antiporter activity<br>GTPase activator activity<br>Transmembrane receptor protein tyrosine	GO:0000166<br>GO:0005125<br>GO:0005149<br>GO:0043560<br>GO:0043548<br>GO:0005315<br>GO:0008113<br>GO:0004842<br>GO:0003824<br>GO:0000287<br>GO:0015299<br>GO:0005096<br>GO:0004714	0.01950<br>0.02319<br>0.02913<br>0.02913<br>0.02913<br>0.02913<br>0.02913<br>0.03145<br>0.03146<br>0.03365<br>0.03771<br>0.03997<br>0.04178
	Integral component of membrane<br>Extracellular space<br>Fibrinogen complex<br>RNA polymerase III complex	GO:0016021<br>GO:0005615<br>GO:0005577<br>GO:0005666	0.00073<br>0.00891<br>0.03139<br>0.03139
L. yerbabuenae (nectar-pollen)	Signal transduction<br>Anion transport<br>Mitotic sister chromatid cohesion<br>Phospholipid catabolic process<br>Tetrahydrofolate interconversion<br>Glycine biosynthetic process<br>Phosphatidylserine biosynthetic process<br>Regulation of endocytosis<br>Protein ADP-ribosylation<br>Response to oxidative stress<br>Cell redox homeostasis<br>Protein phosphorylation<br>Pseudouridine synthesis<br>RNA catabolic process<br>Nucleoside metabolic process<br>Inflammatory response<br>Methylation	GO:0007165<br>GO:0006820<br>GO:0007064<br>GO:0009395<br>GO:0035999<br>GO:0006545<br>GO:0006659<br>GO:0030100<br>GO:0006471<br>GO:0006979<br>GO:0045454<br>GO:0006468<br>GO:0001522<br>GO:0006401<br>GO:0009116<br>GO:0006954<br>GO:0032259	0.0005<br>0.0062<br>0.0065<br>0.0100<br>0.0147<br>0.0147<br>0.0147<br>0.0147<br>0.0147<br>0.0159<br>0.0160<br>0.0190<br>0.0351<br>0.0405<br>0.0424<br>0.0427<br>0.0429
	ATP binding<br>Binding<br>Protein binding<br>Heme binding<br>Palmitoyl hydrolase activity<br>Glycine hydroxymethyltransferase activity<br>Structural constituent of nuclear pore<br>Peroxidase activity<br>Protein kinase activity<br>Pseudouridine synthase activity<br>Nucleotide binding<br>Transferase activity	GO:0005524<br>GO:0005488<br>GO:0005515<br>GO:0020037<br>GO:0098599<br>GO:0004372<br>GO:0017056<br>GO:0004601<br>GO:0004672<br>GO:0009982<br>GO:0000166<br>GO:0016740	2.4e-05<br>4.7e-05<br>0.00014<br>0.00889<br>0.01442<br>0.01442<br>0.01442<br>0.02389<br>0.02460<br>0.03416<br>0.03423<br>0.03831

	Serine-type carboxypeptidase activity<br>3'-5' exonuclease activity<br>RNA helicase activity<br>NAD binding<br>tRNA binding<br>Steroid binding<br>DNA-directed DNA polymerase activity<br>Microtubule binding	GO:0004185<br>GO:0008408<br>GO:0003724<br>GO:0051287<br>GO:0000049<br>GO:0005496<br>GO:0003887<br>GO:0008017	0.03981<br>0.03981<br>0.03981<br>0.04068<br>0.04171<br>0.04171<br>0.04171<br>0.04199
	Membrane<br>Mitochondrial outer membrane translocase...<br>Extracellular space<br>Ctf18 RFC-like complex<br>MICOS complex<br>Microtubule cytoskeleton	GO:0016020<br>GO:0005742<br>GO:0005615<br>GO:0031390<br>GO:0061617<br>GO:0015630	0.0042<br>0.0101<br>0.0133<br>0.0314<br>0.0314<br>0.0314
L. nivalis (nectar-pollen)	Regulation of autophagy<br>Nuclear-transcribed mRNA catabolic proce...<br>Protein phosphorylation<br>DNA repair<br>Phospholipid catabolic process<br>Lipid catabolic process<br>ATP synthesis coupled proton transport<br>Cilium organization<br>Positive regulation of vasoconstriction<br>Leucyl-tRNA aminoacylation<br>Malate metabolic process<br>Plasma membrane tubulation<br>Positive regulation of cell cycle G2/M phase<br>Oxidation-reduction process<br>Regulation of JNK cascade<br>Double-strand break repair via homologous<br>DNA recombination	GO:0010506<br>GO:0000184<br>GO:0006468<br>GO:0006281<br>GO:0009395<br>GO:0016042<br>GO:0015986<br>GO:0044782<br>GO:0045907<br>GO:0006429<br>GO:0006108<br>GO:0097320<br>GO:1902751<br>GO:0055114<br>GO:0046328<br>GO:0000724<br>GO:0006310	0.0006<br>0.0006<br>0.0018<br>0.0045<br>0.0069<br>0.0186<br>0.0190<br>0.0244<br>0.0246<br>0.0246<br>0.0246<br>0.0246<br>0.0246<br>0.0289<br>0.0300<br>0.0300<br>0.0388

Protein binding	GO:0005515	1.3e-06
Protein kinase activity	GO:0004672	4.6e-05
ATP binding	GO:0005524	6.2e-05
Phosphatidylinositol binding	GO:0035091	0.00073
Nuclear receptor transcription coactivat...	GO:0030374	0.00248
tRNA binding	GO:0000049	0.00323
Fatty-acyl-CoA binding	GO:0000062	0.00367
Phospholipase activity	GO:0004620	0.00650
Oxidoreductase activity	GO:0016491	0.00703
Ubiquitin protein ligase activity	GO:0061630	0.00859
Protein tyrosine/serine/threonine phosph...	GO:0008138	0.01113
Aminoacyl-tRNA editing activity	GO:0002161	0.01299
Nuclear hormone receptor binding	GO:0035257	0.01299
Protein tyrosine kinase activity	GO:0004713	0.01899
Catalytic activity	GO:0003824	0.02091
Leucine-tRNA ligase activity	GO:0004823	0.02382
Histamine receptor activity	GO:0004969	0.02382
Phosphatidylinositol-3,4,5-trisphosphate	GO:0005547	0.02382
Ribonuclease activity	GO:0004540	0.02874
G protein-coupled GABA receptor activity	GO:0004965	0.02876
Acyl-CoA dehydrogenase activity	GO:0003995	0.02876
Signaling receptor activity	GO:0038023	0.04251
External side of plasma membrane	GO:0009897	0.0027
Ciliary basal body	GO:0036064	0.0027
Centrosome	GO:0005813	0.0050
Golgi apparatus	GO:0005794	0.0164
Cell surface	GO:0009986	0.0190
Membrane attack complex	GO:0005579	0.0193
MICOS complex	GO:0061617	0.0193
ELL-EAF complex	GO:0032783	0.0193
HAUS complex	GO:0070652	0.0193
Mitochondrial proton-transporting ATP	GO:0000276	0.0215
Cytoplasm	GO:0005737	0.0433

Probability at each node at specific position (ancestral reconstruction sequence)

	ACCS				
**Site**	**Node**	**AA Derived**	**Probability**	**AA Frequent**	**Probability**
20	72,73,75	R	0.991, 1, 0.967	G	0.009,0,0.028
492	72,73,75	T	0.937,0.952,0.889	A	0.051,0.008,0.104
529	75	S	0.954	G	0.04
530	72,73	P	0.996,1	A	0.004,0
721	73,75	S	0.998, 0.950	P	0.002,0.047
803	73	L	1	S	0

Nodes: 72 (*M. harrisoni, L. yerbabuenae, L.nivalis*)
73 (*L. yerbabuenae, L.nivalis*)
75 (*R. aegyptiacus, P. alecto*)

ALKBH7

**Site**	**Node**	**AA Derived**	**Probability**	**AA Frequent**	**Probability**
151	68	K	1	E	0
151	77	E	1	E	0
182	67	R	0.998	R	0.998
182	76,77	Q	0.997, 1	R	0.003,0

Nodes: 76 (*M. harrisoni, L. yerbabuenae, L.nivalis*)
77 (*L. yerbabuenae, L.nivalis*)
67 (*R. aegyptiacus, P. alecto, P. vampyrus*)
68 (*P. alecto, P. vampyrus*)

UNC-45 B

**Site**	**Node**	**AA Derived**	**Probability**	**AA Frequent**	**Probability**
114	72	R	1	L	0
114	73	R	1	L	0
114	74	R	1	L	0
114	75	R	1	L	0

Nodes: 76 (*M. harrisoni, L. yerbabuenae, L.nivalis*)
77 (*L. yerbabuenae, L.nivalis*)
67 (*R. aegyptiacus, P. alecto, P. vampyrus*)
68 (*P. alecto, P. vampyrus*)

Table S10

Protein 3D- structure alignments for the protein ACCS

Pymol align *L. yerbabuenae vs P. alecto*
Match: read scoring matrix.
Match: assigning 614 x 582 pairwise scores.
MatchAlign: aligning residues (614 vs 582)...
MatchAlign: score 2303.000
ExecutiveAlign: 4224 atoms aligned.
ExecutiveRMS: 266 atoms rejected during cycle 1 (RMSD=5.94).
ExecutiveRMS: 168 atoms rejected during cycle 2 (RMSD=1.55).
ExecutiveRMS: 216 atoms rejected during cycle 3 (RMSD=0.54).
ExecutiveRMS: 137 atoms rejected during cycle 4 (RMSD=0.12).
ExecutiveRMS: 82 atoms rejected during cycle 5 (RMSD=0.02).
Executive: RMSD = 0.003 (3355 to 3355 atoms)

Pymol align *L. yerbabuenae vs H. armiger*
Match: read scoring matrix.
Match: assigning 614 x 612 pairwise scores.
MatchAlign: aligning residues (614 vs 612)...
MatchAlign: score 2663.500
ExecutiveAlign: 4568 atoms aligned.
ExecutiveRMS: 183 atoms rejected during cycle 1 (RMSD=5.99).
ExecutiveRMS: 240 atoms rejected during cycle 2 (RMSD=1.78).
ExecutiveRMS: 186 atoms rejected during cycle 3 (RMSD=0.54).
ExecutiveRMS: 86 atoms rejected during cycle 4 (RMSD=0.08).
ExecutiveRMS: 93 atoms rejected during cycle 5 (RMSD=0.01).
Executive: RMSD = 0.002 (3780 to 3780 atoms)

Pymol align *P. alecto vs H. armiger*
Match: read scoring matrix.
Match: assigning 582 x 612 pairwise scores.
MatchAlign: aligning residues (582 vs 612)...
MatchAlign: score 2247.000
ExecutiveAlign: 4205 atoms aligned.
ExecutiveRMS: 337 atoms rejected during cycle 1 (RMSD=7.29).
ExecutiveRMS: 222 atoms rejected during cycle 2 (RMSD=2.65).
ExecutiveRMS: 265 atoms rejected during cycle 3 (RMSD=0.94).
ExecutiveRMS: 139 atoms rejected during cycle 4 (RMSD=0.22).
ExecutiveRMS: 95 atoms rejected during cycle 5 (RMSD=0.04).
Executive: RMSD = 0.006 (3147 to 3147 atoms)

Pymol align *Lyerbabuenae vs Mwaterhousii*
Match: read scoring matrix.
Match: assigning 614 x 585 pairwise scores.
MatchAlign: aligning residues (614 vs 585)...
MatchAlign: score 2561.000

ExecutiveAlign: 4099 atoms aligned.
ExecutiveRMS: 168 atoms rejected during cycle 1 (RMSD=3.86).
ExecutiveRMS: 221 atoms rejected during cycle 2 (RMSD=1.02).
ExecutiveRMS: 138 atoms rejected during cycle 3 (RMSD=0.26).
ExecutiveRMS: 61 atoms rejected during cycle 4 (RMSD=0.04).
ExecutiveRMS: 24 atoms rejected during cycle 5 (RMSD=0.00).
Executive: RMSD = 0.000 (3487 to 3487 atoms)

Pymol align *P. alecto vs Mwaterhousii*
Match: read scoring matrix.
Match: assigning 582 x 585 pairwise scores.
MatchAlign: aligning residues (582 vs 585)...
MatchAlign: score 2038.500
ExecutiveAlign: 3680 atoms aligned.
ExecutiveRMS: 267 atoms rejected during cycle 1 (RMSD=5.75).
ExecutiveRMS: 175 atoms rejected during cycle 2 (RMSD=1.97).
ExecutiveRMS: 181 atoms rejected during cycle 3 (RMSD=0.64).
ExecutiveRMS: 93 atoms rejected during cycle 4 (RMSD=0.15).
ExecutiveRMS: 64 atoms rejected during cycle 5 (RMSD=0.02).
Executive: RMSD = 0.004 (2900 to 2900 atoms)

PyMOL align *M. harrisoni vs P. alecto*
Match: read scoring matrix.
Match: assigning 614 x 582 pairwise scores.
MatchAlign: aligning residues (614 vs 582)...
MatchAlign: score 2334.000
ExecutiveAlign: 4251 atoms aligned.
ExecutiveRMS: 266 atoms rejected during cycle 1 (RMSD=5.66).
ExecutiveRMS: 197 atoms rejected during cycle 2 (RMSD=1.40).
ExecutiveRMS: 173 atoms rejected during cycle 3 (RMSD=0.47).
ExecutiveRMS: 100 atoms rejected during cycle 4 (RMSD=0.09).
ExecutiveRMS: 103 atoms rejected during cycle 5 (RMSD=0.02).
Executive: RMSD = 0.003 (3412 to 3412 atoms)

PyMOL align *M. harrisoni vs L. yerbabuenae*
Match: read scoring matrix.
Match: assigning 614 x 614 pairwise scores.
MatchAlign: aligning residues (614 vs 614)...
MatchAlign: score 3019.000
ExecutiveAlign: 4717 atoms aligned.
ExecutiveRMS: 43 atoms rejected during cycle 1 (RMSD=3.39).
ExecutiveRMS: 246 atoms rejected during cycle 2 (RMSD=0.86).
ExecutiveRMS: 144 atoms rejected during cycle 3 (RMSD=0.20).
ExecutiveRMS: 78 atoms rejected during cycle 4 (RMSD=0.03).
ExecutiveRMS: 70 atoms rejected during cycle 5 (RMSD=0.00).
Executive: RMSD = 0.000 (4136 to 4136 atoms)

PyMOL *M. harrisoni vs L. nivalis*
Match: read scoring matrix.

Match: assigning 614 x 599 pairwise scores.
MatchAlign: aligning residues (614 vs 599)...
MatchAlign: score 2858.500
ExecutiveAlign: 4571 atoms aligned.
ExecutiveRMS: 112 atoms rejected during cycle 1 (RMSD=4.18).
ExecutiveRMS: 178 atoms rejected during cycle 2 (RMSD=0.80).
ExecutiveRMS: 128 atoms rejected during cycle 3 (RMSD=0.11).
ExecutiveRMS: 101 atoms rejected during cycle 4 (RMSD=0.02).
ExecutiveRMS: 68 atoms rejected during cycle 5 (RMSD=0.00).
Executive: RMSD = 0.000 (3984 to 3984 atoms)

PyMOL *L. nivalis vs L. yerbabuenae*
Match: read scoring matrix.
Match: assigning 599 x 614 pairwise scores.
MatchAlign: aligning residues (599 vs 614)...
MatchAlign: score 2976.500
ExecutiveAlign: 4620 atoms aligned.
ExecutiveRMS: 118 atoms rejected during cycle 1 (RMSD=2.77).
ExecutiveRMS: 210 atoms rejected during cycle 2 (RMSD=0.74).
ExecutiveRMS: 99 atoms rejected during cycle 3 (RMSD=0.14).
ExecutiveRMS: 102 atoms rejected during cycle 4 (RMSD=0.03).
ExecutiveRMS: 32 atoms rejected during cycle 5 (RMSD=0.00).
Executive: RMSD = 0.000 (4059 to 4059 atoms)

3D-structure model generated by Phyre2

ACCS *M. waterhousii*
Confidence: 100.0% Coverage: 97%
577 residues (97% of your sequence) have been modelled with 100.0% confidence by the single highest scoring template.

ACCS *D. rotundus*
Confidence: 100.0% Coverage: 91%
612 residues (91% of your sequence) have been modelled with 100.0% confidence by the single highest scoring template.

ACCS *M. harrisoni*
Confidence: 100.0% Coverage: 91%
603 residues (91% of your sequence) have been modelled with 100.0% confidence by the single highest scoring template.
Additional confident templates have been detected (see Domain analysis) which cover other regions of your sequence.
655 residues (99%) could be modelled at >90% confidence using multiple-templates.

ACCS *L. nivalis*
Confidence: 100.0% Coverage: 91%
588 residues (91% of your sequence) have been modelled with 100.0% confidence by the single highest scoring template.

ACCS *L. yerbabuenae*
Confidence: 100.0% Coverage: 91%
603 residues (91% of your sequence) have been modelled with 100.0% confidence by the single highest scoring template.

ACCS *P. alecto*
Confidence: 100.0% Coverage: 91%
571 residues (91% of your sequence) have been modelled with 100.0% confidence by the single highest scoring template.
Additional confident templates have been detected (see Domain analysis) which cover other regions of your sequence.
627 residues (100%) could be modelled at >90% confidence using multiple-templates.

Phyre2

Email	yoce_pf@hotmail.com
Description	Model_ACCS
Date	Tue Mar 17 19:28:44 GMT 2020
Unique Job ID	fa9454616346ebf9

Secondary structure and disorder prediction

	1–60
Sequence	M S K K T R A R Q D E I L E G Q V M W E P D S K K I A L M D R F R A A V S A A C G L A L E N Y H D L Y Q W S V E S Y A D
Secondary structure	
SS confidence	
Disorder	? ? ? ? ? ? ? ? ? ? ? ? ? ? ? (1–15); ? (41); ? ? (43–44)
Disorder confidence	

	61–120
Sequence	F W A E F W K F S G I V S S R M Y D E A V D T S T G V A D V P E W F K G S R L N Y A E N L L G T K R E G K E D I G K V T
Secondary structure	
SS confidence	
Disorder	
Disorder confidence	

	121–180
Sequence	F D E L R Q K V A V F A A A M R K M G V K V G D R V T G Y L P N G E H A V E A M L A A V S I G A I W S S T S P D F G V N
Secondary structure	
SS confidence	
Disorder	
Disorder confidence	

	181–240
Sequence	G V L D R F S Q I Q P K L I F S V E A V G Y N G K R H A H L D K L Q Q V V R G L P D L E K V V V I P Y I A P K E E I D I
Secondary structure	
SS confidence	
Disorder	? ? ? ? ? ? (234–239)
Disorder confidence	

	241–300
Sequence	S K I P N S V F L D D F L A T G K G D Q A P Q L E F E Q L P F S H P L F I L F S S G R T G A P K C M V H S A G G T L I Q
Secondary structure	
SS confidence	
Disorder	? ? (258–259); ? (261)
Disorder confidence	

	301–360
Sequence	H L K E H L L H G D M T S S D T V F Y Y T T V G W I M W N W L V S T L A T G A A V V L Y D G S P L L P S P N V L W D L V
Secondary structure	
SS confidence	
Disorder	? (353)
Disorder confidence	

	361–420
Sequence	D R V G I T I F G T G P K W L S V L E E R K V R P A D T H S L Q T L H T I L S A G S P L K A Q S Y D Y V Y R C V K S S V
Secondary structure	
SS confidence	
Disorder	
Disorder confidence	

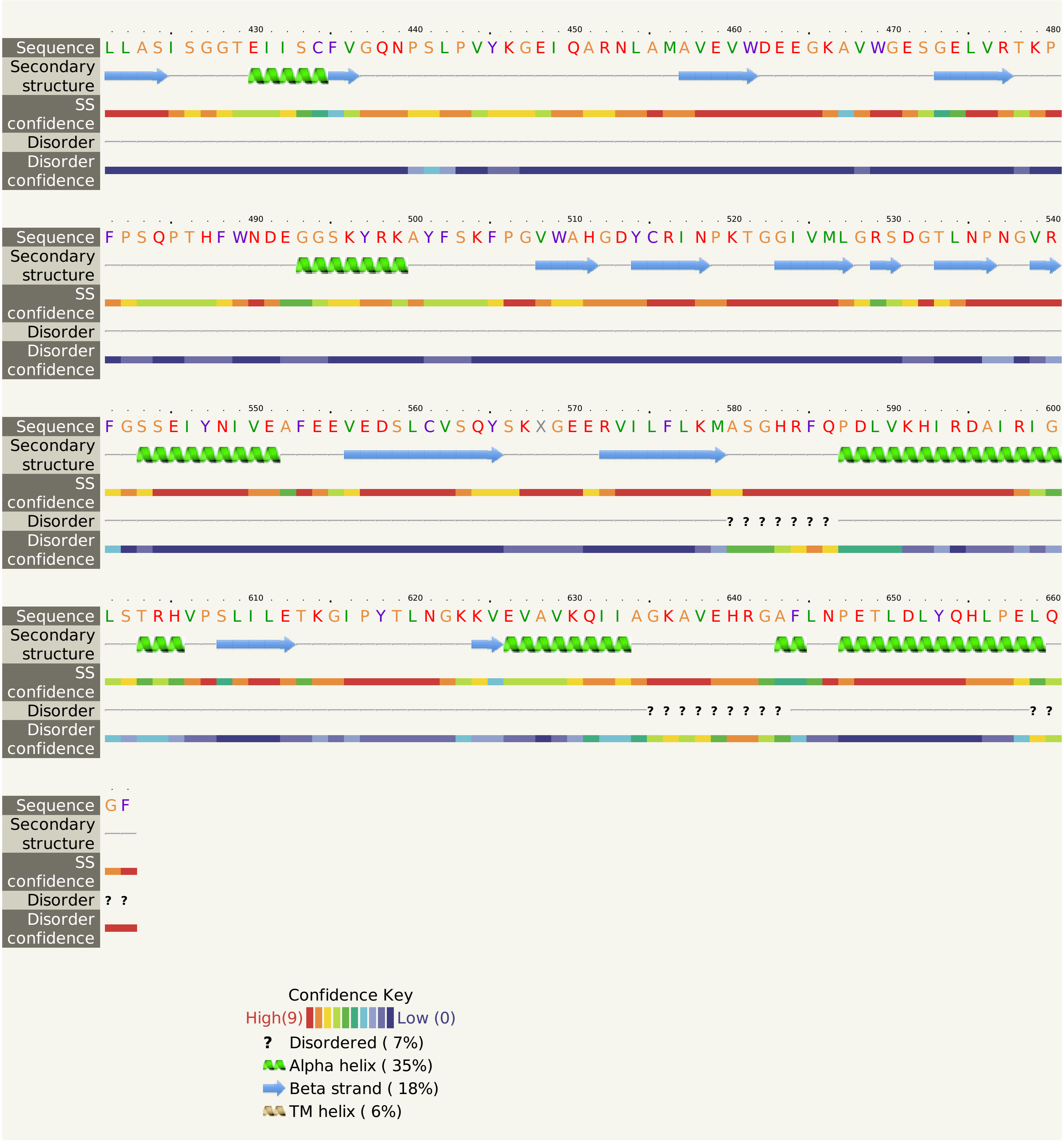
430 440 450 460 470 480
Sequence L L A S I S G G T E I I S C F V G Q N P S L P V Y K G E I Q A R N L A M A V E V W D E E G K A V W G E S G E L V R T K P
Secondary structure
SS confidence
Disorder
Disorder confidence
490 500 510 520 530 540
Sequence F P S Q P T H F W N D E G G S K Y R K A Y F S K F P G V W A H G D Y C R I N P K T G G I V M L G R S D G T L N P N G V R
Secondary structure
SS confidence
Disorder
Disorder confidence
550 560 570 580 590 600
Sequence F G S S E I Y N I V E A F E E V E D S L C V S Q Y S K X G E E R V I L F L K M A S G H R F Q P D L V K H I R D A I R I G
Secondary structure
SS confidence
Disorder ? ? ? ? ? ? ?
Disorder confidence
610 620 630 640 650 660
Sequence L S T R H V P S L I L E T K G I P Y T L N G K K V E V A V K Q I I A G K A V E H R G A F L N P E T L D L Y Q H L P E L Q
Secondary structure
SS confidence
Disorder ? ? ? ? ? ? ? ? ? ? ?
Disorder confidence
Sequence G F
Secondary structure
SS confidence
Disorder ? ?
Disorder confidence
Confidence Key
High(9) Low (0)
? Disordered (7%)
Alpha helix (35%)
Beta strand (18%)
TM helix (6%)

Phyre²

Email	yoss_279@comunidad.unam.mx
Description	ACCS_Lni_
Date	Mon Mar 23 07:11:38 GMT 2020
Unique Job ID	b32d4283961eeedf

Secondary structure and disorder prediction

	1–60
Sequence	S K K T R A R Q D E I L E G Q V M W E P D S K K I T L M D R F R A A V S A A Y G L A L E N Y H D L Y Q W S V E S Y A D F
Secondary structure	
SS confidence	
Disorder	? ? ? ? ? ? ? ? ? ? ? ? ? ? ? ? ?
Disorder confidence	

	61–120
Sequence	W A E F W K F S G I V S S R M Y D E V V D T S K G V A D V P E W F K G S R L N Y A E N L L G T K R E G K E D I G K V T F
Secondary structure	
SS confidence	
Disorder	
Disorder confidence	

	121–180
Sequence	D E L R Q X V A V F A A A M R K M G V K V G D R V T G Y L P N G E H A V E A M L A T A S I G A I W S S X S P D F G M N G
Secondary structure	
SS confidence	
Disorder	? ? ? ? ? ?
Disorder confidence	

	181–240
Sequence	V L D R F S Q I H P K L I L S V E A V V Y N G K R H A H L D K L Q Q V V R G L P D L E K V V V I P Y I A P X E E I D I S
Secondary structure	
SS confidence	
Disorder	? ? ? ? ? ? ? ? ? ? ? ? ? ? ? ? ?
Disorder confidence	

	241–300
Sequence	K I P N S V F L D D F L A T G K G D Q A P Q L E F E Q L P F S H P L F I L F S S G T T G A P K C M V H S A G G T L I Q H
Secondary structure	
SS confidence	
Disorder	? ? ? ? ? ? ? ? ? ? ? ? ?
Disorder confidence	

	301–360
Sequence	L K E H L L H G D M T R S D T V F C C T T V G W T M W N W M V S S L A T G A A V V L Y D G S P L L P S P N V L W D L V D
Secondary structure	
SS confidence	
Disorder	
Disorder confidence	

	361–420
Sequence	P K W L S R R E E A D T H S L Q T L H T I L S A G S P L K A Q S Y E Y V Y R C V K S S V L L A S I S G G T E I I S C F V
Secondary structure	
SS confidence	
Disorder	
Disorder confidence	

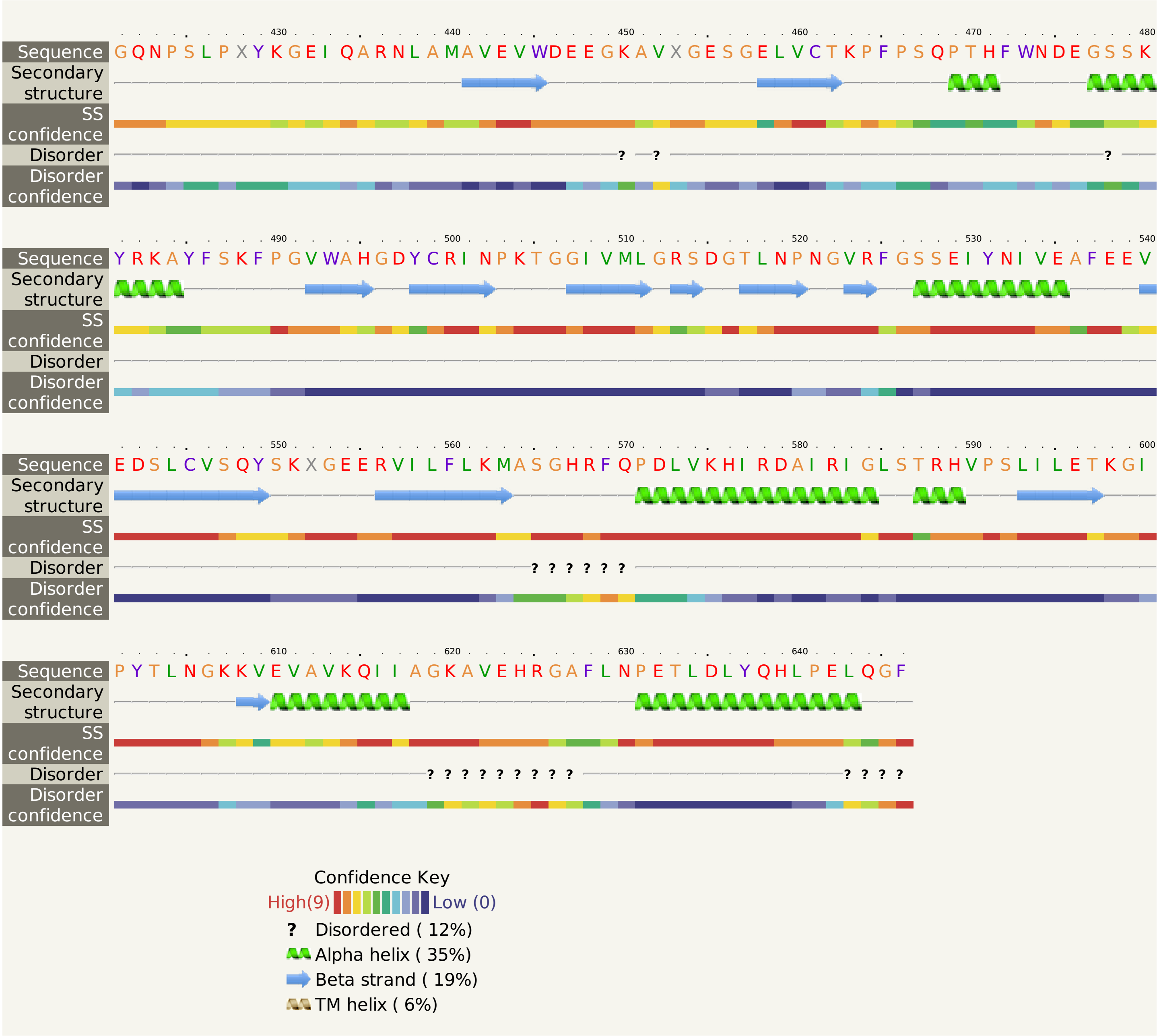

430 440 450 460 470 480
Sequence GQNPSLPXYKGEIQARNLAMAVEVWDEEGKAVXGESGELVCTKPFPSQPTHFWNDEGSSK
Secondary structure
SS confidence
Disorder ? ? ?
Disorder confidence
490 500 510 520 530 540
Sequence YRKAYFSKFPGVWAHGDYCRINPKTGGIVMLGRSDGTLNPNGVRFGSSEIYNIVEAFEEV
Secondary structure
SS confidence
Disorder
Disorder confidence
550 560 570 580 590 600
Sequence EDSLCVSQYSKXGEERVILFLKMASGHRFQPDLVKHIRDAIRIGLSTRHVPSLILETKGI
Secondary structure
SS confidence
Disorder ? ? ? ? ? ?
Disorder confidence
610 620 630 640
Sequence PYTLNGKKVEVAVKQIIAGKAVEHRGAFLNPETLDLYQHLPELQGF
Secondary structure
SS confidence
Disorder ? ? ? ? ? ? ? ? ? ? ? ? ?
Disorder confidence
Confidence Key
High(9) Low (0)
? Disordered (12%)
Alpha helix (35%)
Beta strand (19%)
TM helix (6%)

Phyre[2]

Email	yoce_pf@hotmail.com
Description	ACCS_Mha_
Date	Mon Mar 23 07:09:35 GMT 2020
Unique Job ID	cc7783c06d3d5166

Secondary structure and disorder prediction

Positions	Sequence
1–60	MSKEPWARQDEILEGQVMWEPDNKKITLMDRFRAAVSAACGLALENYHDLYQWSVESYSD
61–120	FWAEFWKFSGIISSHMYDKVVDTSKGVADVPEWFKGSRLNYAENVLGTKREGKEEIGKVT
121–180	FEELRQKVAVFAAAMRKMGVKVGDRVIGYLPNGEHAVEAMLAAVSIGAIWSSTSPDFGVN
181–240	GVLDRFSQIQPKLIFSVEAVVYNGKEHAHLNKLLQVVRGLPDLERVVVIPYITSREEIDI
241–300	SKIPNSVFLDDFLATGKGDQVPQLEFEQLPFNHPLFIMFSSGTTRAPKCMVHSAGGTLIQ
301–360	HLKEHLLHGDMTSSDTIFWYTTVGWMMWNWLVSTLATGAAVVLYDGSPLLPTPNVLWDLV
361–420	DRVGITVFGTGPKWLAVLEERKMRPADTHRLQTLHTIMSTGAPLKAQSYEYVYRCVKSDV

Row labels of each block: Sequence / Secondary structure / SS confidence / Disorder / Disorder confidence

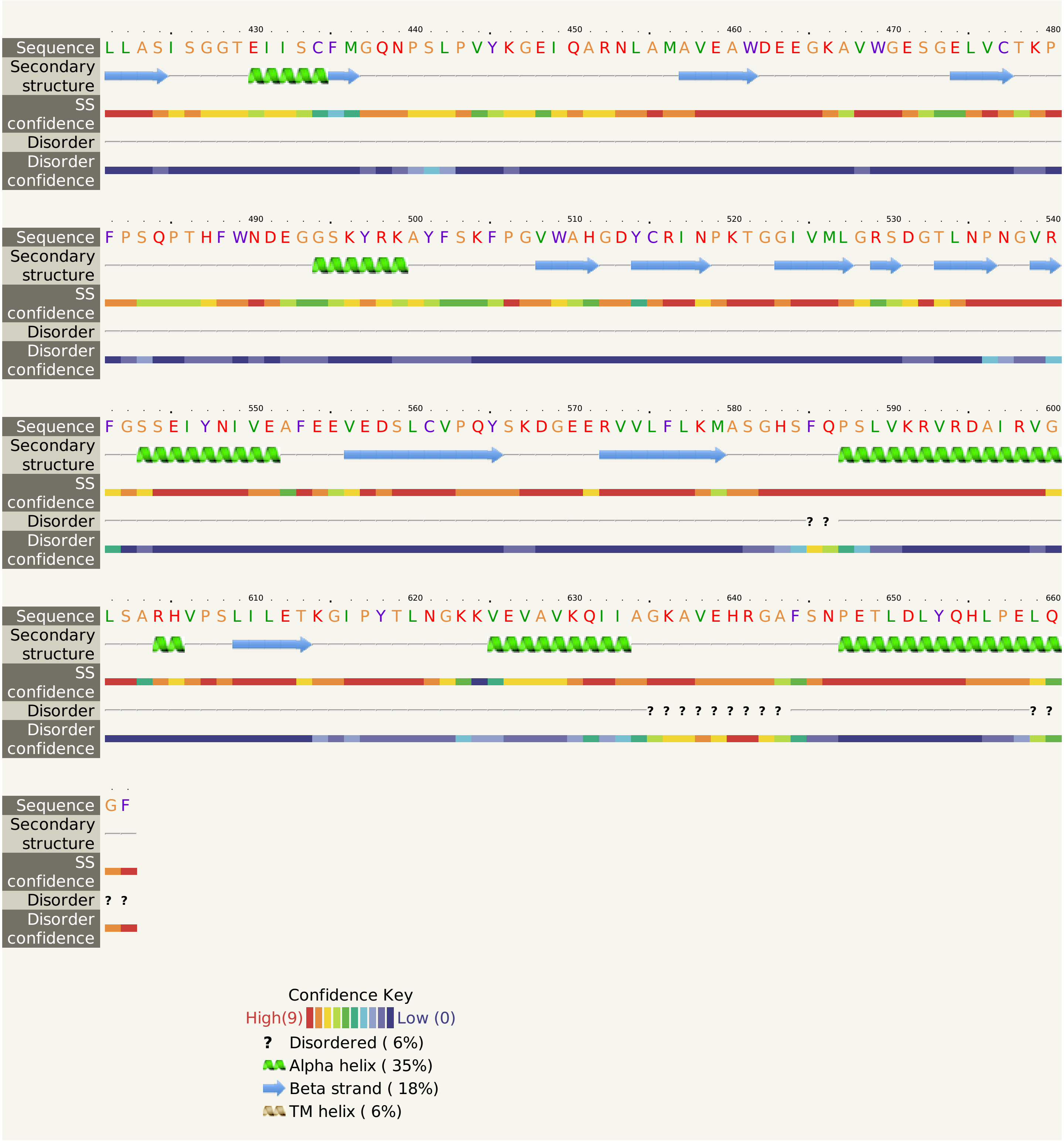
430 440 450 460 470 480
Sequence L L A S I S G G T E I I S C F M G Q N P S L P V Y K G E I Q A R N L A M A V E A W D E E G K A V W G E S G E L V C T K P
Secondary structure
SS confidence
Disorder
Disorder confidence
490 500 510 520 530 540
Sequence F P S Q P T H F W N D E G G S K Y R K A Y F S K F P G V W A H G D Y C R I N P K T G G I V M L G R S D G T L N P N G V R
Secondary structure
SS confidence
Disorder
Disorder confidence
550 560 570 580 590 600
Sequence F G S S E I Y N I V E A F E E V E D S L C V P Q Y S K D G E E R V V L F L K M A S G H S F Q P S L V K R V R D A I R V G
Secondary structure
SS confidence
Disorder ? ?
Disorder confidence
610 620 630 640 650 660
Sequence L S A R H V P S L I L E T K G I P Y T L N G K K V E V A V K Q I I A G K A V E H R G A F S N P E T L D L Y Q H L P E L Q
Secondary structure
SS confidence
Disorder ? ? ? ? ? ? ? ? ? ? ?
Disorder confidence
Sequence G F
Secondary structure
SS confidence
Disorder ? ?
Disorder confidence
Confidence Key
High(9) Low (0)
? Disordered (6%)
Alpha helix (35%)
Beta strand (18%)
TM helix (6%)

Phyre²

Email	yoce_pf@hotmail.com
Description	Accs_pteropodid
Date	Wed Mar 18 17:10:00 GMT 2020
Unique Job ID	8c6ebbd2b0f4998e

Secondary structure and disorder prediction

1 . . . . 10 . . . . 20 . . . . 30 . . . . 40 . . . . 50 60

Sequence: M S K E L Q A R Q D E I L E C R V M W E P D S K R N T E M D R F R A A V S A A R G L S L Q N Y N D L Y R W S I E S Y S D

Secondary structure

SS confidence

Disorder: ? ? ? ? ? ? ? ? ? ? ? ? ? ? ? ? ? ?

Disorder confidence

. . . . 70 . . . . 80 . . . . 90 . . . . 100 . . . . 110 120

Sequence: F W A E F W K F S R I V Y S R M Y D E V V D P S K G I A D V P E W F R G S R L N Y A E N L L R H G E D G R V A L Y V V R

Secondary structure

SS confidence

Disorder

Disorder confidence

. . . . 130 . . . . 140 . . . . 150 . . . . 160 . . . . 170 180

Sequence: E D K E E I V K V T F E E L R Q Q V A L F A A A M R K M G V E K G D R V V G Y L P N G E H A V E A M L A A A S I G A I W

Secondary structure

SS confidence

Disorder

Disorder confidence

. . . . 190 . . . . 200 . . . . 210 . . . . 220 . . . . 230 240

Sequence: S S M S P D F G V T G V L D R C S Q I R P K L I F S V E A V V F N G K E H S H L D K V Q Q V T K G L S D L K K V V V I P

Secondary structure

SS confidence

Disorder

Disorder confidence

. . . . 250 . . . . 260 . . . . 270 . . . . 280 . . . . 290 300

Sequence: Y V T S R D K I D I S K I P N S V F L D D F L A A G R G D R A P Q L E F T Q L P F N H P L F I L F S S G T T G V P K C L

Secondary structure

SS confidence

Disorder: ? ? ? ? ? ? ?

Disorder confidence

. . . . 310 . . . . 320 . . . . 330 . . . . 340 . . . . 350 360

Sequence: V H S A G G T L I Q H L K E H S L H G N M T S S D I I L F Y T T V G W M V W N W I L S T L A M G M A V V L Y D G S V L K

Secondary structure

SS confidence

Disorder

Disorder confidence

. . . . 370 . . . . 380 . . . . 390 . . . . 400 . . . . 410 420

Sequence: P T L N V L W D L V D R I G V T I L G T S P T W L S M L E R K N L K P V E T H D L R S L R M V M S T G S P L L A Q G Y E

Secondary structure

SS confidence

Disorder

Disorder confidence

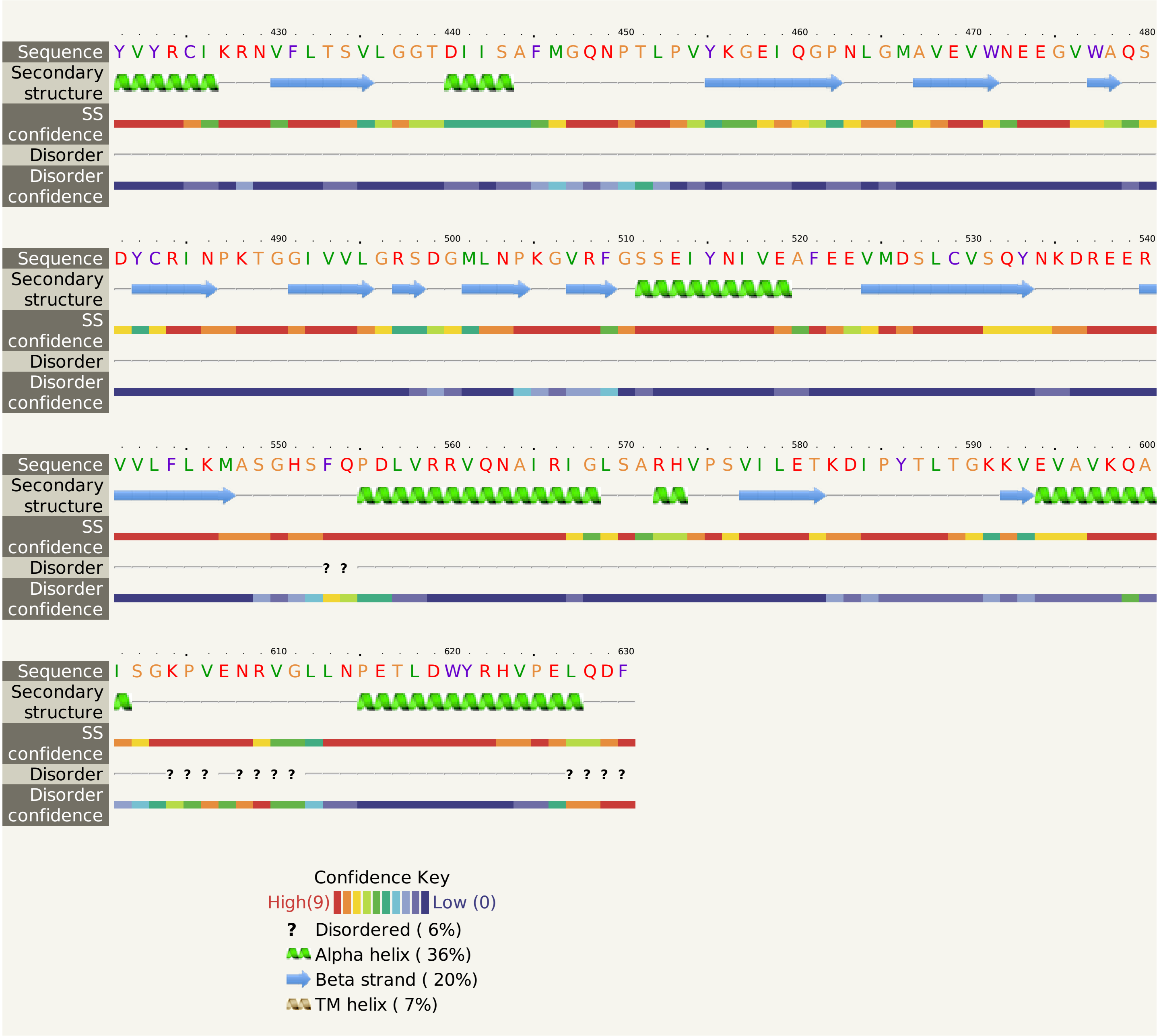

430 440 450 460 470 480
Sequence YVYRCIKRNVFLTSVLGGTDIISAFMGQNPTLPVYKGEIQGPNLGMAVEVWNEEGVWAQS
Secondary structure
SS confidence
Disorder
Disorder confidence
490 500 510 520 530 540
Sequence DYCRINPKTGGIVVLGRSDGMLNPKGVRFGSSEIYNIVEAFEEVMDSLCVSQYNKDREER
Secondary structure
SS confidence
Disorder
Disorder confidence
550 560 570 580 590 600
Sequence VVLFLKMASGHSFQPDLVRRVQNAIRIGLSARHVPSVILETKDIPYTLTGKKVEVAVKQA
Secondary structure
SS confidence
Disorder
Disorder confidence
610 620 630
Sequence ISGKPVENRVGLLNPETLDWYRHVPELQDF
Secondary structure
SS confidence
Disorder
Disorder confidence
Confidence Key
High(9) Low (0)
? Disordered (6%)
Alpha helix (36%)
Beta strand (20%)
TM helix (7%)

Phyre²

Email	yossgtzgro26@gmail.com
Description	ACCS_Dro_
Date	Mon Mar 23 07:13:30 GMT 2020
Unique Job ID	a644207efe576da7

Secondary structure and disorder prediction

	1–60
Sequence	MSKEPRAGREEILEGQVMWEPDSKKITLMDRFRAAVGAACGLALENYHDLYQWSVESYSD
Secondary structure	
SS confidence	
Disorder	? ? ? ? ? ? ? ? ? ? ? ? ? ? — ? — ? ? —
Disorder confidence	

	61–120
Sequence	FWAEFWKFSGIVSSRMYDQVVDTSKGVADIPEWFKGSRLNYAENLLRHKENDKVALYVAR
Secondary structure	
SS confidence	
Disorder	
Disorder confidence	

	121–180
Sequence	EDKEEIGKVTFEELRQKVALFAAAMRKMGVKVGDRVVGYLPNGEHAVEAMLATASIGAIW
Secondary structure	
SS confidence	
Disorder	
Disorder confidence	

	181–240
Sequence	SSTSPDFGVNGVLDRFSQIQPKLIFSVEAVVYNGKEHAHLDKLLQVVRGLSDLKKVVVIP
Secondary structure	
SS confidence	
Disorder	
Disorder confidence	

	241–300
Sequence	YITSREKIDISKIPNSVFLDDFLATGKGDQVPQLEFEQLPFSHPLFIVFSSGTTGAPKCM
Secondary structure	
SS confidence	
Disorder	— ? ? ? ? ? ? ? — ? —
Disorder confidence	

	301–360
Sequence	VHSAGGTLIQHLKEHLLHGDMTSSDIILYYTTVGWMMWNWLVSALATGAAVVLYDGSPLL
Secondary structure	
SS confidence	
Disorder	
Disorder confidence	

	361–420
Sequence	PTPNVLWDLVDRVGITILGTGAKWLAVLEERNLRPVDTHSLQTLHTILSTGSPLKAQSYE
Secondary structure	
SS confidence	
Disorder	
Disorder confidence	

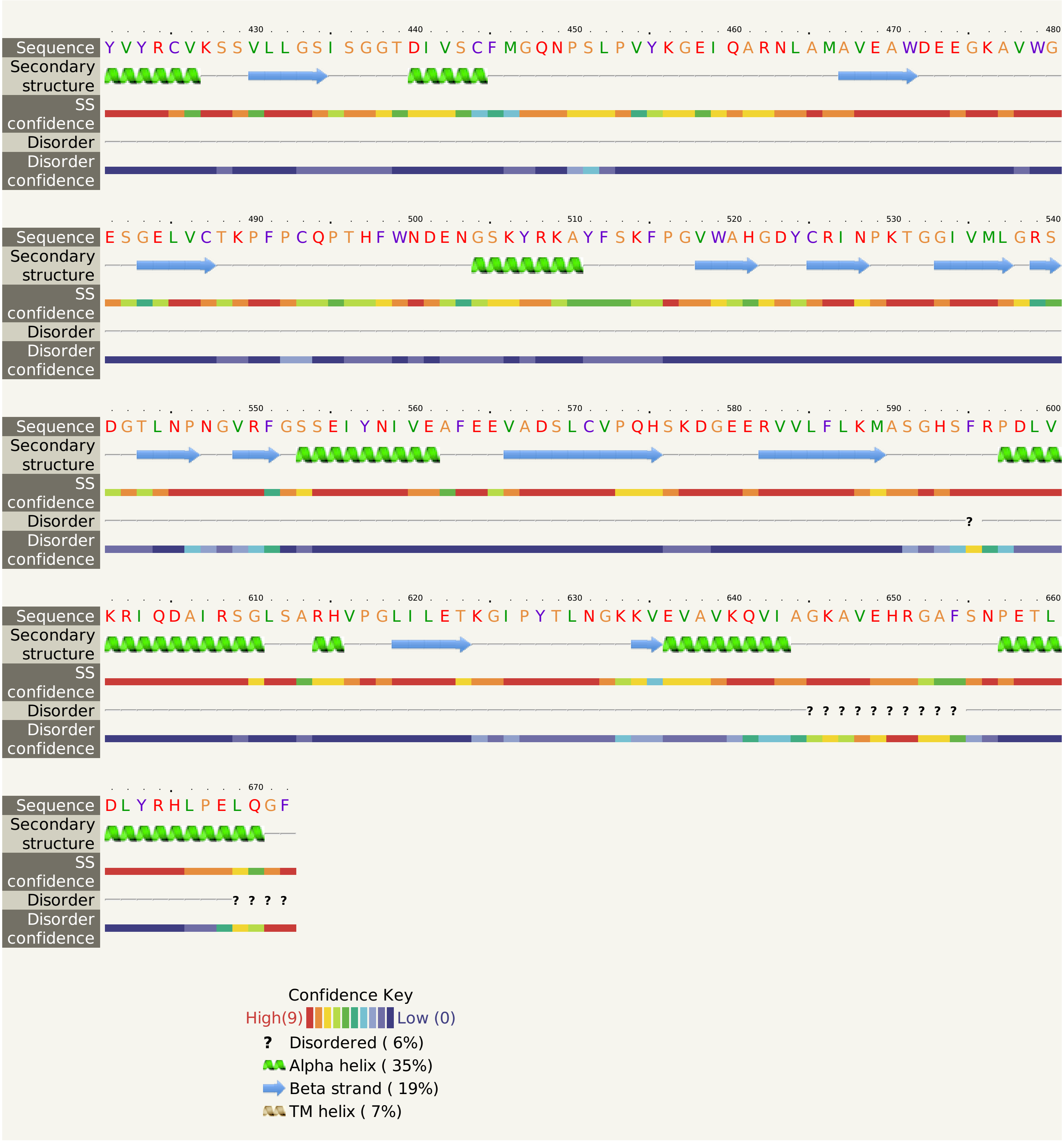

430 440 450 460 470 480
Sequence YVYRCVKSSVLLGSISGGTDIVSCFMGQNPSLPVYKGEIQARNLAMAVEAWDEEGKAVWG
Secondary structure
SS confidence
Disorder
Disorder confidence
490 500 510 520 530 540
Sequence ESGELVCTKPFPCQPTHFWNDENGSKYRKAYFSKFPGVWAHGDYCRINPKTGGIVMLGRS
Secondary structure
SS confidence
Disorder
Disorder confidence
550 560 570 580 590 600
Sequence DGTLNPNGVRFGSSEIYNIVEAFEEVADSLCVPQHSKDGEERVVLFLKMASGHSFRPDLV
Secondary structure
SS confidence
Disorder ?
Disorder confidence
610 620 630 640 650 660
Sequence KRIQDAIRSGLSARHVPGLILETKGIPYTLNGKKVEVAVKQVIAGKAVEHRGAFSNPETL
Secondary structure
SS confidence
Disorder ? ? ? ? ? ? ? ? ? ?
Disorder confidence
670
Sequence DLYRHLPELQGF
Secondary structure
SS confidence
Disorder ? ? ? ?
Disorder confidence
Confidence Key
High(9) Low (0)
? Disordered (6%)
Alpha helix (35%)
Beta strand (19%)
TM helix (7%)

Phyre2

Email	yossgtzgro26@gmail.com
Description	Accs_Mwa_
Date	Wed Mar 18 20:07:32 GMT 2020
Unique Job ID	18ac7aa06c62858c

Secondary structure and disorder prediction

1 . . . . 10 . . . . 20 . . . . 30 . . . . 40 . . . . 50 60

Sequence: MSKEPRAGRDEILEGQVMWEPDSKKITQMDRFRAAVGAACGLALGEGKEEIGKVTFEELR

Secondary structure | SS confidence | Disorder | Disorder confidence

. . . . 70 . . . . 80 . . . . 90 . . . . 100 . . . . 110 120

Sequence: QKVALFAAAMRKMGVKVGDRVTGYLPNGEHAVEAMLAVSSIGAIWSSTSPDFGVNGVLDR

Secondary structure | SS confidence | Disorder | Disorder confidence

. . . . 130 . . . . 140 . . . . 150 . . . . 160 . . . . 170 180

Sequence: FSQIQPKLIFSVEAVVYNGKEHTHLDKLHQVVRDLKKVVVIPYITSREKIDISKIPNSMF

Secondary structure | SS confidence | Disorder | Disorder confidence

. . . . 190 . . . . 200 . . . . 210 . . . . 220 . . . . 230 240

Sequence: LDDFLATGKGDQVPQLEFEQLPFSHPLFIMFSSGTTGAPKCMVHSAGGTLIQHLKEHLLH

Secondary structure | SS confidence | Disorder | Disorder confidence

. . . . 250 . . . . 260 . . . . 270 . . . . 280 . . . . 290 300

Sequence: GNMTSSDIIFCYTTVGWMMWNWLVSALATGAAVVLYDGSPLVPTPTVLWDLVDRIGITIF

Secondary structure | SS confidence | Disorder | Disorder confidence

. . . . 310 . . . . 320 . . . . 330 . . . . 340 . . . . 350 360

Sequence: GTGAKWLSVLEEKNMRPMETHSLQTLHTILSTGSPLKAQSYEYVYRCVKSSVLLGSISGG

Secondary structure | SS confidence | Disorder | Disorder confidence

. . . . 370 . . . . 380 . . . . 390 . . . . 400 . . . . 410 420

Sequence: TDIVSCFMGQNPSLPVHKGEIQARNLAMAVEAWDEEGKAVWGESGELVCTKPFPCQPTHF

Secondary structure | SS confidence | Disorder | Disorder confidence

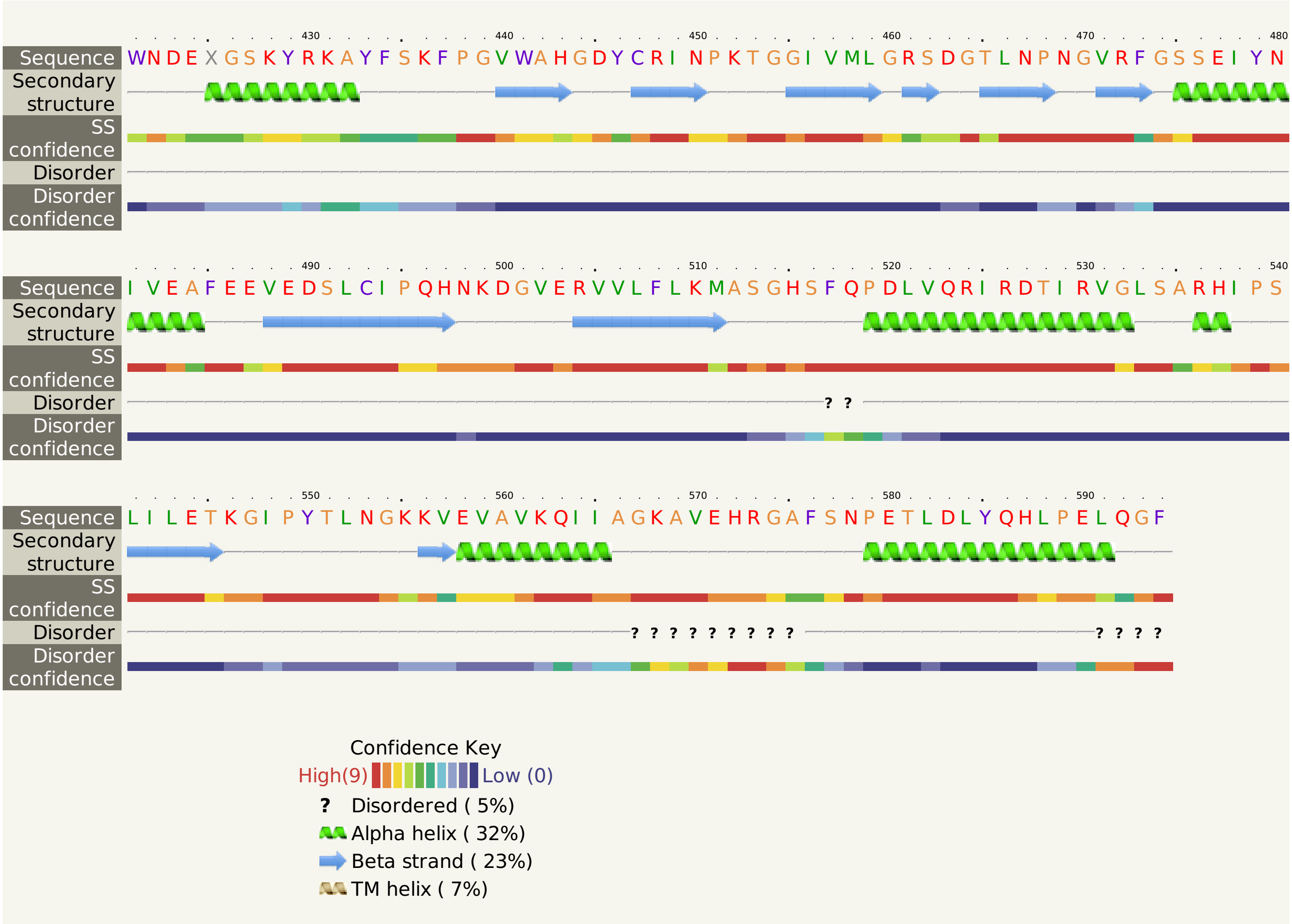

Sequence
Secondary structure
SS confidence
Disorder
Disorder confidence
430 440 450 460 470 480
WNDEXGSKYRKAYFSKFPGVWAHGDYCRINPKTGGIVMLGRSDGTLNPNGVRFGSSEIYN
490 500 510 520 530 540
IVEAFEEVEDSLCIPQHNKDGVERVVLFLKMASGHSFQPDLVQRIRDTIRVGLSARHIPS
? ?
550 560 570 580 590
LILETKGIPYTLNGKKVEVAVKQIIAGKAVEHRGAFSNPETLDLYQHLPELQGF
? ? ? ? ? ? ? ? ?
? ? ? ?
Confidence Key
High(9)
Low (0)
? Disordered (5%)
Alpha helix (32%)
Beta strand (23%)
TM helix (7%)

Phyre2

Email	yoss_279@comunidad.unam.mx
Description	Accs_Harmi
Date	Wed Mar 18 17:31:33 GMT 2020
Unique Job ID	e404d88c85dc7673

Secondary structure and disorder prediction

	1–60
Sequence	MSKEPRSGRGEILECQVMWEPDSKKNTLMDRFRAAVSAACGLALENYGDLYHWSVESYSD
Secondary structure	
SS confidence	
Disorder	? ? ? ? ? ? ? ? ? ? ? ? ? ? ? ? ? ?
Disorder confidence	

	61–120
Sequence	FWAEFWKFSGIVCSRTYDEVVDTSKGVADVPEWFKGSRLNYAENLLLHKENDRVALYVAR
Secondary structure	
SS confidence	
Disorder	
Disorder confidence	

	121–180
Sequence	DPQHRAGLTTVNFRTFSPPQKEPHTRYLPNGVHAVEAMLATASIGAIWSATSPDFGMNGV
Secondary structure	
SS confidence	
Disorder	
Disorder confidence	

	181–240
Sequence	LDRFSQIQPKLMFSVEAVVYNGKEHGHMHKLQQVVKGLPDLKRVVVIPYIASRENIDISK
Secondary structure	
SS confidence	
Disorder	
Disorder confidence	

	241–300
Sequence	IPNSVFLDDFLATGRGEQVPQLEFEQLPFSHPLFIMFSSGTTGAPKCMVHSAGGTLIQHL
Secondary structure	
SS confidence	
Disorder	
Disorder confidence	

	301–360
Sequence	KEHVLHGNMTSSDIILYYSTVGWMMWNWMVSALATGAAVVLYDGSPLVPTPNVLWDLVDR
Secondary structure	
SS confidence	
Disorder	
Disorder confidence	

	361–420
Sequence	IGITILGTGAKWLSVLEERNMKPVETHSLQTLHTILSTGSPLKAQSYEYVYRCIKSSVLL
Secondary structure	
SS confidence	
Disorder	
Disorder confidence	

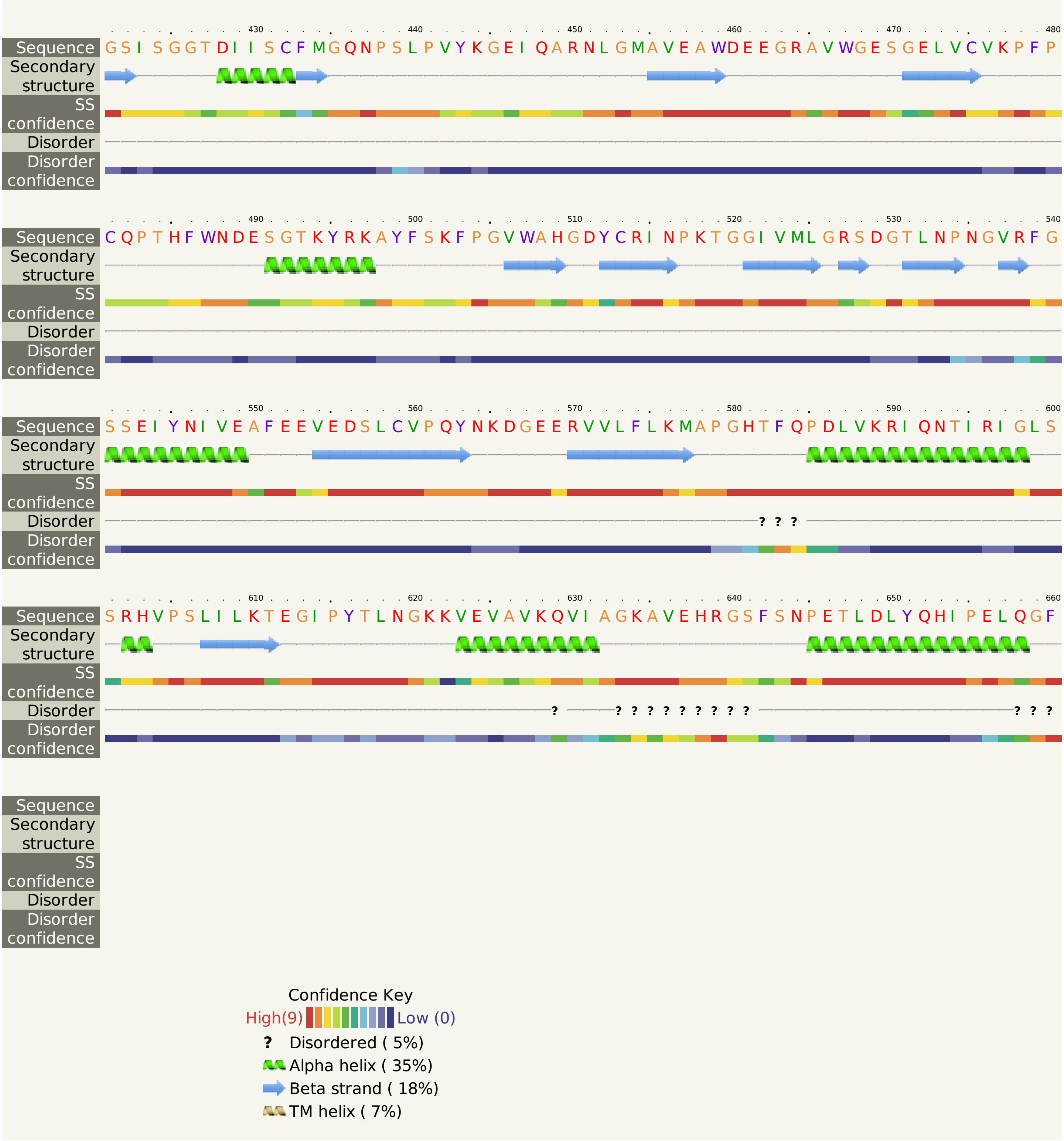
430 440 450 460 470 480
Sequence GSISGGTDIISCFMGQNPSLPVYKGEIQARNLGMAVEAWDEEGRAVWGESGELVCVKPFP
Secondary structure
SS confidence
Disorder
Disorder confidence
490 500 510 520 530 540
Sequence CQPTHFWNDESGTKYRKAYFSKFPGVWAHGDYCRINPKTGGIVMLGRSDGTLNPNGVRFG
Secondary structure
SS confidence
Disorder
Disorder confidence
550 560 570 580 590 600
Sequence SSEIYNIVEAFEEVEDSLCVPQYNKDGEERVVLFLKMAPGHTFQPDLVKRIQNTIRIGLS
Secondary structure
SS confidence
Disorder ? ? ?
Disorder confidence
610 620 630 640 650 660
Sequence SRHVPSLILKTEGIPYTLNGKKVEVAVKQVIAGKAVEHRGSFSNPETLDLYQHIPELQGF
Secondary structure
SS confidence
Disorder ? ? ? ? ? ? ? ? ? ? ? ? ?
Disorder confidence
Sequence
Secondary structure
SS confidence
Disorder
Disorder confidence
Confidence Key
High(9) Low (0)
? Disordered (5%)
Alpha helix (35%)
Beta strand (18%)
TM helix (7%)

Table S12

Database: Laurasiatheria and other mammals

Homo sapiens	ftp://ftp.ensembl.org/pub/release-99/fasta/homo_sapiens/pep/*all.fa.gz
Mus musculus	ftp://ftp.ensembl.org/pub/release-99/fasta/mus_musculus/pep/*all.fa.gz
Erinaceus europaeus	ftp://ftp.ensembl.org/pub/release-99/fasta/erinaceus_europaeus/pep/*all.fa.gz
Sorex araneus	ftp://ftp.ensembl.org/pub/release-99/fasta/sorex_araneus/pep/*all.fa.gz
Myotis lucifugus	ftp://ftp.ensembl.org/pub/release-99/fasta/myotis_lucifugus/pep/*all.fa.gz
Rhinolophus ferrumequinum	ftp://ftp.ensembl.org/pub/release-99/fasta/rhinolophus_ferrumequinum/pep/*all.fa.gz
Pteropus vampyrus	ftp://ftp.ensembl.org/pub/release-99/fasta/pteropus_vampyrus/pep/*all.fa.gz
Equus caballus	ftp://ftp.ensembl.org/pub/release-99/fasta/equus_caballus/pep/*all.fa.gz
Equus asinus asinus	ftp://ftp.ensembl.org/pub/release-99/fasta/equus_asinus_asinus/pep/*all.fa.gz
Panthera pardus	ftp://ftp.ensembl.org/pub/release-99/fasta/panthera_pardus/pep/*all.fa.gz
Panthera tigris altaica	ftp://ftp.ensembl.org/pub/release-99/fasta/panthera_tigris_altaica/pep/*all.fa.gz
Felis catus	ftp://ftp.ensembl.org/pub/release-99/fasta/felis_catus/pep/*all.fa.gz
Lynx canadensis	ftp://ftp.ensembl.org/pub/release-99/fasta/lynx_canadensis/pep/*all.fa.gz
Suricata suricatta	ftp://ftp.ensembl.org/pub/release-99/fasta/suricata_suricatta/pep/*all.fa.gz
Canis familiaris	ftp://ftp.ensembl.org/pub/release-99/fasta/canis_familiaris/pep/*all.fa.gz
Canis lupus dingo	ftp://ftp.ensembl.org/pub/release-99/fasta/canis_lupus_dingo/pep/*all.fa.gz
Vulpes vulpes	ftp://ftp.ensembl.org/pub/release-99/fasta/vulpes_vulpes/pep/*all.fa.gz
Ursus americanus	ftp://ftp.ensembl.org/pub/release-99/fasta/ursus_americanus/pep/*all.fa.gz
Ursus maritimus	ftp://ftp.ensembl.org/pub/release-99/fasta/ursus_maritimus/pep/*all.fa.gz
Ailuropoda melanoleuca	ftp://ftp.ensembl.org/pub/release-99/fasta/ailuropoda_melanoleuca/pep/*all.fa.gz
Mustela putorius furo	ftp://ftp.ensembl.org/pub/release-99/fasta/mustela_putorius_furo/pep/*all.fa.gz
Neovison vison	ftp://ftp.ensembl.org/pub/release-99/fasta/neovison_vison/pep/*all.fa.gz
Tursiops truncatus	ftp://ftp.ensembl.org/pub/release-99/fasta/tursiops_truncatus/pep/*all.fa.gz
Physeter catodon	ftp://ftp.ensembl.org/pub/release-99/fasta/physeter_catodon/pep/*all.fa.gz
Camelus dromedarius	ftp://ftp.ensembl.org/pub/release-99/fasta/camelus_dromedarius/pep/*all.fa.gz
Vicugna pacos	ftp://ftp.ensembl.org/pub/release-99/fasta/vicugna_pacos/pep/*all.fa.gz
Sus scrofa	ftp://ftp.ensembl.org/pub/release-99/fasta/sus_scrofa/pep/*all.fa.gz
Catagonus wagneri	ftp://ftp.ensembl.org/pub/release-99/fasta/catagonus_wagneri/pep/*all.fa.gz
Capra hircus	ftp://ftp.ensembl.org/pub/release-99/fasta/capra_hircus/pep/*all.fa.gz
Ovis aries	ftp://ftp.ensembl.org/pub/release-99/fasta/ovis_aries/pep/*all.fa.gz
Bos taurus	ftp://ftp.ensembl.org/pub/release-99/fasta/bos_taurus/pep/*all.fa.gz
Bos grunniens	ftp://ftp.ensembl.org/pub/release-99/fasta/bos_grunniens/pep/*all.fa.gz
Bos mutus	ftp://ftp.ensembl.org/pub/release-99/fasta/bos_mutus/pep/*all.fa.gz
Bison bison bison	ftp://ftp.ensembl.org/pub/release-99/fasta/bison_bison_bison/pep/*all.fa.gz
Moschus moschiferus	ftp://ftp.ensembl.org/pub/release-99/fasta/moschus_moschiferus/pep/*all.fa.gz
Ornithorhynchus anatinus	https://ftp.ncbi.nlm.nih.gov/genomes/all/GCF/004/115/215/GCF_004115215.1_mOrnAna
Rousettus aegyptiacus	https://ftp.ncbi.nlm.nih.gov/genomes/all/GCF/001/466/805/GCF_001466805.2_Raegyp2
Pteropus vampyrus	https://ftp.ncbi.nlm.nih.gov/genomes/all/GCF/000/151/845/GCF_000151845.1_Pvam_2.
Miniopterus natalensis	ftp://ftp.ncbi.nlm.nih.gov/genomes/all/GCF/001/595/765/GCF_001595765.1_Mnat.v1/*_
Eptesicus fuscus	https://ftp.ncbi.nlm.nih.gov/genomes/all/GCF/000/308/155/GCF_000308155.1_EptFus1.
Myotis davidii	https://ftp.ncbi.nlm.nih.gov/genomes/all/GCF/000/327/345/GCF_000327345.1_ASM327
Myotis brandtii	https://ftp.ncbi.nlm.nih.gov/genomes/all/GCF/000/412/655/GCF_000412655.1_ASM412
Hipposideros armiger	ftp://ftp.ncbi.nlm.nih.gov/genomes/all/GCF/001/890/085/GCF_001890085.1_ASM18900

Table S13

Drivers of parallel molecular evolution for the genes: *ACCS*, *ALKBH7* and *UNC-45 B*, for the fruit-bats (Pteropodids), pollen-nectar feeder bats (Glossophaginae), and other bats.

Species	*R. aegyptiacus*	*P. alecto*	*P. vampyrus*	*M. harrisoni*	*L. nivalis*	*L. yerbabuenae*	Other bats	Human
Gene	ACCS<br>ALKBH7<br>UNC-45 B	ACCS<br>ALKBH7<br>UNC-45 B	ACCS<br>ALKBH7<br>UNC-45 B	ACCS<br>ALKBH7<br>UNC-45 B	ACCS<br>ALKBH7<br>UNC-45B	ACCS<br>ALKBH7<br>UNC-45 B	ACCS<br>ALKBH7<br>UNC-45 B	ACCS<br>ALKBH7<br>UNC-45 B
Length DNA	2,016<br>663<br>2,787	1,890<br>663<br>2,787	 663<br>2,787	1,986<br>663<br>2,799	1,938<br>663<br>2,799	1,986<br>663<br>2,787	2,016<br>663<br>2,799	2,016<br>663<br>2,787
% GC	65.48%<br>71.49%<br>64.26%	64.34%<br>70.74%<br>64.30%	 70.59%<br>64.20%	65.31%<br>70.29%<br>64.85%	64.96%<br>70.44%<br>64.45%	65.56%<br>69.83%<br>64.80%	65.53%<br>70.14%<br>64.59%	64.29%<br>70.59%<br>64.66%
Length Protein	672<br>221<br>929	630<br>221<br>929	 221<br>720	662<br>221<br>933	642<br>221<br>933	662<br>221<br>929	672<br>221<br>933	672<br>221<br>929
Isoelectric point	5.96<br>6.89<br>6.85	6.11<br>8.08<br>7.09	 8.08<br>7.35	5.76<br>7.3<br>6.92	5.72<br>6.37<br>6.82	5.97<br>7.69<br>6.83	5.92<br>7.51<br>7	5.63<br>6.37<br>7.03

Table S14

Gene and proteins abbreviations

Abbreviation	Name
TREH	Trehalase
CHIA	Acid mammalian chitinase
PFKP	Phosphofructokinase
ADPRM	Manganese-dependent ADP-ribose/CDP-alcohol diphosphatase
CS	Citrate synthase
PYGM	Glycogen phosphorylase
LPL	Lipoprotein lipase
PNLIP	Pancreatic triacylglycerol lipase
APOA2	Apoliprotein A-II
BDH2	3-hydroxybutyrate dehydrogenase type 2
CYP7A1	Cholesterol 7-alpha-monooxygenase
THBD	Thrombomodulin
THPO	Thrombopoietin
A2M	Alpha-2-macroglobulin
ALPI	Intestinal-type alkaline phosphatase
UNC-45 B	Unc-45 Myosin Chaperone B
AACS	Acetoacetyl-CoA synthethase
ALKBH7	Alpha-Ketoglutarate-Dependent Dioxygenase Homolog 7
MGAT2	Alpha-1,6-Mannosyl-Glycoprotein 2-Beta-N-Acetylglucosaminyltransferase
AMPK	AMP-activated protein kinase
FABP1	Fatty Acid Binding Protein 1
APOBR	Apolipoprotein B Receptor
PLA2G16	Phospholipase
GFOD1	Glucose-Fructose Oxidoreductase Domain Containing 1
Fslt1	Follistation-related protein 1
FTL	Ferretin light chain
CALPS	Calcyphosin
CD248	Endosialin
