# Supplementary material for: Genomic consequences of dietary diversification and parallel evolution due to nectarivory in leaf-nosed bats
Source: Gigascience. 2020 Jun 6;9(6):giaa059. doi: 10.1093/gigascience/giaa059 (PMC7276932; doi:10.1093/gigascience/giaa059)
Supplement: giaa059_Response_to_Reviewer_Comments_Original_Submission [file giaa059_response_to_reviewer_comments_original_submission.pdf]

Author's Response To Reviewer Comments

Close

Reviewer's Comments

This study has sequenced and assembled the genomes of a number of bats showing diverse feeding strategies, and explored the evolutionary adaptations underpinning dietary niche specialization. The authors have fully addressed my initial concerns regarding selection tests, have provided a wealth of data to support their findings and have also provided an incredibly thorough guide on their methods that I think will benefit many researchers. I have some small, largely trivial issues below. Most these are concerned with the written English rather than the methods used. I am therefore happy to support this paper for publication, pending these minor changes.

We appreciate your support and comments.
We are certain that your suggestions have been crucial to improve and make more accurate our manuscript.
In particular, we appreciate your remark on the changes in description of the methods, that we hope will be useful.

Minor points

Line 163: insects -> insect's

Thank you for your correction, we made the change.
Line 165

Line 163: I feel like trehalOse should be the sugar in insect blood, rather than trehalase, if trehalase is the enzyme that degrades it.

Thank you for your observation, we are talking about trehalose, we are sorry for the mistake.
Line 165

Line 231: those than -> those that

Thank you, we changed it.
Line 232

Line 233: This relates to my comment on line 163. Does trehalase digest trehalase in insects, such that one enzyme degrades another enzyme, or does trehalase degrade the trehalose sugar?

We appreciate your comment, as it is very relevant. Most of the vertebrates have the capacity to digest dietary trehalose with the membrane bound intestinal enzyme trehalase.
We have modified this section.
Lines 234-235

Line 233: Do the authors have any ideas as to why the ability to digest insects may be maintained in bats, nit not the ability to digest the trehalase/trehalose sugar/enzyme in insect blood?

This issue is really interesting, as a parallel change seem to have happened in birds. Even those specialist bats, such as hematophagous and nectar-feeding species have the capacity to digest insects exoskeletal chitin. We consider two possibilities for the loss of trehalase. One is that the main dietary value of the insects is for lipids and proteins, and energy (as sugars) would be less important, and once the ability to digest trehalose is loss, there is no way they can recuperate it. On the other hand, we suggest that gut microbiome plays an important role to digest trehalose. The microbiome role is discussed in line 291-296.

Line 260: that it may -> that may

Thank you.
Line 262

Line 277: When the authors mention convergent evolution here, do they mean specifically dietary genes or the genome and physiology of the bat as a whole? Please clarify.

Thank you. We meant specifically parallel evolution due to nectar-feeding dietary specialization.

"Our findings suggest that parallel evolution due to nectar-feeding dietary specialization is likely a consequence of high metabolic demands required for foraging on flowers and fruits."
Lines 279-280

Line 318: I have not seen 'accurate' used in the context the authors use it here. Perhaps another word such as 'validate'can be used instead?

We apologize for the mistake.

" To optimize and extend the genome assembly"
Lines 321-322

Line 354 Perhaps consider "Repeatmasker pipeline" rather than "pipeline of repeatmasker"

We appreciate your suggestion.
Line 358

Line 373: I think "proteins" should be "protein's"

Thank you.
Line 377

Line 373: DIAMOND is also a program, so consider saying "programs DIAMOND and Proteinortho"

Thank you, we made the change.
Line 381

Line 380: "paralogous, sequences" -> "paralogous sequences"

Thank you, we made the change.
Line 383

Line 384: Were the poorly aligned regions removed based on a visual inspection or something like Gblocks?

We carried out a visual inspection and calculated the alignment length with a bash script.

"Each cluster was aligned with MAFFT aligner tool (67), we retained alignment sequences where the length is within 80 to 120% relative to the human and mouse sequences, and poorly aligned regions were removed by a visual inspection. "
Line 386-388

Line 387: I think "RAxML tool" can just be "RaxML"

We appreciate your suggestion.
Line 391
Line 390: The authors describe how they calculated "synonymous sites and nonsynonymous sites (dN/dS) rates, and the average ratio of substitution per site (ω=dN/dS)", however I would have assumed that these were essentially the same things, and don't need to be stated twice as it is written, at least as far as dN/dS and w=dN/dS is concerned.
Thank you for your observation, we estimated the ratio of substitution per site.
Line 395

Line 402: No need for the “,” after the word aBSREL.

Thank you.
Line 407

Line 417: “was composed from 12 to maximum 30” -> “was composed of between 12 and a maximum 30” perhaps?

Thank you for your suggestion.
Lines 422-423

Line 424: “the accurate” –> “ the accurate ones”

Thank you.
Line 429

Line 424: “program” -> “programs”

Thank you.
Line 430

Line 442: “Independantly” –> “Independent”

Thank you.
Line 453

Line 446: The phylogenetic tree section seems out of context here, as trees have been generated throughout the methods up to this point. The authors should consider moving this section or being explicit as to the function of the tree generated in this section.

Thank you, we re-ordenized this section.
Lines 446-450

Line 451: The authors should consider adding one line at the start to give context for the reasoning behind modelling, for example “To explore the effects of selected sites on the protein 3D structure..” or something similar.

We appreciate your suggestion.

“To explore the effects of positive selection and the radical amino acid substitutions, we modeled the second and tertiary structure of the protein Acetoacetyl CoA Synthetase (ACCS) for M. waterhousii, D. rotundus, M. harrisoni, L. nivalis, L. yerbabuenae and P. alecto. ”
Lines 459-461

Table 2: there was an odd symbol in the brackets under nucleotide diversity on my screen. Double check that it is not an error!

Thank you, we modified this section.

Figure 1:Purely out of curiosity, do the major expansion events correlate with known climate events occurring in the various geological epochs?

This is an interesting question., but we have not formally explored this. In the case of the nectar-pollen feeder clade, we found an important gene family expansion event. This is interesting, because the divergence of the Glossophagini bats started in the Mid-Miocene from 21 to 7 Mya, coinciding with some environmental changes and the increase of food resources at the “Climatic Optimum” period.

On the other hand, the major gene family expansion was detected at the Microchiroptera node, in the Eocene period, where the Earth responded to higher levels of carbon dioxide and an increment in the temperature, warmer than today.

We will analyze in detail these gene families expansions in a future manuscript, incorporating some

analysis such as phylostratigraphy and gene family calibration. Thank you for the comment.

Additional File 1, Table S1-6: Some numbers have "," in them, others don't. Please ensure they all do.

We apologize for the mistake, we made the change.

Table S6: please change LTR to LRT. Are these p-values corrected for multiple testing? It would also be helpful to highlight significant ones with a "*" or something similar.

We included a column with the p-values adjust by FDR and we highlighted those significant genes.

Reviewer #2: The authors made a great effort to make changes in this revision based on reviewers' comments. I generally agree with the authors for their responses to my previous comments. However, as I look through the whole MS, I found many minor errors which can be avoided if authors are meticulous during writing. So I strongly recommend the authors to reread the whole MS carefully to correct possible minor errors.

We appreciate your support and comments. We have read carefully all the manuscript, and double-checked.

Below are some examples.

In "Rapidly evolving genes across the whole genome", the authors did not provide the specific total number of positively selected genes, and also some words about enrichment analysis.

We appreciate your observation, we have incorporated more information.
Lines 151-155.

"For all Phyllostomid bats, we identified 42 genes with robust signals of positive selection (FDR p < 0.05). According with the enrichment analysis, most of the adaptive genes are related to immune response, DNA repair, inflammatory response, RNA catalytic process and genes that mediate muscle function (such as Myoblast and PAMR1) (Fig. 2; see Additional file 1, Table S6-TableS8) (19)."

In Table S6, LTR is still used (another reviewer had pointed out this mistake).

We deeply apologize for this repeated mistake.
We changed LTR to LRT.

In Additional file 1, "Table S7" was wrote as "Table S8", so there are two "Table S8".

We are sorry and we changed the number of this figures.

"Table S6. LTR construction and ω ratio", I did not see results aboutω ratio, but just P values.

We appreciate your observation.
We have incorporated the p-value correction and highlighted those significant genes.

Table S7 "GO enrichment for those positive selected genes for each Phyllostomid specie", the last word should be "species"

Thank you, we modified it.

Line 231, "than" should be "that"

We apologize for the mistake, we change it.
Line 232

Line 359, what software was used to construct the phylogeny based a total of 132 genes? I find it in the additional file 3, PhyML3. I think that the authors should mention this in the main text. In addition, the authors did not mention that whether these 132 genes are concatenated or not in building the tree.

Thank you, we included the information in the main text.

“A total of 132 single-copy orthologous genes (61,331 amino acids sites), across 18 mammals were concatenated to reconstruct a phylogenomic tree (best-fit model distribution JTT, +G +I +I+G and 80% consensus threshold) using PhyML3 (62) (see Additional file 3, Methods). ”
Lines 362-364

Line 387, RaxML

Thank you, we changed RaxML to RAxML.
Line 391

Line 442, "independently" should be "independent"

Thank you, we modified it.
Line 453

Line 450, no parameters are provided for RAxML analysis.

Thank you, we included the parameters used in the analysis

“ The phylogenetic tree was constructed using a Maximum Likelihood method with RAxML (-p 12345 -m PROTCATLG).”
Lines 449-450

Line 707, genes

We apologize for this mistake.
Line 721

Close
