# Supplementary material for: Genomic consequences of dietary diversification and parallel evolution due to nectarivory in leaf-nosed bats
Source: Gigascience. 2020 Jun 6;9(6):giaa059. doi: 10.1093/gigascience/giaa059 (PMC7276932; doi:10.1093/gigascience/giaa059)
Supplement: giaa059_Reviewer_1_Report_Original_Submission — Graham Hughes -- 4/16/2020 Reviewed [file giaa059_reviewer_1_report_original_submission.pdf]

Reviewer Report

Title: Genomic consequences of dietary diversification and parallel evolution due to nectarivory in Leaf-nosed bats.

Version: Original Submission **Date:** 4/16/2020

Reviewer name: Graham Hughes

Reviewer Comments to Author:

Reviewer's comments
This study has sequenced and assembled the genomes of a number of bats showing diverse feeding strategies, and explored the evolutionary adaptations underpinning dietary niche specialization. The authors have fully addressed my initial concerns regarding selection tests, have provided a wealth of data to support their findings and have also provided an incredibly thorough guide on their methods that I think will benefit many researchers. I have some small, largely trivial issues below. Most these are concerned with the written English rather than the methods used. I am therefore happy to support this paper for publication, pending these minor changes.
Minor points
Line 163: insects -> insect's
Line 163: I feel like trehalOse should be the sugar in insect blood, rather than trehalase, if trehalase is the enzyme that degrades it.
Line 231: those than -> those that
Line 233: This relates to my comment on line 163. Does trehalase digest trehalase in insects, such that one enzyme degrades another enzyme, or does trehalase degrade the trehalose sugar?
Line 233: Do the authors have any ideas as to why the ability to digest insects may be maintained in bats, nit not the ability to digest the trehalase/trehalose sugar/enzyme in insect blood?
Line 260: that it may -> that may
Line 277: When the authors mention convergent evolution here, do they mean specifically dietary genes or the genome and physiology of the bat as a whole? Please clarify.
Line 318: I have not seen 'accurate' used in the context the authors use it here. Perhaps another word such as 'validate'can be used instead?
Line 354 Perhaps consider "Repeatmasker pipeline" rather than "pipeline of repeatmasker"
Line 373: I think "proteins" should be "protein's"
Line 373: DIAMOND is also a program, so consider saying "programs DIAMOND and Proteinortho"
Line 380: "paralogous, sequences" -> "paralogous sequences"
Line 384: Were the poorly aligned regions removed based on a visual inspection or something like Gblocks?
Line 387: I think "RAxML tool" can just be "RAxML"
Line 390: The authors describe how they calculated "synonymous sites and nonsynonymous sites (dN/dS) rates, and the average ratio of substitution per site (Ï‰=dN/dS)", however I would have

assumed that these were essentially the same things, and don't need to be stated twice as it is written, at least as far as dN/dS and w=dN/dS is concerned.
Line 402: No need for the "," after the word aBSREL.
Line 417: "was composed from 12 to maximum 30" -> "was composed of between 12 and a maximum 30" perhaps?
Line 424: "the accurate" -> " the accurate ones"
Line 424: "program" -> "programs"
Line 442: "Independantly" -> "Independent"
Line 446: The phylogenetic tree section seems out of context here, as trees have been generated throughout the methods up to this point. The authors should consider moving this section or being explicit as to the function of the tree generated in this section.
Line 451: The authors should consider adding one line at the start to give context for the reasoning behind modelling, for example "To explore the effects of selected sites on the protein 3D structure.." or something similar.
Table 2: there was an odd symbol in the brackets under nucleotide diversity on my screen. Double check that it is not an error!
Figure 1:Purely out of curiosity, do the major expansion events correlate with known climate events occurring in the various geological epochs?
Additional File 1, Table S1-6: Some numbers have "," in them, others don't. Please ensure they all do.
Table S6: please change LTR to LRT. Are these p-values corrected for multiple testing? It would also be helpful to highlight significant ones with a "*" or something similar.

Methods

Are the methods appropriate to the aims of the study, are they well described, and are necessary controls included? Choose an item.

Conclusions

Are the conclusions adequately supported by the data shown? Choose an item.

Reporting Standards

Does the manuscript adhere to the journal's guidelines on [minimum standards of reporting?](#) Choose an item.

Choose an item.

Statistics

Are you able to assess all statistics in the manuscript, including the appropriateness of statistical tests used? Choose an item.

Quality of Written English

Please indicate the quality of language in the manuscript: Choose an item.

Declaration of Competing Interests

Please complete a declaration of competing interests, considering the following questions:

- Have you in the past five years received reimbursements, fees, funding, or salary from an organisation that may in any way gain or lose financially from the publication of this manuscript, either now or in the future?
- Do you hold any stocks or shares in an organisation that may in any way gain or lose financially from the publication of this manuscript, either now or in the future?
- Do you hold or are you currently applying for any patents relating to the content of the manuscript?
- Have you received reimbursements, fees, funding, or salary from an organization that holds or has applied for patents relating to the content of the manuscript?
- Do you have any other financial competing interests?
- Do you have any non-financial competing interests in relation to this paper?

If you can answer no to all of the above, write 'I declare that I have no competing interests' below. If your reply is yes to any, please give details below.

I declare that I have no competing interests

I agree to the open peer review policy of the journal. I understand that my name will be included on my report to the authors and, if the manuscript is accepted for publication, my named report including any attachments I upload will be posted on the website along with the authors' responses. I agree for my report to be made available under an Open Access Creative Commons CC-BY license (http://creativecommons.org/licenses/by/4.0/). I understand that any comments which I do not wish to be included in my named report can be included as confidential comments to the editors, which will not be published.

Choose an item.

To further support our reviewers, we have joined with Publons, where you can gain additional credit to further highlight your hard work (see: https://publons.com/journal/530/gigascience). On publication of this paper, your review will be automatically added to Publons, you can then choose whether or not to claim your Publons credit. I understand this statement.

Yes Choose an item.
