# Supplementary material for: Genomic consequences of dietary diversification and parallel evolution due to nectarivory in leaf-nosed bats
Source: Gigascience. 2020 Jun 6;9(6):giaa059. doi: 10.1093/gigascience/giaa059 (PMC7276932; doi:10.1093/gigascience/giaa059)
Supplement: giaa059_Reviewer_2_Report_Original_Submission — Xiuguang Mao -- 4/17/2020 Reviewed [file giaa059_reviewer_2_report_original_submission.pdf]

Reviewer Report

Title: Genomic consequences of dietary diversification and parallel evolution due to nectarivory in Leaf-nosed bats.

Version: Original Submission **Date:** 4/17/2020

Reviewer name: Xiuguang Mao

Reviewer Comments to Author:

The authors made a great effort to make changes in this revision based on reviewers' comments. I generally agree with the authors for their responses to my previous comments. However, as I look through the whole MS, I found many minor errors which can be avoided if authors are meticulous during writing. So I strongly recommend the authors to reread the whole MS carefully to correct possible minor errors. Below are some examples.
In "Rapidly evolving genes across the whole genome", the authors did not provide the specific total number of positively selected genes, and also some words about enrichment analysis. In Table S6, LTR is still used (another reviewer had pointed out this mistake). In Additional file 1, "Table S7" was wrote as "Table S8", so there are two "Table S8". "Table S6. LTR construction and Ï‰ ratio", I did not see results aboutÏ‰ ratio, but just P values. Table S7 "GO enrichment for those positive selected genes for each Phyllostomid specie", the last word should be "species"
Line 231, "than" should be "that"
Line 359, what software was used to construct the phylogeny based a total of 132 genes? I find it in the additional file 3, PhyML3. I think that the authors should mention this in the main text. In addition, the authors did not mention that whether these 132 genes are concatenated or not in building the tree.
Line 387, RaxML
Line 442, "independently" should be "independent"
Line 450, no parameters are provided for RAxML analysis.
Line 707, genes

Methods

Are the methods appropriate to the aims of the study, are they well described, and are necessary controls included? Choose an item.

Conclusions

Are the conclusions adequately supported by the data shown? Choose an item.

Reporting Standards

Does the manuscript adhere to the journal's guidelines on [minimum standards of reporting?]() Choose an item.

Choose an item.

Statistics

Are you able to assess all statistics in the manuscript, including the appropriateness of statistical tests used? Choose an item.

Quality of Written English

Please indicate the quality of language in the manuscript: Choose an item.

Declaration of Competing Interests

Please complete a declaration of competing interests, considering the following questions:

- Have you in the past five years received reimbursements, fees, funding, or salary from an organisation that may in any way gain or lose financially from the publication of this manuscript, either now or in the future?
- Do you hold any stocks or shares in an organisation that may in any way gain or lose financially from the publication of this manuscript, either now or in the future?
- Do you hold or are you currently applying for any patents relating to the content of the manuscript?
- Have you received reimbursements, fees, funding, or salary from an organization that holds or has applied for patents relating to the content of the manuscript?
- Do you have any other financial competing interests?
- Do you have any non-financial competing interests in relation to this paper?

If you can answer no to all of the above, write 'I declare that I have no competing interests' below. If your reply is yes to any, please give details below.

I declare that I have no competing interests

I agree to the open peer review policy of the journal. I understand that my name will be included on my report to the authors and, if the manuscript is accepted for publication, my named report including any attachments I upload will be posted on the website along with the authors' responses. I agree for my report to be made available under an Open Access Creative Commons CC-BY license (http://creativecommons.org/licenses/by/4.0/). I understand that any comments which I do not wish to be included in my named report can be included as confidential comments to the editors, which will not be published.

Choose an item.

To further support our reviewers, we have joined with Publons, where you can gain additional credit to further highlight your hard work (see: https://publons.com/journal/530/gigascience). On publication of this paper, your review will be automatically added to Publons, you can then choose whether or not to claim your Publons credit. I understand this statement.

Yes Choose an item.
